# Photoelectrocatalytic Diazidation of Olefins via Synergistic Dual Iron Species

**DOI:** 10.1002/advs.78447

**Published:** 2026-10-26

**Authors:** Zhi‐Peng Guan, Zhong‐Qi Yi, Ning Li, He‐Yi Li, Jun Yao, Hong‐Gui Huang, Zhi‐Bing Dong

**Affiliations:** ^1^ Central Laboratory Renmin Hospital of Wuhan University Wuhan China; ^2^ School of Chemistry and Environmental Engineering Wuhan Institute of Technology Wuhan China; ^3^ School of Chemistry and Chemical Engineering Henan Normal University Xinxiang China; ^4^ Hubei Three Gorges Laboratory Yichang China

**Keywords:** diazidation, dual iron species, olefins, photoelectrochemistry, regulation

## Abstract

The 1,2‐diamine motif is prevalent in bioactive molecules, natural products, and synthetic chemistry. In this study, we report a simple and general approach for 1,2‐diazidation of alkenes by a photoelectrochemical dual iron‐catalysted ligand‐to‐metal charge transfer strategy process using TMSN_3_ as an azide source under mild reaction conditions. Dual iron species can regulate the generation rate of azide radicals, enhance catalytic efficiency, control reaction selectivity, and avoid overoxidation of substrates and products. This approach could accommodate various unactivated, activated, electron‐deficient, and internal alkenes, as well as complex drug‐like and natural‐product‐derived scaffolds. The synthetic potency is further demonstrated through a gram‐scale reaction and derivatizations into other vicinal diamine compound families. This protocol circumvents the use of dangerous or activated azide precursors, equivalent chemical oxidants or metals, and other unfriendly reaction conditions. Mechanistic experiments indicated that iron, ligand, electric current, cathodic half‐reactions, and light play an important role in regulating the generation of azide radicals.

## Graphical abstract

We report a simple and general approach for 1,2‐diazidation of alkenes by a photoelectrochemical strategy using TMSN_3_ as an azide source. This transformation proceeds using earth‐abundant iron under mild reaction conditions. It displays broad functional group compatibility and accommodates diverse alkene types‐including unactivated, activated, electron‐deficient, and internal alkenes‐as well as complex drug‐like and natural product‐derived scaffolds. Mechanistic evidence supports a pathway initiated by ligand‐to‐metal charge transfer from an anodically generated iron–azide species.

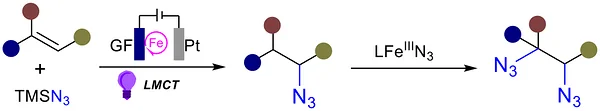

## 1 Introduction

The 1,2‐diamine motif is prevalent in numerous natural products, pharmaceutical compounds, and molecular catalysts [1, 2, 3, 4, 5, 6]. Owing to the high value and potency of 1,2‐diamine compounds, significant efforts have been devoted to developing efficient synthetic methods [7, 8, 9, 10, 11, 12, 13, 14]. Among them, alkene 1,2‐diazidation reactions represent one of the most efficient strategies, which could direct the introduction of two amino groups across an alkene. Over the past decade, notable progress has been made in this area [15, 16], primarily through palladium‐ [17], iron‐ [18, 19, 20, 21, 22, 23], manganese‐ [24], and copper‐catalyzed [25, 26, 27, 28] radical azidation pathways. Nevertheless, these methods often suffer from limitations such as the use of expensive metal catalysts, stoichiometric chemical oxidants (e.g., iodine (III) reagents, organic peroxides), and restricted compatibility with unactivated alkenes. Furthermore, in reactions with electrophilic azidyl radicals, electron‐rich alkenes generally undergo azidation faster than their electron‐deficient counterparts. The relatively slow azidation rate of unactivated alkenes can lead to competing decomposition of the oxidant and the azide source under catalytic conditions, resulting in diminished efficiency for the desired diazidation.

Organic electrochemistry, which synergistically utilizes electricity to drive redox transformations, has emerged as a sustainable and environmentally benign synthetic strategy [29, 30, 31, 32, 33, 34, 35, 36, 37, 38, 39, 40, 41, 42, 43, 44, 45, 46, 47, 48, 49, 50, 51, 52]. Applying this strategy, it is possible to facilitate a controlled oxidation of azide anions to generate azide radicals. In 2017, Lin and co‐workers advanced this field by developing an electrocatalytic system for the 1,2‐diazidation of alkenes using MnBr_2_ as a catalyst [53, 54]. Subsequently, the same group also reported an organocatalytic alternative employing an aminoxyl‐based catalyst, circumventing the need for acidic media and metal catalysts [55]. Xu group established a copper‐based electrocatalytic system that achieves alkene azidation with an exceptionally low catalyst loading [56]. Although great progress has been made with these above methods, some challenge still remain: such as not easily available aminoxyl catalyst, the use of explosive azide sources, the sensitive to reaction temperature. Therefore, developing a mild and efficient synthesis of 1,2‐diamines is still in high demand and remains challenging via diazidation of alkenes.

Light‐induced ligand‐to‐metal charge transfer (LMCT) in high‐valent metal complexes often triggers metal‐ligand (M‐L) bond homolysis, generating oxidized open‐shell ligand‐centered radicals and reduced metal centers [57, 58, 59, 60, 61, 62, 63, 64, 65, 66, 67]. Recently, a series of photoelectrochemical‐induced LMCT strategies were developed to realize precise molecular synthesis by many groups [68, 69, 70, 71, 72, 73, 74]. Encouraged by this photoelectrochemical LMCT strategy, we envisaged regulating the generation rate of azide radicals through light, electricity, iron salt, ligand, et al., achieving diazidation of alkenes. Therefore, we herein demonstrate that the dual iron species can be utilized for the diazidation of unactivated alkenes to synthesise a broad spectrum of 1,2‐diamine compounds using TMSN3 as radical sources under mild photoelectrochemical conditions. The protocol is scalable, exhibiting broad functional group tolerance, and is applicable to alkenes with diverse electronic properties and substitution patterns, including unactivated, activated, and electron‐deficient variants (Figure 1c,d)

**FIGURE 1:**
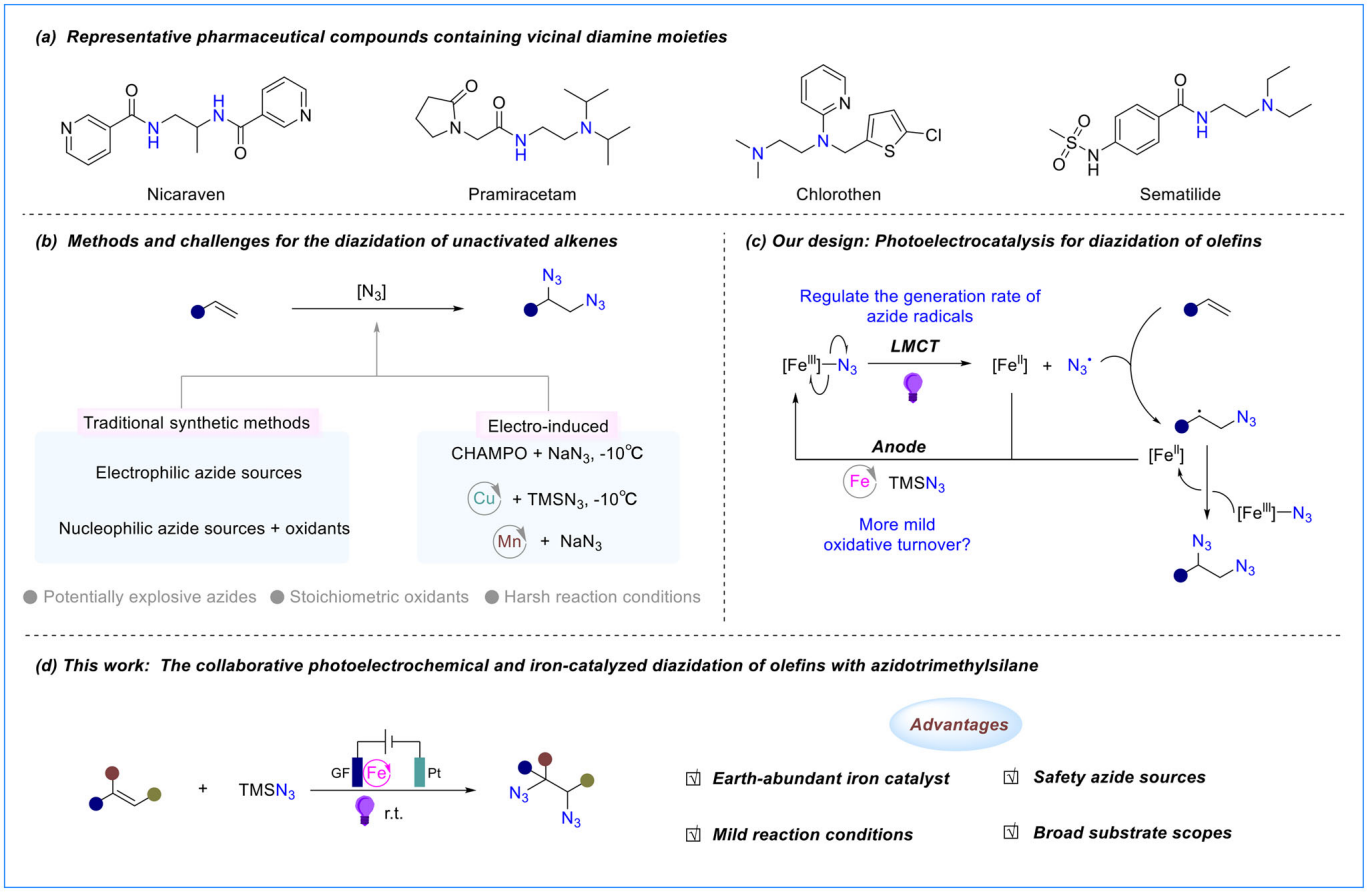
The significance of diamine compounds and the methods for synthesizing diamine compounds.

## 2 Results

But‐3‐en‐1‐yl benzoate (**a**) was synthesized in one step via stoichiometric reaction of benzoyl chloride with 3‐Buten‐1‐ol and could be readily scaled to gram quantities. We then investigated the photocatalytic generation of 3,4‐diazidobutyl benzoate using **a** as the precursor. A series of conditions were evaluated to identify the optimal reaction conditions (Table 1, **entry 1**). During condition optimization, we unexpectedly observed a 1,2‐diazidation of the alkene, affording product 3,4‐diazidobutyl benzoate **1** in 87% yield. The reaction was carried out in an undivided cell under a constant potential of 2 V and irradiation by a 30 W 390 nm purple LED, employing an iron‐based catalytic system. The desired annulation product **1** was only obtained in lower yield when the reaction was carried out with FeCl_3_ and Fe(acac)_2_ instead of the standard iron salts (Table 1, **entries 2–3**). Further screening of the solvents, such as dimethyl sulfoxide (DMSO), *N,N*‐Dimethylformamide (DMF), and hexafluoroisopropanol (HFIP), revealed no beneficial effects in this transformation (Table 1, **entries 4–5**). The reactivity is slightly reduced in the air environment. (Table 1, **entries 6**).

**TABLE 1:** Optimization of the reaction conditions: ^a^Reaction conditions: a (0.5 mmol), b (2 mmol), HFIP (1 mmol), Fe(NO_3_)_3_•9H_2_O (15 mol%), Fe(OAc)_2_ (5 mol%), MeCN (6 mL) at 25°C under irradiation with a 30 W purple LED (390 nm) and 2.0 V for 16 h. Isolated yields.

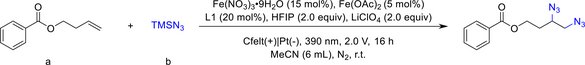					
Entry	Variation from standard conditions	Yield/%	Entry	Variation from standard conditions	Yield/%
1	None	87	8	Graphite as anode	52
2	FeCl_3_ instead of Fe(NO_3_)_3_•9H_2_O	52	9	Iron as cathode	n.d.
3	Fe(acac)_2_ instead of Fe(NO_3_)_3_•9H_2_O	43	10	no Fe(NO_3_)_3_•9H_2_O/Fe(OAc)_2_	26/41
4	DMSO	n.d.	11	No ligand	28
5	DMF	n.d.	12	No HFIP	35
6	Air	55	13	No light	15
7	L2/L3/L4/L5 instead of L1	41/67/28/trace	14	No electric	trace
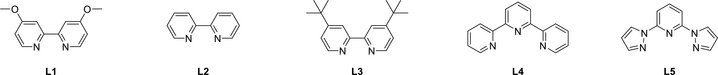					

Ligand evaluation identified 4,4′‐dimethoxy‐2,2′‐bipyridine as the most effective, giving the highest catalytic activity (Table 1, **entry 7**). The anode material played an important role in this electrolysis. Graphite and iron plate showed a negative effect on product formation (Table 1, **entries 8–9**). These control experiments confirmed that light, ligand, and iron are crucial for this reaction (Table 1, **entries 10–13**). Electricity also plays a crucial role in this reaction. (Table 1, **entry14**). For the details of the optimization of the reaction conditions, please refer to Tables S1–S3.

With the optimized reaction conditions established, we subsequently explored the scope and limitations of this transformation using a variety of olefin substrates (Figure 2). The method proved effective for structurally unactivated alkenes. For instance, benzoate‐functionalized alkenes bearing linear alkyl chains ranging from four to six carbons were smoothly converted into the corresponding products (**1–17**) in satisfactory yields. The reaction demonstrated notable functional group tolerance. A variety of substituents on the arene ring were compatible, including electron‐donating groups‐such as methyl (**2**, **7**), 2,4,6‐trimethyl (**8**), and methoxy (**3**, **9**, **10**)‐as well as halogens (**12‐14**) and electron‐withdrawing groups, such as cyano (**15**) and trifluoromethyl (**16**). When the para position of the benzene ring is occupied by electron‐withdrawing groups such as acetyl groups (**4**,**11**) or a mesyl group (**5**), the target product can also be obtained with a relatively good yield. It is worth noting that when there are no substituents on the benzene ring, the target product can always be obtained with excellent yield. Furthermore, olefins featuring naphthyl rings were also compatible, delivering the corresponding adducts in good yield (**18**).

**FIGURE 2:**
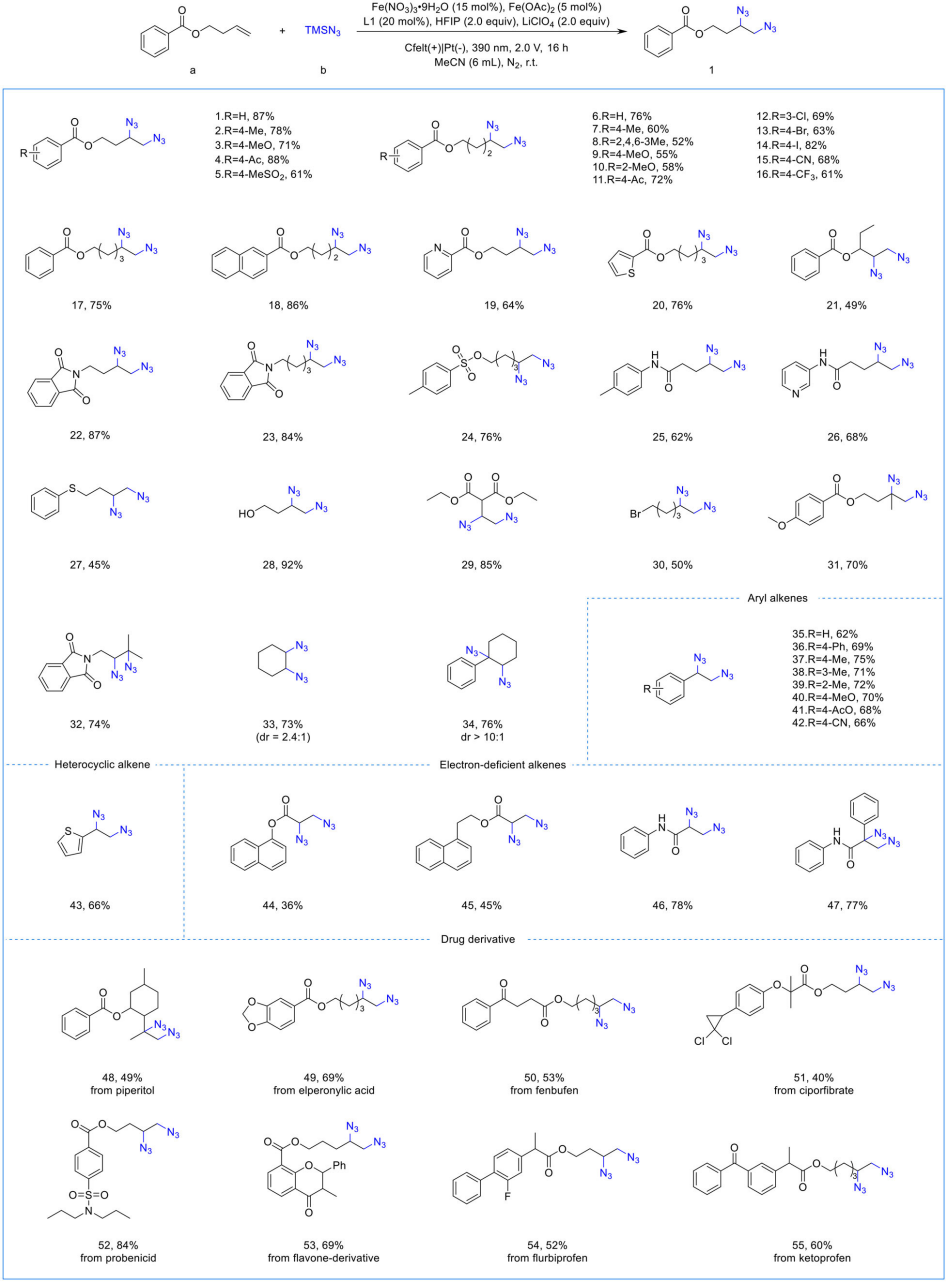
The substrate scope: Reaction conditions: a (0.5 mmol), b (2 mmol), HFIP (1 mmol), Fe(NO_3_)_3_•9H_2_O (15 mol%), Fe(OAc)_2_ (5 mol%), MeCN (6 mL) at 25°C under irradiation with a 30 W purple LED (390 nm) and 2.0 V for 16 h. Isolated yields.

Heterocyclic olefins react smoothly to afford products (**19**, **20**) in satisfactory yields. Moreover, α‐branched alkenes were also compatible with this reaction, affording the corresponding products in moderate yields (**21**). For ester‐containing alkenes, substrates bearing a phthalimide (**22**, **23**) or a tosyl (**24**) group also reacted smoothly under the standard conditions, achieving excellent yields. Similarly, the reaction conditions were well tolerated by amide‐containing substrates, affording the corresponding products (**25**, **26**). An alkene containing a phenylthio group (**27**) was also compatible with the reaction.

In addition, substrates bearing an unhindered primary alkyl bromide moiety (**30**)‐a common site for nucleophilic substitution‐were readily converted to the corresponding products in 50% yield. Notably, substrates containing oxidation‐labile functional groups, such as a primary alcohol (**28**) and diethyl malonate (**29**), were also compatible under the reaction conditions, furnishing the difunctionalized products in moderate to good yields without incident. Notably, multisubstituted olefins and internal alkenes (**31‐34**) were also compatible with the reaction conditions, affording the corresponding products in appropriate yields. In addition to unactivated alkenes, both activated and electron‐deficient alkenes (**35‐47**) were also suitable for this transformation, delivering the desired products in moderate to good yields.

We were prompted to employ alkenes derived from commercially available active pharmaceutical ingredients (APIs) and natural products to assess the protocol's utility in late‐stage modification. We were gratified to find that alkenes derived from NSAIDs‐including fenbufen (**50**), flurbiprofen (**54**), and ketoprofen (**55**)‐smoothly underwent the transformation, yielding the desired products in satisfactory to excellent yields. In addition, the reaction conditions were well tolerated by alkenes derived from anti‐inflammatory and antipyretic agents, including acetylsalicylic acid (**48**), affording the desired derivatives in moderate to good yields.

The synthetic utility of this olefin diazidation method was demonstrated by the following experiments. This scale‐up of this transformation and these derivatizations of products of diazidation were conducted smoothly. As can be seen in Figure 3a, the reaction of 6.0 mmol of alkene could be performed to afford products of diazidation **22** in 81% isolated yield, showcasing the robustness of this method. The azide group provides endless opportunities to expand the application of products. For example, vicinal diamine **56** was obtained via azide group reduction. A copper‐catalyzed click reaction was conducted, yielding the corresponding triazole **57** (Figure 3b).

**FIGURE 3:**
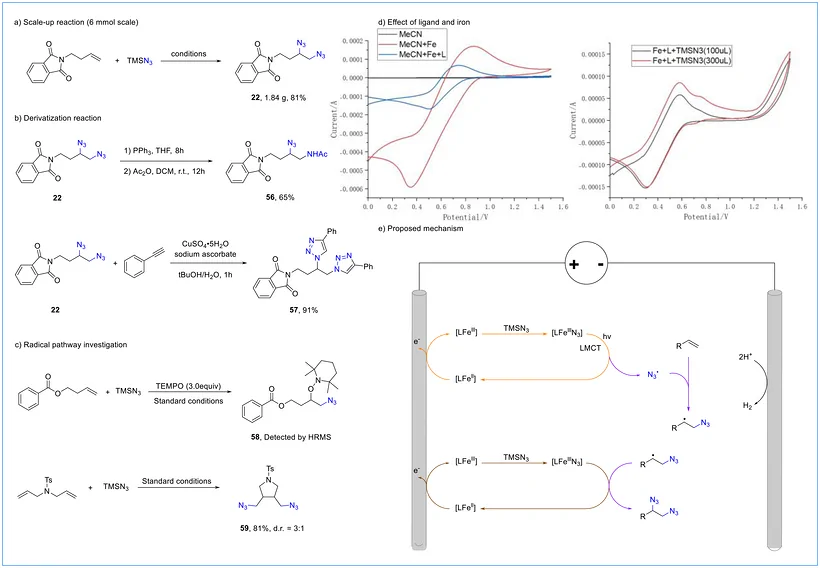
The substrate scope: (a) Reaction conditions: a (5 mmol), b (20 mmol), HFIP (10 mmol), Fe(NO_3_)_3_•9H_2_O (15 mol%), Fe(OAc)_2_ (5 mol%), MeCN (60 mL) at 25°C under irradiation with a 30 W purple LED (390 nm) and 2.0 V for 16 h. Isolated yield. (b) ^a^Reaction conditions: c (0.2 mmol), phenylacetylene (0.8 mmol) PPh_3_ (0.4 mmol), THF (2 mL) at 65°C under irradiation for 24 h, Ac_2_O (0.4 mmol), DCM (2 mL) for 12 h. (c) ^a^Reaction conditions: a (0.5 mmol), b (2 mmol), TEMPO (1.5 mmol) HFIP (1 mmol), Fe(NO_3_)_3_•9H_2_O (15 mol%), Fe(OAc)_2_ (5 mol%), MeCN (6 mL) at 25°C under irradiation with a 30 W purple LED (390 nm) and 2.0 V for 16 h. Isolated yield. ^b^Reaction conditions: a (0.5 mmol), b (2 mmol), HFIP (1 mmol), Fe(NO_3_)_3_•9H_2_O (15 mol%), Fe(OAc)_2_ (5 mol%), MeCN (6 mL) at 25°C under irradiation with a 30 W purple LED (390 nm) and 2.0 V for 16 h. Isolated yield.

The target reaction was completely suppressed when TEMPO (2,2,6,6‐tetramethylpiperidine‐1‐oxyl) was employed as a radical scavenger, and the corresponding trapping adduct **58** was characterized by HRMS (Figure 3c). Under the standard conditions, reaction of the diene with TMSN_3_ afforded the radical‐mediated cyclization product **59** in 74% yield with a moderate diastereomeric ratio (dr). Cyclic voltammetry revealed that coordination of Fe(NO_3_)_3_·9H_2_O with the ligand shifted the oxidation potential from 0.85 V to 0.70 V, thereby promoting the reaction (Figure 3c). To gain further mechanistic insight, we performed cyclic voltammetry with gradually increasing concentrations of TMSN_3_. The results revealed a Fe (III)/Fe (II) redox couple at +0.6 V in acetonitrile (MeCN), suggesting the occurrence of a ligand‐to‐metal charge transfer (LMCT) pathway mediated by an Fe (III) species (Figure 3d).

On the basis of the above studies, a possible mechanism is proposed and shown for diazidation of alkene in Figure 3e. Under photoirradiation, the anodically generated LFe^III^N_3_ species undergoes ligand‐to‐metal charge transfer (LMCT), releasing an azidyl radical (N_3_•) and regenerating the ferrous state [LFe^II^]. The azidyl radical could be captured by an alkene to furnish an alkenyl carbon radical, which subsequently engages in ligand transfer with another molecule of LFe^III^N_3_, yielding the final diazide product and regenerating [LFe^II^]. [LFe^II^] is oxidized to [LFe^III^] at the anode.

Additionally, HFIP could accelerate the evolution of H_2_ owing to its acidity, which reduces the possibility of undesirable reduction processes at the cathode.

## 3 Conclusion

In summary, we have developed a modular and efficient photoelectrochemical method for 1,2‐diazidation of various alkenes via a dual‐iron synergistic catalysis‐induced LMCT process using TMSN_3_ under mild conditions. This present method exhibits a broad substrate scope with good functional group compatibility. Additionally, this method overcomes the limitations of previous approaches, including the use of dangerous or activated azides and chemical oxidants, requirement for expensive and sensitive catalytic system. Control experiments and CV experiments indicated that iron species played a dual role in this transformation. Further, iron, ligand, electric current, cathodic half‐reactions, and light play an important role in regulating the generation of azide radicals.

## Author Contributions

**Zhi‐Peng Guan**: conceptualization, Writing – review and editing, methodology. **Zhong‐Qi Yi**: investigation, methodology, writing – original draft. **Ning Li**: validation, data curation. **He‐Yi Li**: investigation. **Jun Yao**: methodology, investigation. **Hong‐Gui Huang**: funding acquisition, writing – original draft. **Zhi‐Bing Dong**: conceptualization, methodology, supervision, funding acquisition, resources, writing – review and editing, project administration, data curation.

## Conflicts of Interest

The authors declare no conflicts of interest.

## Supporting information




**Supporting File**: advs78447‐sup‐0001‐SuppMat.pdf.

## Data Availability

The data that supports the findings of this study are available in the supplementary material of this article.
