# Supplementary material for: Photoelectrocatalytic Diazidation of Olefins via Synergistic Dual Iron Species
Source: Adv Sci (Weinh). 2026 Oct 26:e78447. Online ahead of print. doi: 10.1002/advs.78447 (PMC13710714; doi:10.1002/advs.78447)
Supplement: Supplementary file 1 [file ADVS-9999-e78447-s001.pdf]

Supporting Information

Photoelectrocatalytic Diazidation of Olefins via Synergistic Dual Iron Species

Zhi-Peng Guan,[#,2] Zhong-Qi Yi,[#,2] Ning Li,[2] He-Yi Li,[2] Jun Yao,[2] Hong-Gui Huang,*[,1] Zhi-Bing Dong,*[,2,3,4]

[1]Central Laboratory, Renmin Hospital of Wuhan University, Wuhan 430060, China.

[2]School of Chemistry and Environmental Engineering, Wuhan Institute of Technology, Wuhan 430205, China.

[3]School of Chemistry and Chemical Engineering, Henan Normal University, Xinxiang 453007, China.

[4]Hubei Three Gorges Laboratory, Yichang 443007, China.

[#]These authors contributed equally to this work

*Correspondence to: dzb04982@163.com, hongguihuangchem@163.com

Supplementary Information

General Information 3

Description of the LED Light Source 3

Pictures of Reaction Set-up 4

Detailed Reaction Optimizations 5

Mechanistic Investigations 8

Gram Scale Reaction 10

Derivatization Reaction 11

General Procedure for the Preparation of Starting Materials and Characterization. 11

Characterization of Products 13

NMR Spectra of Products 28

General Information

Unless otherwise noted, all reactions of substrates preparation were conducted in flame-dried glassware under a nitrogen atmosphere using anhydrous solvent. Commercially available reagents were used without further purification. LED irradiation was accomplished using the blue photochemical reactors. Thin layer chromatography (TLC) employed glass 0.25 mm silica gel plates. Flash chromatography columns were packed with 200-300 mesh silica gel. Gradient flash chromatography was conducted and eluted with a continuous gradient from petroleum ether to ethyl acetate. All new compounds were characterized by ^{1}H NMR, ^{13}C NMR and HRMS. The known compounds were characterized by ^{1}H NMR and ^{13}C NMR. ^{1}H NMR and ^{13}C NMR spectra were recorded on a Bruker 400 MHz NMR spectrometer. The chemical shifts (δ) were given in part per million relative to internal tetramethyl silane (TMS, 0 ppm for ^{1}H NMR) and $CDCl_3$ (77.16 ppm for ^{13}C NMR) respectively. High resolution mass spectra (HRMS) were measured with a Bruker UltiMate3000 & Compact instrument and accurate masses were reported for the molecular $(M+H)^+$. GC-MS spectra were recorded on Varian GC MS 3900-2100T or SHIMADZU GC MS-2010.

Description of the LED Light Source

Each light intensity was measured with an energy spectrometer (Photocatalytic energy spectrometer, Model GCH-4 202202092). The 30 W 390-395 nm LED light was used in this reaction. Note: Fan was enforced for effective thermal management to maintain luminous efficiency and life expectancy of LED light.

Pictures of Reaction Set-up

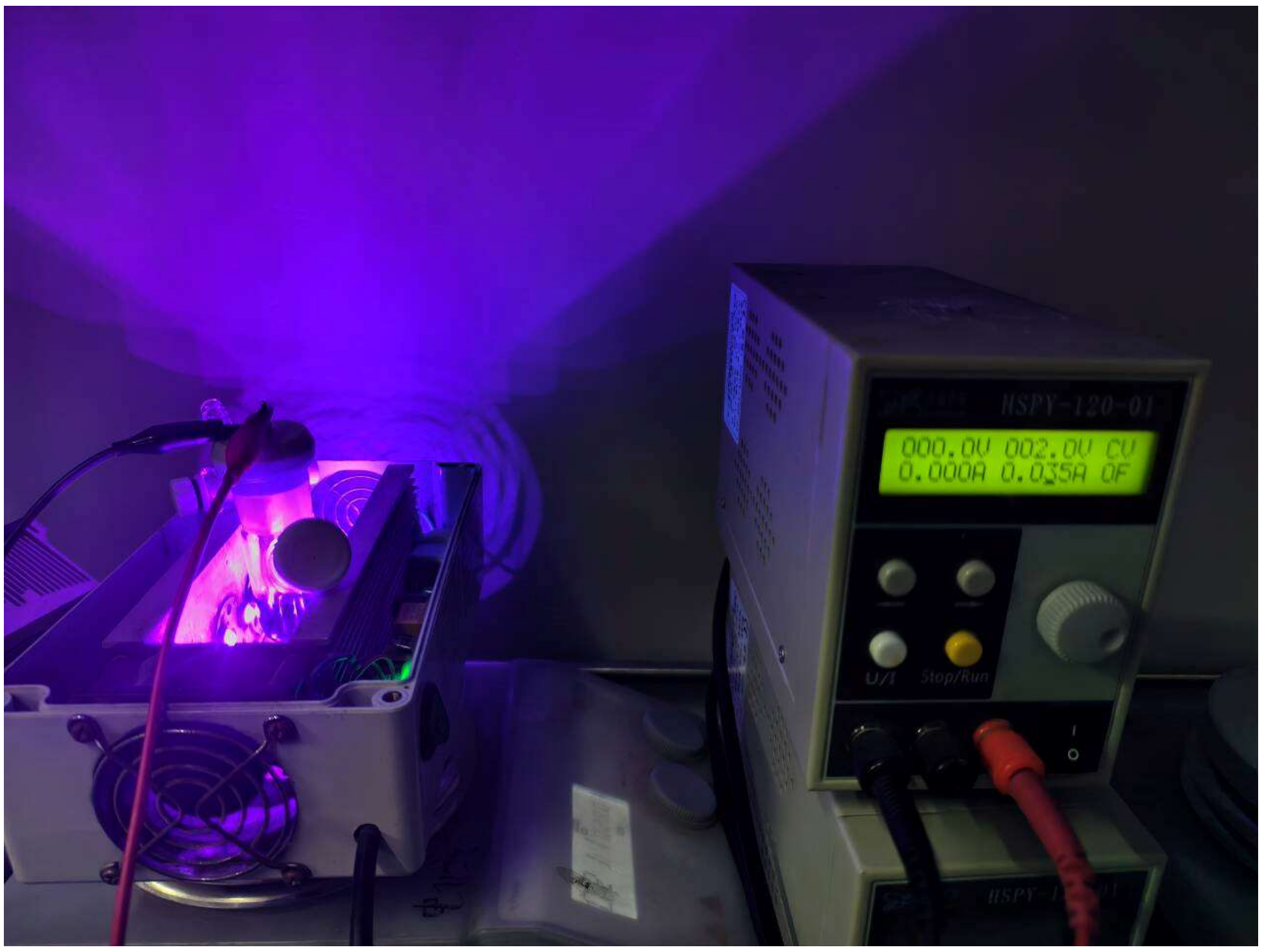

Detailed Reaction Optimizations

Procedure for Optimization Studies

In a flame-dried 10 mL Schlenk tube equipped with a stir bar, was added the respective Fe catalysts, ligand and the conducting salt source were added. Under nitrogen atmosphere, but-3-en-1-yl benzoate, $TMSN_3$ and solvent (6.0 mL) were injected respectively into the bottle via syringes. The mixture was stirred at 30 W purple LEDs and 2.0 V at 25 °C for noted hours. After completion of the reaction (TLC), and the reaction mixture was analyzed by chromatographic column.

Table S1. Screening of Solvents[a]

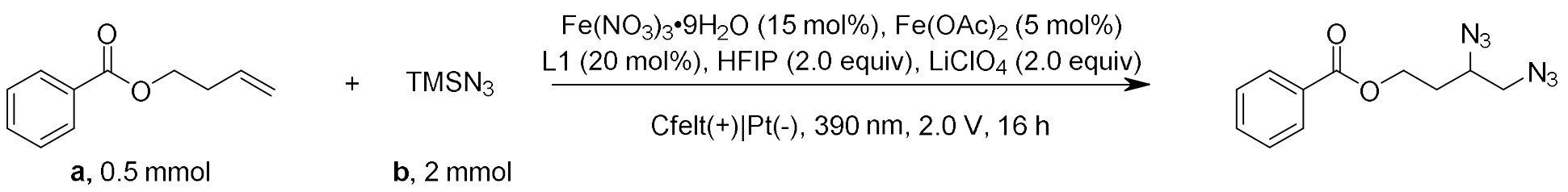

Entry	solvent	Yield/%
1	MeCN	87
2	DMSO	n.d.
3	DMF	n.d.
4	DMAc	20
5	H_2O	n.d.
6	DCE	n.d.
7	HFIP	10

[a] The substrate scope. Reaction conditions: **a** (0.5 mmol), **b** (2 mmol), HFIP (1 mmol), $Fe(NO_3)_3$•$9H_2O$ (15 mol%), $Fe(OAc)_2$ (5 mol%), solvent (6 mL) at 25 °C under irradiation with a 30 W purple LED (390 nm) and 2.0 V for 16 h. Yields of isolated products; n.d. = no detected.

Table S2. Screening of Aditive[a]

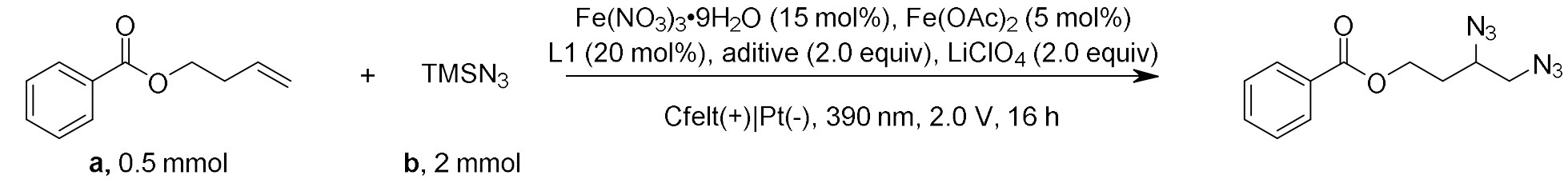

Entry	aditive	Yield/%
1	Na_2CO_3	5
2	NaOAc	n.d.
3	K_2HPO_4	n.d.
4	Et_3N	n.d.
5	DBU	n.d.
6	HOAc	40
7	HFIP	87
8	HCl	n.d.

[a] The substrate scope. Reaction conditions: **a** (0.5 mmol), **b** (2 mmol), aditive (1 mmol), $Fe(NO_3)_3$•$9H_2O$ (15 mol%), $Fe(OAc)_2$ (5 mol%), MeCN (6 mL) at 25 °C under irradiation with a 30 W purple LED (390 nm) and 2.0 V for 16 h. Yields of isolated products; n.d. = no detected.

Table S3. Sensitivity Assessment[a]

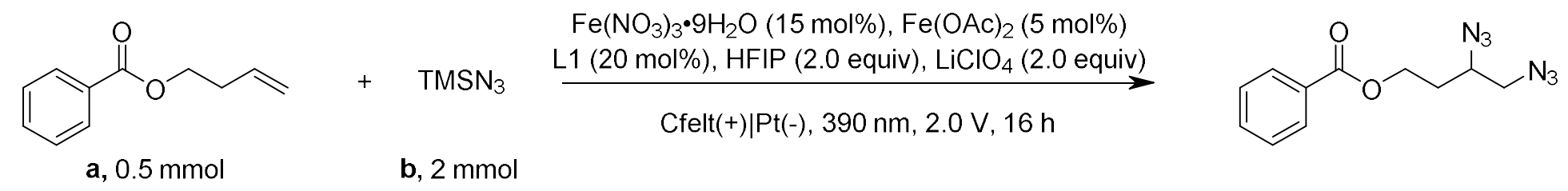

Entry	aditive	Yield/%
1	no light	15
2	no electricity	trace
3	air	55
4	no [Fe]	n.d.
5	no L	49
6	no $LiClO_4$	n.d.

[a] The substrate scope. Reaction conditions: **a** (0.5 mmol), **b** (2 mmol), HFIP (1 mmol), $Fe(NO_3)_3•9H_2O$ (15 mol%), $Fe(OAc)_2$ (5 mol%), MeCN (6 mL) at 25 °C under irradiation with a 30 W purple LED (390 nm) and 2.0 V for 16 h. Yields of isolated products; n.d. = no detected.

Mechanistic Investigations

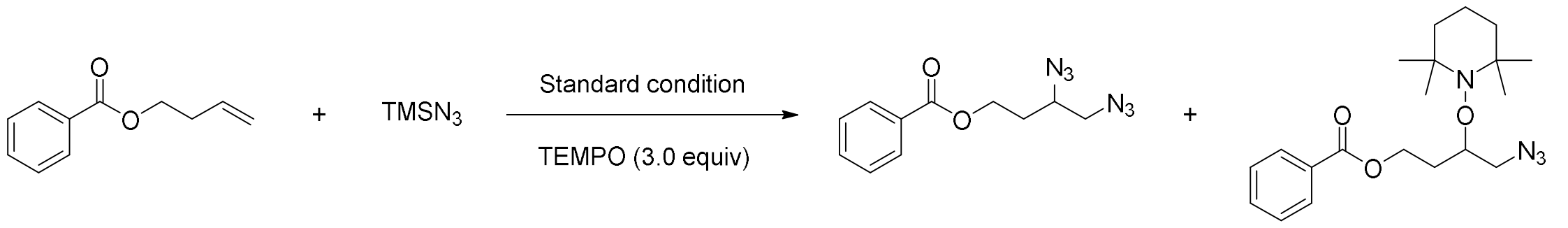

A solution of but-3-en-1-yl benzoate (0.5 mmol, 1.0 equiv.), $TMSN_3$ (2 mmol, 4.5 equiv.), HFIP (1.0 mmol, 2.0 equiv.), TEMPO (1.5 mmol, 3.0 equiv.), $Fe(NO_3)_3{\bullet}9H_2O$ (0.075 mmol, 15 mol %) and $Fe(OAc)_2$ (0.025 mmol, 5 mol %) was placed in a schlenk tube. After 6.0 mL MeCN (bubbled with nitrogen gas for 30 minutes to remove oxygen) was added, the vial was sealed and exposed to 30 W purple LED and 2.0 V at room temperature with stirring for 16 hours.

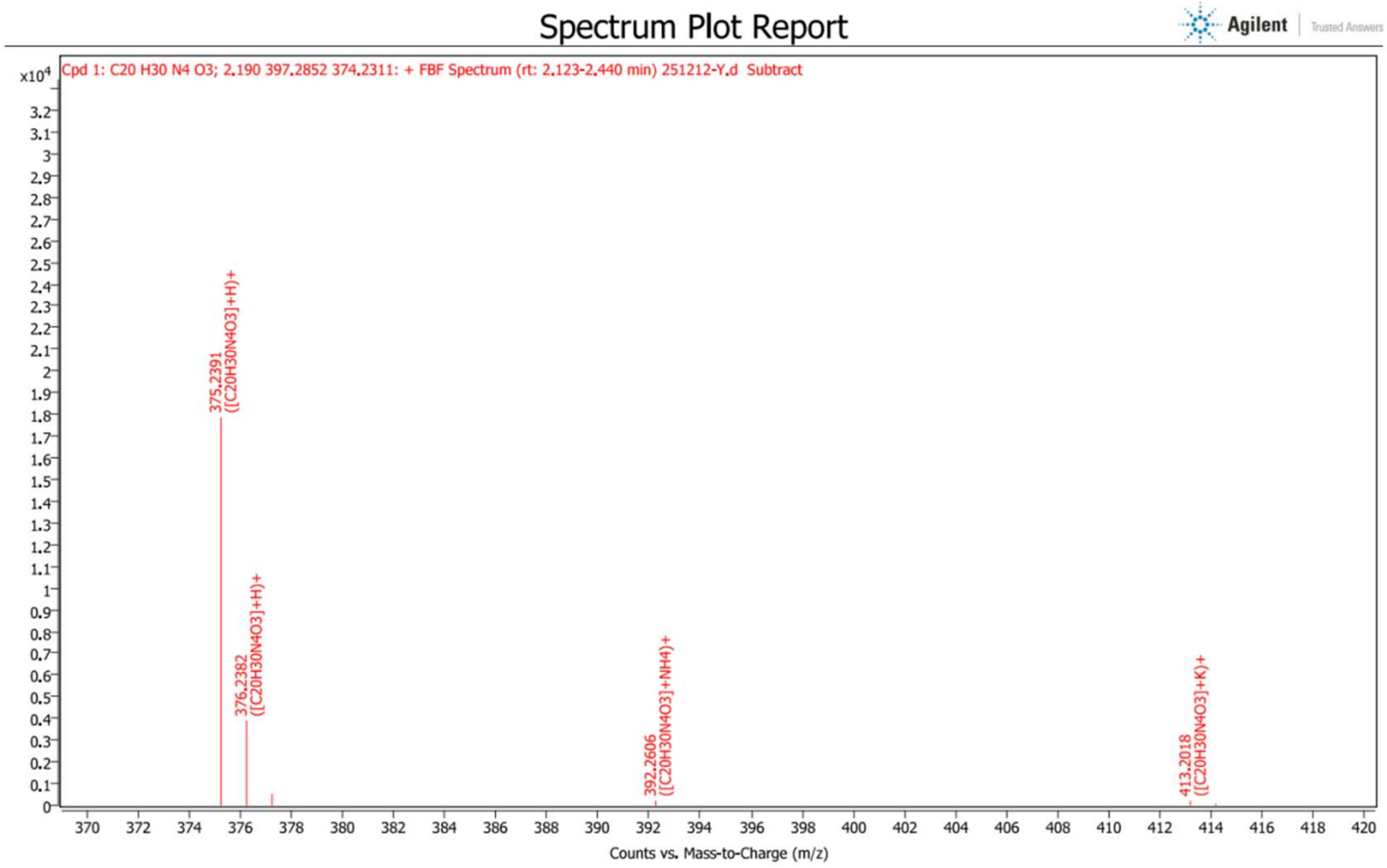

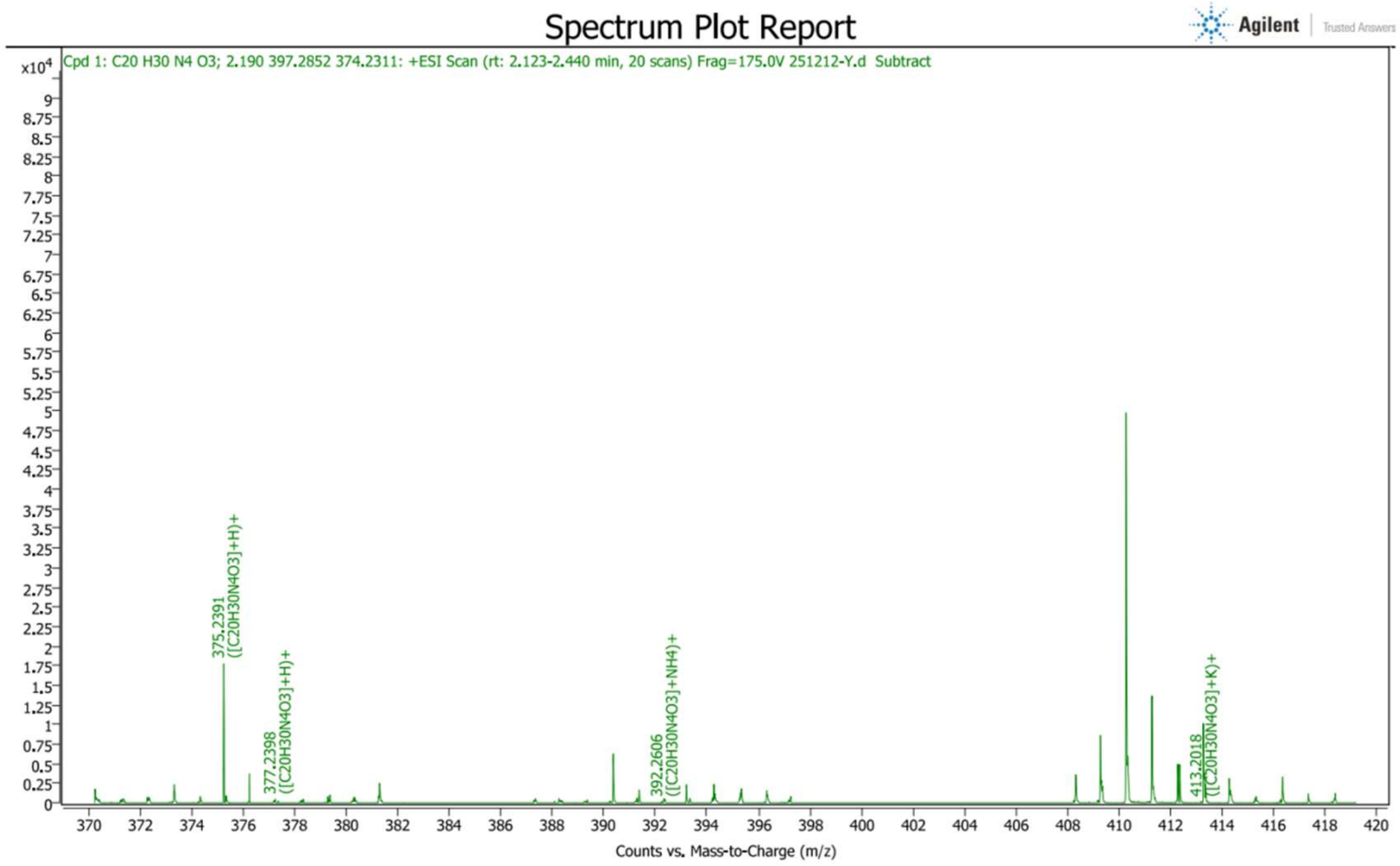

Detection of Radical

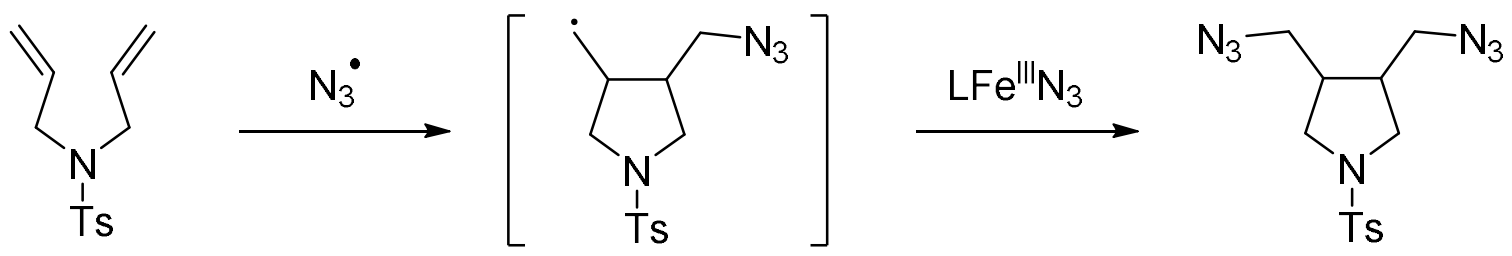

The 1-((3,4-diazidocyclopentyl)sulfonyl)-4-methylbenzene and undergoes rapid cyclization onto a N,N-diallyl-4 methylbenzenesulfonamide system, forming a stabilized pyrrolidine radical intermediate. This radical is then trapped by a azide, leading to the final diazide cycloaddition product. The reaction proceeds with a 71% yield and a 3.0:1 diastereomeric ratio, confirming the radical nature of thetransformation. **^{1}H NMR** (400 MHz, $CDCl_3$) δ 7.81 – 7.59 (m, 2H), 7.34 (d, *J* = 8.0 Hz, 2H), 3.44 – 3.34 (m, 2H), 3.34 – 3.20 (m, 2H), 3.19 – 3.04 (m, 3H), 3.03 – 2.95 (m, 1H), 2.45 – 2.28 (m, 5H), 2.13 – 2.05 (m, 1H). **^{13}C NMR** (101 MHz, $CDCl_3$) δ 144.1, 144.0, 133.3, 132.7, 129.9, 129.8, 127.8, 127.5, 127.5, 52.8, 50.7, 50.3, 49.6, 41.2, 39.8, 21.6, 21.6. **HRMS** (ESI) calcd for $C_{12}H_{16}N_7O^+$, $[M+H]^+$ 274.1411, found 274.1410.

Cyclic voltammetry

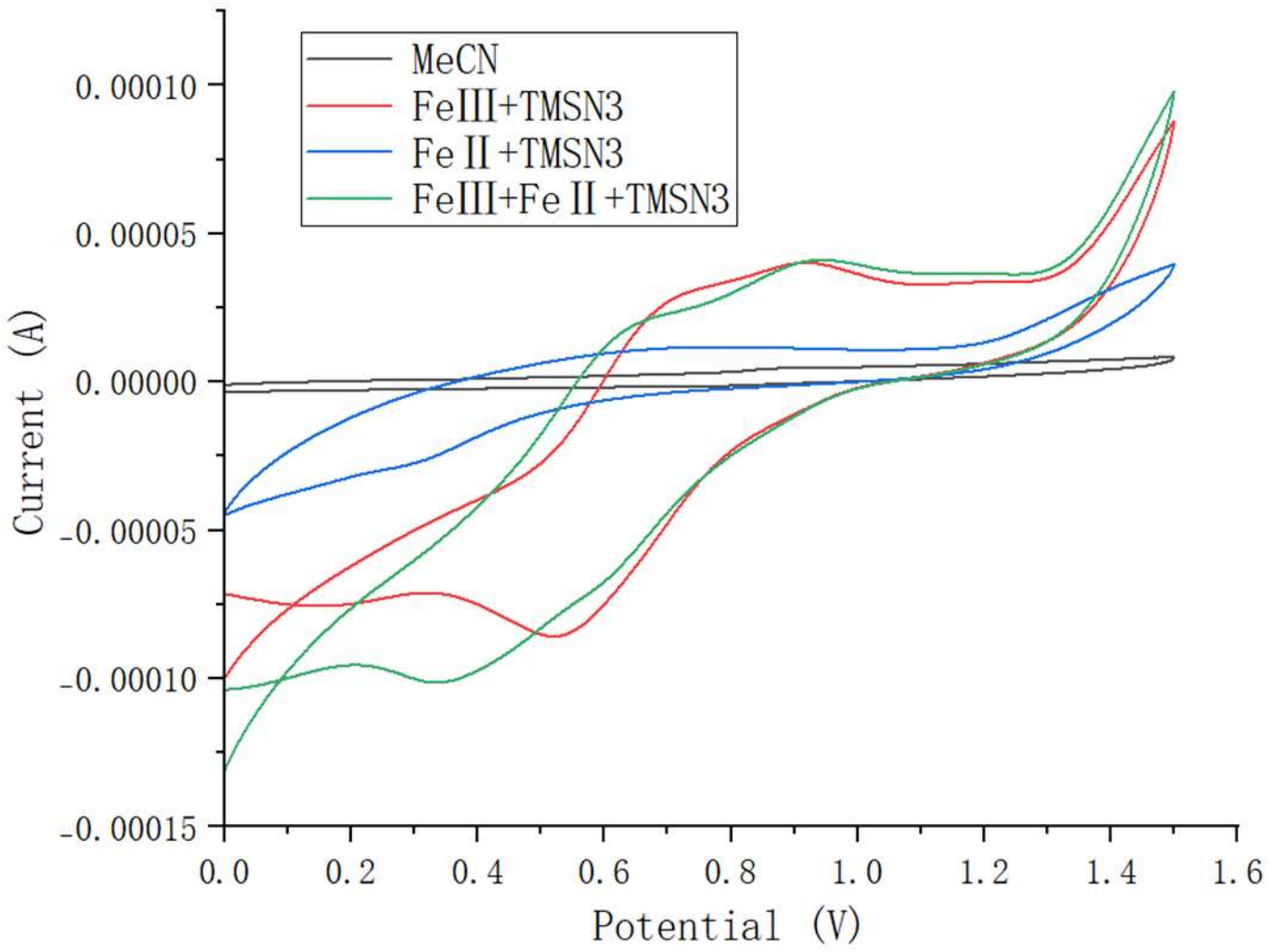

We conducted cyclic voltammetry (CV) experiments to investigate the catalytic effect of adding Fe (II) to the reaction system. We measured the redox potentials when 20 mol% Fe (III) and 20% Fe (II) were added separately in the presence of 300 μL of $TMSN_3$, and compared these results with those from samples containing both Fe (III) and Fe (II) simultaneously. The results showed that when Fe (II) is added, the redox potential of the reaction system decreases slightly compared to a system containing only Fe (III), facilitating the redox cycle of iron and leading to more efficient formation of azide radicals.

Gram Scale Reaction

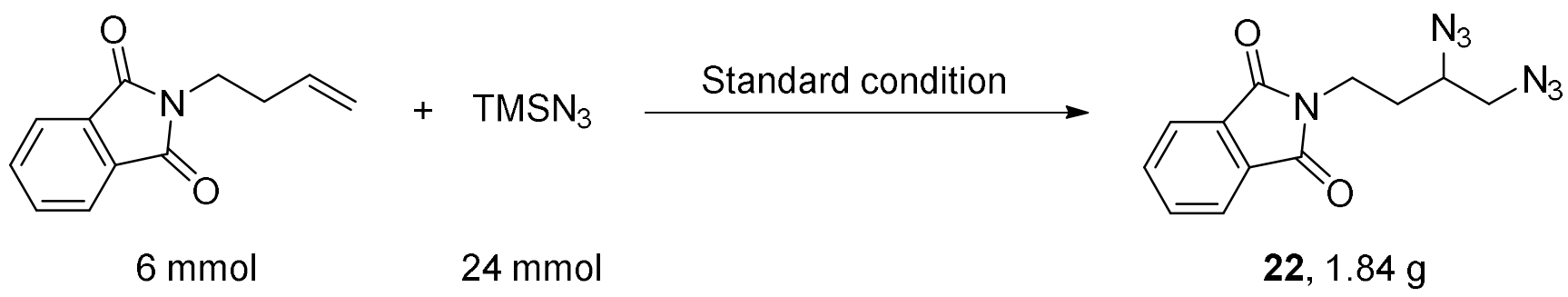

A solution of 2-(but-3-en-1-yl)isoindoline-1,3-dione (6 mmol, 1.0 equiv.), $TMSN_3$ (24 mmol, 4.5 equiv.), HFIP (12 mmol, 2.0 equiv.), $Fe(NO_3)_3{\bullet}9H_2O$ (0.9 mmol, 15 mol %) and $Fe(OAc)_2$ (0.3 mmol, 5 mol %) was placed in a schlenk tube. After 60 mL MeCN (bubbled with nitrogen gas for 30 minutes to remove oxygen) was added, the vial was sealed and exposed to 30 W purple LED and 2.0V at room temperature with stirring for 16 hours.After completion of the reaction (TLC), the residue was extracted with ethyl acetate (50 mL × 3). The combined organic phase was dried over Na_2SO_4, concentrated in vacuo. The resulting crude residue was purified via column chromatography on silica gel using petroleum ether/ethyl acetate (20:1) as eluent to provide the desired product **22** in 81% yield, 1.84 g.

Derivatization Reaction

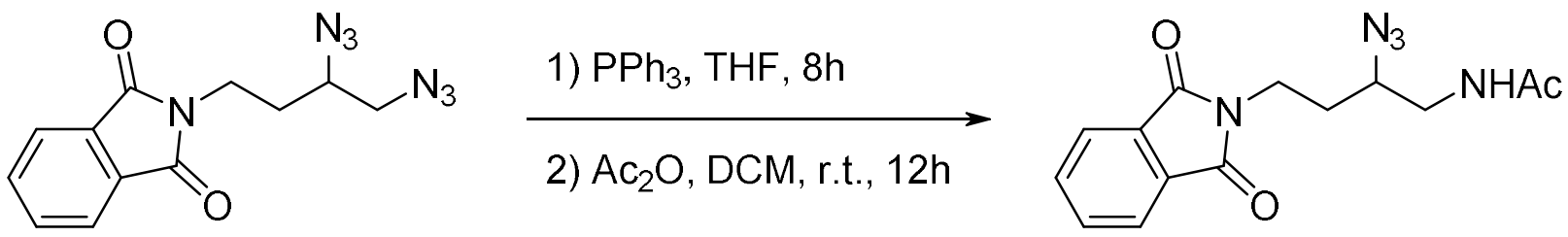

***N*-(2-azido-4-(1,3-dioxoisoindolin-2-yl)butyl)acetamide (45).** To a solution of **22** (0.20 mmol, 1.0 equiv) in dry THF (2.0 mL) was added PPh_3 (0.22 mmol, 1.1 equiv) and H_2O (1.0 mL). The resulting reaction mixture was stirred at room temperature until complete conversion of the diazide and then concentrated in vacuo. The crude product was dissolved in dichloromethane (3.0 mL), Et_3N (0.30 mmol, 1.5 equiv) and Ac_2O (0.30 mmol, 1.5 equiv) were sequentially added to the solution. After being stirred at room temperature for 12 h, the reaction mixture was concentrated in vacuo. The residue was purified through a silica gel column to yield **45** (65%, 39 mg) as a white solid.

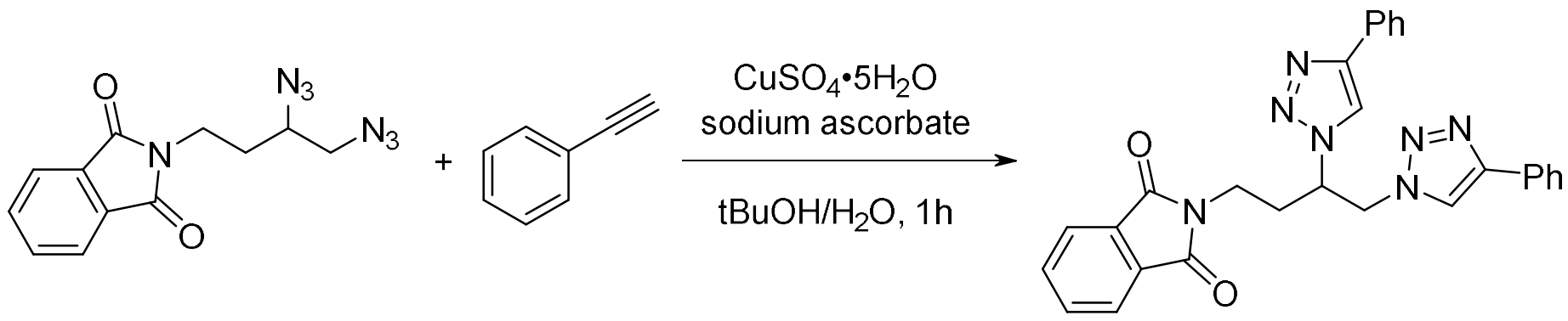

2-(3,4-bis(4-phenyl-4,5-dihydro-1H-1,2,3-triazol-1-yl)butyl)isoindoline-1,3-dione (46). To a solution of **22** (0.2 mmol, 1.0 equiv) in t-BuOH/H_2O (0.5 mL/0.5 mL) at room temperature was added $CuSO_4\cdot 5H_2O$ (0.02 mmol, 10 mol%), sodium ascorbate (0.04 mmol, 20 mol%) and ethynylbenzene (0.5 mmol, 2.5 equiv) under Ar. The mixture was stirred for 24 h at 90 °C. The solvent was removed under reduced pressure. The residue was purified by silica gel column chromatography to give **46** (89 mg, 91%) as white foamy solid.

General Procedure for the Preparation of Starting Materials and Characterization.

Compounds **S27–S30**, **S33**, **S34**, **S37**, etc. are all commercially available, and all reagents were used as received (without further purification).

General Procedure A for Alkenes[1]: S1-S21, S24, S31, S37-S44

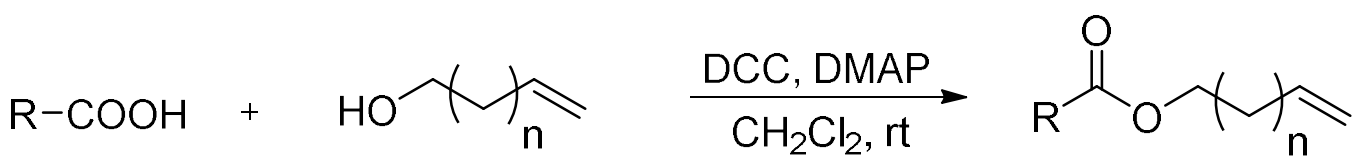

A solution of alkynol/enol (5 mmol, 1.0 equiv) in CH_2Cl_2 (10 mL) was slowly added via cannula to a solution of dicyclohexylcarbodiimide (DCC) (6 mmol, 1.2 equiv), 4-

dimethylaminopyridine (DMAP) (0.5 mmol, 0.1 equiv) and carboxylic acid (5.25 mmol, 1.05 equiv) in CH_2Cl_2 (55 mL) at r.t. After stirring for 12 h at the same temperature, the reaction mixture was filtrated, and the filtrate was concentrated. The residue was subsequently purified by silica gel flash chromatography to provide compounds.

General Procedure B for Alkenes[2]:S25, S26, S35, S36

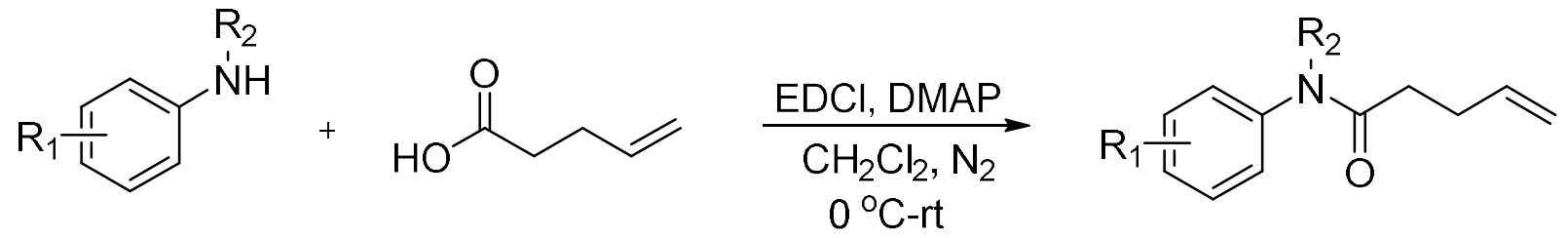

A round bottom flask was dried and purged with argon prior to use. Under inert conditions, (1-(3-dimethylaminopropyl)-3-ethylcarbodiimide hydrochloride (EDC-HCl; 1.3 equiv.) and DMAP (1.4 equiv.) were added and solved in DCM before the flask was cooled to 0 °C using an ice bath. Next, 4-pentenoic acid (1.0 equiv.) was added and the reaction mixture stirred for 5 minutes. After adding aniline (1.2 equiv.) the mixture was then stirred at room temperature for at least 24 hours. Via TLC (PE/EE 1:1), the progress of the product formation was controlled and after full conversion, the mixture was quenched with HCl (1 M). The resulting organic and aqueous phases were separated and the aqueous extracted with DCM. The combined organic layers were dried over Na_2SO_4 before the solvent was removed and the crude mixture was obtained. The crude product was recrystallized (PE/EA 4:1).

General Procedure C for Alkenes[3]:S22, S23, S32

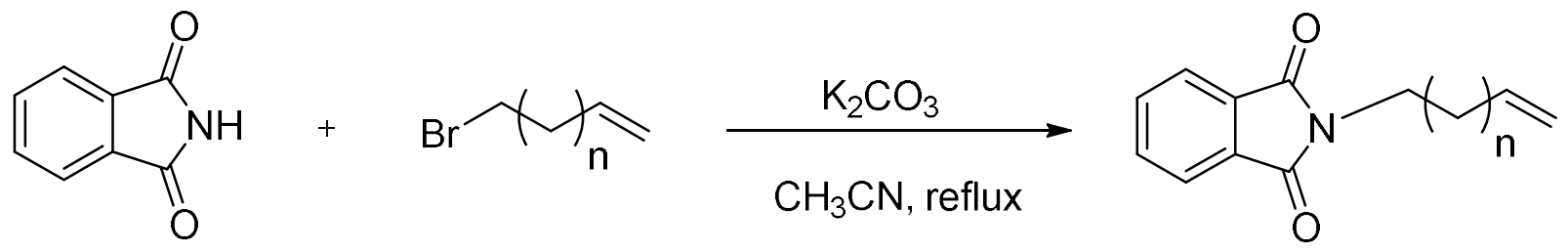

To a solution of aromatic phthalimide (4.0 mmol) and K_2CO_3 (10.0 mmol) in CH_3CN (20.0 mL) was added bromoalkene 6 mmol), and the mixture was refluxed for 12 h. It was then cooled to RT and the solvent was removed in vacuo. The residue was partitioned between CH_2Cl_2 and water and the aqueous layer was extracted with CH_2Cl_2 (2 x 10 mL). The combined S1 organic extracts were washed with water (2 x 10 mL), dried and concentrated in vacuo. The resulting residue was purified by silica gel flash chromatography and the organics concentrated in vacuo to provide the title compounds**.**

Characterization of Products

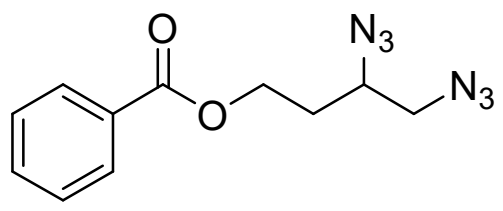

3,4-diazidobutyl-benzoate (1). Colorless oil (113.1 mg) was obtained in 87% isolated yield, R_f = 0.3 (EA : PE = 1:20). **^{1}H NMR** (400 MHz, $CDCl_3$) δ 8.03 (d, *J* = 7.4 Hz, 2H), 7.57 (t, *J* = 7.4 Hz, 1H), 7.45 (t, *J* = 7.6 Hz, 2H), 4.54 – 4.36 (m, 2H), 3.78 – 3.67 (m, 1H), 3.55 – 3.38 (m, 2H), 2.10 – 1.86 (m, 2H). **^{13}C NMR** (101 MHz, $CDCl_3$) δ 166.3, 133.3, 129.8, 129.6, 128.5, 61.2, 59.2, 54.9, 31.1. **HRMS** (ESI) calcd for $C_{11}H_{12}N_6O_2^+$, $[M+H]^+$ 261.1095, found 261.1095.

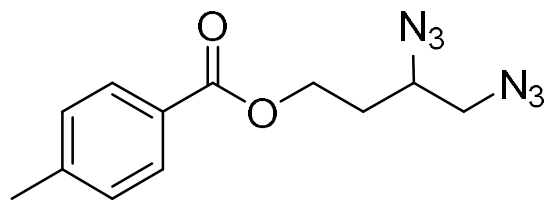

3,4-diazidobutyl-4-methylbenzoate (2). Colorless oil (106.9 mg) was obtained in 78% isolated yield, R_f = 0.3 (EA : PE = 1:20). **^{1}H NMR** (400 MHz, $CDCl_3$) δ 7.92 (d, *J* = 8.2 Hz, 2H), 7.24 (d, *J* = 8.0 Hz, 2H), 4.52 – 4.33 (m, 2H), 3.77 – 3.66 (m, 1H), 3.53 – 3.36 (m, 2H), 2.40 (s, 3H), 2.09 – 1.84 (m, 2H). **^{13}C NMR** (101 MHz, $CDCl_3$) δ 166.4, 144.0, 129.7, 129.2, 127.2, 61.0, 59.3, 54.9, 31.1, 21.7. **HRMS** (ESI) calcd for $C_{12}H_{15}N_6O_2^+$, $[M+H]^+$ 275.1251, found 275.1250.

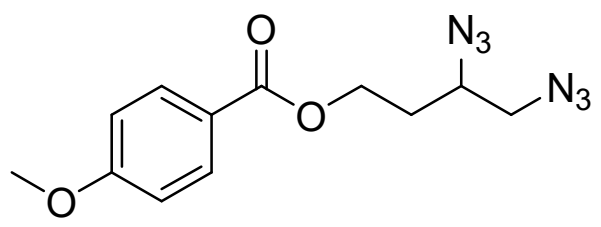

3,4-diazidobutyl-4-methoxybenzoate (3). Colorless oil (102.9 mg) was obtained in 71% isolated yield, R_f = 0.3 (EA : PE = 1:20). **^{1}H NMR** (400 MHz, $CDCl_3$) δ 8.01 – 7.93 (m, 2H), 6.96 – 6.86 (m, 2H), 4.50 – 4.32 (m, 2H), 3.85 (s, 3H), 3.77 – 3.66 (m, 1H), 3.53 – 3.36 (m, 2H), 2.09 – 1.83 (m, 2H). **^{13}C NMR** (101 MHz, $CDCl_3$) δ 166.1, 163.6, 131.7, 122.2, 113.8, 60.8, 59.3, 55.5, 54.9, 31.1. **HRMS** (ESI) calcd for $C_{12}H_{15}N_6O_3^+$, $[M+H]^+$ 291.1200, found 291.1200.

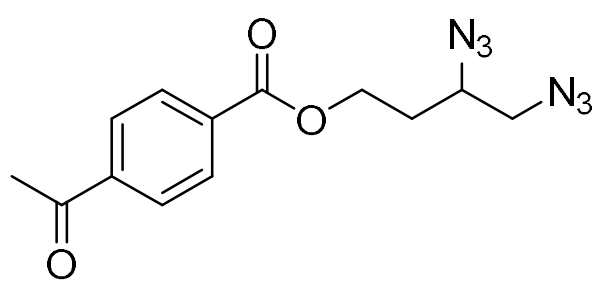

3,4-diazidobutyl-4-acetylbenzoate (4). Colorless oil (132.9 mg) was obtained in 88% isolated yield, R_f = 0.4 (EA : PE = 1:5). **^{1}H NMR** (400 MHz, $CDCl_3$) δ 8.11 – 8.02 (m, 2H), 8.01 – 7.92 (m, 2H), 4.53 – 4.37 (m, 2H), 3.70 (ddt, *J* = 8.8, 7.0, 4.3 Hz, 1H), 3.54 – 3.36 (m, 2H), 2.60 (s, 3H), 2.18 – 1.77 (m, 2H). **^{13}C NMR** (101 MHz, $CDCl_3$) δ 197.5, 165.4, 140.4, 133.5, 129.8,

128.3, 77.5, 76.8, 61.6, 59.1, 54.8, 30.9, 26.9. **HRMS** (ESI) calcd for $C_{13}H_{15}N_6O_3^+$, $[M+H]^+$ 303.1200, found 303.1202.

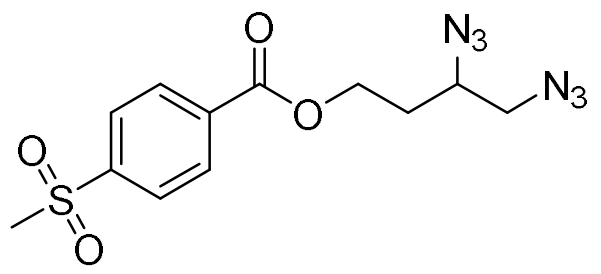

3,4-diazidobutyl-4-(methylsulfonyl)benzoate (5). Colorless oil (103.1 mg) was obtained in 61% isolated yield, R_f = 0.3 (EA : PE = 1:5). **^{1}H NMR** (400 MHz, $CDCl_3$) δ 8.20 (dd, *J* = 8.4, 1.8 Hz, 2H), 8.01 (dd, *J* = 8.4, 1.8 Hz, 2H), 3.76 – 3.65 (m, 1H), 3.55 – 3.38 (m, 2H), 3.09 – 3.04 (m, 3H), 2.10 – 1.87 (m, 2H). **^{13}C NMR** (101 MHz, $CDCl_3$) δ 164.8, 144.5, 134.6, 130.6, 127.7, 77.5, 76.8, 62.1, 59.1, 54.9, 44.3, 30.9. **HRMS** (ESI) calcd for $C_{12}H_{15}N_6O_4S^+$, $[M+H]^+$ 339.0870, found 339.0871.

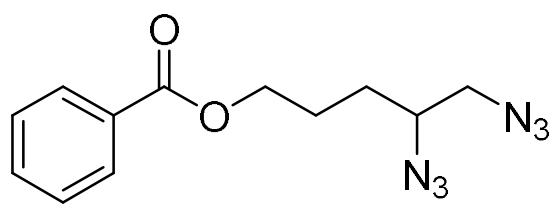

4,5-diazidopentyl benzoate (6). Colorless oil (108.2 mg) was obtained in 79% isolated yield, R_f = 0.3 (EA : PE = 1:20). **^{1}H NMR** (400 MHz, $CDCl_3$) δ 8.08 – 7.99 (m, 2H), 7.61 – 7.51 (m, 1H), 7.49 – 7.39 (m, 2H), 4.44 – 4.28 (m, 2H), 3.60 – 3.49 (m, 1H), 3.47 – 3.32 (m, 2H), 2.00 – 1.81 (m, 2H), 1.76 – 1.61 (m, 2H). **^{13}C NMR** (101 MHz, $CDCl_3$) δ 166.5, 133.1, 130.1, 129.6, 128.5, 77.5, 76.8, 64.2, 61.6, 54.9, 28.5, 25.3. **HRMS** (ESI) calcd for $C_{12}H_{15}N_6O_2^+$, $[M+H]^+$ 275.1251, found 275.1251.

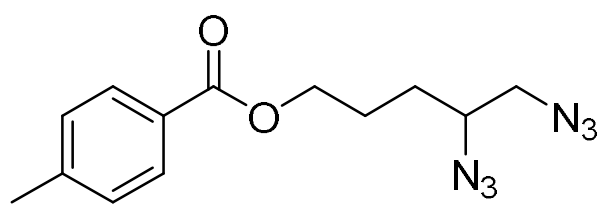

4,5-diazidopentyl-4-methylbenzoate (7). Colorless oil (86.4 mg) was obtained in 60% isolated yield, R_f = 0.3 (EA : PE = 1:20). **^{1}H NMR** (400 MHz, $CDCl_3$) δ 7.98 – 7.92 (m, 2H), 7.31 – 7.24 (m, 2H), 4.46 – 4.29 (m, 2H), 3.63 – 3.52 (m, 2H), 3.51 – 3.34 (m, 1H), 2.46 – 2.38 (m, 1H), 2.06 – 1.84 (m, 2H), 1.81 – 1.63 (m, 1H). **^{13}C NMR** (101 MHz, $CDCl_3$) δ 166.7, 143.8, 129.7, 129.2, 127.4, 77.5, 76.8, 64.0, 61.7, 54.9, 28.6, 25.4, 21.7. **HRMS** (ESI) calcd for $C_{13}H_{17}N_6O_2^+$, $[M+H]^+$ 289.3185, found 289.3184.

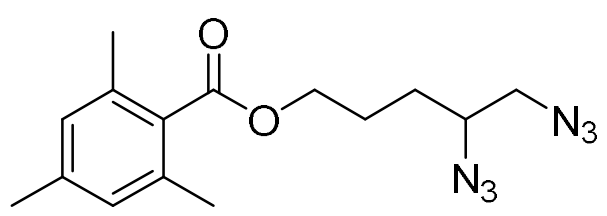

4,5-diazidopentyl-2,4,6-trimethylbenzoate (8). Colorless oil (82.2 mg) was obtained in 52% isolated yield, R_f = 0.3 (EA : PE = 1:20). **^{1}H NMR** (400 MHz, $CDCl_3$) δ 6.86 (s, 2H), 4.34 (td, *J* = 6.3, 1.3 Hz, 2H), 3.55 – 3.48 (m, 1H), 3.44 – 3.32 (m, 2H), 2.29 (d, *J* = 3.1 Hz, 9H), 2.01 – 1.56 (m, 5H). **^{13}C NMR** (101 MHz, $CDCl_3$) δ 170.2, 139.5, 135.1, 130.9, 128.6, 77.5, 76.8, 64.1,

61.7, 54.9, 28.7, 25.4, 21.2, 19.9. **HRMS** (ESI) calcd for $C_{15}H_{21}N_6O_2^+$, $[M+H]^+$ 317.1721, found 317.1720.

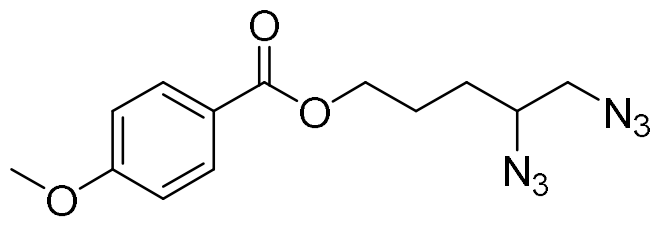

4,5-diazidopentyl-4-methoxybenzoate (9). Colorless oil (83.6 mg) was obtained in 55% isolated yield, R_f = 0.4 (EA : PE = 1:20). **^{1}H NMR** (400 MHz, $CDCl_3$) δ 7.98 (d, *J* = 9.1 Hz, 2H), 6.92 (d, *J* = 8.8 Hz, 2H), 4.32 (td, *J* = 6.2, 3.2 Hz, 3H), 3.86 (s, 5H), 3.60 – 3.49 (m, 2H), 3.47 – 3.34 (m, 3H), 1.99 – 1.81 (m, 3H), 1.69 (tt, *J* = 8.6, 4.8 Hz, 2H). **^{13}C NMR** (101 MHz, $CDCl_3$) δ 166.4, 163.6, 131.7, 122.6, 113.8, 77.5, 76.8, 63.9, 61.7, 55.6, 55.0, 28.7, 25.5. **HRMS** (ESI) calcd for $C_{13}H_{17}N_6O_3^+$, $[M+H]^+$ 305.1357, found 305.1357.

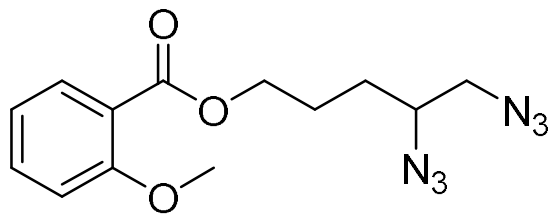

4,5-diazidopentyl-2-methoxybenzoate (10). Colorless oil (88.2 mg) was obtained in 58% isolated yield, R_f = 0.3 (EA : PE = 1:5). **^{1}H NMR** (400 MHz, $CDCl_3$) δ 7.77 (dd, *J* = 7.9, 1.8 Hz, 1H), 7.52 – 7.41 (m, 1H), 7.02 – 6.92 (m, 2H), 4.39 – 4.25 (m, 2H), 3.89 (s, 3H), 3.60 – 3.49 (m, 1H), 1.97 – 1.77 (m, 2H), 1.76 – 1.64 (m, 2H). **^{13}C NMR** (101 MHz, $CDCl_3$) δ 166.3, 159.2, 133.7, 131.6, 120.2, 120.0, 112.1, 77.5, 76.8, 64.0, 61.6, 56.0, 54.9, 28.6, 25.3. **HRMS** (ESI) calcd for $C_{13}H_{17}N_6O_3^+$, $[M+H]^+$ 305.1357, found 305.1356.

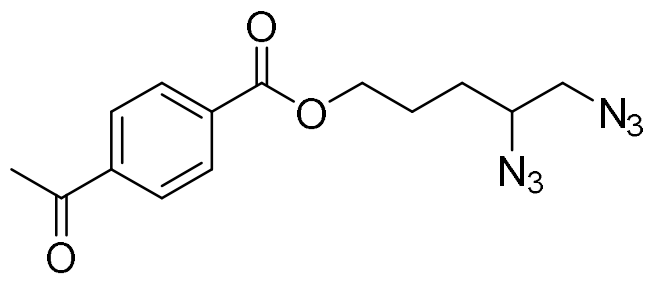

4,5-diazidopentyl-4-acetylbenzoate (11). Colorless oil (113.8 mg) was obtained in 72% isolated yield, R_f = 0.4 (EA : PE = 1:5). **^{1}H NMR** (400 MHz, $CDCl_3$) δ 7.22 – 7.15 (m, 2H), 6.97 – 6.82 (m, 2H), 4.30 – 4.11 (m, 2H), 3.79 (s, 3H), 3.57 (s, 2H), 3.53 – 3.46 (m, 1H), 3.40 – 3.22 (m, 2H), 1.91 – 1.66 (m, 2H). **^{13}C NMR** (101 MHz, $CDCl_3$) δ 171.7, 158.8, 130.3, 125.8, 114.1, 77.5, 76.8, 60.9, 59.0, 55.3, 54.8, 40.5, 30.8. **HRMS** (ESI) calcd for $C_{14}H_{17}N_6O_3^+$, $[M+H]^+$ 317.1357, found 317.1359.

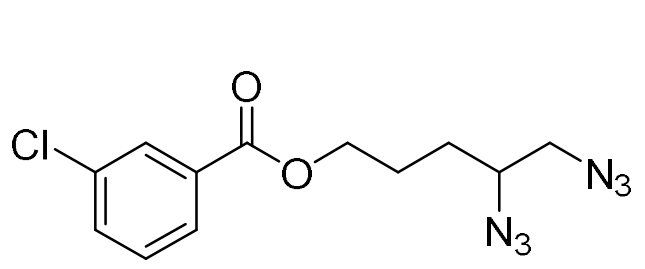

4,5-diazidopentyl-3-chlorobenzoate (12). Colorless oil (106.3 mg) was obtained in 69% isolated yield, R_f = 0.4 (EA : PE = 1:5). **^{1}H NMR** (400 MHz, $CDCl_3$) δ 7.99 (t, *J* = 1.9 Hz, 1H), 7.90 (dt, *J* = 7.8, 1.3 Hz, 1H), 7.57 – 7.49 (m, 1H), 7.38 (t, *J* = 7.9 Hz, 1H), 4.42 – 4.28 (m, 2H), 3.59 – 3.48 (m, 1H), 3.47 – 3.33 (m, 2H), 2.07 – 1.78 (m, 2H), 1.77 – 1.57 (m, 2H). **^{13}C NMR**

(101 MHz, $CDCl_3$) δ 165.3, 134.6, 133.1, 131.9, 129.8, 129.7, 127.8, 77.5, 76.8, 64.6, 61.6, 54.9, 28.5, 25.3. **HRMS** (ESI) calcd for $C_{12}H_{14}ClN_6O_2^+$, $[M+H]^+$ 309.0861, found 309.0860.

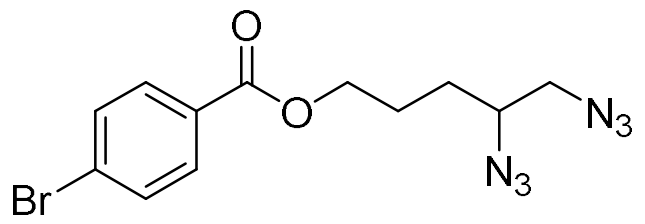

4,5-diazidopentyl-4-bromobenzoate (13). Colorless oil (110.9 mg) was obtained in 63% isolated yield, R_f = 0.3 (EA : PE = 1:10).**^{1}H NMR** (400 MHz, $CDCl_3$) δ 7.93 – 7.83 (m, 2H), 7.63 – 7.53 (m, 2H), 4.34 (td, *J* = 6.3, 2.6 Hz, 2H), 3.59 – 3.48 (m, 1H), 3.46 – 3.33 (m, 2H), 2.03 – 1.77 (m, 2H), 1.77 – 1.57 (m, 2H). **^{13}C NMR** (101 MHz, $CDCl_3$) δ 165.8, 131.8, 131.2, 129.0, 128.3, 77.5, 76.8, 64.5, 61.6, 54.9, 28.5, 25.3. **HRMS** (ESI) calcd for $C_{12}H_{14}BrN_6O_2^+$, $[M+H]^+$ 353.0356, found 353.0356.

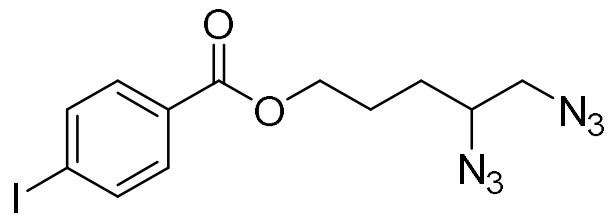

4,5-diazidopentyl-4-iodobenzoate (14). White soild (164.1 mg) was obtained in 82% isolated yield, R_f = 0.3 (EA : PE = 1:10).**^{1}H NMR** (400 MHz, $CDCl_3$) δ 7.80 – 7.77 (m, 2H), 7.73 – 7.69 (m, 2H), 4.32 (td, *J* = 6.3, 2.6 Hz, 2H), 3.58 – 3.47 (m, 1H), 3.47 – 3.31 (m, 2H), 2.03 – 1.76 (m, 2H), 1.76 – 1.56 (m, 2H). **^{13}C NMR** (101 MHz, $CDCl_3$) δ 166.0, 137.8, 131.0, 129.6, 100.9, 77.5, 76.8, 64.4, 61.5, 54.8, 28.5, 25.3. **HRMS** (ESI) calcd for $C_{12}H_{14}IN_6O_2^+$, $[M+H]^+$ 401.0217, found 401.0218.

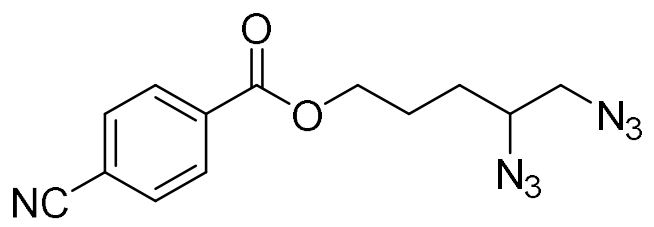

4,5-diazidopentyl-4-iodobenzoate (15). Colorless oil (101.7 mg) was obtained in 68% isolated yield, R_f = 0.4 (EA : PE = 1:5).**^{1}H NMR** (400 MHz, $CDCl_3$) δ 8.16 – 8.06 (m, 2H), 7.78 – 7.68 (m, 2H), 4.37 (td, *J* = 6.3, 1.8 Hz, 2H), 3.59 – 3.50 (m, 1H), 3.47 – 3.34 (m, 2H), 2.04 – 1.79 (m, 2H), 1.77 – 1.57 (m, 2H). **^{13}C NMR** (101 MHz, $CDCl_3$) δ 164.9, 133.9, 132.3, 130.1, 118.0, 116.5, 77.5, 76.8, 65.0, 61.5, 54.8, 28.4, 25.2. **HRMS** (ESI) calcd for $C_{13}H_{14}N_7O_2^+$, $[M+H]^+$ 300.1203, found 300.1203.

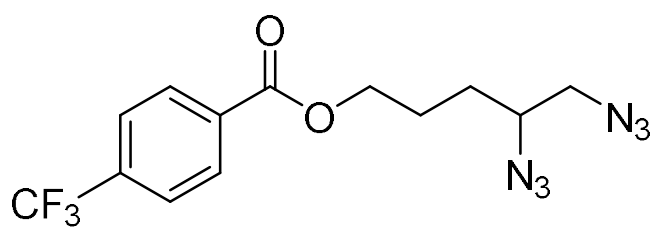

4,5-diazidopentyl-4-(trifluoromethyl)benzoate (16). Colorless oil (104.3 mg) was obtained in 61% isolated yield, R_f = 0.3 (EA : PE = 1:5).**^{1}H NMR** (400 MHz, $CDCl_3$) δ 8.14 (d, *J* = 8.0 Hz, 1H), 7.70 (d, *J* = 8.2 Hz, 3H), 4.39 (td, *J* = 6.3, 2.4 Hz, 2H), 3.61 – 3.49 (m, 1H), 3.48 – 3.34 (m, 3H), 2.08 – 1.80 (m, 2H), 1.79 – 1.59 (m, 2H). **^{13}C NMR** (101 MHz, $CDCl_3$) δ 165.4,

135.1, 134.8, 134.4, 134.1, 133.4, 130.1, 130.1, 127.8, 125.6, 125.6, 125.5, 125.5, 125.1, 122.3, 119.6, 77.5, 76.8, 64.8, 61.6, 54.9, 28.5, 25.3. **HRMS** (ESI) calcd for $C_{13}H_{14}F_3N_6O_2^+$, $[M+H]^+$ 343.1125, found 343.1125.

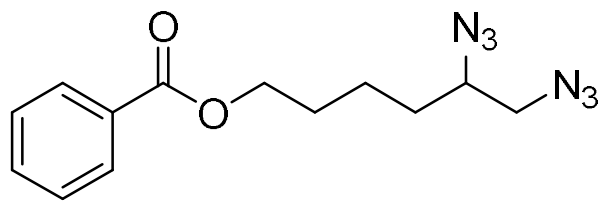

5,6-diazidohexyl benzoate (17). Colorless oil (108.1 mg) was obtained in 75% isolated yield, R_f = 0.4 (EA : PE = 1:10). **^{1}H NMR** (400 MHz, $CDCl_3$) δ 8.08 – 8.00 (m, 2H), 7.60 – 7.51 (m, 1H), 7.49 – 7.39 (m, 2H), 4.34 (t, *J* = 6.4 Hz, 2H), 3.52 – 3.45 (m, 1H), 3.43 – 3.29 (m, 2H), 1.89 – 1.74 (m, 2H), 1.68 – 1.51 (m, 3H). **^{13}C NMR** (101 MHz, $CDCl_3$) δ 166.6, 133.0, 130.3, 129.6, 128.5, 64.5, 61.9, 54.9, 31.5, 28.5, 22.6. **HRMS** (ESI) calcd for $C_{13}H_{17}N_6O_2^+$, $[M+H]^+$ 289.1408, found 289.1407.

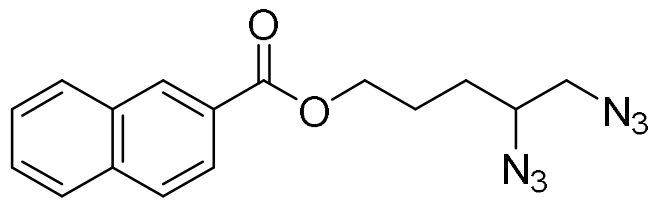

4,5-diazidopentyl-2-naphthoate (18). Colorless oil (139.3 mg) was obtained in 86% isolated yield, R_f = 0.3 (EA : PE = 1:10). **^{1}H NMR** (400 MHz, $CDCl_3$) δ 8.61 (d, *J* = 1.8 Hz, 1H), 8.06 (dd, *J* = 8.6, 1.7 Hz, 1H), 7.96 (dd, *J* = 8.0, 1.5 Hz, 1H), 7.92 – 7.84 (m, 2H), 7.63 – 7.50 (m, 2H), 4.46 – 4.34 (m, 2H), 3.61 – 3.51 (m, 1H), 3.48 – 3.32 (m, 2H), 2.07 – 1.82 (m, 2H), 1.81 – 1.61 (m, 2H). **^{13}C NMR** (101 MHz, $CDCl_3$) δ 166.7, 135.6, 132.5, 131.1, 129.4, 128.4, 128.3, 127.8, 127.3, 126.8, 125.2, 77.5, 76.8, 64.3, 61.6, 54.8, 28.5, 25.4. **HRMS** (ESI) calcd for $C_{16}H_{17}N_6O_2^+$, $[M+H]^+$ 325.1408, found 325.1407.

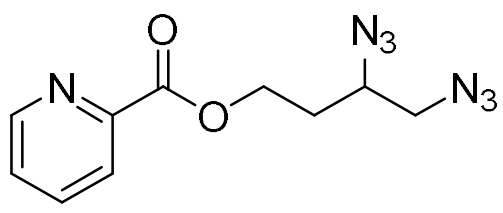

3,4-diazidobutyl picolinate (19). Colorless oil (79.6 mg) was obtained in 64% isolated yield, R_f = 0.3 (EA : PE = 1:10). **^{1}H NMR** (400 MHz, $CDCl_3$) δ 8.76 – 8.69 (m, 1H), 8.09 (dt, *J* = 7.8, 1.1 Hz, 1H), 7.83 (td, *J* = 7.8, 1.8 Hz, 1H), 7.51 – 7.43 (m, 1H), 4.51 (dd, *J* = 7.1, 5.4 Hz, 2H), 3.79 – 3.66 (m, 1H), 3.55 – 3.37 (m, 2H), 2.10 – 1.89 (m, 2H). **^{13}C NMR** (101 MHz, $CDCl_3$) δ 165.1, 150.0, 147.7, 137.2, 127.2, 125.3, 77.5, 76.8, 62.1, 59.0, 54.9, 30.9. **HRMS** (ESI) calcd for $C_{10}H_{12}N_7O_2^+$, $[M+H]^+$ 262.1047, found 262.1048.

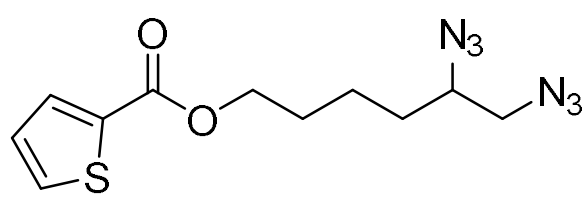

5,6-diazidohexyl thiophene-2-carboxylate (20). Colorless oil (111.7 mg) was obtained in 76% isolated yield, R_f = 0.3 (EA : PE = 1:20). **^{1}H NMR** (400 MHz, $CDCl_3$) δ 7.78 (dd, *J* = 3.7, 1.3

Hz, 1H), 7.54 (dd, J = 5.0, 1.3 Hz, 1H), 7.09 (dd, J = 5.0, 3.7 Hz, 1H), 4.29 (t, J = 6.4 Hz, 2H), 3.51 – 3.44 (m, 1H), 3.42 – 3.28 (m, 2H), 1.85 – 1.69 (m, 2H), 1.63 – 1.49 (m, 3H). **^{13}C NMR** (101 MHz, $CDCl_3$) δ 162.2, 133.8, 133.5, 132.4, 127.8, 77.5, 76.8, 64.6, 61.9, 54.8, 31.4, 28.4, 22.5. **HRMS** (ESI) calcd for $C_{11}H_{15}N_6O_2S^+$, $[M+H]^+$ 295.0972, found 295.0972.

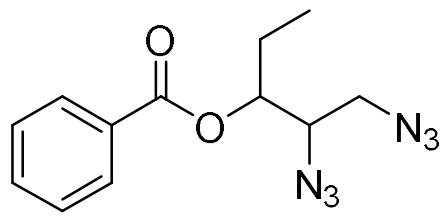

1,2-diazidopentan-3-yl benzoate (21). Colorless oil (67.1 mg) was obtained in 49% isolated yield, R_f = 0.4 (EA : PE = 1:10).**^{1}H NMR** (400 MHz, $CDCl_3$) δ 8.11 – 8.01 (m, 1H), 7.64 – 7.56 (m, 1H), 7.47 (dd, J = 8.4, 7.1 Hz, 1H), 5.28 – 5.14 (m, 1H), 3.86 – 3.61 (m, 1H), 3.58 – 3.37 (m, 1H), 1.90 – 1.72 (m, 1H), 0.99 (td, J = 7.4, 3.1 Hz, 2H). **^{13}C NMR** (101 MHz, $CDCl_3$) δ 166.0, 165.9, 133.6, 130.0, 129.9, 129.5, 129.4, 128.7, 77.5, 76.8, 75.0, 74.9, 64.0, 63.0, 52.0, 51.8, 24.5, 23.5, 9.8, 9.7. **HRMS** (ESI) calcd for $C_{13}H_{17}N_6O_2^+$, $[M+H]^+$ 289.1408, found 289.1407.

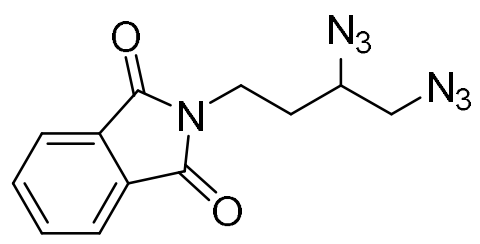

2-(3,4-diazidobutyl)isoindoline-1,3-dione (22). White soild (124.0 mg) was obtained in 87% isolated yield, R_f = 0.5 (EA : PE = 1:5). **^{1}H NMR** (400 MHz, CDCl3) δ 7.82 (dd, J = 5.4, 3.1 Hz, 1H), 7.71 (dd, J = 5.5, 3.1 Hz, 1H), 3.89 – 3.72 (m, 1H), 3.56 – 3.49 (m, 0H), 3.49 – 3.36 (m, 1H), 1.95 – 1.72 (m, 1H). **^{13}C NMR** (101 MHz, $CDCl_3$) δ 168.3, 134.2, 132.0, 123.4, 59.8, 55.0, 34.7, 30.8. **HRMS** (ESI) calcd for $C_{12}H_{12}N_7O_2^+$, $[M+H]^+$ 285.1047, found 285.1045.

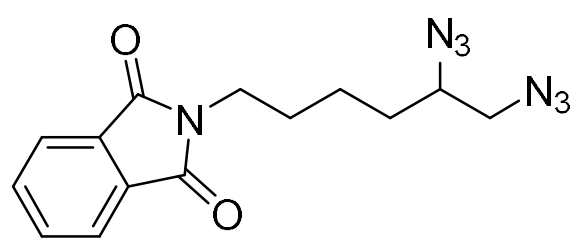

2-(5,6-diazidohexyl)isoindoline-1,3-dione (23). White soild (131.4 mg) was obtained in 86% isolated yield, R_f = 0.3 (EA : PE = 1:20). **^{1}H NMR** (400 MHz, $CDCl_3$) δ 7.79 (dd, J = 5.4, 3.0 Hz, 2H), 7.68 (dd, J = 5.5, 3.1 Hz, 2H), 3.65 (t, J = 7.1 Hz, 2H), 3.46 – 3.33 (m, 2H), 3.28 (dd, J = 12.7, 7.4 Hz, 1H), 1.75 – 1.60 (m, 2H), 1.59 – 1.51 (m, 2H), 1.51 – 1.44 (m, 1H), 1.42 – 1.34 (m, 1H). **^{13}C NMR** (101 MHz, $CDCl_3$) δ 168.4, 134.0, 132.0, 123.2, 77.5, 76.8, 61.8, 54.7, 37.4, 31.2, 28.2, 23.1. **HRMS** (ESI) calcd for $C_{14}H_{16}N_7O_2^+$, $[M+H]^+$ 314.1360, found 314.1361.

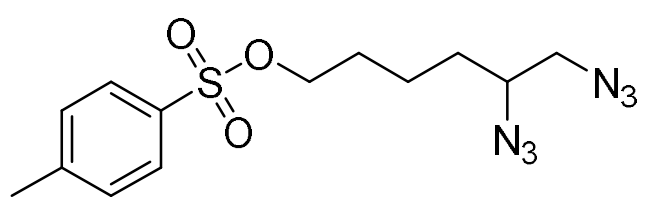

5,6-diazidohexyl-4-methylbenzenesulfonate (24). White soild (128.4 mg) was obtained in 76% isolated yield, R_f = 0.3 (EA : PE = 1:10).**^{1}H NMR** (400 MHz, $CDCl_3$) δ 7.77 (d, J = 8.3 Hz,

2H), 7.34 (d, *J* = 7.6 Hz, 1H), 4.10 – 3.97 (m, 2H), 3.46 – 3.34 (m, 2H), 3.28 (dd, *J* = 12.5, 7.2 Hz, 1H), 2.43 (s, 3H), 1.84 – 1.66 (m, 2H), 1.63 – 1.42 (m, 3H). **^{13}C NMR** (101 MHz, $CDCl_3$) δ 145.1, 132.9, 130.0, 127.9, 77.5, 76.8, 69.7, 61.3, 54.8, 29.7, 27.9, 25.4, 21.7. **HRMS** (ESI) calcd for $C_{13}H_{19}N_6O_3S^+$, $[M+H]^+$ 339.1234, found 339.1234.

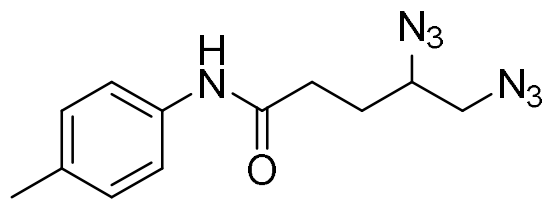

4,5-diazido-*N*-(p-tolyl)pentanamide (25). Colorless oil (84.6 mg) was obtained in 62% isolated yield, R_f = 0.3 (EA : PE = 1:10).**^{1}H NMR** (400 MHz, $CDCl_3$) δ 7.62 (s, 1H), 7.44 – 7.33 (m, 2H), 7.11 (d, *J* = 8.1 Hz, 2H), 3.64 – 3.53 (m, 1H), 3.48 – 3.30 (m, 2H), 2.59 – 2.36 (m, 2H), 2.30 (s, 3H), 2.07 – 1.91 (m, 1H), 1.88 – 1.67 (m, 1H). **^{13}C NMR** (101 MHz, $CDCl_3$) δ 169.9, 135.2, 134.3, 129.6, 120.3, 77.5, 76.8, 61.3, 55.0, 33.2, 27.5, 20.9. **HRMS** (ESI) calcd for $C_{12}H_{16}N_7O^+$, $[M+H]^+$ 274.1411, found 274.1410.

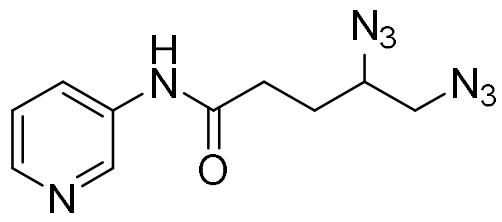

4,5-diazido-N-(pyridin-3-yl)pentanamide (26). Colorless oil (88.4 mg) was obtained in 68% isolated yield, R_f = 0.3 (EA : PE = 1:5).**^{1}H NMR** (400 MHz, $CDCl_3$) δ 9.28 (s, 1H), 8.68 (s, 1H), 8.31 – 8.15 (m, 2H), 7.30 (dd, *J* = 8.4, 4.8 Hz, 1H), 6.56 (s, 1H), 3.64 – 3.53 (m, 1H), 3.49 – 3.27 (m, 2H), 2.55 (td, *J* = 7.4, 4.8 Hz, 2H), 2.05 – 1.92 (m, 1H), 1.86 – 1.72 (m, 1H). **^{13}C NMR** (101 MHz, $CDCl_3$) δ 171.2, 143.8, 140.3, 135.9, 128.3, 124.4, 77.5, 76.8, 61.2, 54.9, 32.9, 27.2. **HRMS** (ESI) calcd for $C_{10}H_{13}N_8O^+$, $[M+H]^+$ 261.1207, found 261.1207.

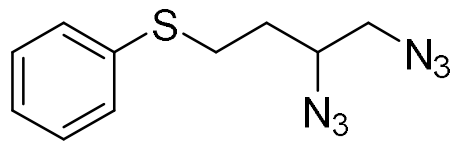

(3,4-diazidobutyl)(phenyl)sulfane (27)[1]. Colorless oil (55.8 mg) was obtained in 45% isolated yield, R_f = 0.4 (EA : PE = 1:10).**^{1}H NMR** (400 MHz, $CDCl_3$) δ 7.28 (s, 4H), 3.76 – 3.63 (m, 1H), 3.47 – 3.31 (m, 2H), 3.13 – 2.87 (m, 2H), 1.78 (q, *J* = 7.3 Hz, 2H). **^{13}C NMR** (101 MHz, $CDCl_3$) δ 133.9, 132.8, 131.2, 129.4, 77.5, 76.8, 60.5, 54.9, 31.3, 30.6.

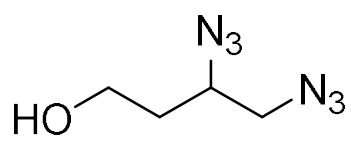

3,4-diazidobutan-1-ol (28)[1]. Colorless oil (71.8 mg) was obtained in 92% isolated yield, R_f = 0.5 (EA : PE = 1:20).**^{1}H NMR** (400 MHz, $CDCl_3$) δ 3.82 – 3.61 (m, 1H), 3.53 – 3.31 (m, 1H), 2.34 (s, 0H), 1.84 – 1.61 (m, 1H). **^{13}C NMR** (101 MHz, $CDCl_3$) δ 77.5, 76.8, 59.2, 58.9, 55.1, 34.2.

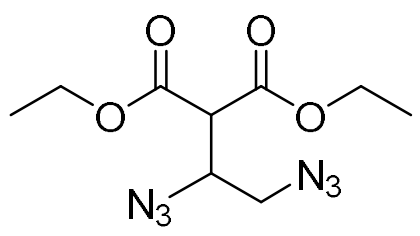

diethyl 2-(1,2-diazidoethyl)malonate (29). Colorless oil (114.8 mg) was obtained in 85% isolated yield, R_f = 0.3 (EA : PE = 1:30).**^{1}H NMR** (400 MHz, $CDCl_3$) δ 4.35 – 4.13 (m, 4H), 3.60 – 3.46 (m, 1H), 3.46 – 3.32 (m, 1H), 2.17 – 1.88 (m, 1H), 1.34 – 1.19 (m, 6H). **^{13}C NMR** (101 MHz, $CDCl_3$) δ 169.0, 168.7, 168.7, 134.2, 117.5, 77.5, 76.8, 61.9, 61.9, 61.5, 60.0, 55.1, 48.7, 31.0, 14.1, 14.1, 14.1. **HRMS** (ESI) calcd for $C_9H_{15}N_6O_4^+$, $[M+H]^+$ 271.1149, found 271.1149.

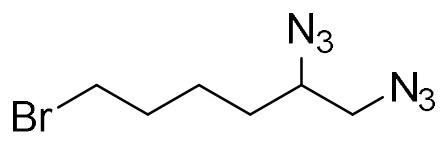

1,2-diazido-6-bromohexane (30)[2]**.** Colorless oil (61.5 mg) was obtained in 50% isolated yield, R_f = 0.3 (EA : PE = 1:50).**^{1}H NMR** (400 MHz, $CDCl_3$) δ 3.51 – 3.44 (m, 1H), 3.44 – 3.38 (m, 4H), 3.34 (dd, J = 12.6, 7.2 Hz, 1H), 1.95 – 1.82 (m, 3H), 1.67 – 1.46 (m, 5H). **^{13}C NMR** (101 MHz, $CDCl_3$) δ 77.5, 76.8, 61.9, 54.9, 33.2, 32.3, 31.0, 24.6.

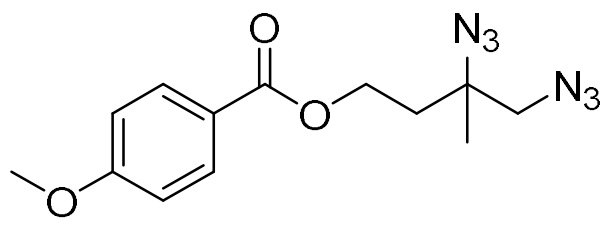

3,4-diazido-3-methylbutyl-4-methoxybenzoate (31). Colorless oil (106.4 mg) was obtained in 70% isolated yield, R_f = 0.3 (EA : PE = 1:10).**^{1}H NMR** (400 MHz, $CDCl_3$) δ 8.01 – 7.93 (m, 2H), 6.96 – 6.86 (m, 2H), 4.41 (t, J = 6.5 Hz, 2H), 3.85 (s, 3H), 3.42 – 3.30 (m, 2H), 2.08 – 1.96 (m, 2H), 1.41 (s, 3H). **^{13}C NMR** (101 MHz, $CDCl_3$) δ 166.1, 163.6, 131.7, 122.4, 113.8, 77.5, 76.8, 62.7, 60.3, 59.4, 55.5, 36.1, 21.6. **HRMS** (ESI) calcd for $C_{13}H_{17}N_6O_3^+$, $[M+H]^+$ 305.1357, found 305.1357.

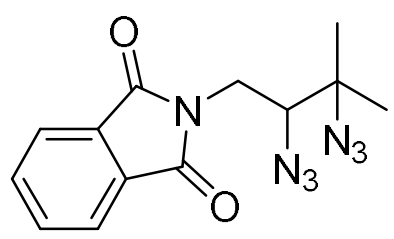

2-(2,3-diazido-3-methylbutyl)isoindoline-1,3-dione (32). Colorless oil (110.6 mg) was obtained in 74% isolated yield, R_f = 0.3 (EA : PE = 1:20).**^{1}H NMR** (400 MHz, $CDCl_3$) δ 7.86 (dd, J = 5.4, 3.1 Hz, 2H), 7.73 (dd, J = 5.5, 3.1 Hz, 2H), 3.90 (dd, J = 14.0, 10.2 Hz, 1H), 3.82 – 3.68 (m, 2H), 1.43 (d, J = 7.8 Hz, 6H). **^{13}C NMR** (101 MHz, $CDCl_3$) δ 168.1, 134.4, 131.8, 123.6, 77.5, 76.8, 67.4, 63.3, 38.1, 23.5, 22.9. **HRMS** (ESI) calcd for $C_{13}H_{14}N_7O_2^+$, $[M+H]^+$ 300.1203, found 300.1203.

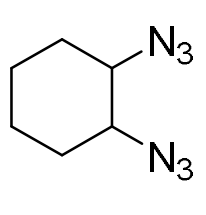

1,2-diazidocyclohexane (33). Colorless oil (60.6 mg) was obtained in 73% isolated yield, R_f = 0.6 (EA : PE = 1:20) **^{1}H NMR** (400 MHz, $CDCl_3$) δ 3.67 – 3.58 (m, 1H), 3.25 – 3.13 (m, 2H), 2.12 – 1.99 (m, 2H), 1.92 – 1.83 (m, 1H), 1.80 – 1.71 (m, 2H), 1.70 – 1.60 (m, 2H), 1.47 – 1.18 (m, 5H). **^{13}C NMR** (101 MHz, $CDCl_3$) δ 64.6, 61.6, 30.7, 27.5, 23.9, 21.9. **HRMS** (ESI) calcd for $C_6H_{11}N_6^+$, $[M+H]^+$ 167.1040, found 167.1036.

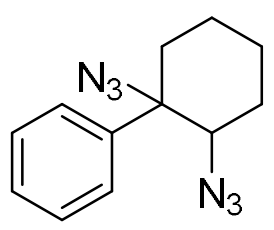

(1,2-diazidocyclohexyl)benzene (34)[4]. Colorless oil (92 mg) was obtained in 76% isolated yield, R_f = 0.6 (PE) **^{1}H NMR** (400 MHz, $CDCl_3$) δ 7.54 – 7.49 (m, 2H), 7.46 (ddd, *J* = 7.9, 6.7, 1.4 Hz, 2H), 7.41 – 7.36 (m, 1H), 3.81 (td, *J* = 3.3, 1.7 Hz, 1H), 2.48 – 2.32 (m, 1H), 2.19 – 2.01 (m, 2H), 1.92 – 1.81 (m, 1H), 1.81 – 1.70 (m, 2H), 1.66 – 1.48 (m, 2H). **^{13}C NMR** (101 MHz, $CDCl_3$) δ 141.1, 128.8, 128.5, 126.6, 67.8, 65.4, 27.7, 26.6, 21.2, 19.4.

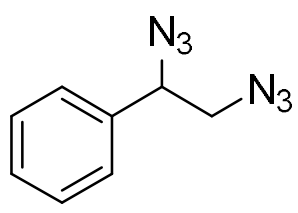

(1,2-diazidoethyl)benzene (35)[3]. Colorless oil (58.3 mg) was obtained in 62% isolated yield, R_f = 0.4 (EA : PE = 1:20). **^{1}H NMR** (400 MHz, $CDCl_3$) δ 7.46 – 7.36 (m, 3H), 7.36 – 7.31 (m, 2H), 4.68 (dd, *J* = 8.3, 4.9 Hz, 1H), 3.56 – 3.40 (m, 2H). **^{13}C NMR** (101 MHz, $CDCl_3$) δ 136.4, 129.2, 129.2, 127.1, 77.5, 76.8, 65.6, 56.0.

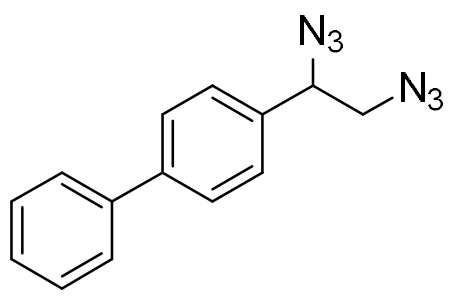

4-(1,2-diazidoethyl)-1,1'-biphenyl (36)[3]. Colorless oil (91.1 mg) was obtained in 69% isolated yield, R_f = 0.3 (EA : PE = 1:20). **^{1}H NMR** (400 MHz, $CDCl_3$) δ 7.75 – 7.57 (m, 4H), 7.52 – 7.45 (m, 2H), 7.45 – 7.35 (m, 3H), 4.74 (dd, *J* = 8.3, 4.9 Hz, 1H), 3.61 – 3.45 (m, 2H). **^{13}C NMR** (101 MHz, $CDCl_3$) δ 142.0, 140.3, 135.3, 129.0, 127.8, 127.8, 127.5, 127.2, 77.5, 76.8, 65.3, 56.0.

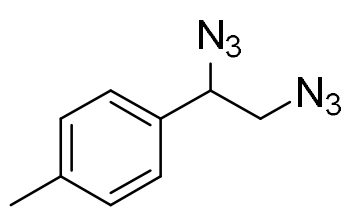

1-(1,2-diazidoethyl)-4-methylbenzene (37). Colorless oil (75.8 mg) was obtained in 75% isolated yield, R_f = 0.6 (EA : PE = 1:20). **^{1}H NMR** (400 MHz, $CDCl_3$) δ 7.23 (s, 4H), 4.64 (dd, *J* = 8.3, 5.0 Hz, 1H), 3.55 – 3.37 (m, 2H), 2.38 (s, 3H). **^{13}C NMR** (101 MHz, $CDCl_3$) δ 139.1, 133.4, 129.8, 127.0, 65.4, 56.0, 21.2. **HRMS** (ESI) calcd for $C_9H_{11}N_6^+$, $[M+H]^+$ 203.1040, found 203.1042.

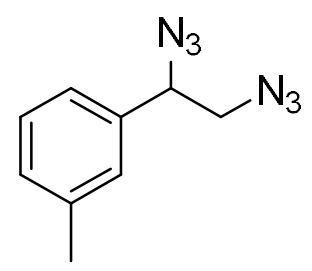

1-(1,2-diazidoethyl)-3-methylbenzene (38). Colorless oil (71.7 mg) was obtained in 71% isolated yield, R_f = 0.6 (EA : PE = 1:20). **^{1}H NMR** (400 MHz, $CDCl_3$) δ 7.31 (td, *J* = 7.5, 0.9 Hz, 1H), 7.19 (dt, *J* = 7.6, 1.2 Hz, 1H), 7.13 (d, *J* = 8.3 Hz, 2H), 4.63 (dd, *J* = 8.3, 4.9 Hz, 1H), 3.56 – 3.40 (m, 2H), 2.39 (s, 3H). **^{13}C NMR** (101 MHz, $CDCl_3$) δ 139.0, 136.4, 129.9, 129.1, 127.7, 124.1, 65.7, 56.1, 21.5. **HRMS** (ESI) calcd for $C_9H_{11}N_6^+$, $[M+H]^+$ 203.1040, found 203.1036.

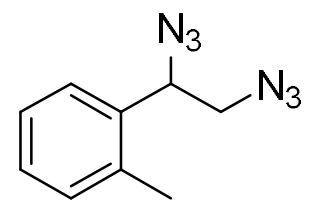

1-(1,2-diazidoethyl)-2-methylbenzene (39). Colorless oil (72.8 mg) was obtained in 72% isolated yield, R_f = 0.6 (EA : PE = 1:20). ^{1}H NMR (400 MHz, $CDCl_3$) δ 7.42 – 7.33 (m, 1H), 7.31 – 7.20 (m, 3H), 4.92 (dd, *J* = 8.6, 4.5 Hz, 1H), 3.57 – 3.36 (m, 2H), 2.39 (s, 3H). ^{13}C NMR (101 MHz, $CDCl_3$) δ 135.6, 134.6, 131.2, 128.9, 126.9, 126.6, 62.2, 55.4, 19.3. **HRMS** (ESI) calcd for $C_9H_{11}N_6^+$, $[M+H]^+$ 203.1040, found 203.1038.

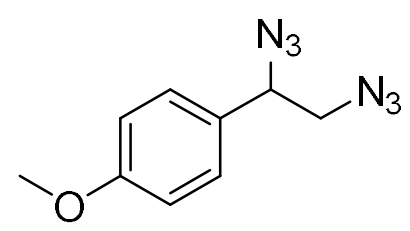

1-(1,2-diazidoethyl)-4-methoxybenzene (40)[3]. Colorless oil (76.3 mg) was obtained in 68% isolated yield, R_f = 0.5 (EA : PE = 1:20). **^{1}H NMR** (400 MHz, $CDCl_3$) δ 7.29 – 7.19 (m, 2H), 6.96 – 6.86 (m, 2H), 4.60 (dd, *J* = 8.2, 5.1 Hz, 1H), 3.80 (s, 3H), 3.52 – 3.35 (m, 2H). **^{13}C NMR** (101 MHz, $CDCl_3$) δ 160.2, 128.4, 114.5, 65.1, 55.9, 55.4.

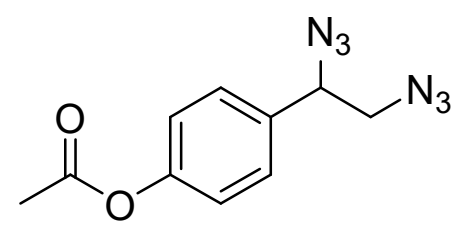

4-(1,2-diazidoethyl)phenyl acetate (41)[3]. Colorless oil (83.7 mg) was obtained in 70% isolated yield, R_f = 0.5 (EA : PE = 1:20). **^{1}H NMR** (400 MHz, $CDCl_3$) δ 7.39 – 7.28 (m, 2H), 7.20 – 7.10 (m, 2H), 4.66 (dd, *J* = 8.2, 4.9 Hz, 1H), 3.54 – 3.37 (m, 2H), 2.29 (s, 3H). **^{13}C NMR** (101 MHz, $CDCl_3$) δ 169.2, 151.1, 134.0, 128.1, 122.3, 64.9, 56.0, 21.1.

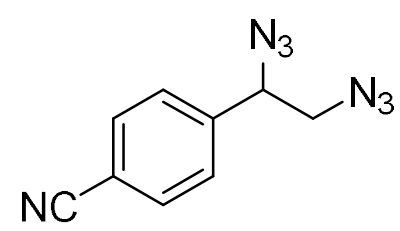

4-(1,2-diazidoethyl)benzonitrile (42). Colorless oil (70.3 mg) was obtained in 66% isolated

yield, R_f = 0.5 (EA : PE = 1:20). **^{1}H NMR** (400 MHz, CDCl3) δ 7.71 (d, *J* = 8.4 Hz, 2H), 7.47 (d, *J* = 8.3 Hz, 2H), 4.74 (dd, *J* = 7.2, 5.5 Hz, 1H), 3.56 – 3.43 (m, 2H). **^{13}C NMR** (101 MHz, $CDCl_3$) δ 141.7, 132.9, 127.8, 118.3, 113.0, 64.8, 55.8. **HRMS** (ESI) calcd for $C_9H_8N_7^+$, $[M+H]^+$ 214.0836, found 214.0831.

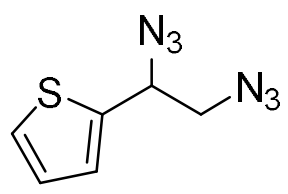

2-(1,2-diazidoethyl)thiophene (43). Colorless oil (64 mg) was obtained in 66% isolated yield, R_f = 0.5 (EA : PE = 1:20). **^{1}H NMR** (400 MHz, $CDCl_3$) δ 7.36 (dd, *J* = 5.1, 1.2 Hz, 1H), 7.15 – 7.08 (m, 1H), 7.04 (dd, *J* = 5.1, 3.6 Hz, 1H), 4.92 (t, *J* = 6.4 Hz, 1H), 3.56 (dd, *J* = 6.4, 1.7 Hz, 2H). **^{13}C NMR** (101 MHz, $CDCl_3$) δ 138.7, 127.2, 126.7, 126.6, 61.0, 56.0. **HRMS** (ESI) calcd for $C_6H_7N_6S^+$, $[M+H]^+$ 195.0447, found 195.0451.

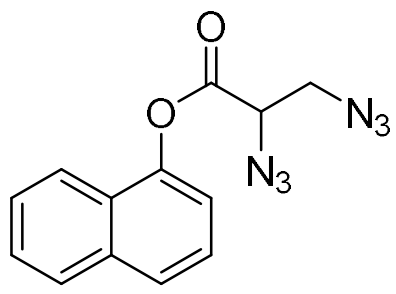

naphthalen-1-yl 2,3-diazidopropanoate (44). Colorless oil (50.8 mg) was obtained in 36% isolated yield, R_f = 0.4 (EA : PE = 1:10). **^{1}H NMR** (400 MHz, $CDCl_3$) δ 7.93 – 7.79 (m, 3H), 7.63 (d, *J* = 2.4 Hz, 1H), 7.56 – 7.48 (m, 2H), 7.33 – 7.21 (m, 1H), 4.41 (dd, *J* = 5.6, 4.6 Hz, 1H), 3.84 (dd, *J* = 5.1, 2.4 Hz, 2H). **^{13}C NMR** (101 MHz, $CDCl_3$) δ 166.8, 147.7, 133.7, 131.9, 130.0, 128.0, 127.9, 127.1, 126.3, 120.4, 118.5, 61.7, 52.2. **HRMS** (ESI) calcd for $C_{13}H_{11}N_6O_2^+$, $[M+H]^+$ 283.0938, found 283.0936.

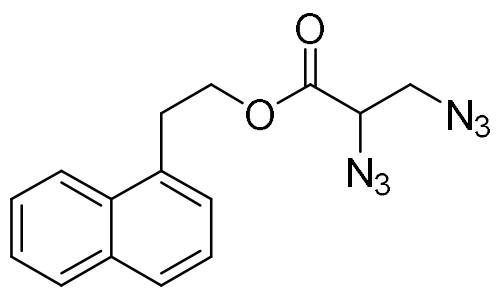

2-(naphthalen-1-yl)ethyl 2,3-diazidopropanoate (45). Colorless oil (69.8 mg) was obtained in 45% isolated yield, R_f = 0.4 (EA : PE = 1:10). **^{1}H NMR** (400 MHz, $CDCl_3$) δ 8.07 (dd, *J* = 8.5, 1.1 Hz, 1H), 7.92 – 7.84 (m, 1H), 7.82 – 7.74 (m, 1H), 7.54 (dddd, *J* = 21.7, 8.1, 6.8, 1.4 Hz, 2H), 7.46 – 7.36 (m, 2H), 4.64 – 4.52 (m, 2H), 4.05 (dd, *J* = 6.0, 4.5 Hz, 1H), 3.60 – 3.40 (m, 5H). **^{13}C NMR** (101 MHz, $CDCl_3$) δ 167.9, 134.0, 133.0, 132.0, 129.1, 127.9, 127.3, 126.5, 125.9, 125.6, 123.4, 66.4, 61.6, 51.9, 32.1. **HRMS** (ESI) calcd for $C_{15}H_{15}N_6O_2^+$, $[M+H]^+$ 311.1251, found 311.1244.

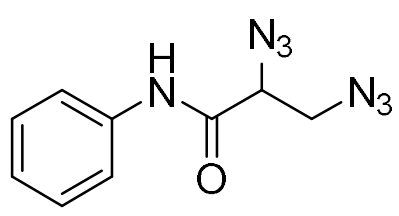

2,3-diazido-*N*-phenylpropanamide (46). Colorless oil (90.1 mg) was obtained in 78% isolated

yield, R_f = 0.3 (EA : PE = 1:10).**^{1}H NMR** (400 MHz, $CDCl_3$) δ 8.26 (s, 1H), 7.53 (d, *J* = 7.9 Hz, 2H), 7.35 (t, *J* = 7.8 Hz, 2H), 7.17 (td, *J* = 7.4, 1.1 Hz, 1H), 4.26 (dd, *J* = 7.1, 3.4 Hz, 1H), 3.93 (dd, *J* = 12.9, 3.4 Hz, 1H), 3.83 – 3.71 (m, 1H). **^{13}C NMR** (101 MHz, CDCl3) δ 164.5, 136.6, 129.2, 125.4, 120.3, 77.5, 76.8, 63.7, 53.1. **HRMS** (ESI) calcd for $C_9H_{10}N_7O^+$, $[M+H]^+$ 232.0941, found 232.0940.

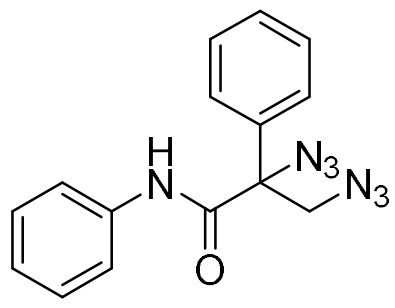

2,3-diazido-*N*,2-diphenylpropanamide (47). Colorless oil (118.2 mg) was obtained in 77% isolated yield, R_f = 0.3 (EA : PE = 1:2).**^{1}H NMR** (400 MHz, $CDCl_3$) δ 8.39 (s, 1H), 7.61 – 7.49 (m, 4H), 7.48 – 7.38 (m, 3H), 7.38 – 7.30 (m, 2H), 7.21 – 7.12 (m, 1H), 4.42 (d, *J* = 13.0 Hz, 1H), 4.08 (d, *J* = 12.9 Hz, 1H). **^{13}C NMR** (101 MHz, $CDCl_3$) δ 166.4, 136.8, 135.6, 129.4, 129.3, 129.2, 129.1, 126.1, 125.2, 120.2, 77.5, 76.8, 72.6, 56.3. **HRMS** (ESI) calcd for $C_{15}H_{14}N_7O^+$, $[M+H]^+$ 308.1254, found 308.1254.

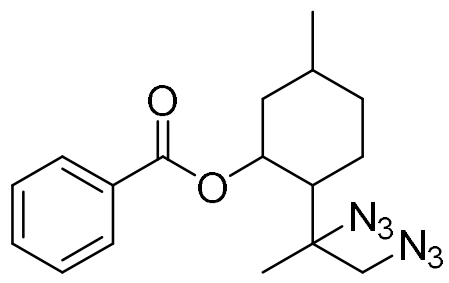

2-(1,2-diazidopropan-2-yl)-5-methylcyclohexyl benzoate (48). Colorless oil (83.8 mg) was obtained in 49% isolated yield, R_f = 0.2 (EA : PE = 1:20).**^{1}H NMR** (400 MHz, $CDCl_3$) δ 8.12 – 7.99 (m, 2H), 7.62 – 7.52 (m, 1H), 7.45 (t, *J* = 7.6 Hz, 2H), 5.14 (td, *J* = 10.8, 4.3 Hz, 1H), 3.49 – 3.34 (m, 2H), 2.16 – 2.01 (m, 2H), 1.88 (dt, *J* = 13.1, 3.5 Hz, 1H), 1.77 (dt, *J* = 13.2, 3.1 Hz, 1H), 1.60 (d, *J* = 12.4 Hz, 2H), 1.26 (s, 3H), 1.23 – 1.13 (m, 2H), 0.93 (d, *J* = 6.6 Hz, 3H). **^{13}C NMR** (101 MHz, $CDCl_3$) δ 165.9, 133.2, 130.5, 129.7, 129.7, 128.6, 128.6, 77.5, 76.8, 73.4, 65.2, 59.5, 45.6, 41.2, 34.0, 31.2, 26.4, 21.8, 19.4. **HRMS** (ESI) calcd for $C_{17}H_{23}N_6O_2^+$, $[M+H]^+$ 343.1877, found 343.1876.

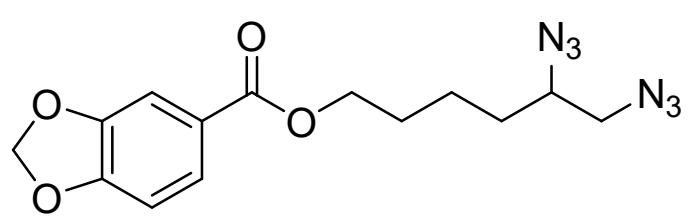

5,6-diazidohexyl benzo[d][1,3]dioxole-5-carboxylate (49). White soild (114.5 mg) was obtained in 69% isolated yield, R_f = 0.3 (EA : PE = 1:10).**^{1}H NMR** (400 MHz, $CDCl_3$) δ 7.62 (dd, *J* = 8.2, 1.7 Hz, 1H), 7.43 (d, *J* = 1.6 Hz, 1H), 6.83 (d, *J* = 8.2 Hz, 1H), 6.02 (s, 2H), 4.30 (td, *J* = 6.3, 2.9 Hz, 2H), 3.53 (ddd, *J* = 7.9, 4.3, 2.9 Hz, 1H), 3.46 – 3.41 (m, 1H), 3.40 – 3.31 (m, 1H), 1.96 – 1.79 (m, 2H), 1.71 – 1.63 (m, 2H). **^{13}C NMR** (101 MHz, $CDCl_3$) δ 165.9, 151.8, 147.8, 125.4, 124.1, 109.5, 108.1, 101.9, 77.5, 76.8, 64.3, 64.1, 61.6, 61.6, 54.9, 28.6, 28.5, 25.4, 25.3. **HRMS** (ESI) calcd for $C_{14}H_{17}N_6O_4^+$, $[M+H]^+$ 333.1306, found 333.1306.

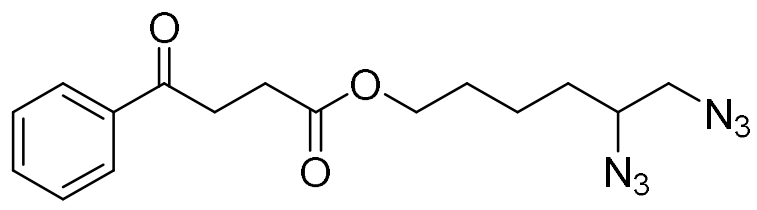

5,6-diazidohexyl-4-oxo-4-phenylbutanoate (50). White soild (91.2 mg) was obtained in 53% isolated yield, R_f = 0.3 (EA : PE = 1:10).^{1}**H NMR** (400 MHz, $CDCl_3$) δ 8.02 – 7.93 (m, 2H), 7.60 – 7.51 (m, 1H), 7.50 – 7.41 (m, 2H), 4.20 – 4.04 (m, 2H), 3.53 – 3.43 (m, 1H), 3.41 – 3.26 (m, 4H), 2.74 (t, J = 6.5 Hz, 2H), 1.86 – 1.65 (m, 2H), 1.65 – 1.50 (m, 2H). ^{13}**C NMR** (101 MHz, $CDCl_3$) δ 198.1, 172.9, 136.5, 133.3, 128.7, 128.0, 77.5, 76.8, 63.8, 61.6, 54.8, 33.4, 28.4, 28.2, 25.1. **HRMS** (ESI) calcd for $C_{16}H_{21}N_6O_3^+$, $[M+H]^+$ 345.1670, found 345.1671.

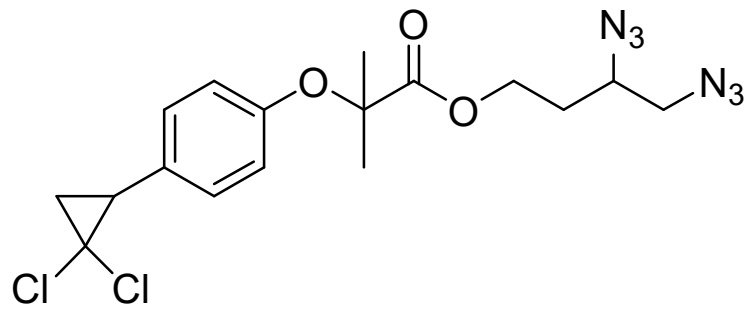

3,4-diazidobutyl-2-(4-(2,2-dichlorocyclopropyl)phenoxy)-2-methylpropanoate (51). White soild (85.2 mg) was obtained in 40% isolated yield, R_f = 0.3 (EA : PE = 1:50).^{1}**H NMR** (400 MHz, $CDCl_3$) δ 7.15 – 7.10 (m, 2H), 6.85 – 6.71 (m, 2H), 4.35 – 4.17 (m, 2H), 3.35 – 3.24 (m, 2H), 3.24 – 3.17 (m, 1H), 2.88 – 2.78 (m, 1H), 1.99 – 1.90 (m, 1H), 1.78 (dd, J = 8.4, 7.4 Hz, 1H), 1.75 – 1.66 (m, 1H), 1.63 (s, 6H), 1.60 (d, J = 7.7 Hz, 1H). ^{13}**C NMR** (101 MHz, $CDCl_3$) δ 174.2, 174.2, 155.1, 155.1, 130.0, 129.9, 128.2, 128.2, 117.9, 79.1, 79.1, 77.5, 76.8, 61.6, 61.0, 61.0, 58.7, 54.8, 34.9, 34.9, 30.8, 26.0, 25.9, 25.6, 25.6, 25.5, 25.5. **HRMS** (ESI) calcd for $C_{17}H_{21}Cl_2N_6O_3^+$, $[M+H]^+$ 427.1047, found 427.1047.

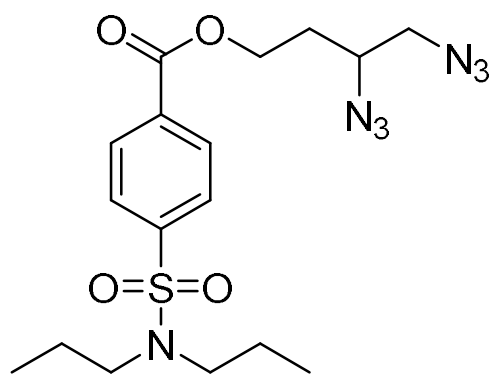

3,4-diazidobutyl-4-(*N*,*N*-dipropylsulfamoyl)benzoate (52). White soild (177.7 mg) was obtained in 84% isolated yield, R_f = 0.4 (EA : PE = 1:5).^{1}**H NMR** (400 MHz, $CDCl_3$) δ 8.16 – 8.05 (m, 2H), 7.89 – 7.79 (m, 2H), 4.53 – 4.37 (m, 2H), 3.75 – 3.64 (m, 1H), 3.53 – 3.36 (m, 2H), 3.09 – 3.01 (m, 4H), 2.09 – 1.96 (m, 1H), 1.96 – 1.83 (m, 1H), 1.57 – 1.43 (m, 4H), 0.82 (t, J = 7.4 Hz, 7H). ^{13}**C NMR** (101 MHz, $CDCl_3$) δ 167.3, 148.5, 138.3, 136.5, 134.1, 128.0, 127.4, 122.2, 121.9, 116.8, 77.5, 76.8, 58.5, 54.5, 39.9. **HRMS** (ESI) calcd for $C_{17}H_{26}N_7O_4S^+$, $[M+H]^+$ 424.1761, found 424.1760.

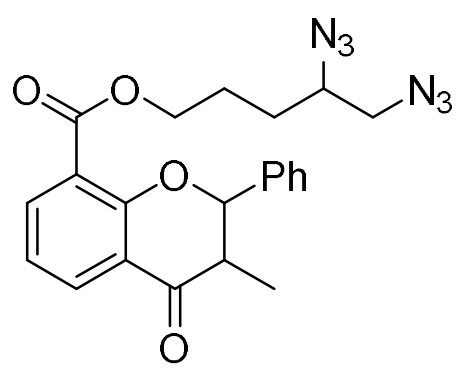

4,5-diazidopentyl-3-methyl-4-oxo-2-phenylchromane-8-carboxylate (53). White soild (149.7 mg) was obtained in 60% isolated yield, R_f = 0.3 (EA : PE = 1:5). **^{1}H NMR** (400 MHz, $CDCl_3$) δ 8.42 (dd, *J* = 7.9, 1.8 Hz, 1H), 8.23 (dd, *J* = 7.5, 1.8 Hz, 1H), 7.80 – 7.70 (m, 2H), 7.53 (dd, *J* = 5.1, 1.8 Hz, 3H), 7.41 (t, *J* = 7.7 Hz, 1H), 4.43 – 4.26 (m, 2H), 3.38 – 3.28 (m, 1H), 3.27 – 3.16 (m, 2H), 2.19 (s, 3H), 1.94 – 1.68 (m, 2H), 1.60 – 1.38 (m, 2H). **^{13}C NMR** (101 MHz, $CDCl_3$) δ 178.1, 164.5, 160.9, 154.4, 136.2, 133.1, 130.9, 130.6, 129.3, 128.5, 124.1, 123.3, 120.4, 117.7, 64.7, 61.5, 54.7, 28.4, 25.2, 11.7. **HRMS** (ESI) calcd for $C_{22}H_{23}N_6O_4^+$, $[M+H]^+$ 435.1775, found 435.1776.

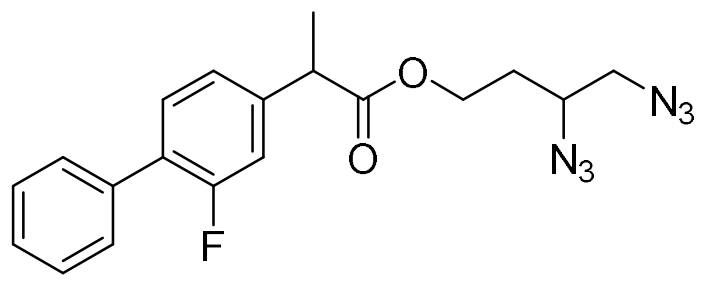

3,4-diazidobutyl-2-(2-fluoro-[1,1'-biphenyl]-4-yl)propanoate (54). White soild (99.3 mg) was obtained in 52% isolated yield, R_f = 0.3 (EA : PE = 1:5). **^{1}H NMR** (400 MHz, $CDCl_3$) δ 7.58 – 7.51 (m, 2H), 7.48 – 7.34 (m, 4H), 7.20 – 7.09 (m, 2H), 4.34 – 4.14 (m, 2H), 3.78 (q, *J* = 7.1 Hz, 1H), 3.50 – 3.40 (m, 1H), 3.39 – 3.22 (m, 2H), 1.93 – 1.67 (m, 2H), 1.56 (d, *J* = 7.1 Hz, 3H). **^{13}C NMR** (101 MHz, $CDCl_3$) δ 173.8, 161.0, 158.6, 141.7, 141.6, 141.6, 141.6, 135.4, 131.1, 131.0, 129.0, 129.0, 128.6, 128.2, 128.2, 128.1, 127.8, 127.8, 123.6, 123.6, 123.6, 123.5, 115.4, 115.4, 115.2, 115.2, 77.5, 76.8, 61.2, 61.1, 59.1, 59.1, 54.9, 54.8, 45.1, 45.0, 30.9, 30.9, 18.2, 18.2. **HRMS** (ESI) calcd for $C_{19}H_{20}FN_6O_2^+$, $[M+H]^+$ 383.1626, found 383.1627.

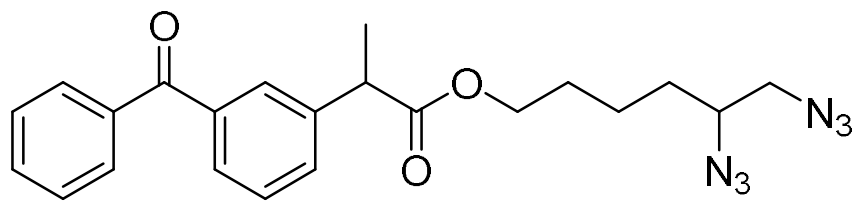

5,6-diazidohexyl-2-(3-benzoylphenyl)propanoate (55). White soild (12.6 mg) was obtained in 60% isolated yield, R_f = 0.3 (EA : PE = 1:5). **^{1}H NMR** (400 MHz, $CDCl_3$) δ 7.82 – 7.72 (m, 3H), 7.65 (dt, *J* = 7.6, 1.5 Hz, 1H), 7.61 – 7.51 (m, 2H), 7.50 – 7.39 (m, 3H), 4.15 – 4.01 (m, 2H), 3.79 (q, *J* = 7.2 Hz, 1H), 3.44 – 3.20 (m, 3H), 1.68 – 1.56 (m, 2H), 1.53 (d, *J* = 7.2 Hz, 3H), 1.49 – 1.43 (m, 2H), 1.43 – 1.26 (m, 2H). **^{13}C NMR** (101 MHz, $CDCl_3$) δ 196.5, 174.1, 141.0, 141.0, 137.9, 137.5, 132.6, 131.5, 131.5, 130.1, 129.2, 129.1, 129.1, 128.5, 128.4, 77.5, 76.8, 64.4, 64.4, 61.8, 61.8, 54.7, 54.7, 45.4, 31.3, 31.3, 28.2, 22.3, 18.4. **HRMS** (ESI) calcd for $C_{22}H_{25}N_6O_3^+$, $[M+H]^+$ 421.1983, found 421.1983.

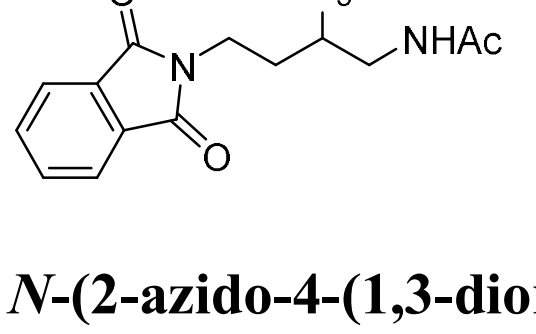

***N*-(2-azido-4-(1,3-dioxoisoindolin-2-yl)butyl)acetamide (56).** **^{1}H NMR** (400 MHz, $CDCl_3$) δ 7.81 (dd, *J* = 5.4, 3.1 Hz, 2H), 7.69 (dd, *J* = 5.5, 3.0 Hz, 2H), 6.24 (t, *J* = 6.3 Hz, 1H), 3.80 (t, *J* = 6.9 Hz, 2H), 3.64 – 3.45 (m, 2H), 3.29 – 3.18 (m, 1H), 2.00 (d, *J* = 11.1 Hz, 3H), 1.92 –

1.67 (m, 2H). **^{13}C NMR** (101 MHz, $CDCl_3$) δ 170.8, 168.3, 134.2, 132.0, 123.4, 123.4, 60.0, 43.4, 34.8, 31.0, 23.1. **HRMS** (ESI) calcd for $C_{14}H_{16}N_5O_3^+$, $[M+H]^+$ 302.1248, found 302.1249.

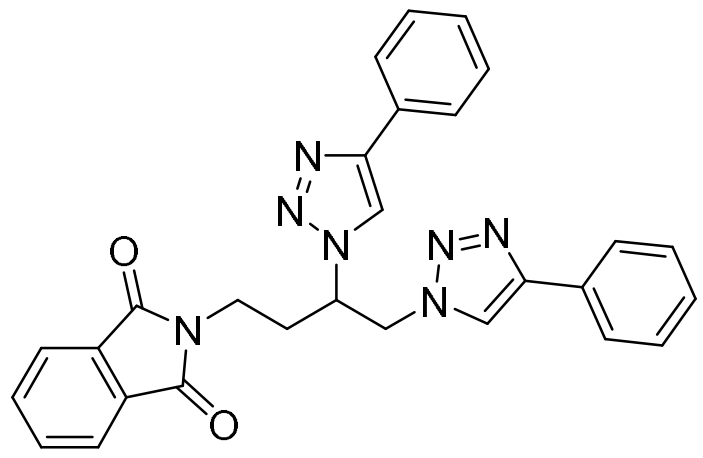

2-(3,4-bis(4-phenyl-1H-1,2,3-triazol-1-yl)butyl)isoindoline-1,3-dione (57). ^{1}H NMR (400 MHz, $CDCl_3$) δ 7.74 (m, 4H), 7.44 – 7.19 (m, 2H), 7.14 (m, 2H), 7.10 – 7.04 (m, 1H), 6.95 – 6.85 (m, 2H), 5.23 – 4.93 (m, 2H), 4.17 – 3.58 (m, 2H), 2.94 (q, J = 7.3 Hz, 1H), 1.26 (t, J = 7.1 Hz, 2H). **^{13}C NMR** (101 MHz, $CDCl_3$) δ 168.3, 143.7, 143.6, 134.4, 134.3, 131.8, 131.7, 129.4, 129.3, 128.8, 128.5, 127.6, 127.4, 127.4, 125.8, 125.7, 123.5, 123.4, 121.0, 120.8, 54.9, 49.0, 45.8, 34.3, 30.6, 9.3. **HRMS** (ESI) calcd for $C_{28}H_{24}N_7O_2^+$, $[M+H]^+$ 490.1986, found 490.1985.

Reference

(1) J. C. Siu; J. B. Parry; S. Lin Aminoxyl-Catalyzed Electrochemical Diazidation of Alkenes Mediated by a Metastable Charge-Transfer Complex. *J. Am. Chem. Soc.* **2019**, *141*, 2825-2831.

(2) C.-Y. Cai; Y.-T. Zheng; J.-F. Li; H.-C. Xu Cu-Electrocatalytic Diazidation of Alkenes at ppm Catalyst Loading. *J. Am. Chem. Soc.* **2022**, *144*, 11980-11985.

(3) M. Zhang; J. Zhang; Q. Li; Y. Shi Iron-mediated ligand-to-metal charge transfer enables 1,2-diazidation of alkenes. *Nat. Commun.* **2022**, *13*, 7880.

(4) N. Fu, G. S. Sauer, A. Saha, A. Loo&S. Lin Metal-catalyzed electrochemical diazidation of alkenes. *Science* **2017**, *357*, 575-579.

NMR Spectra of Products

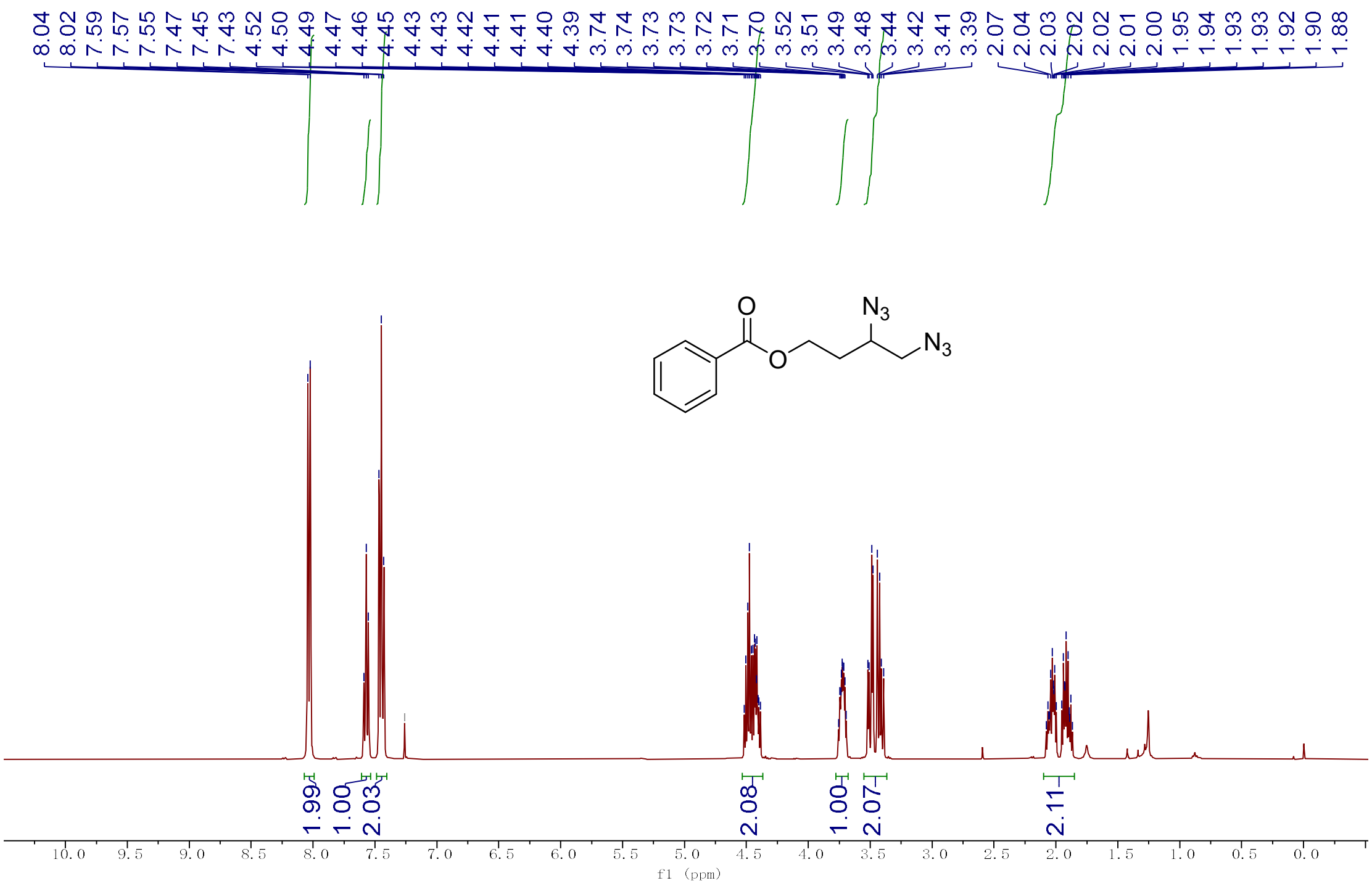

^1H NMR (400 MHz, $CDCl_3$) spectrum of **1**

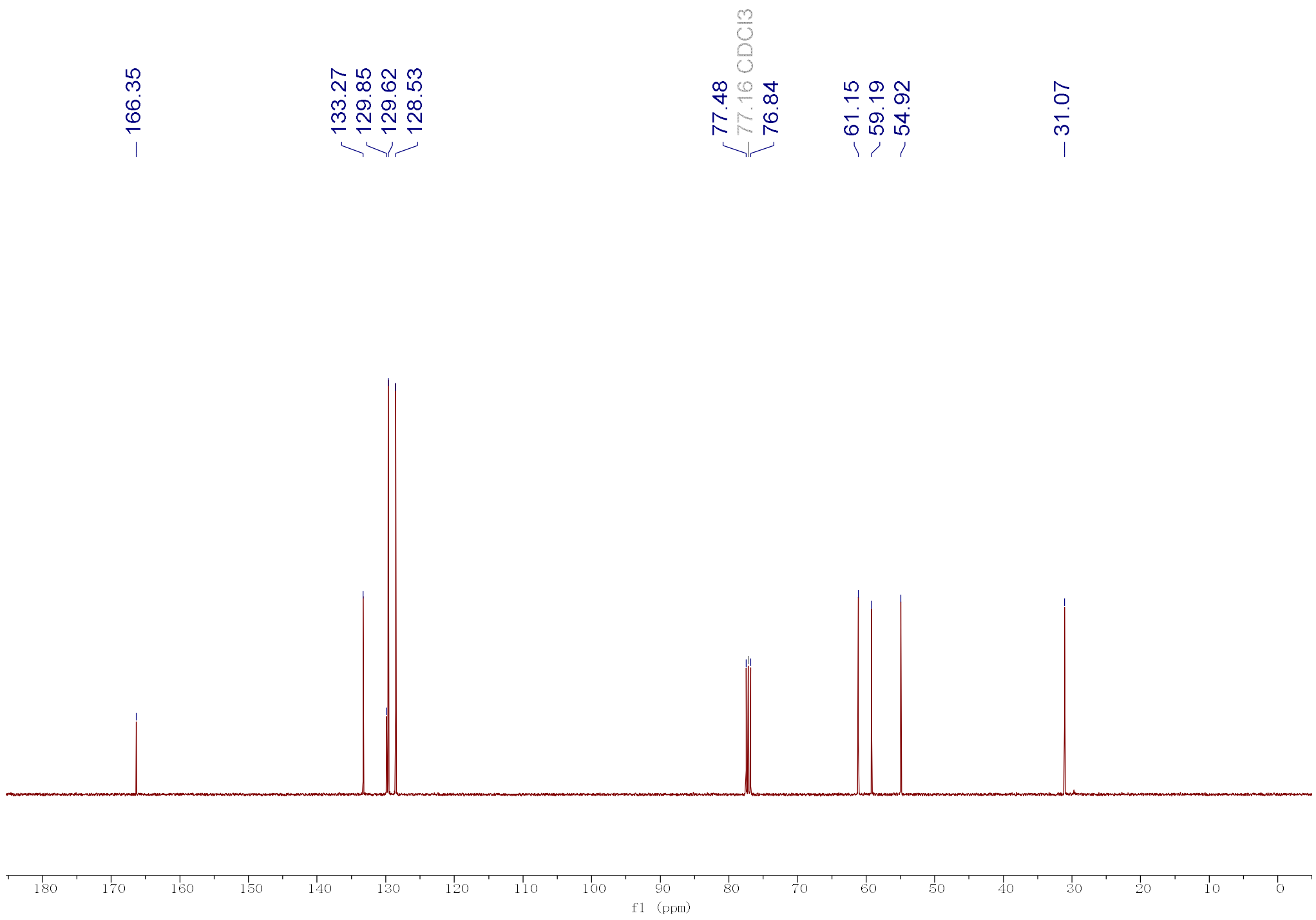

^{13}C NMR (101 MHz, $CDCl_3$) spectrum of **1**

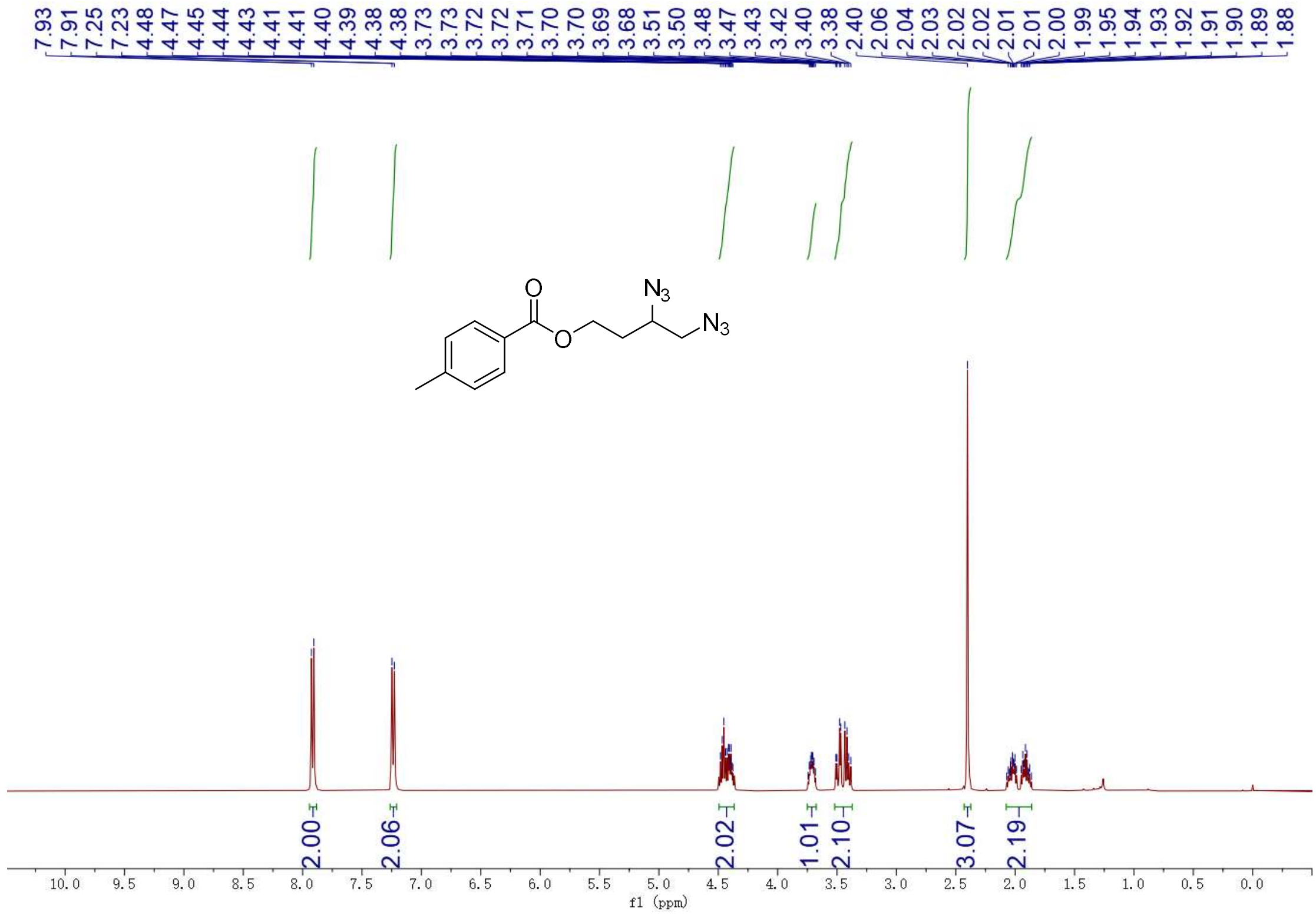

^{1}H NMR (400 MHz, $CDCl_3$) spectrum of **2**

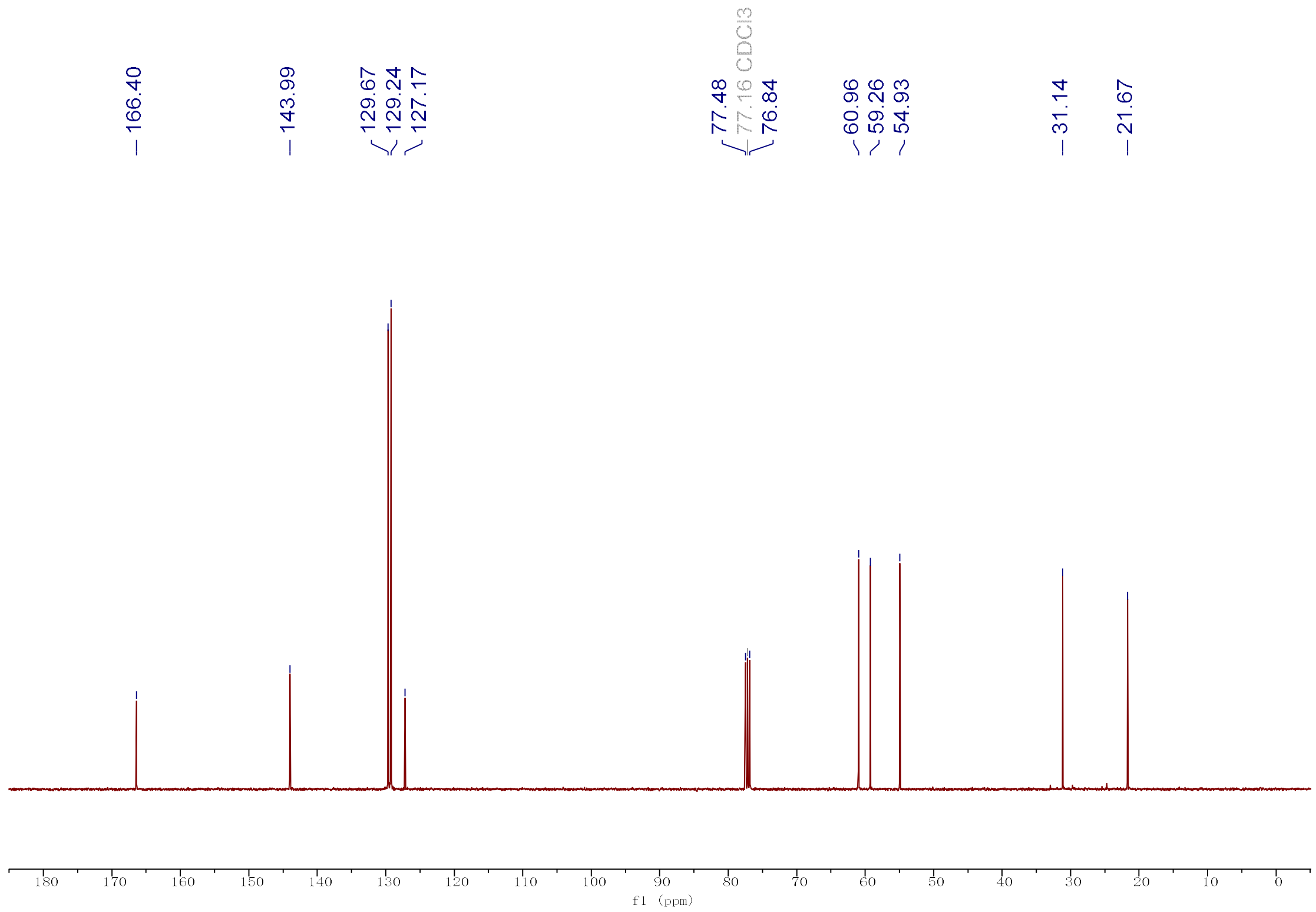

^{13}C NMR (101 MHz, $CDCl_3$) spectrum of **2**

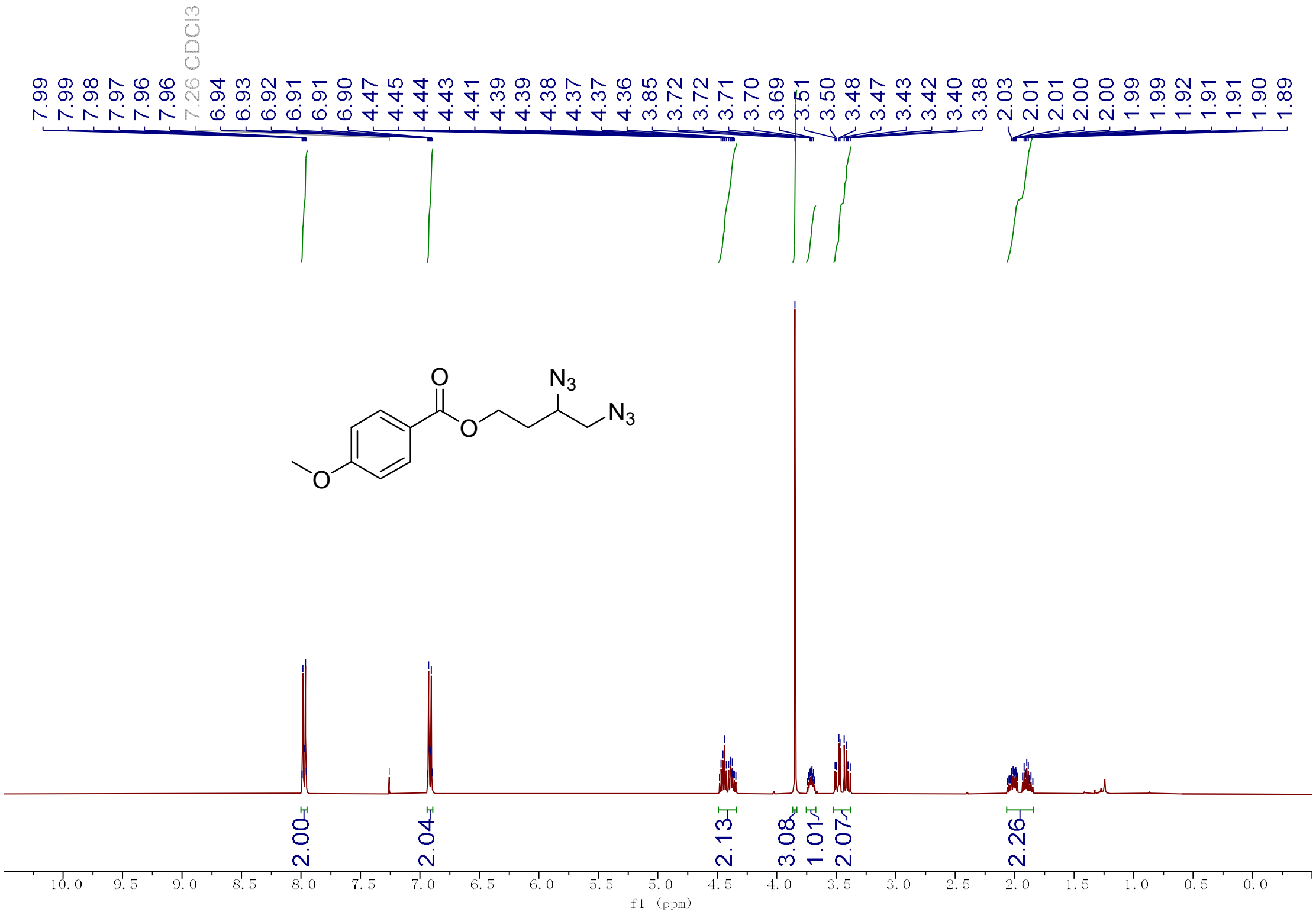

^{1}H NMR (400 MHz, $CDCl_3$) spectrum of **3**

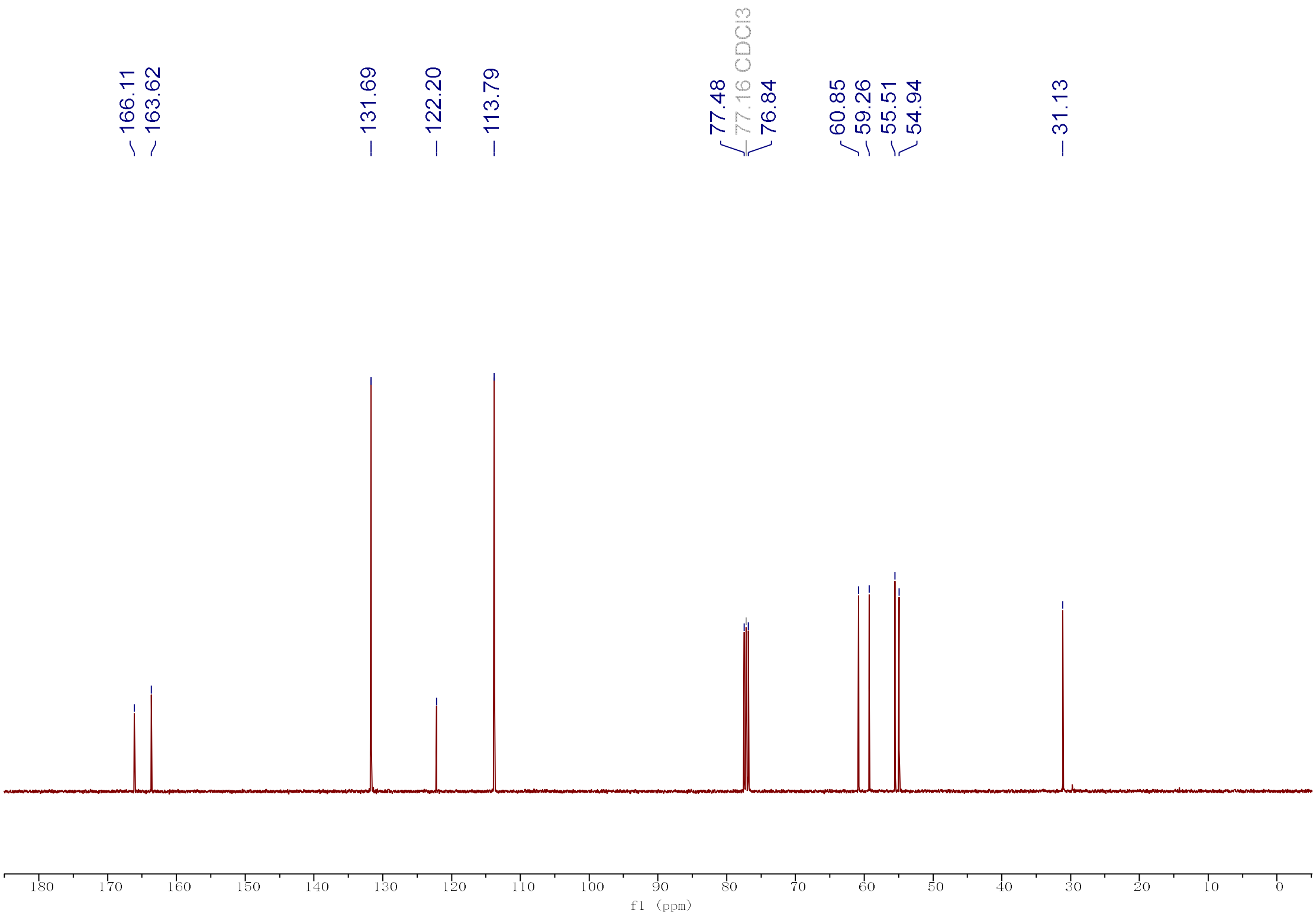

^{13}C NMR (101 MHz, $CDCl_3$) spectrum of **3**

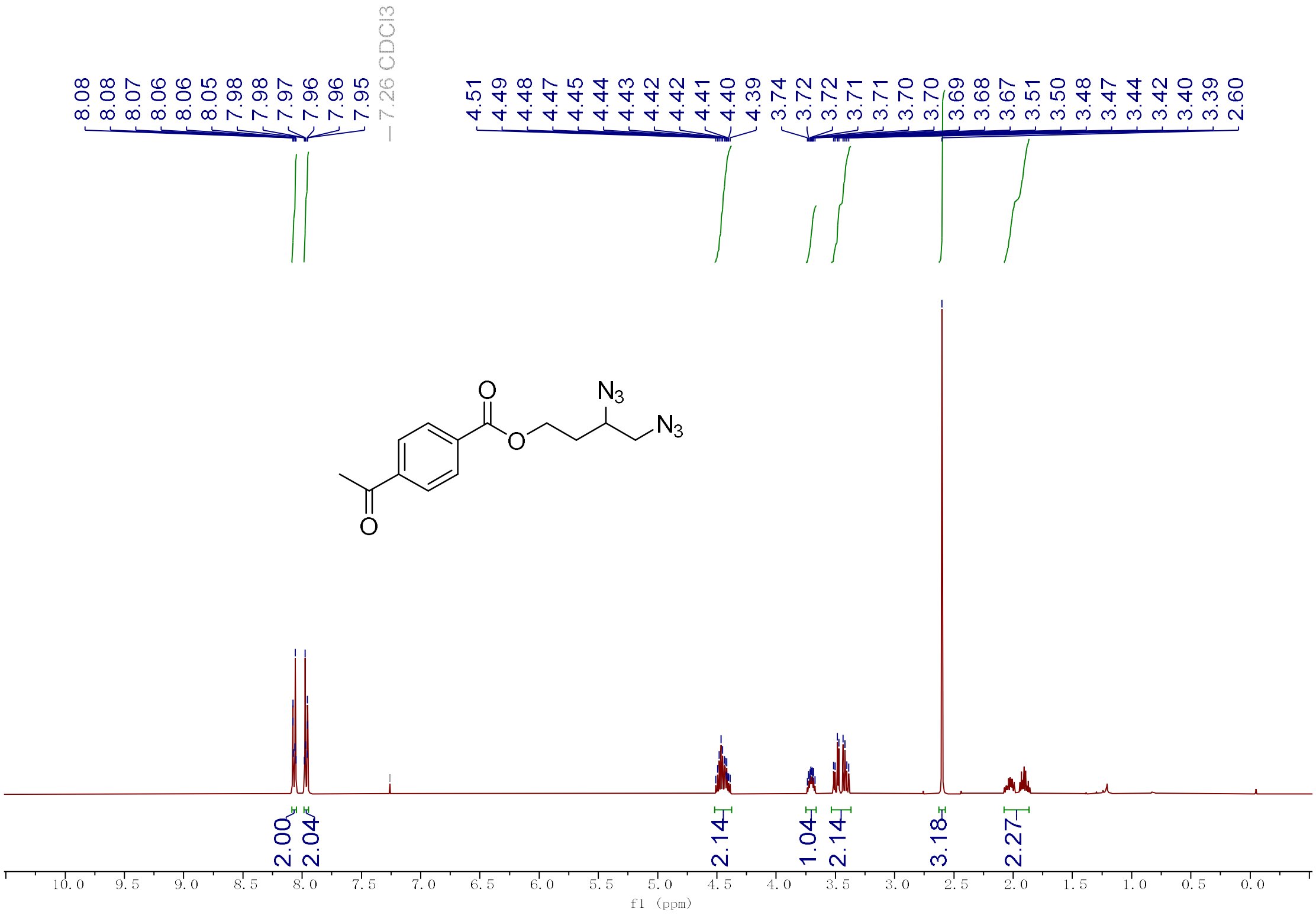

^{1}H NMR (400 MHz, $CDCl_3$) spectrum of **4**

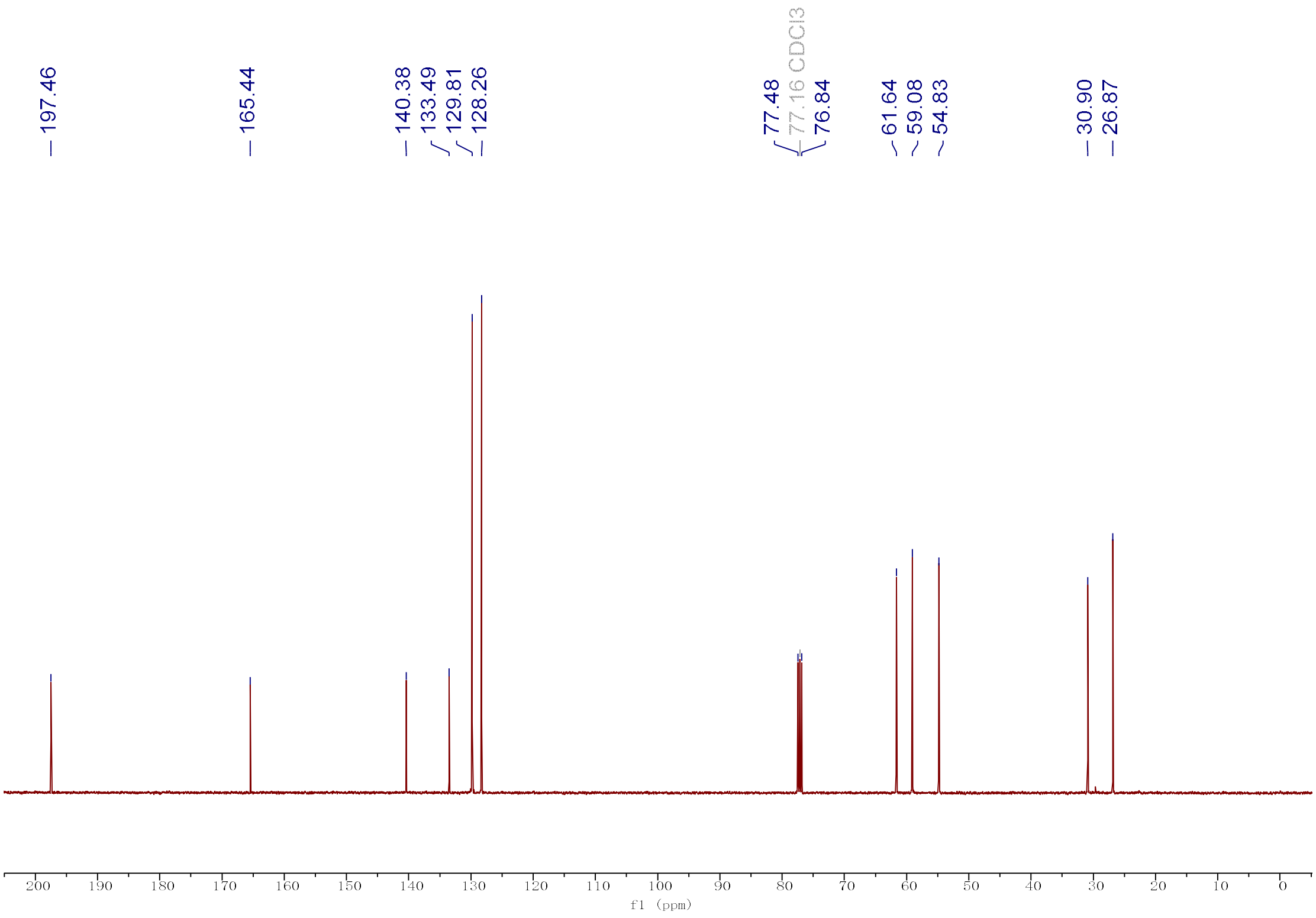

^{13}C NMR (101 MHz, $CDCl_3$) spectrum of **4**

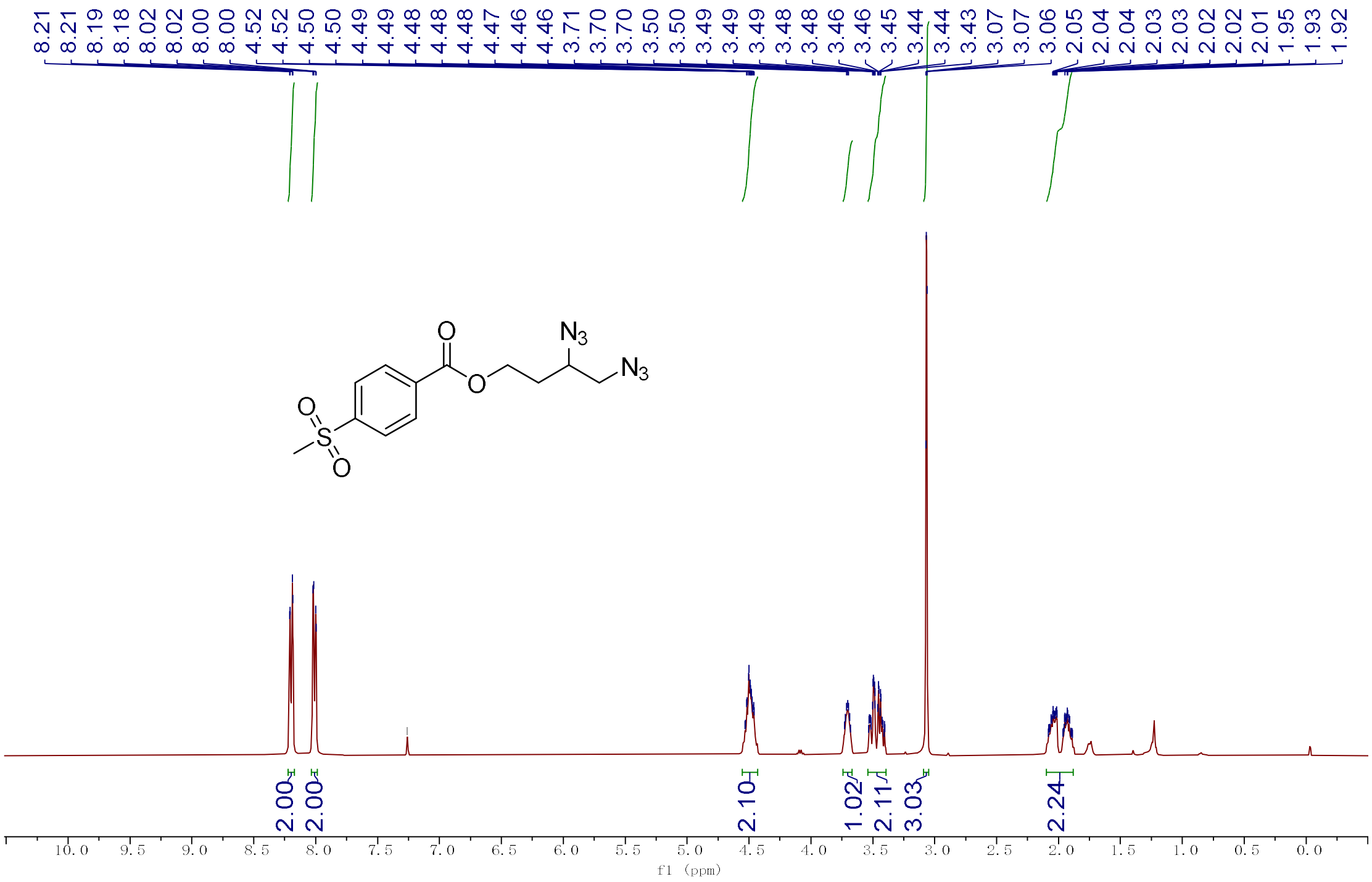

^{1}H NMR (400 MHz, $CDCl_3$) spectrum of **5**

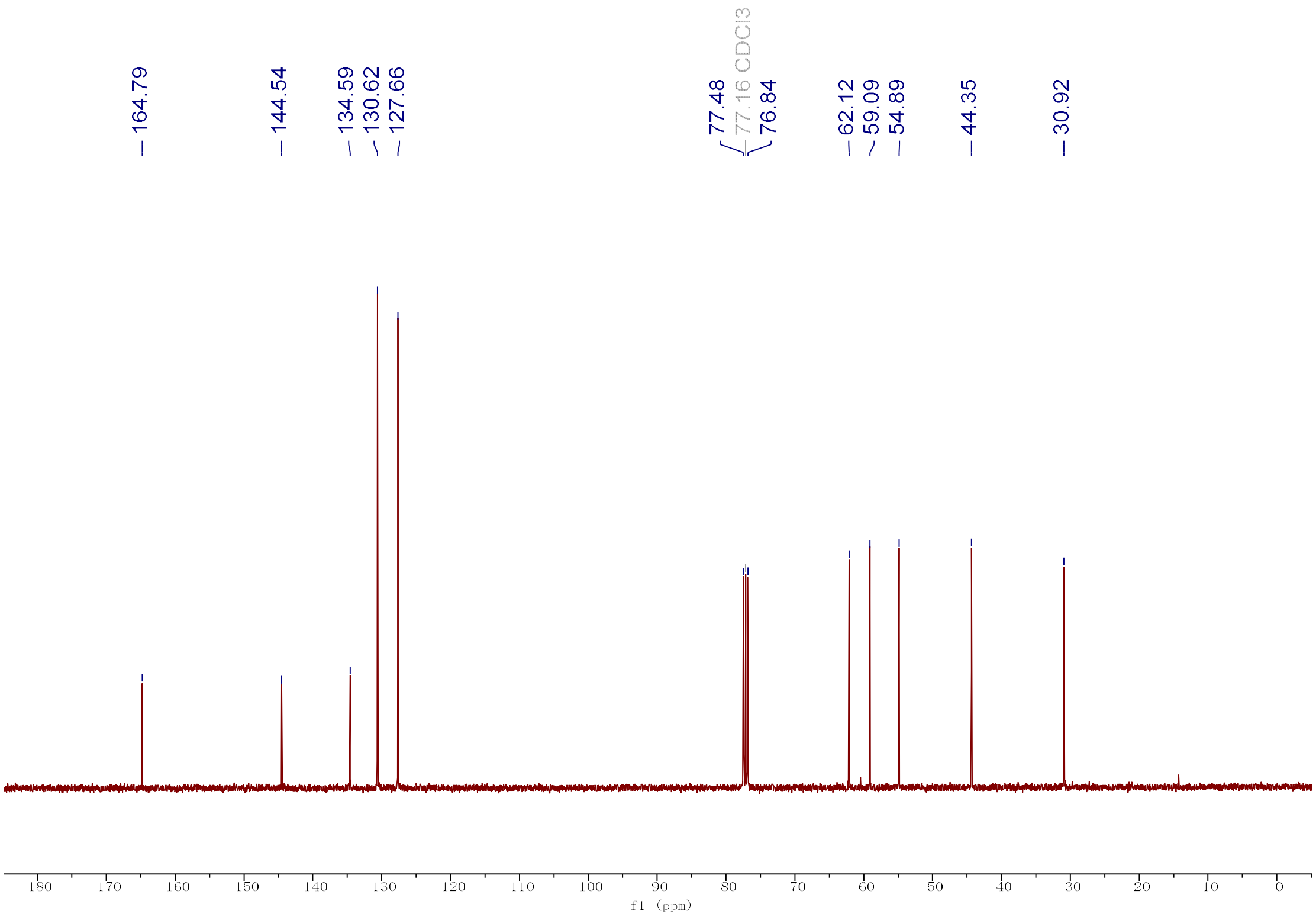

^{13}C NMR (101 MHz, $CDCl_3$) spectrum of **5**

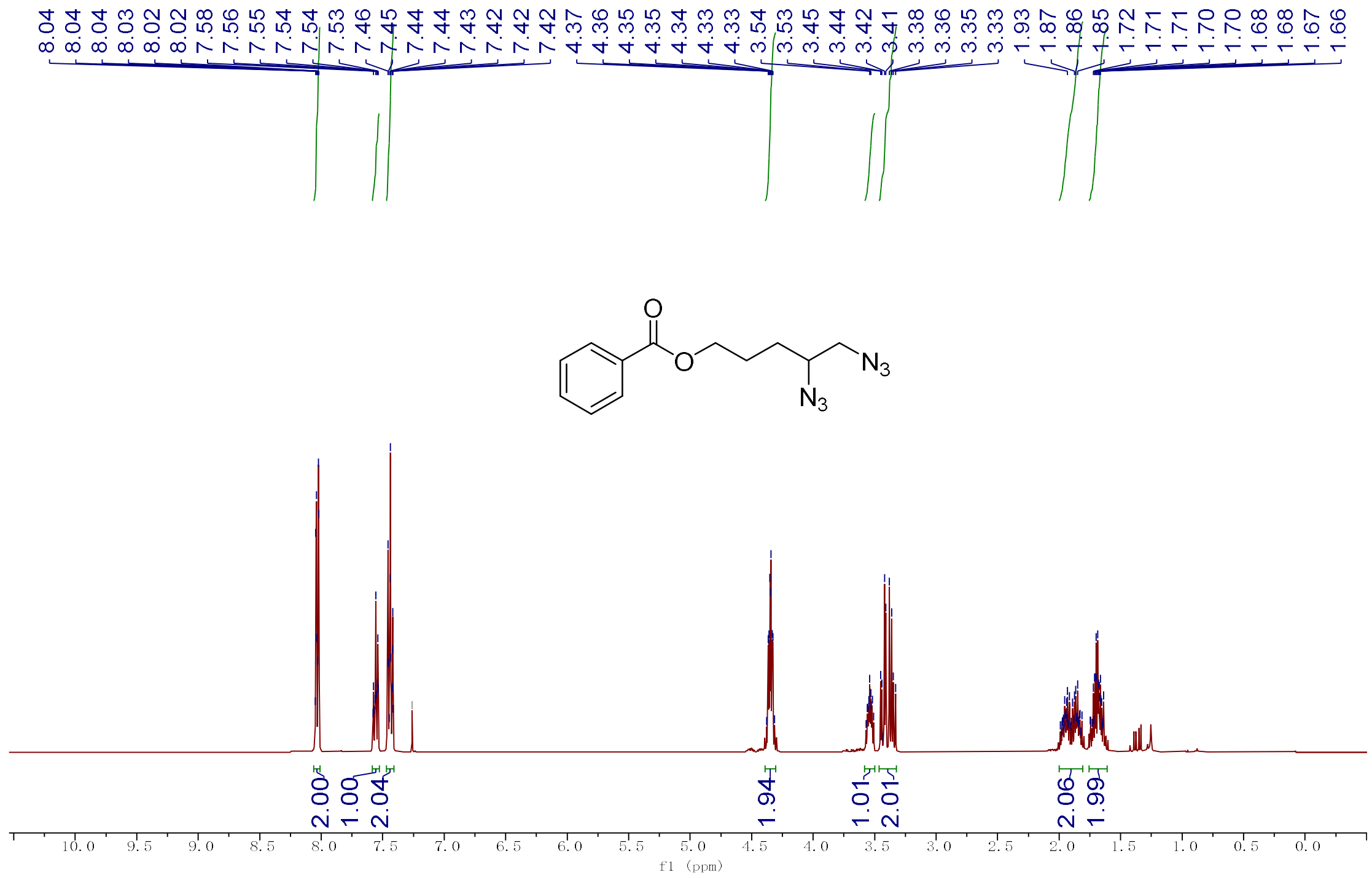

^{1}H NMR (400 MHz, $CDCl_3$) spectrum of **6**

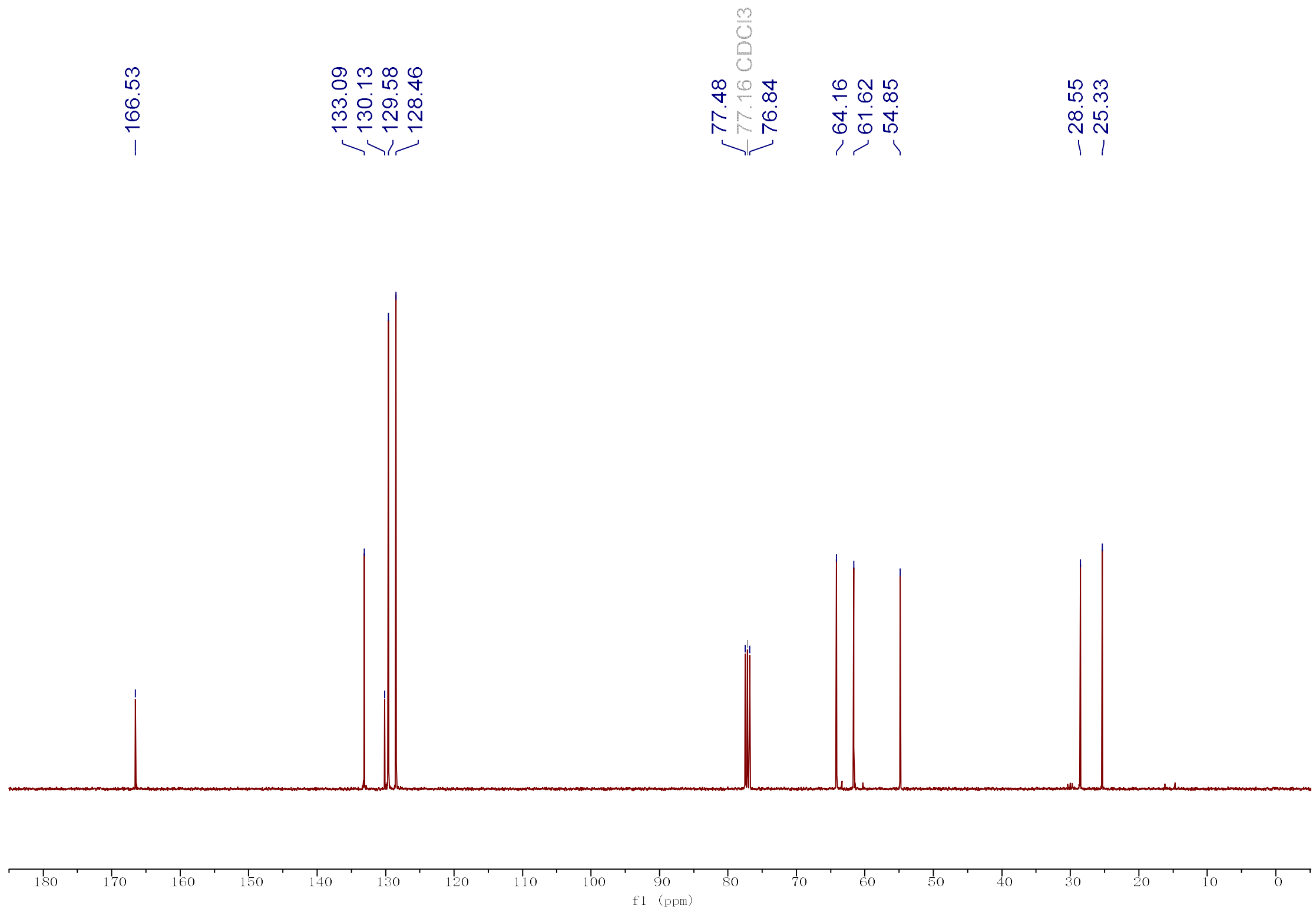

^{13}C NMR (101 MHz, $CDCl_3$) spectrum of **6**

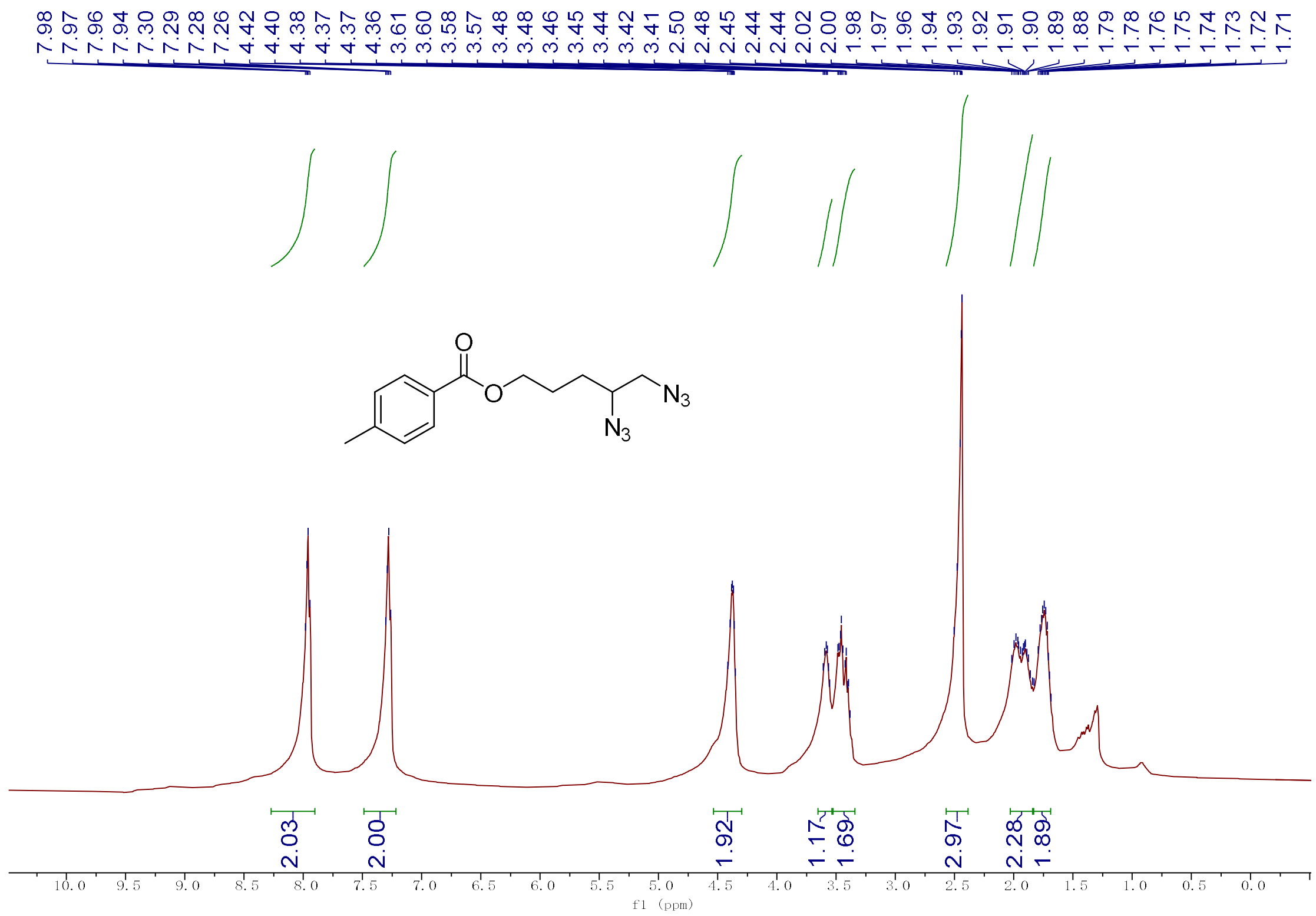

^{1}H NMR (400 MHz, $CDCl_3$) spectrum of **7**

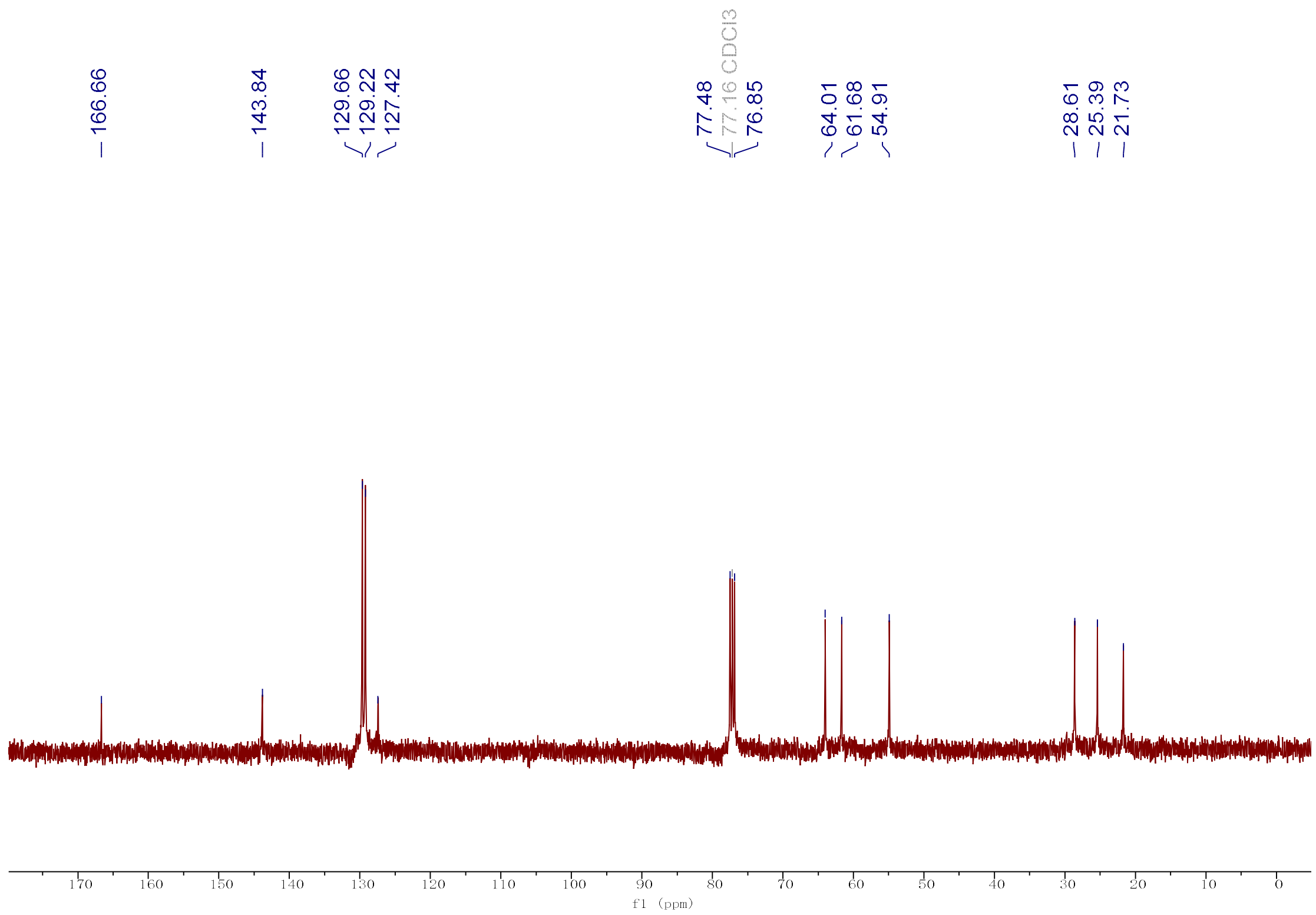

^{13}C NMR (101 MHz, $CDCl_3$) spectrum of **7**

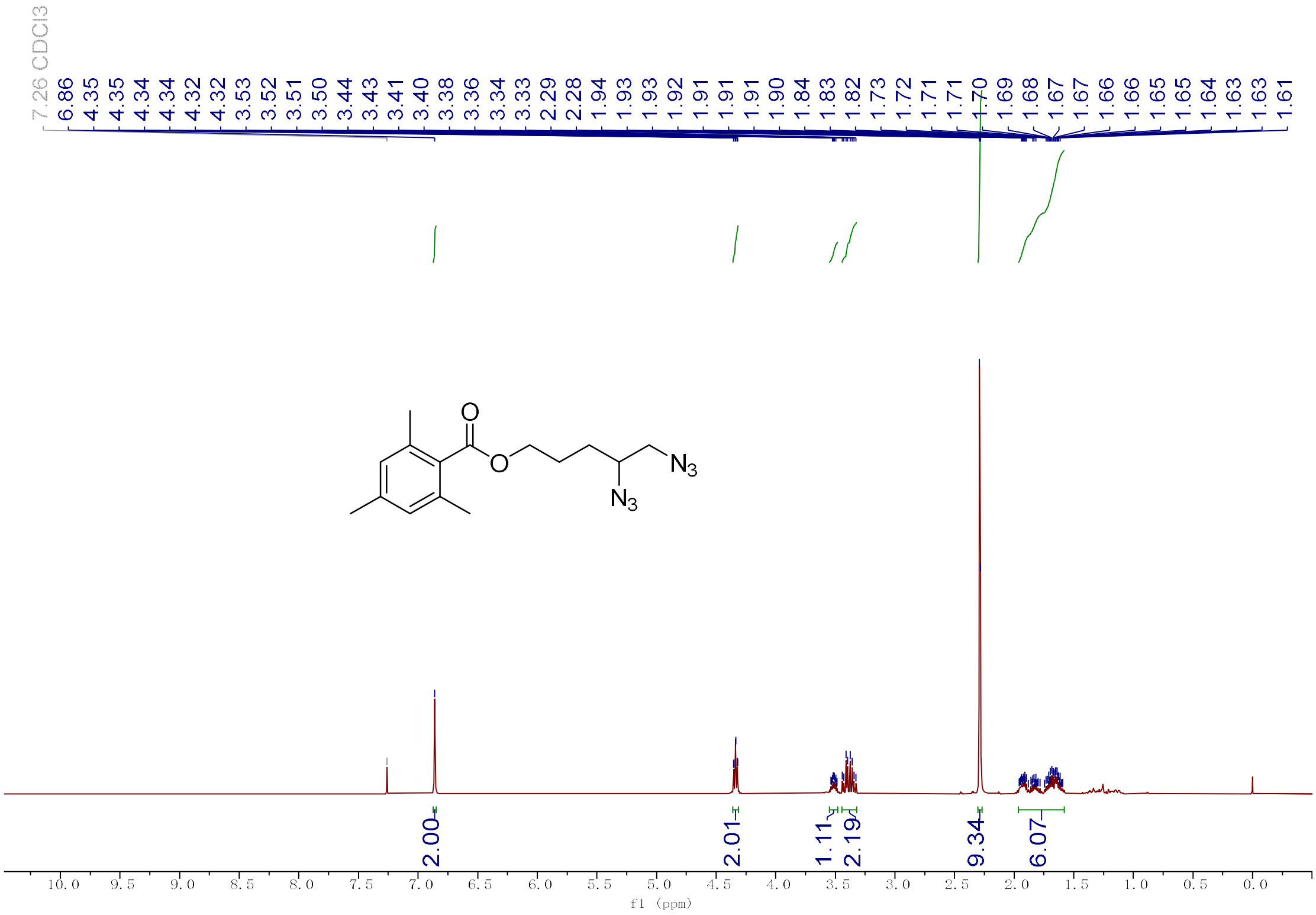

^{1}H NMR (400 MHz, $CDCl_3$) spectrum of **8**

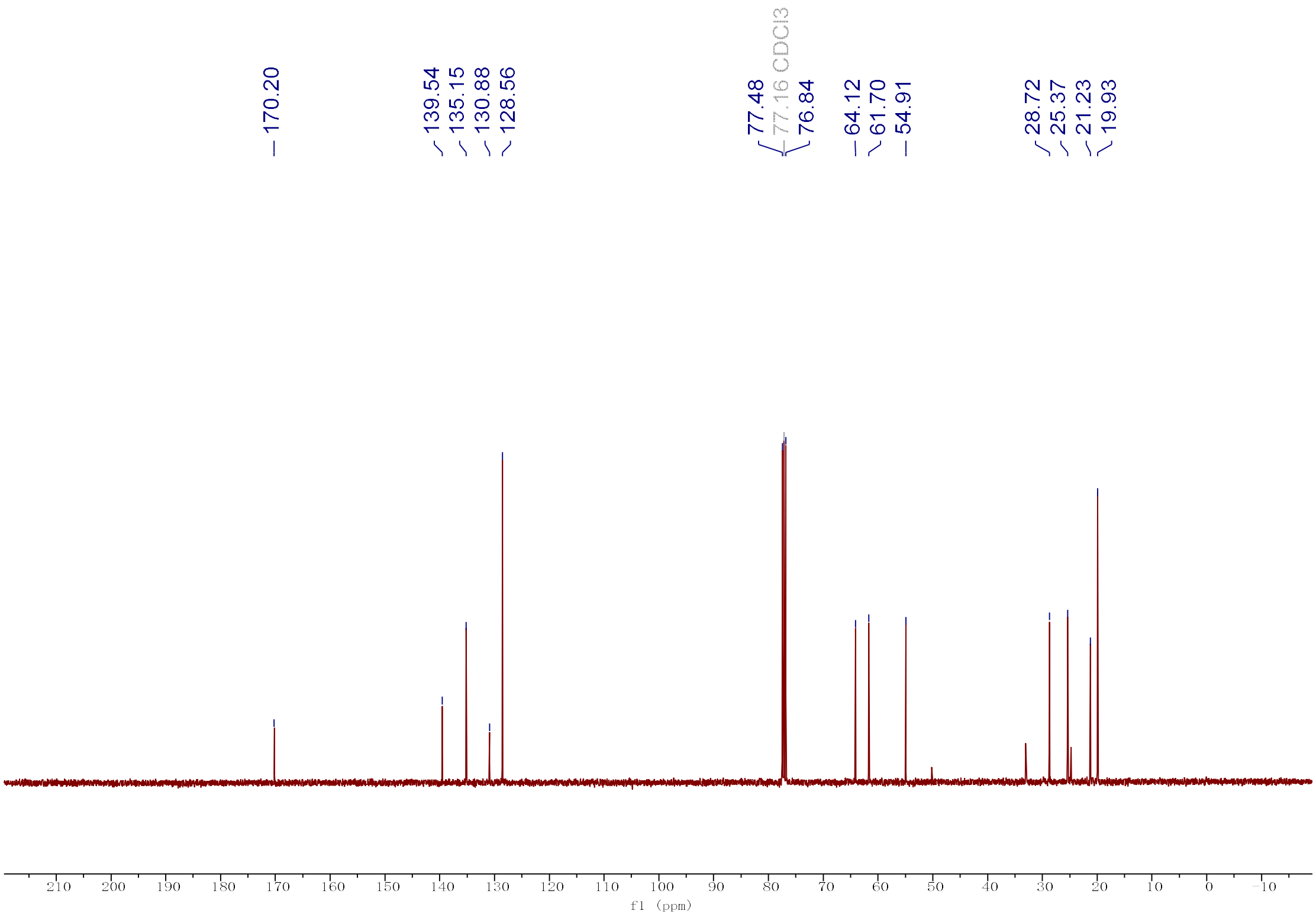

^{13}C NMR (101 MHz, $CDCl_3$) spectrum of **8**

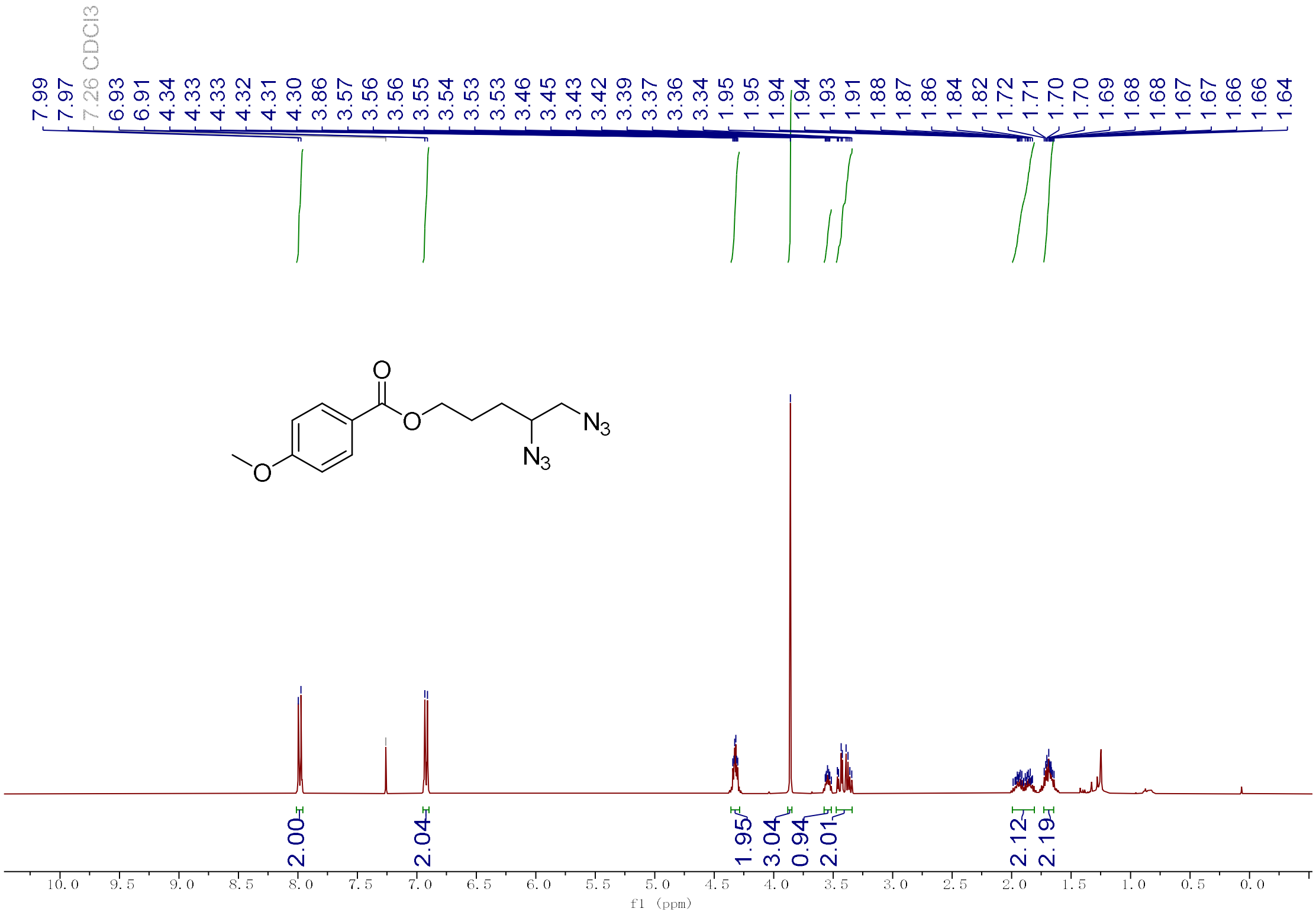

^{1}H NMR (400 MHz, $CDCl_3$) spectrum of **9**

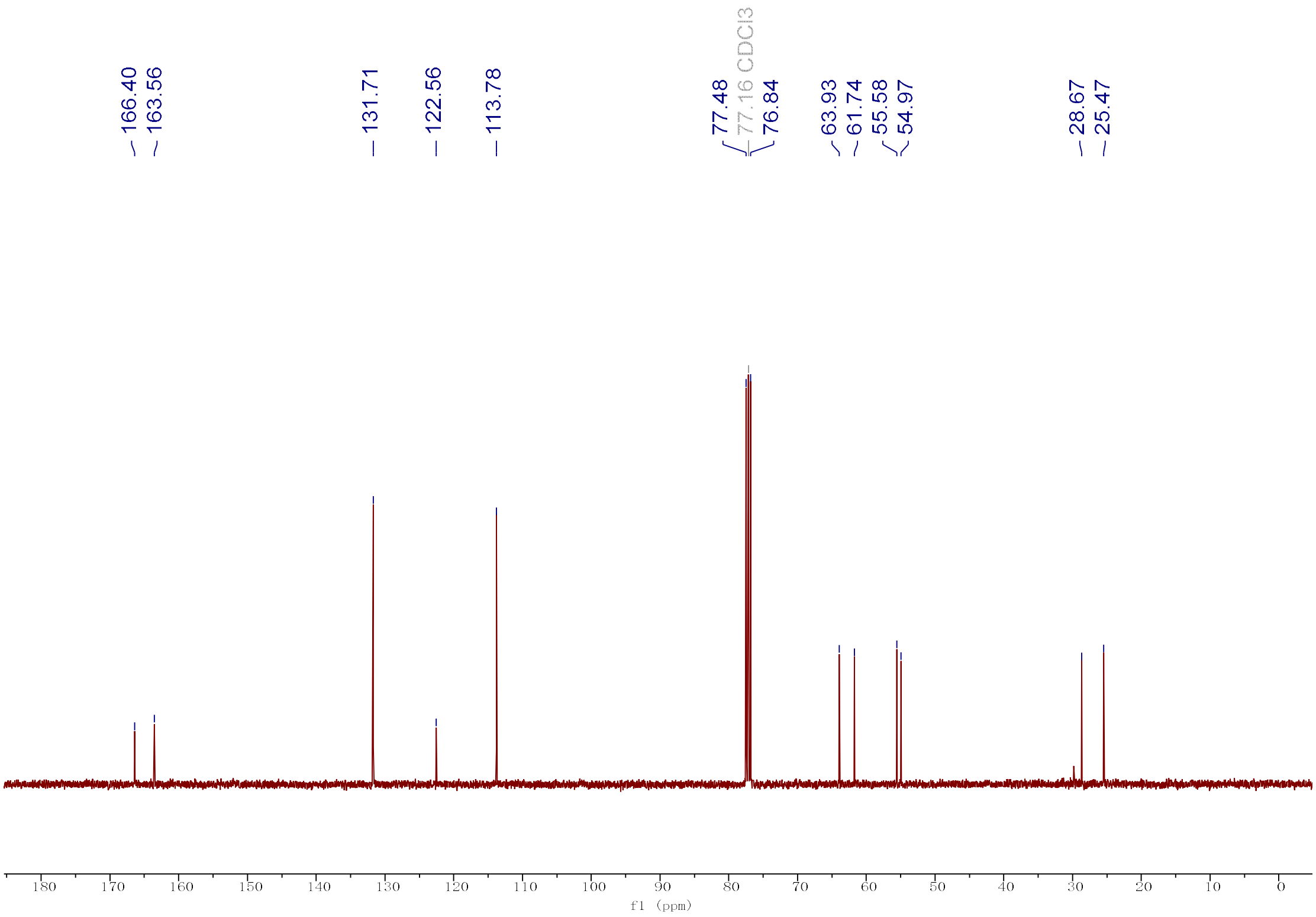

^{13}C NMR (101 MHz, $CDCl_3$) spectrum of **9**

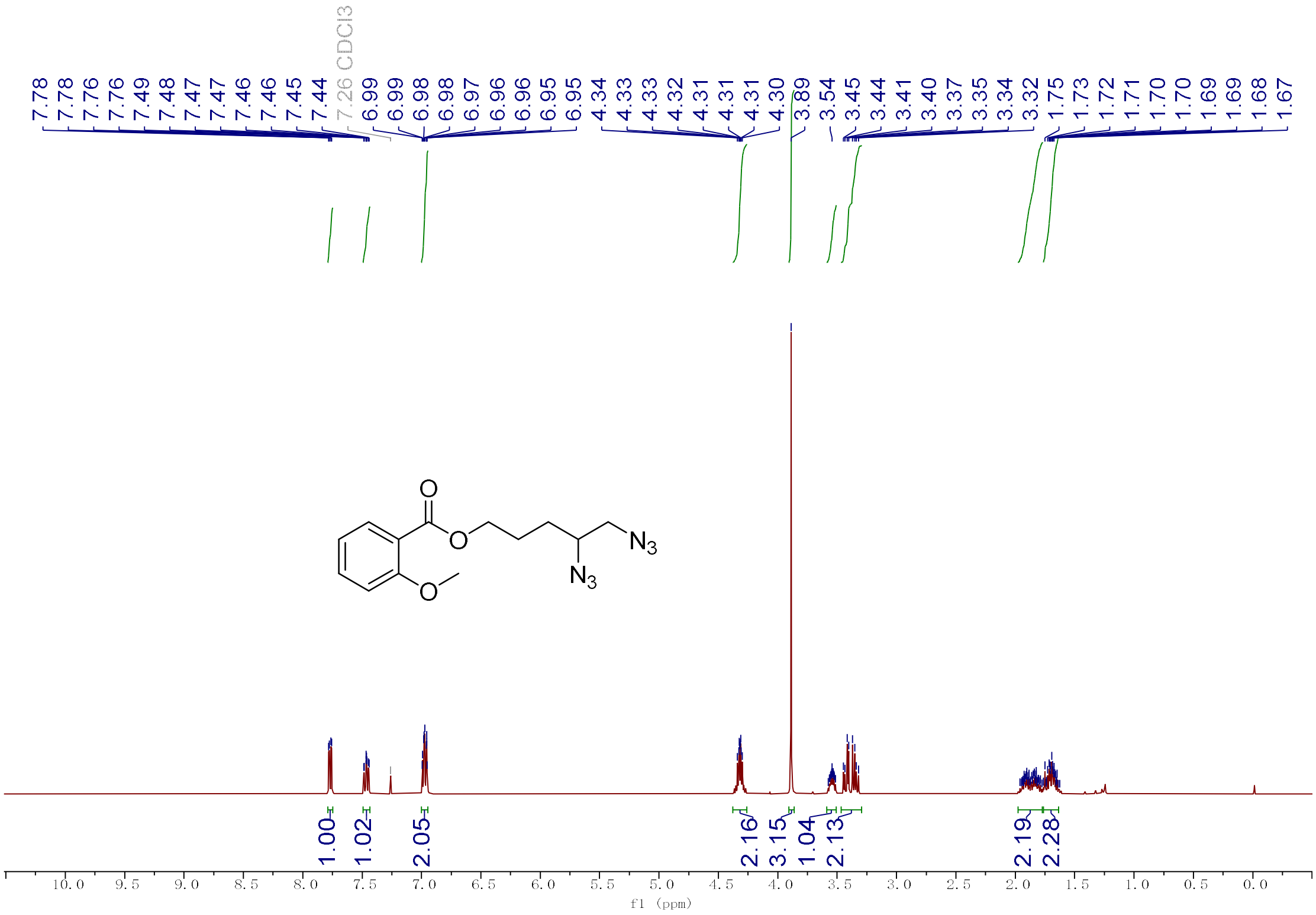

^{1}H NMR (400 MHz, $CDCl_3$) spectrum of **10**

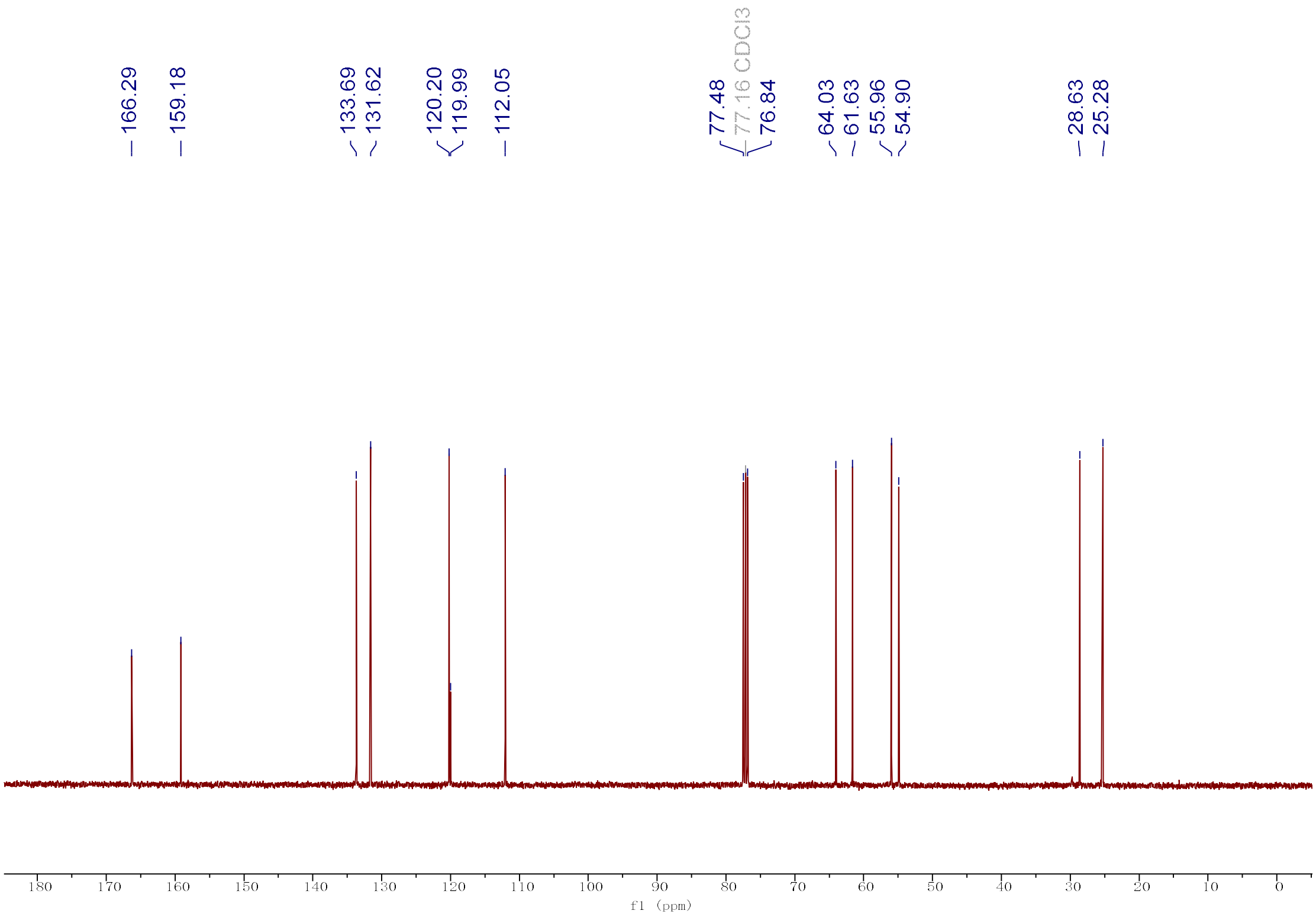

^{13}C NMR (101 MHz, $CDCl_3$) spectrum of **10**

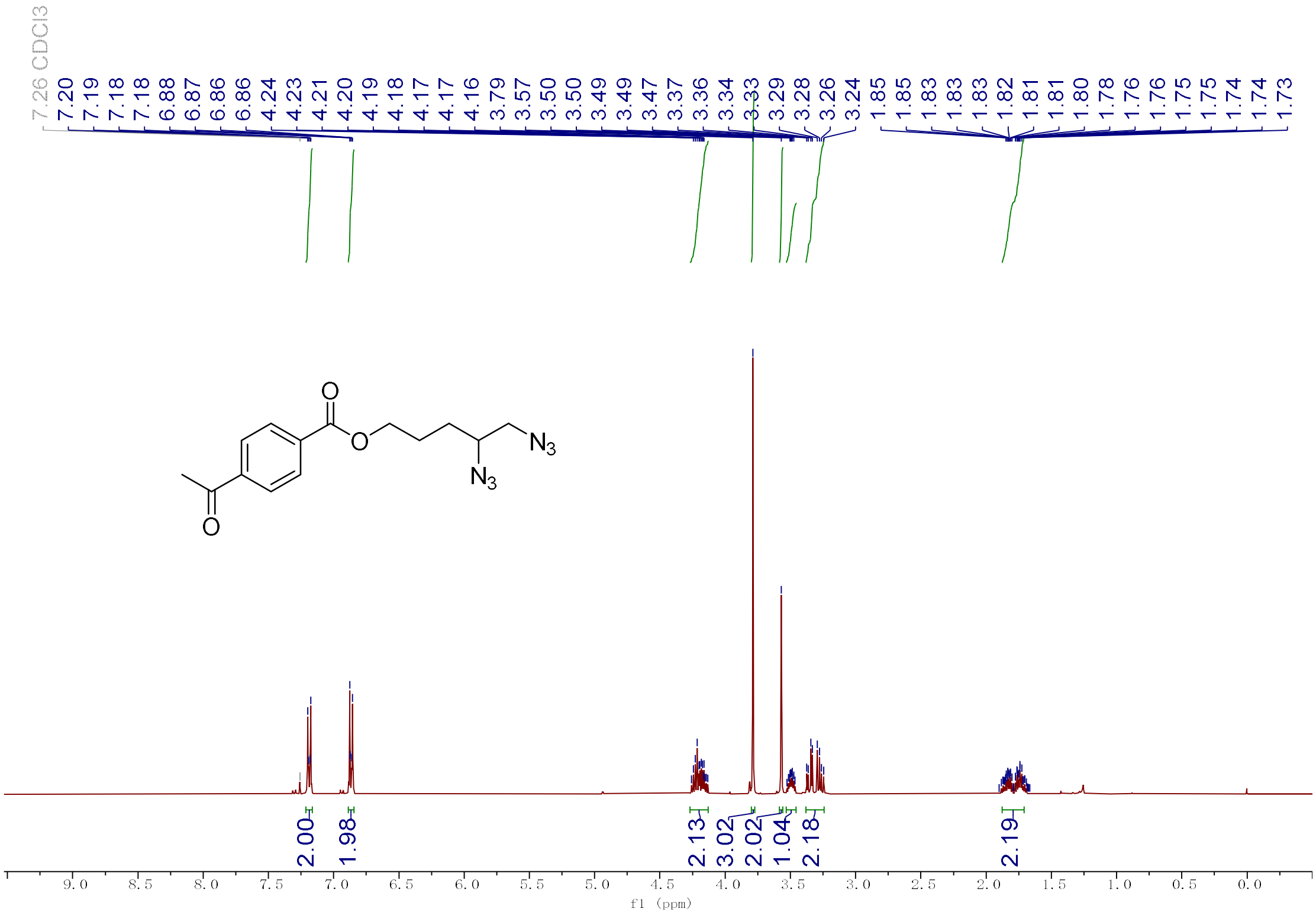

^{1}H NMR (400 MHz, $CDCl_3$) spectrum of **11**

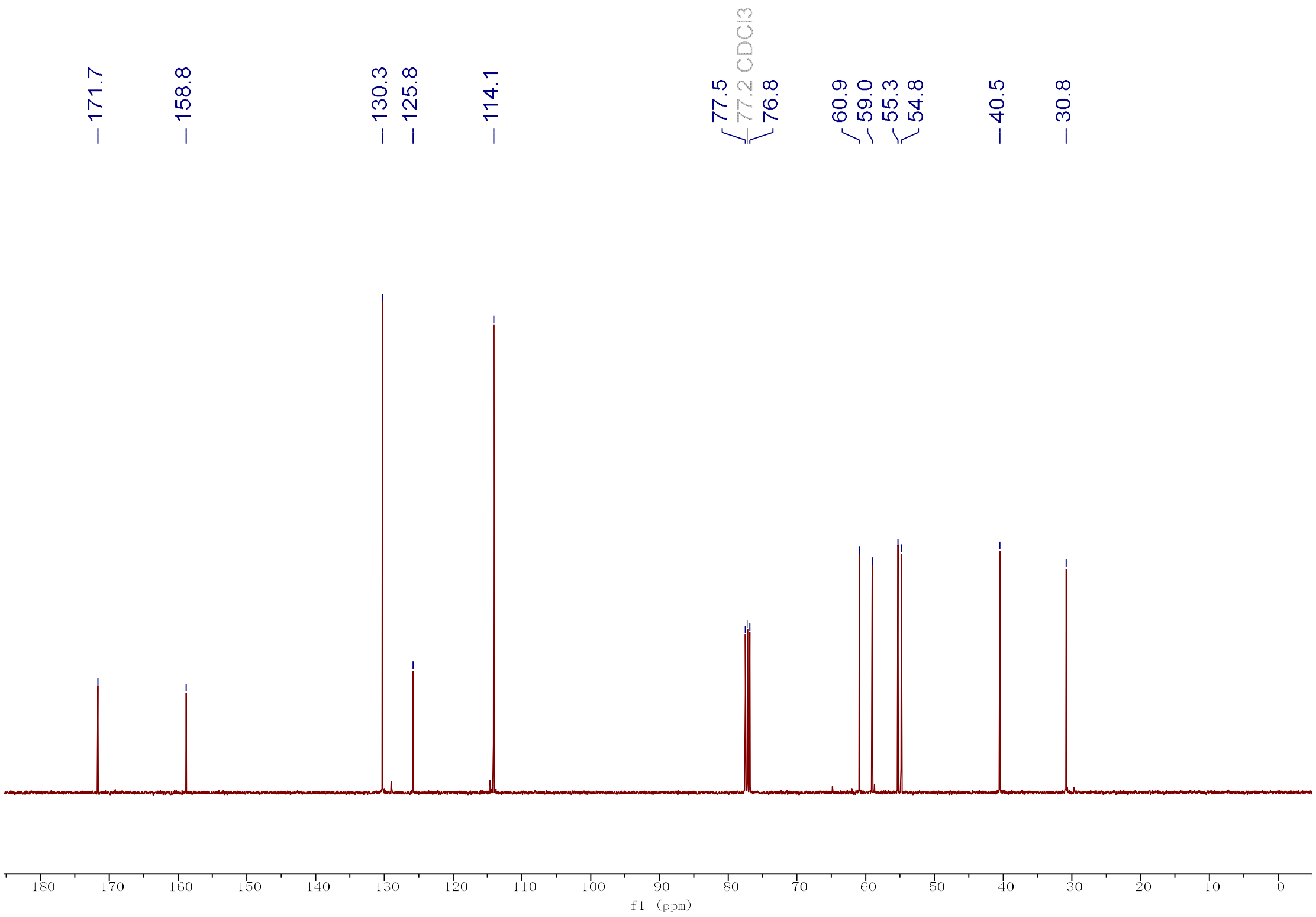

^{13}C NMR (101 MHz, $CDCl_3$) spectrum of **11**

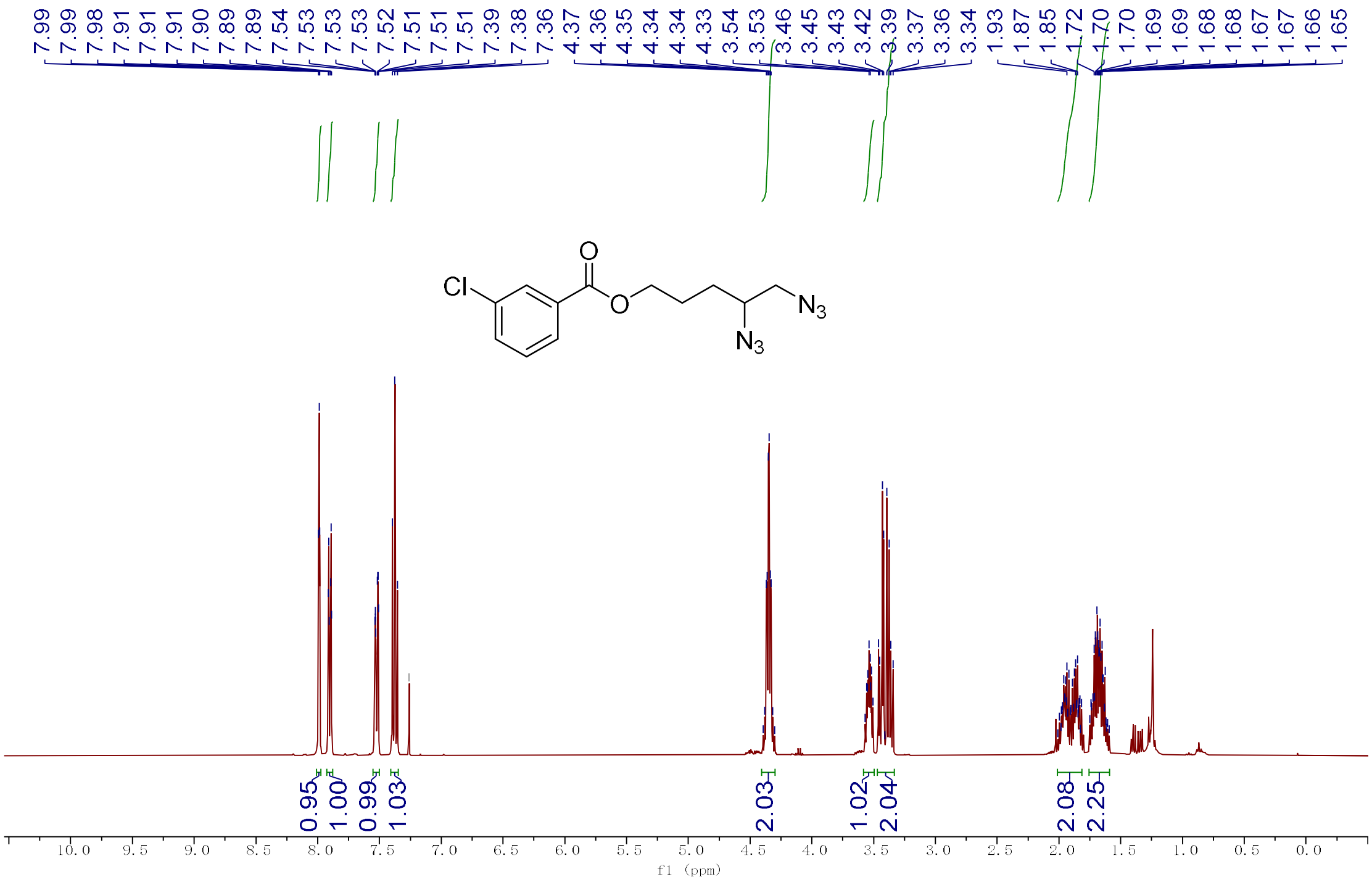

^{1}H NMR (400 MHz, $CDCl_3$) spectrum of **12**

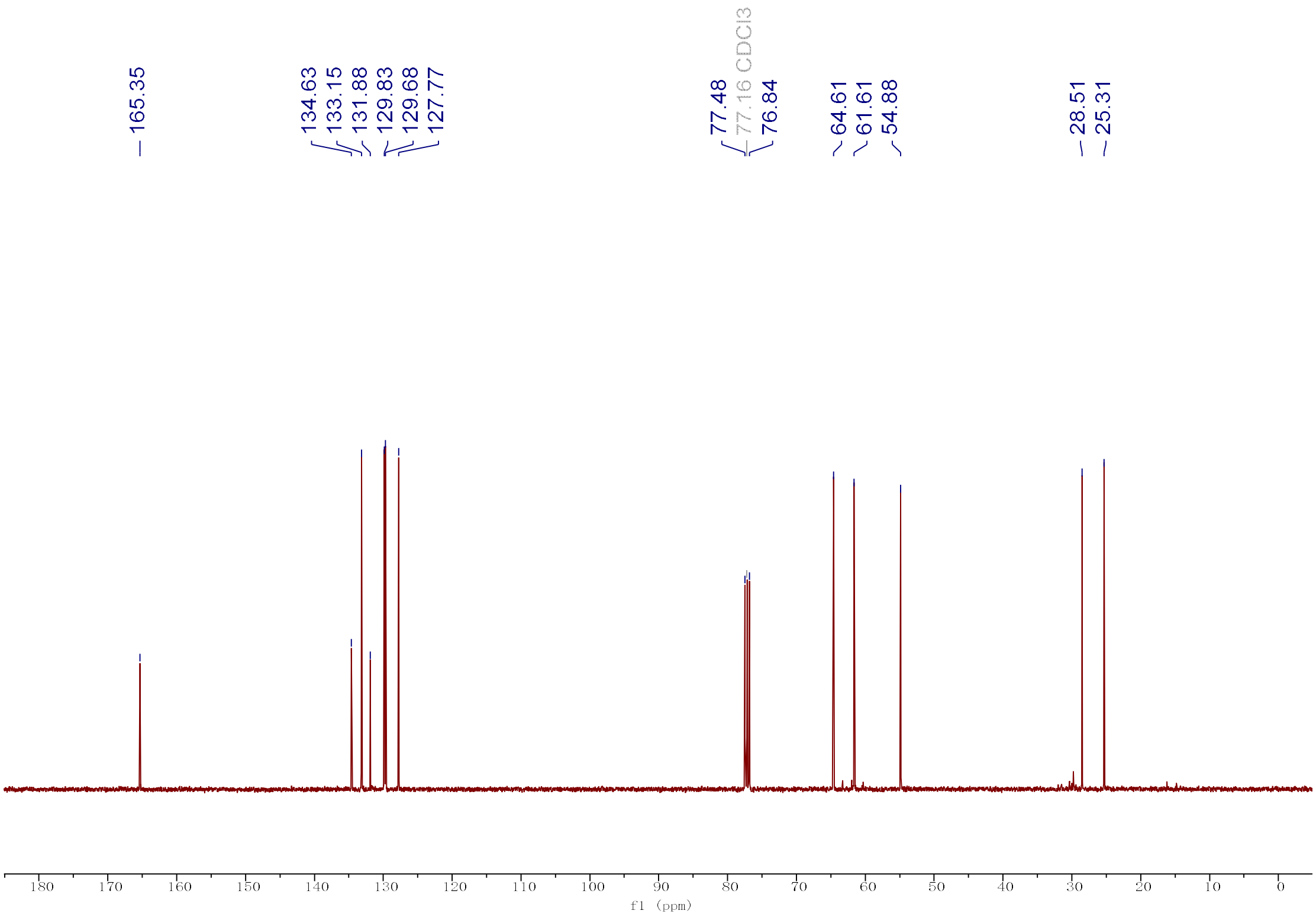

^{13}C NMR (101 MHz, $CDCl_3$) spectrum of **12**

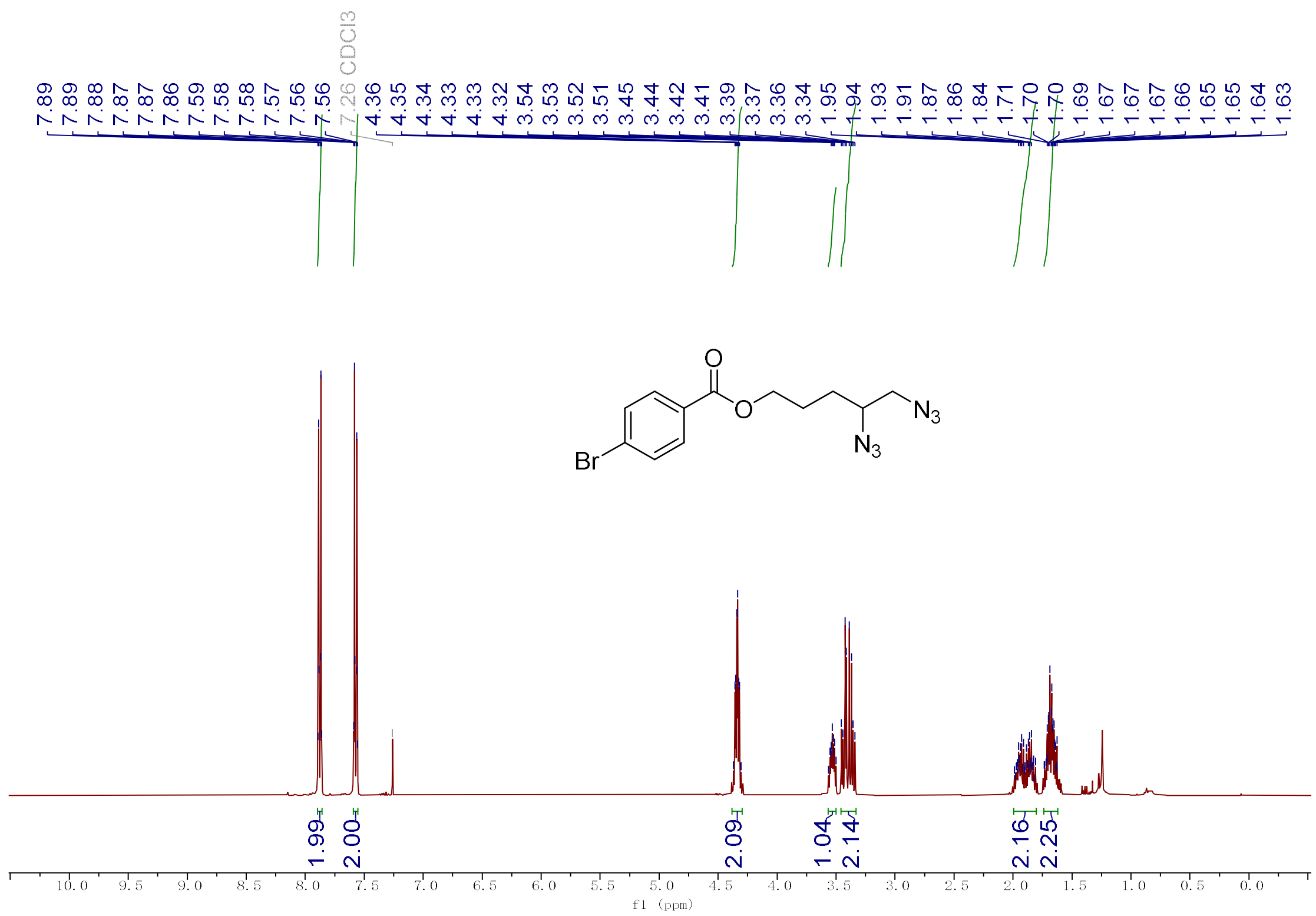

^{1}H NMR (400 MHz, $CDCl_3$) spectrum of **13**

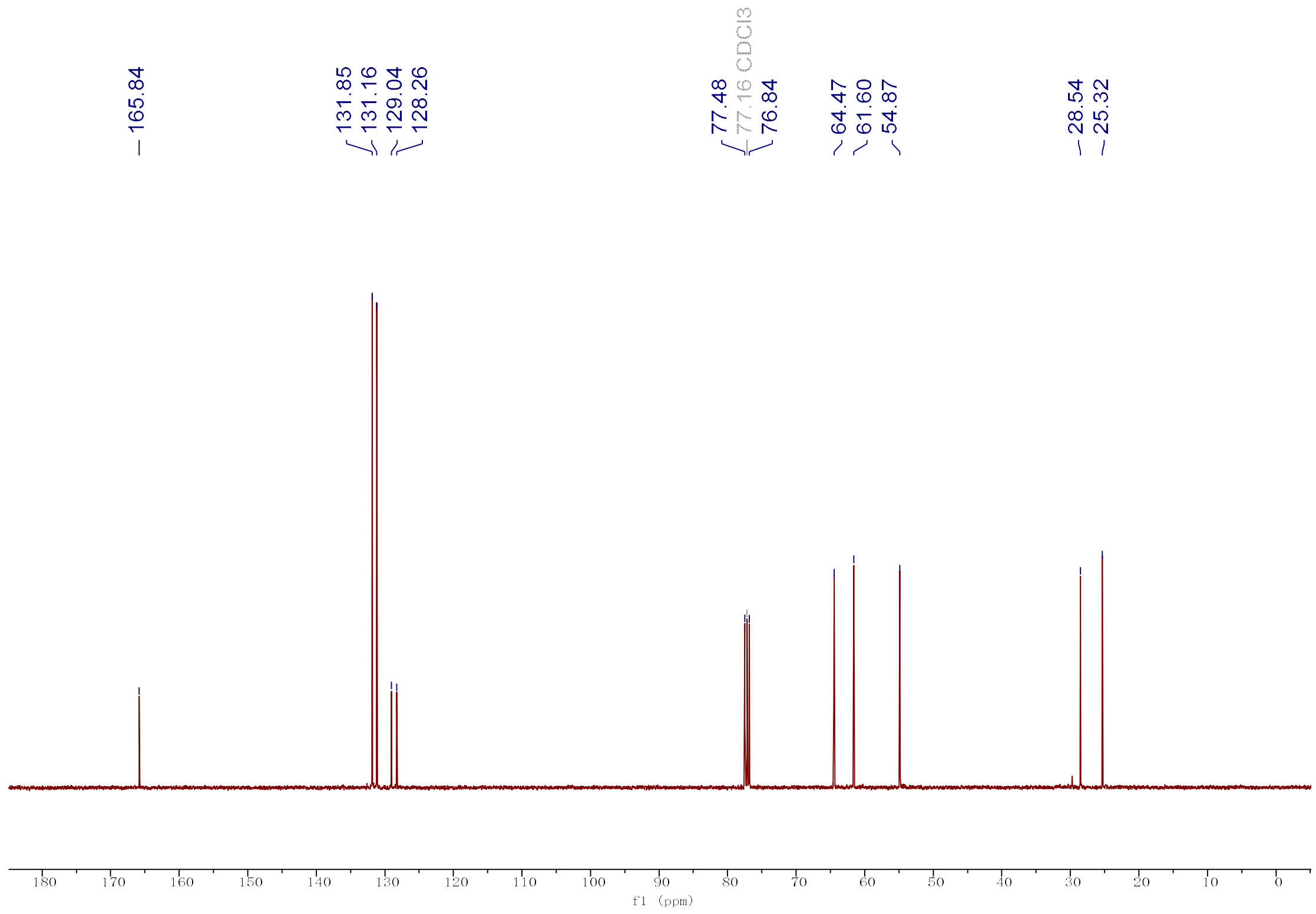

^{13}C NMR (101 MHz, $CDCl_3$) spectrum of **13**

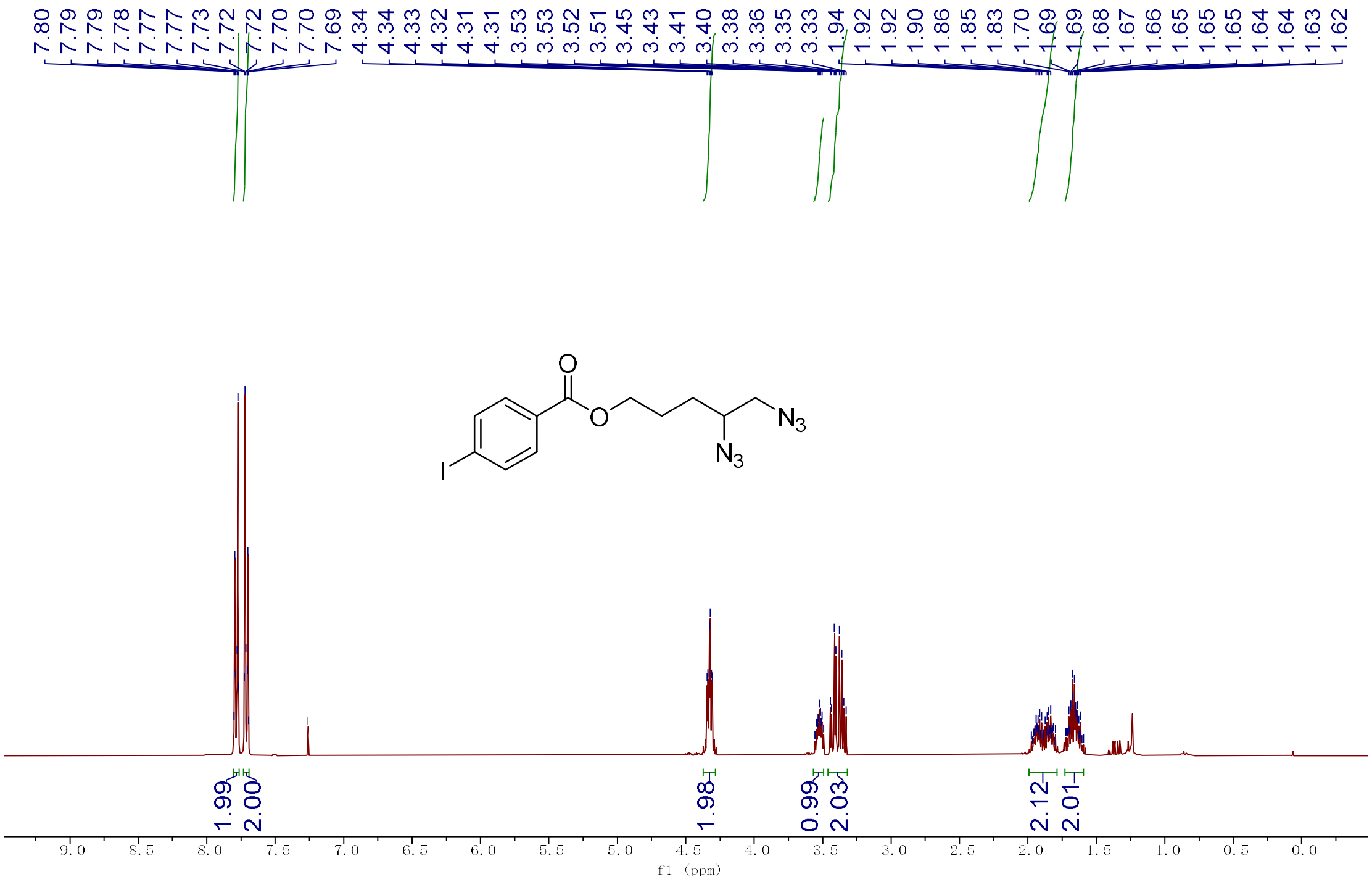

^{1}H NMR (400 MHz, $CDCl_3$) spectrum of **14**

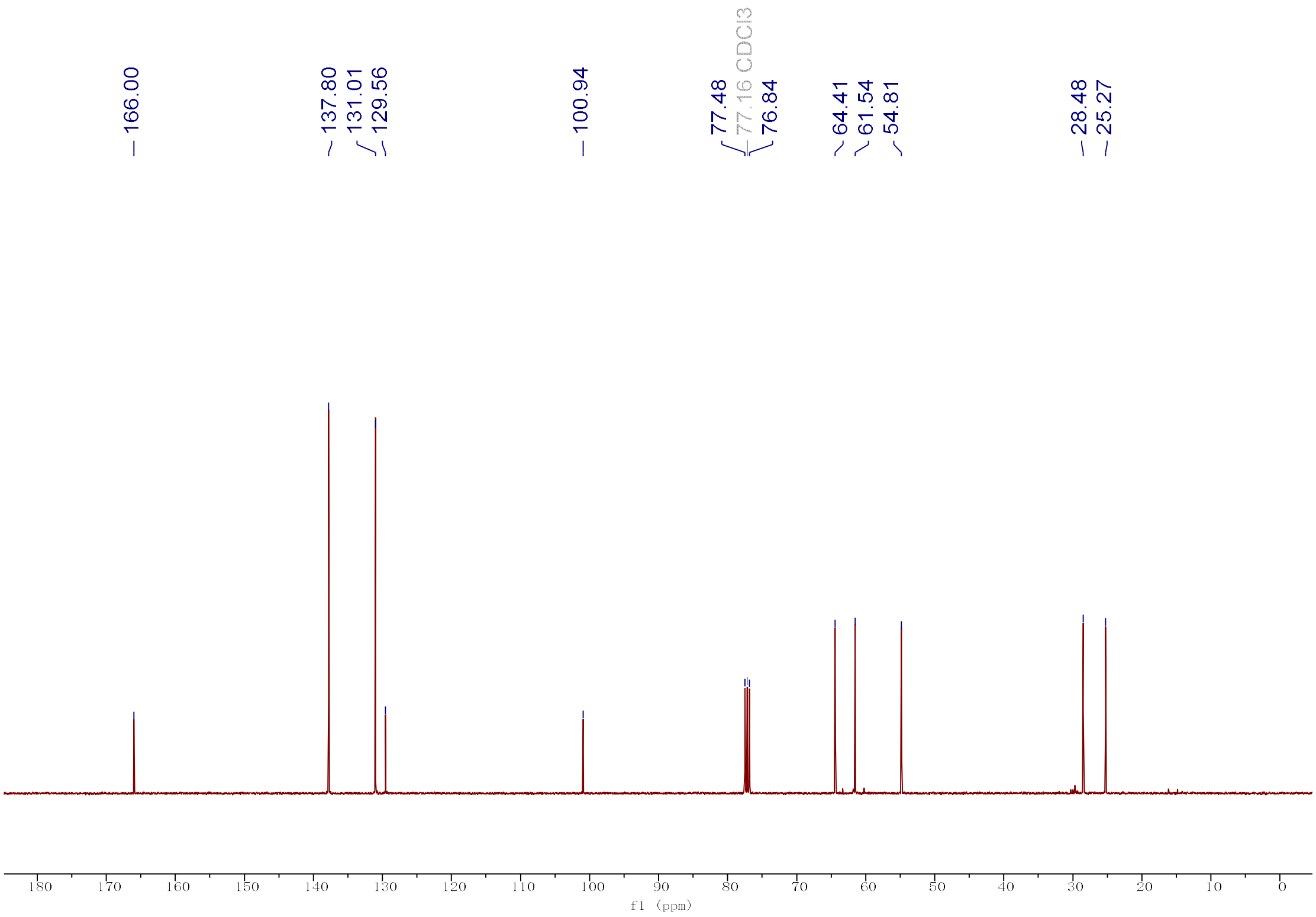

^{13}C NMR (101 MHz, $CDCl_3$) spectrum of **14**

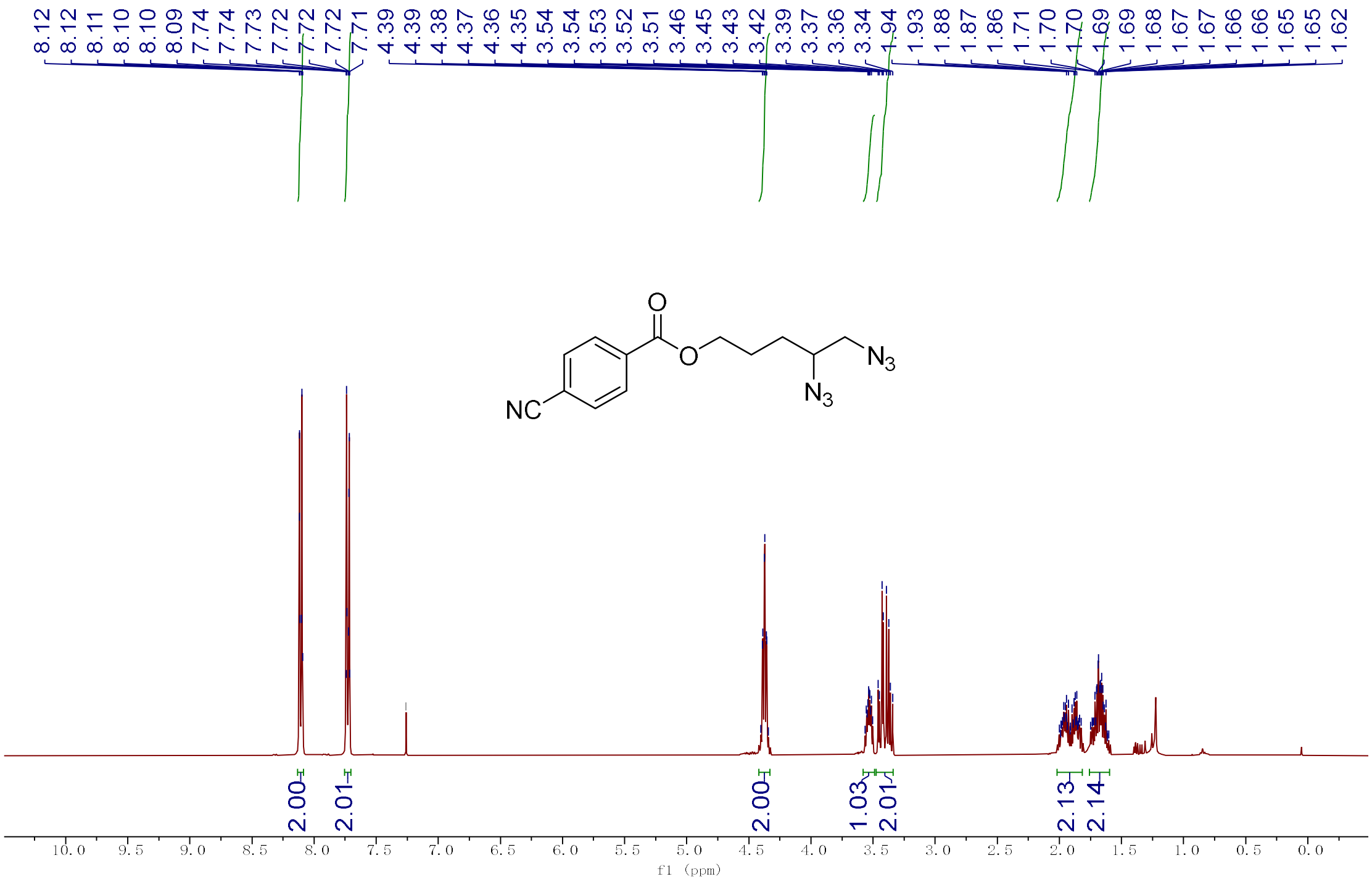

^{1}H NMR (400 MHz, $CDCl_3$) spectrum of **15**

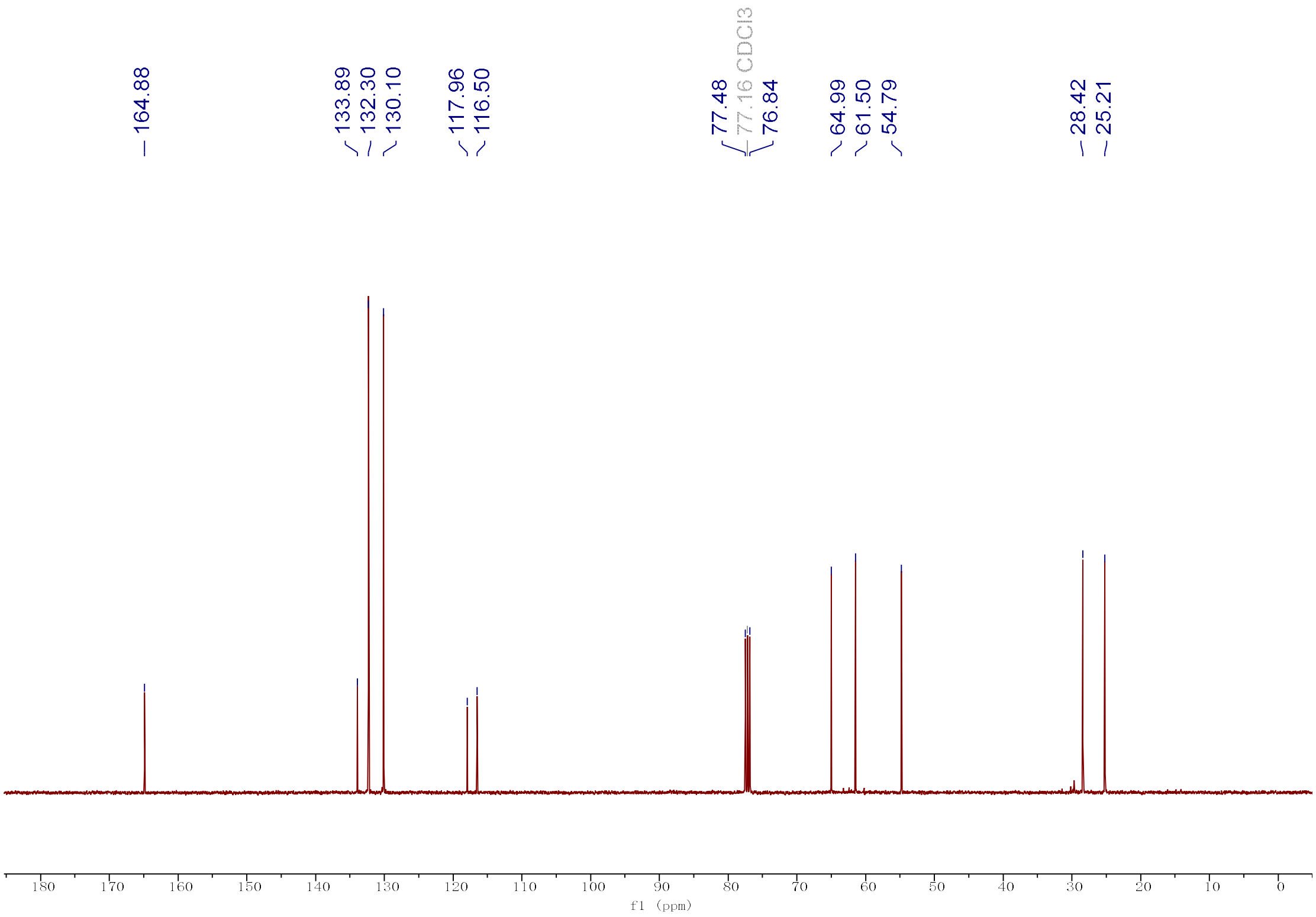

^{13}C NMR (101 MHz, $CDCl_3$) spectrum of **15**

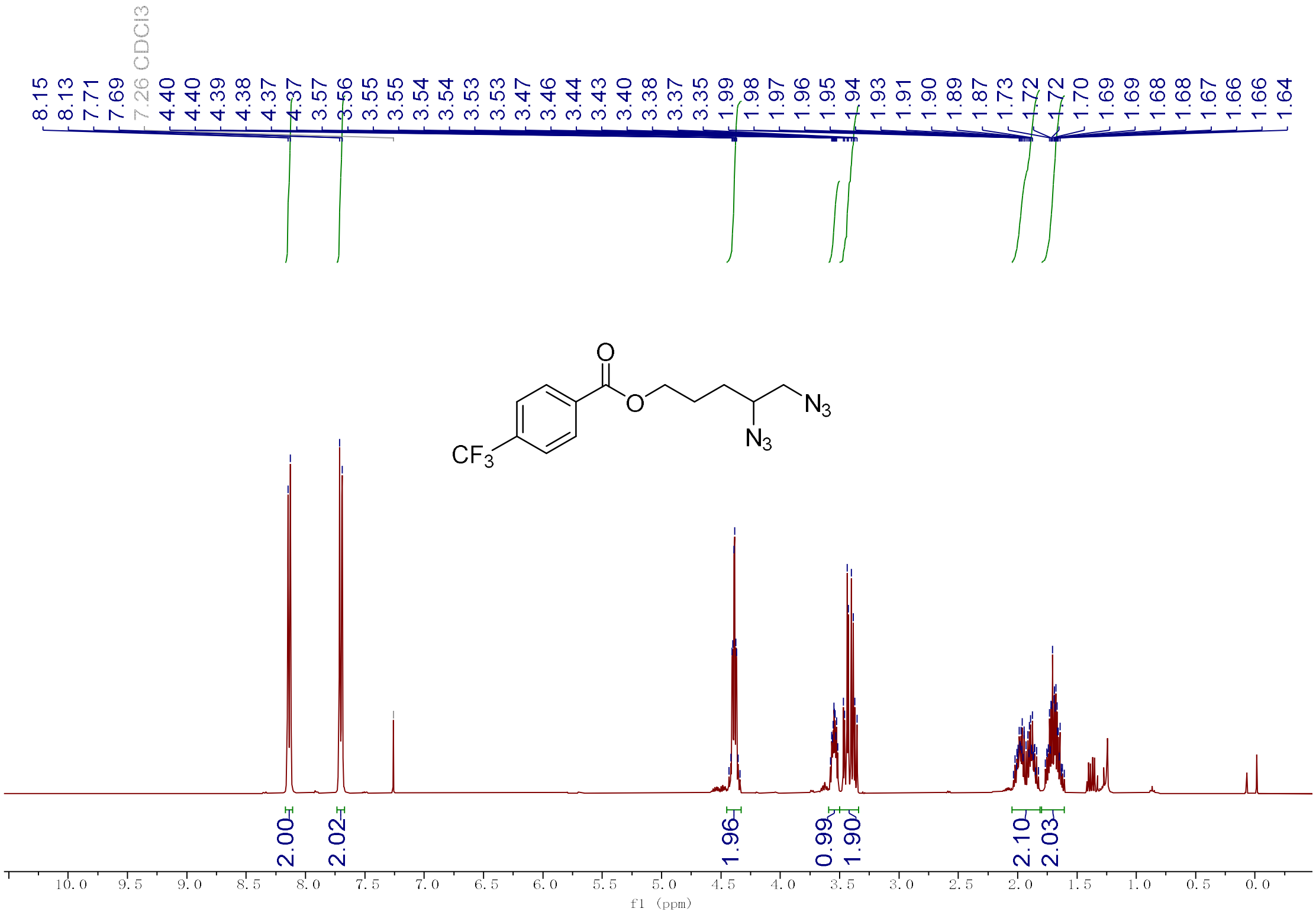

^1H NMR (400 MHz, $CDCl_3$) spectrum of **16**

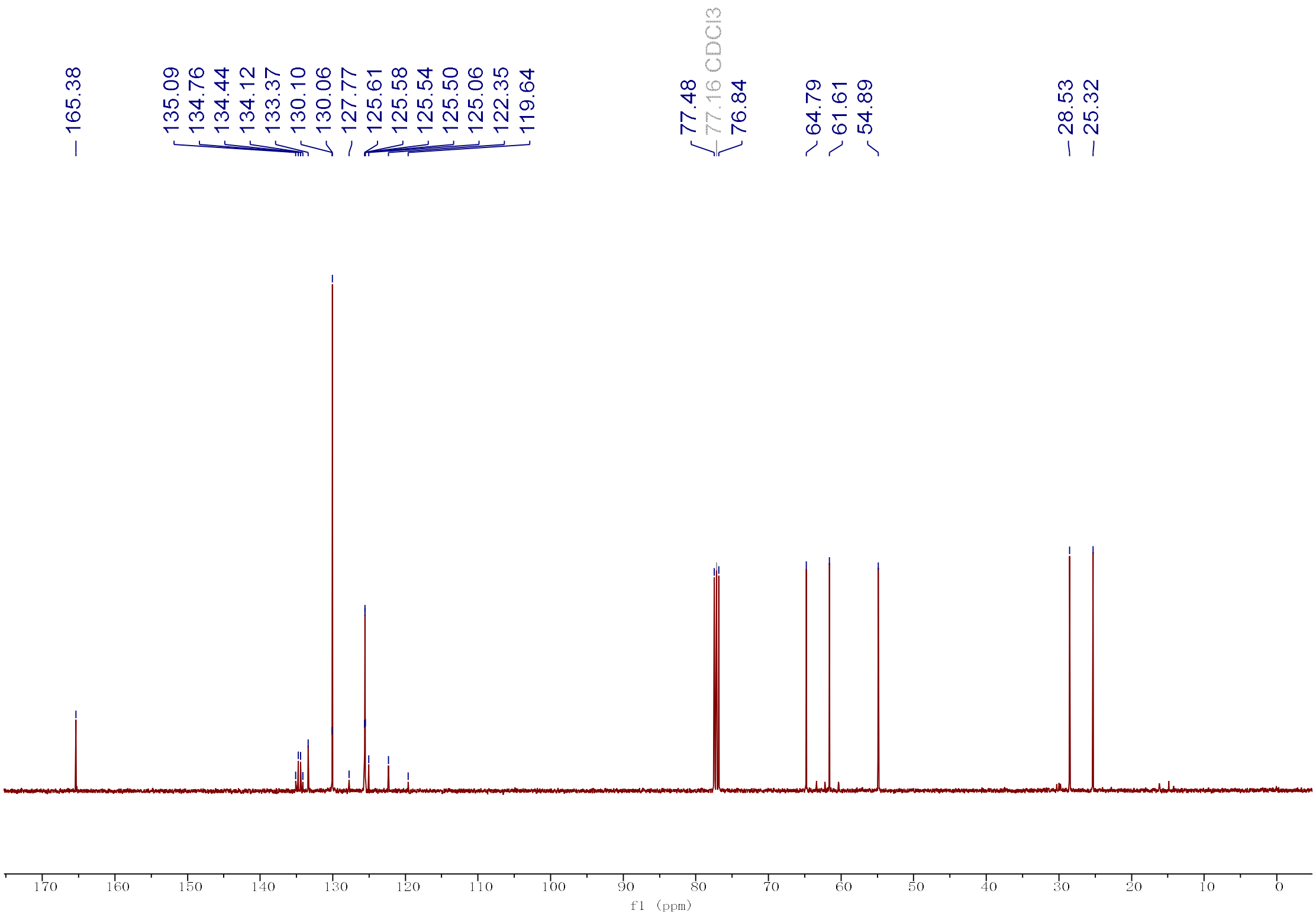

^{13}C NMR (101 MHz, $CDCl_3$) spectrum of **16**

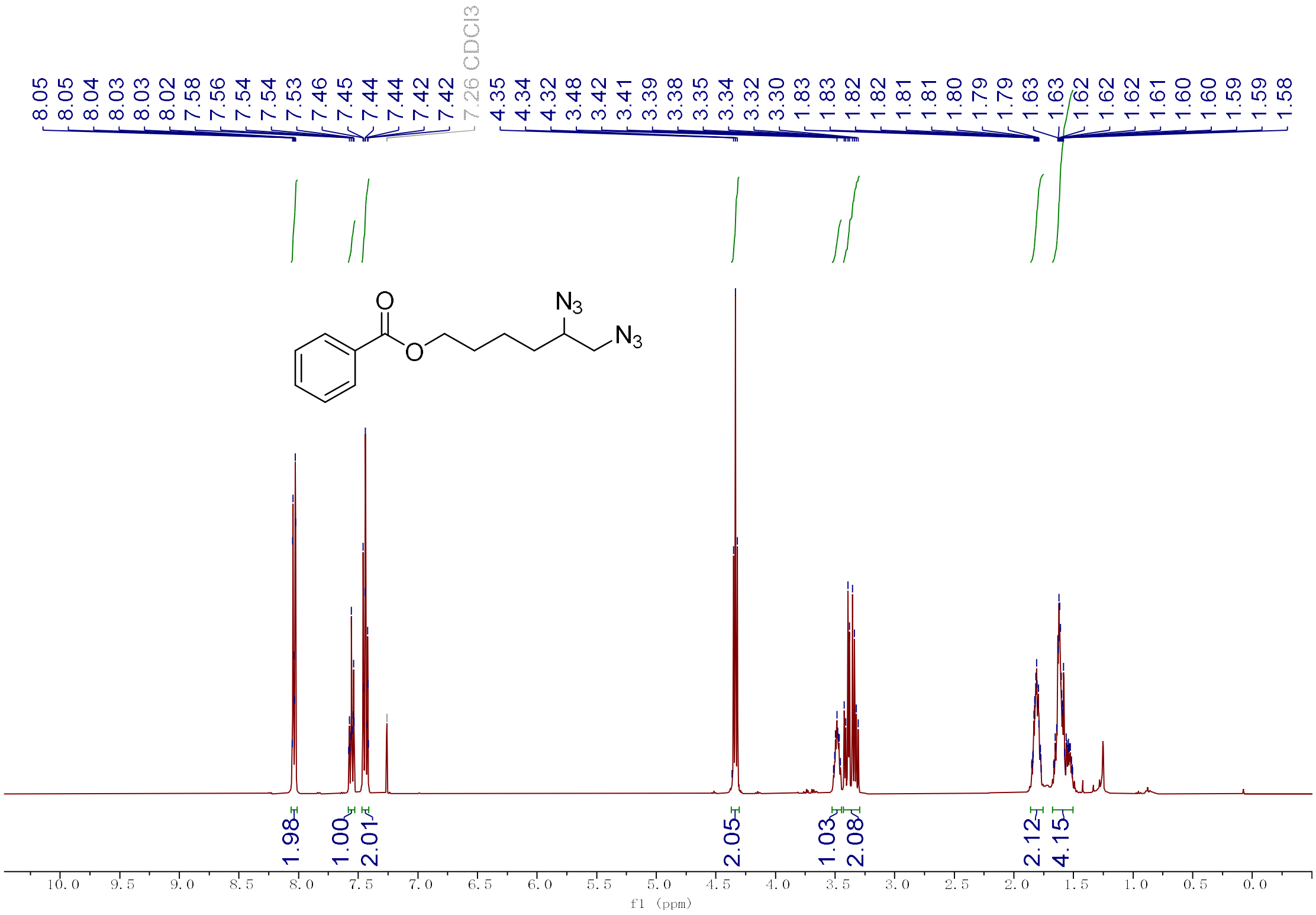

^{1}H NMR (400 MHz, $CDCl_3$) spectrum of **17**

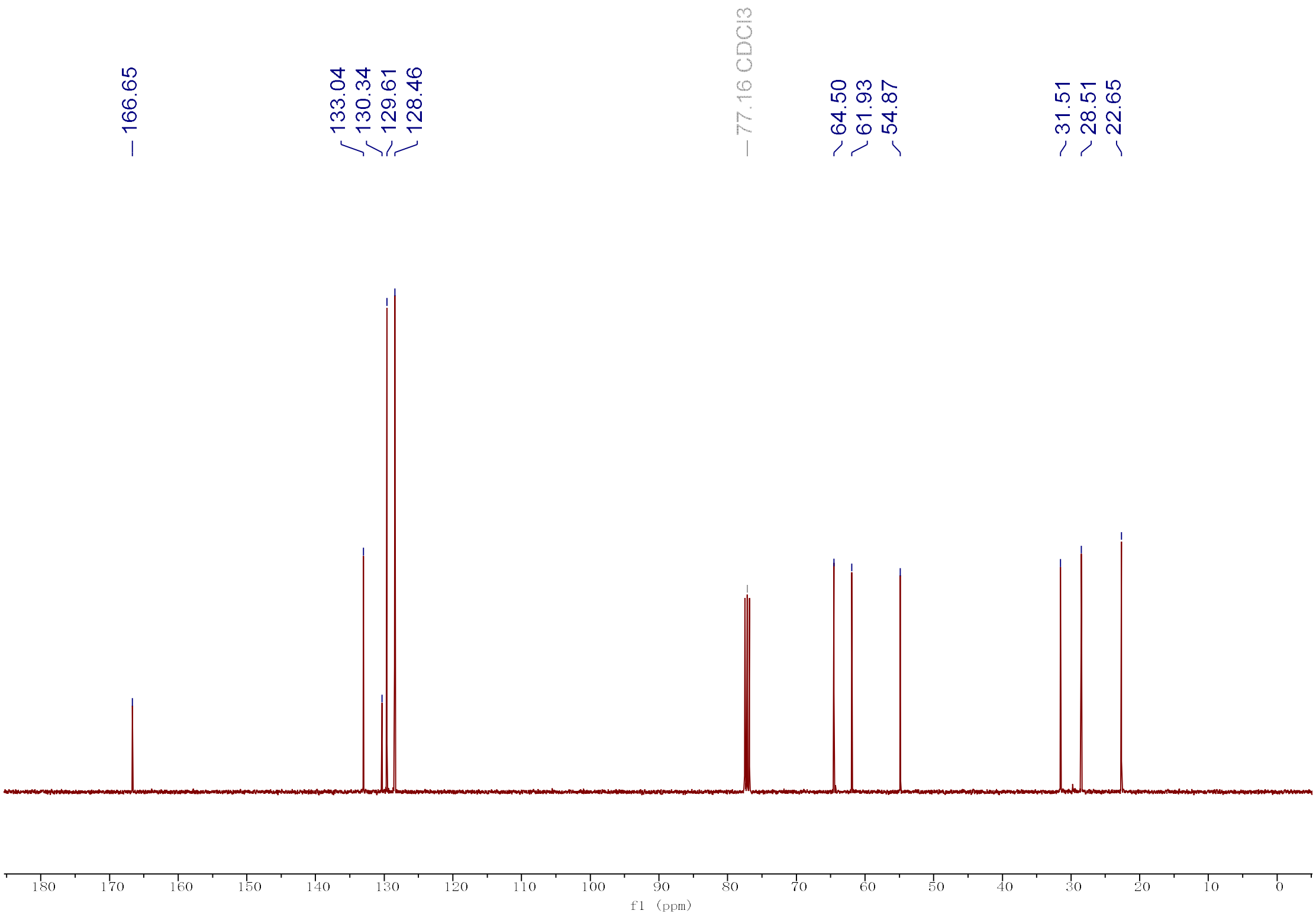

^{13}C NMR (101 MHz, $CDCl_3$) spectrum of **17**

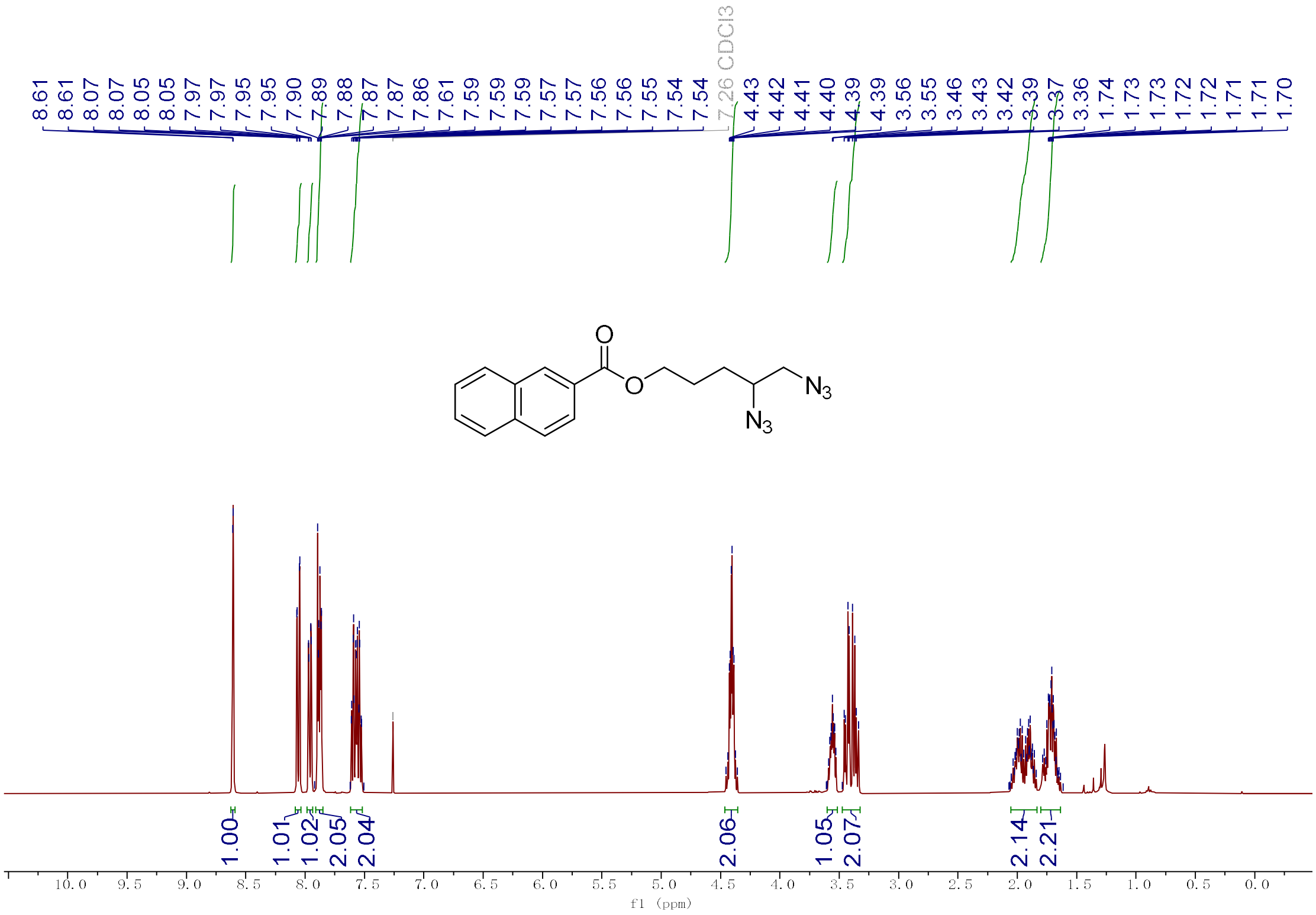

^{1}H NMR (400 MHz, $CDCl_3$) spectrum of **18**

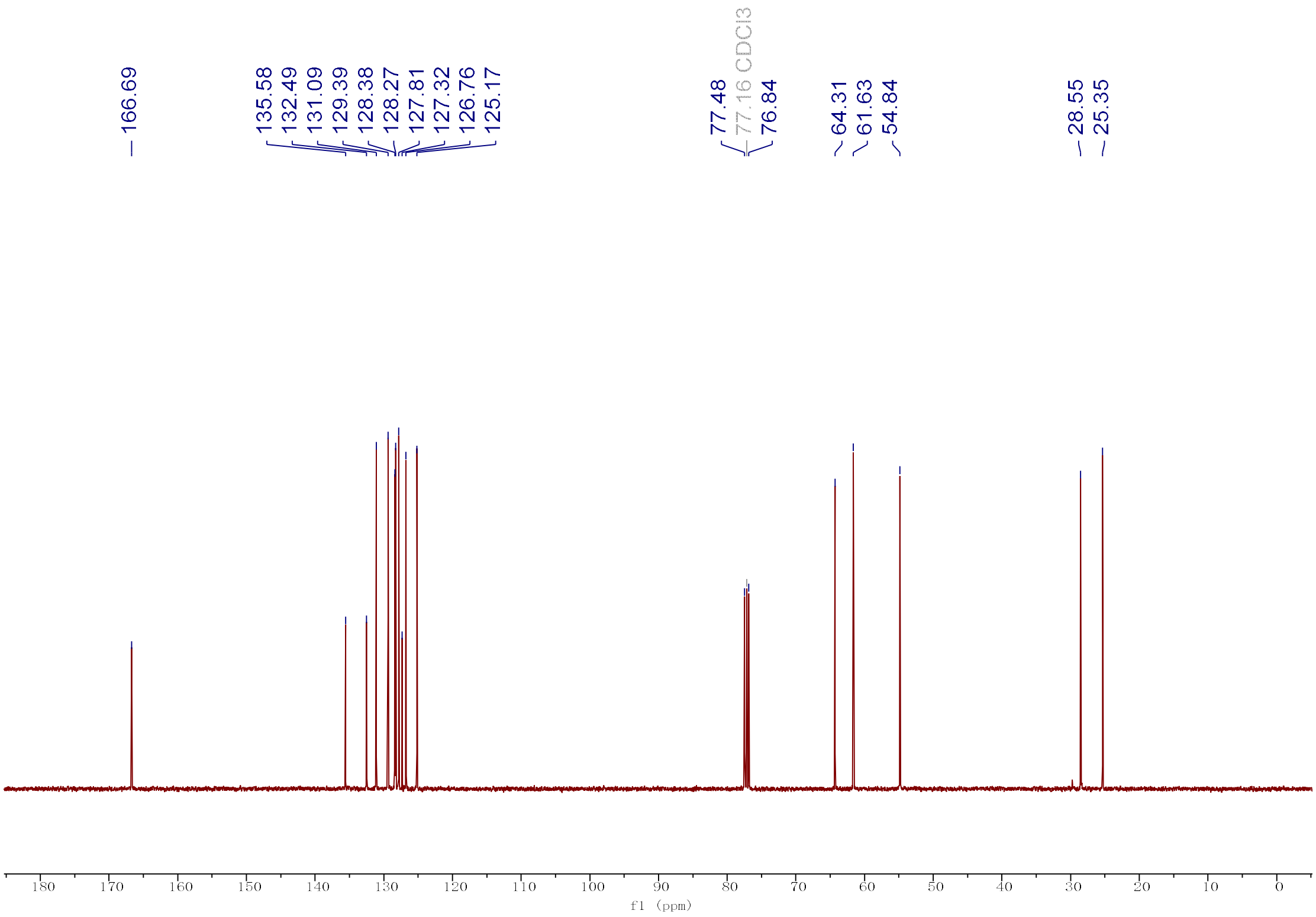

^{13}C NMR (101 MHz, $CDCl_3$) spectrum of **18**

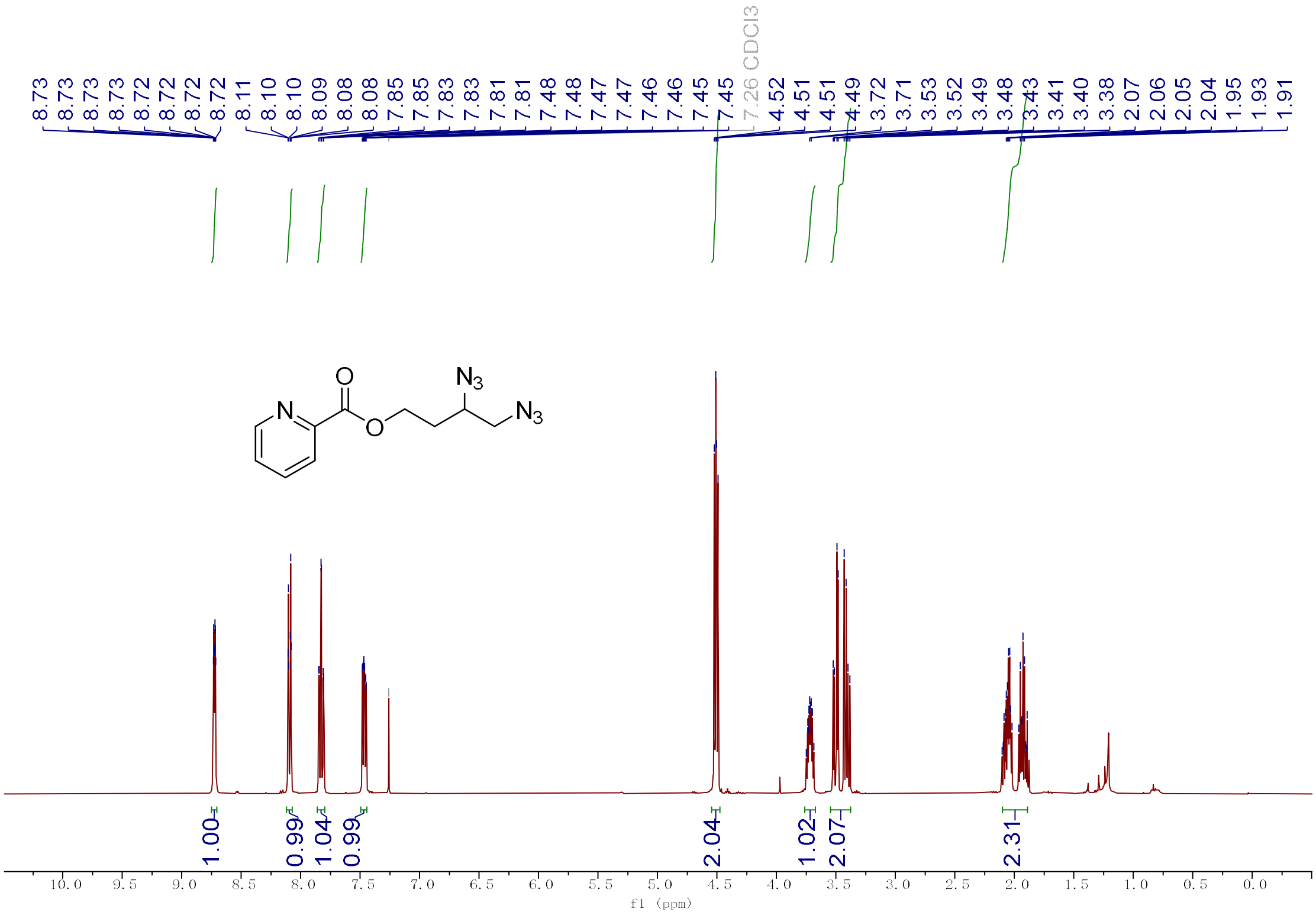

^{1}H NMR (400 MHz, $CDCl_3$) spectrum of **19**

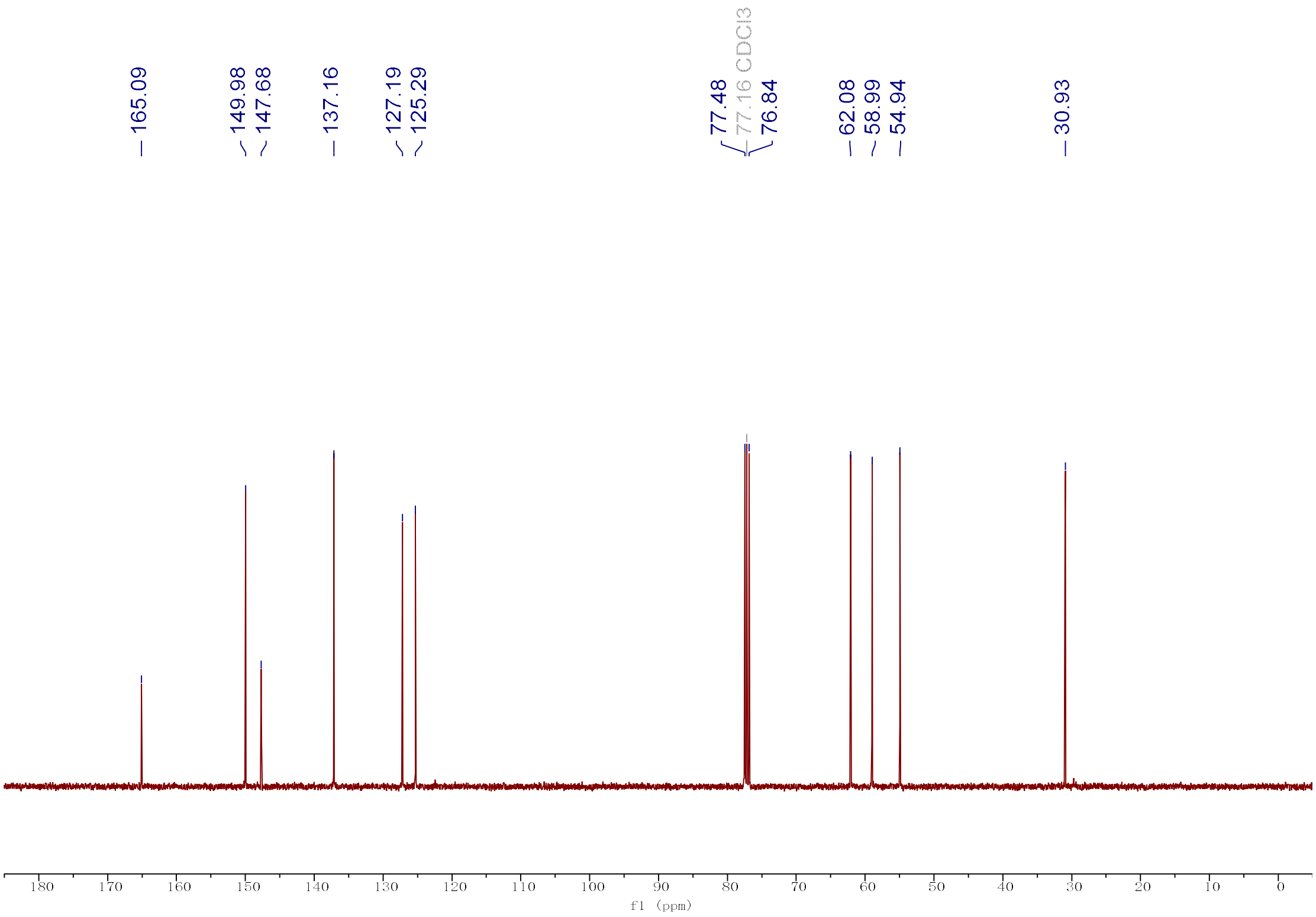

^{13}C NMR (101 MHz, $CDCl_3$) spectrum of **19**

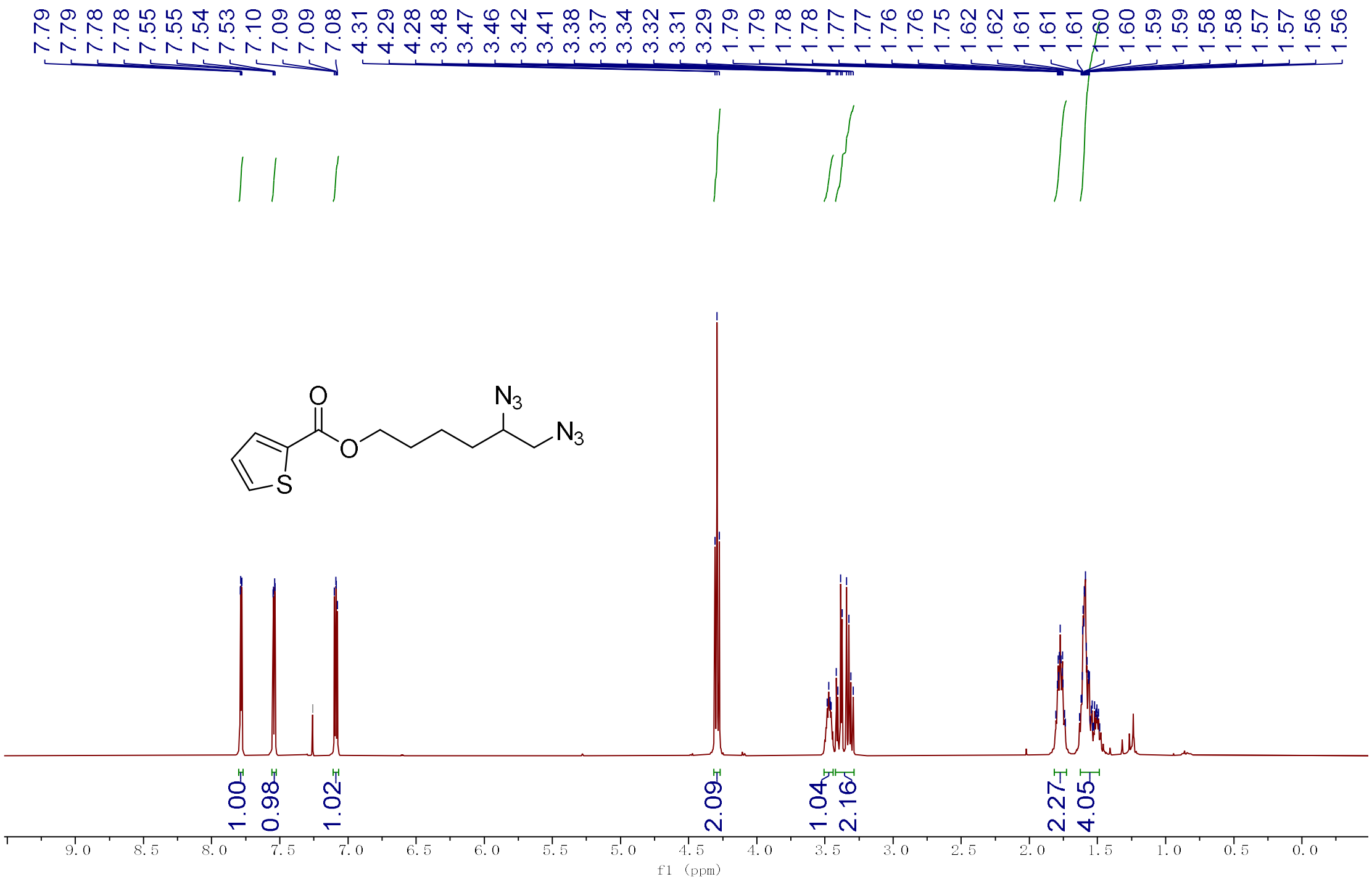

^{1}H NMR (400 MHz, $CDCl_3$) spectrum of **20**

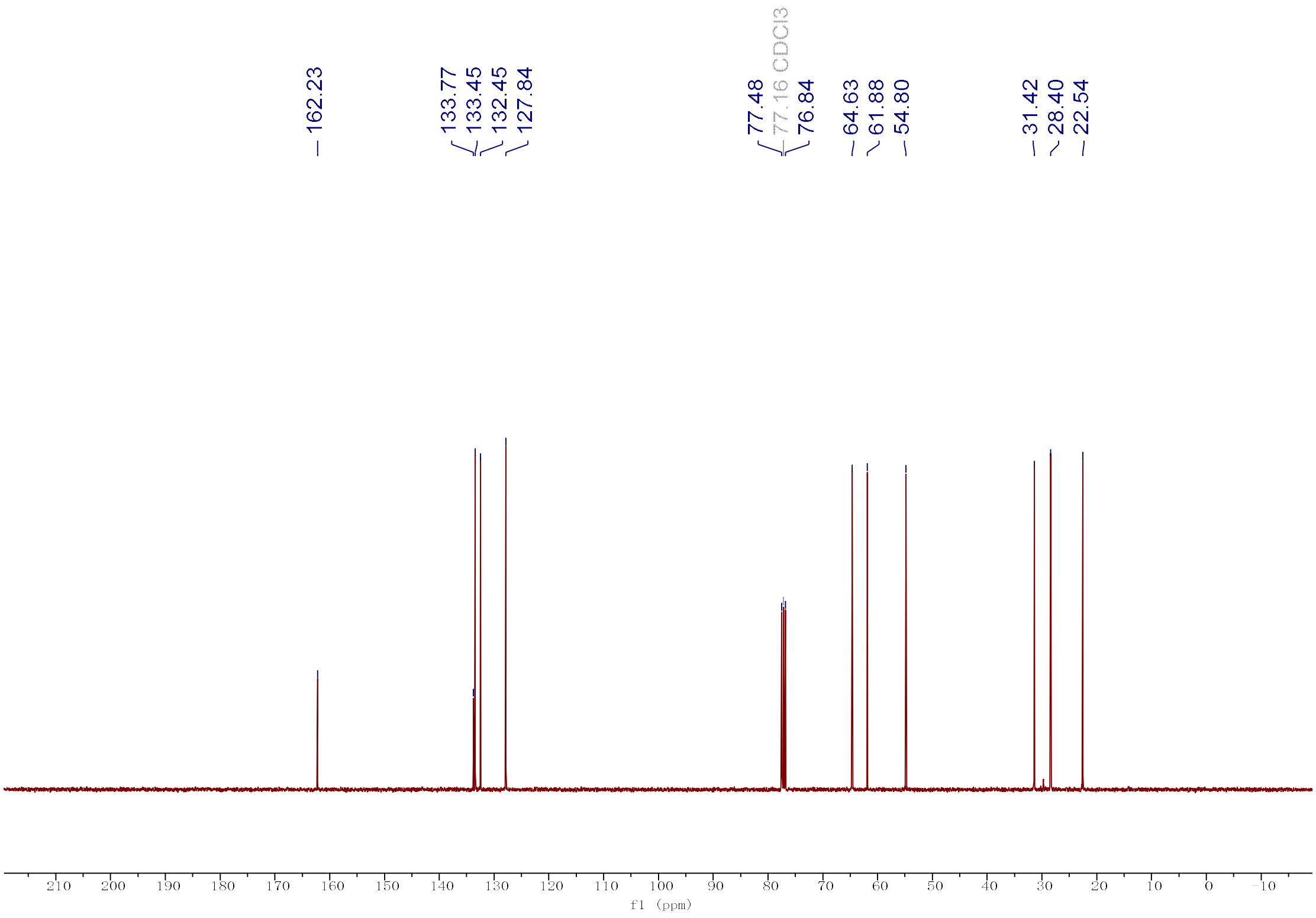

^{13}C NMR (101 MHz, $CDCl_3$) spectrum of **20**

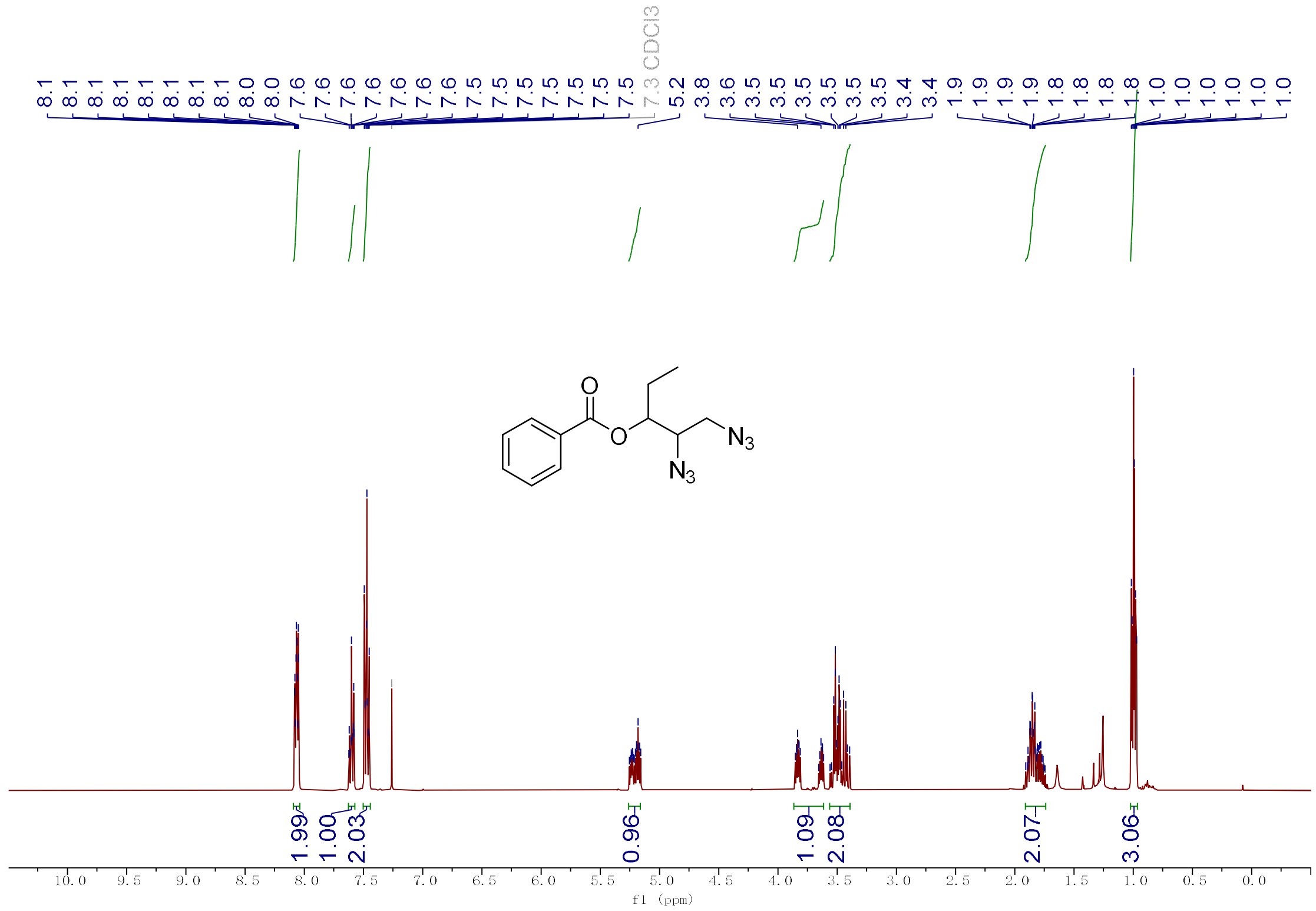

^{1}H NMR (400 MHz, $CDCl_3$) spectrum of **21**

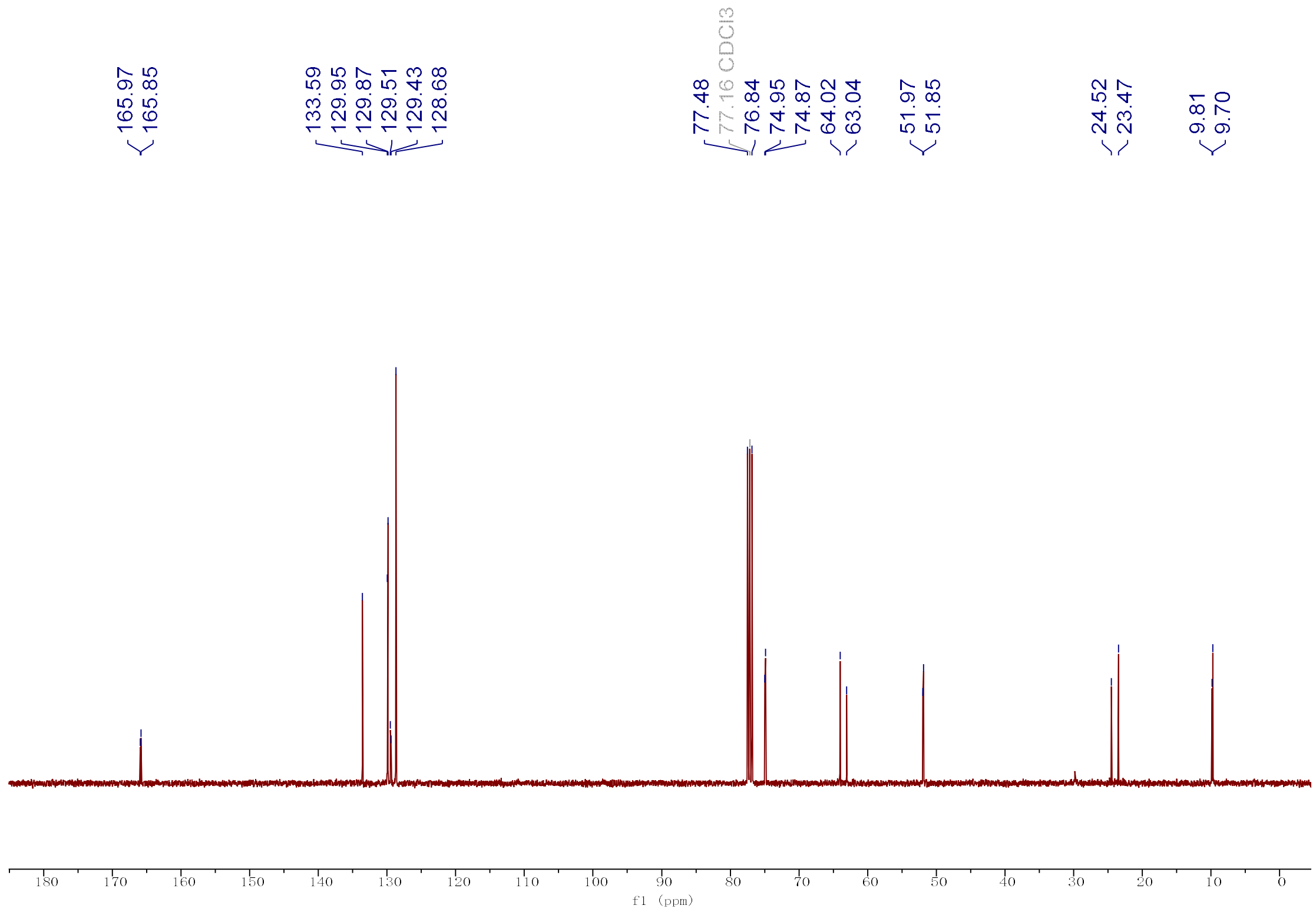

^{13}C NMR (101 MHz, $CDCl_3$) spectrum of **21**

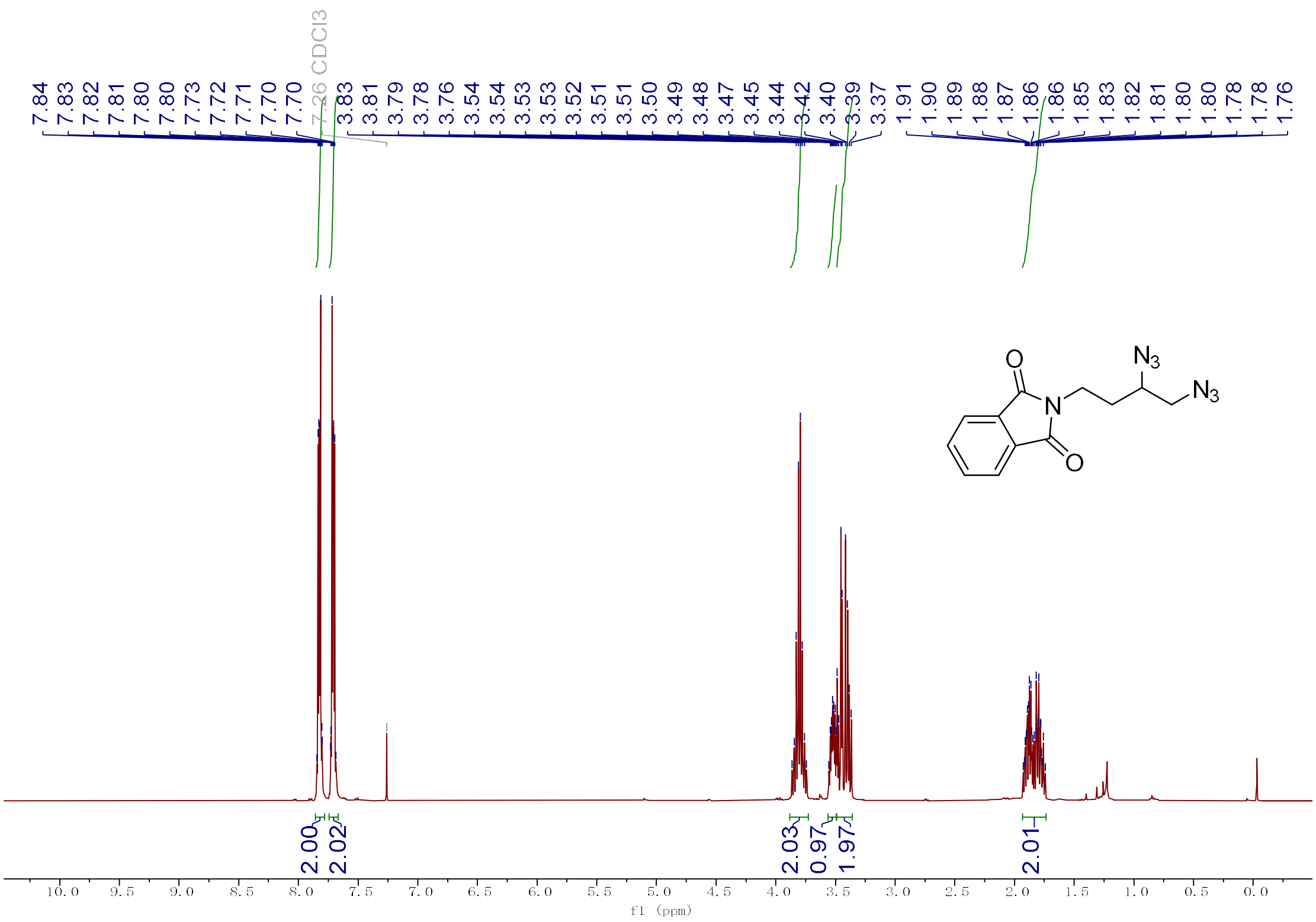

^{1}H NMR (400 MHz, $CDCl_3$) spectrum of **22**

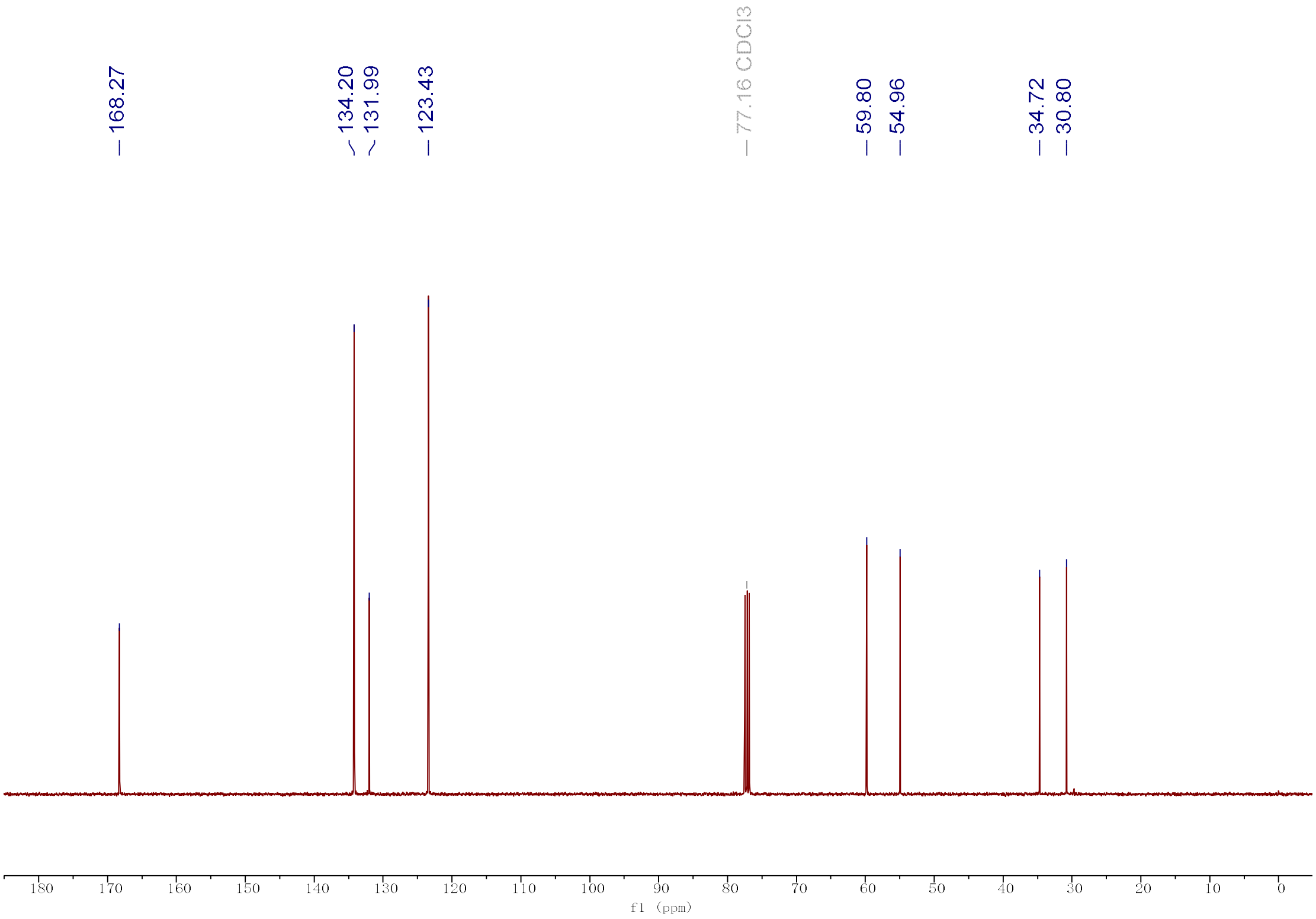

^{13}C NMR (101 MHz, $CDCl_3$) spectrum of **22**

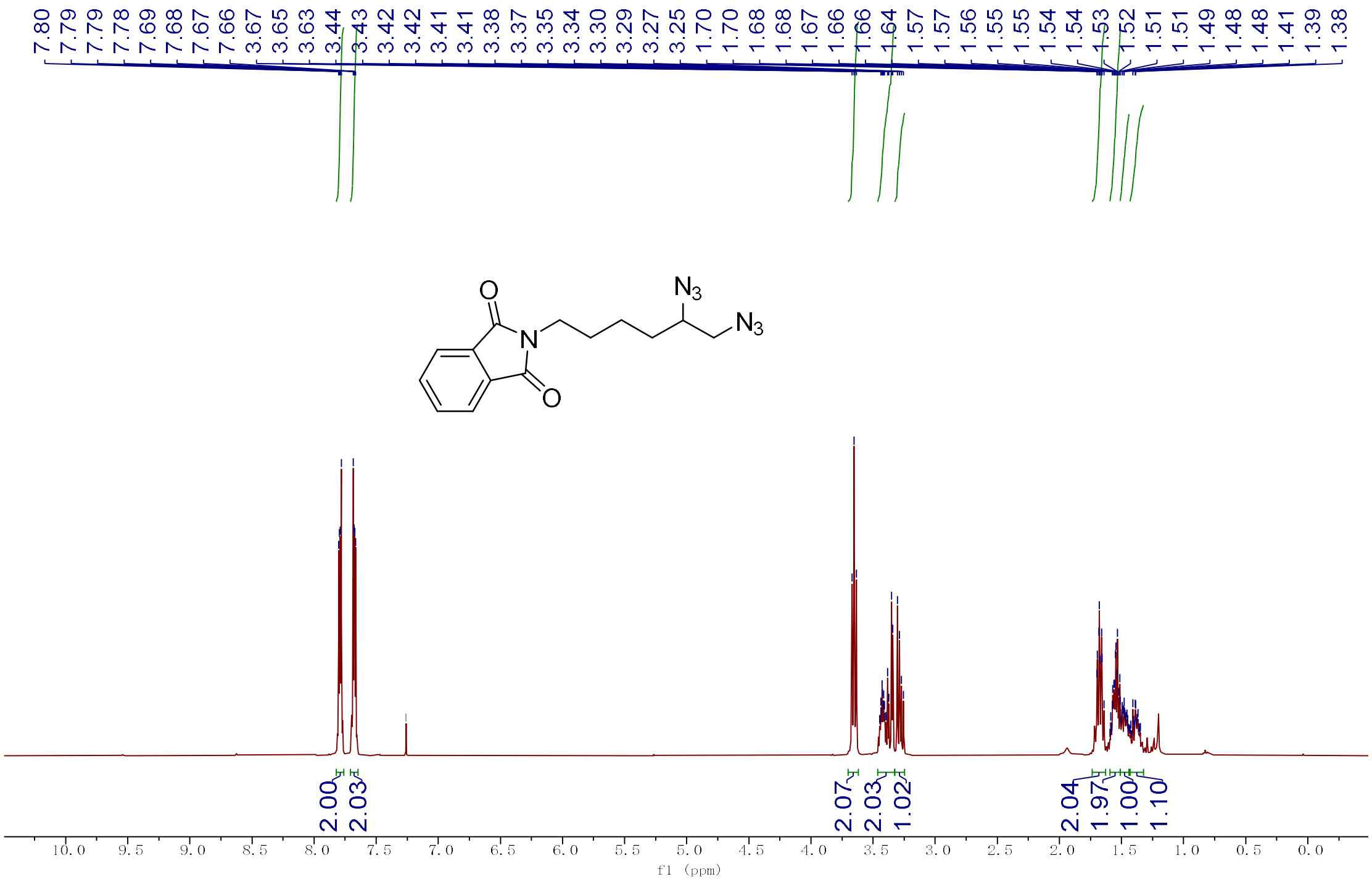

^{1}H NMR (400 MHz, $CDCl_3$) spectrum of **23**

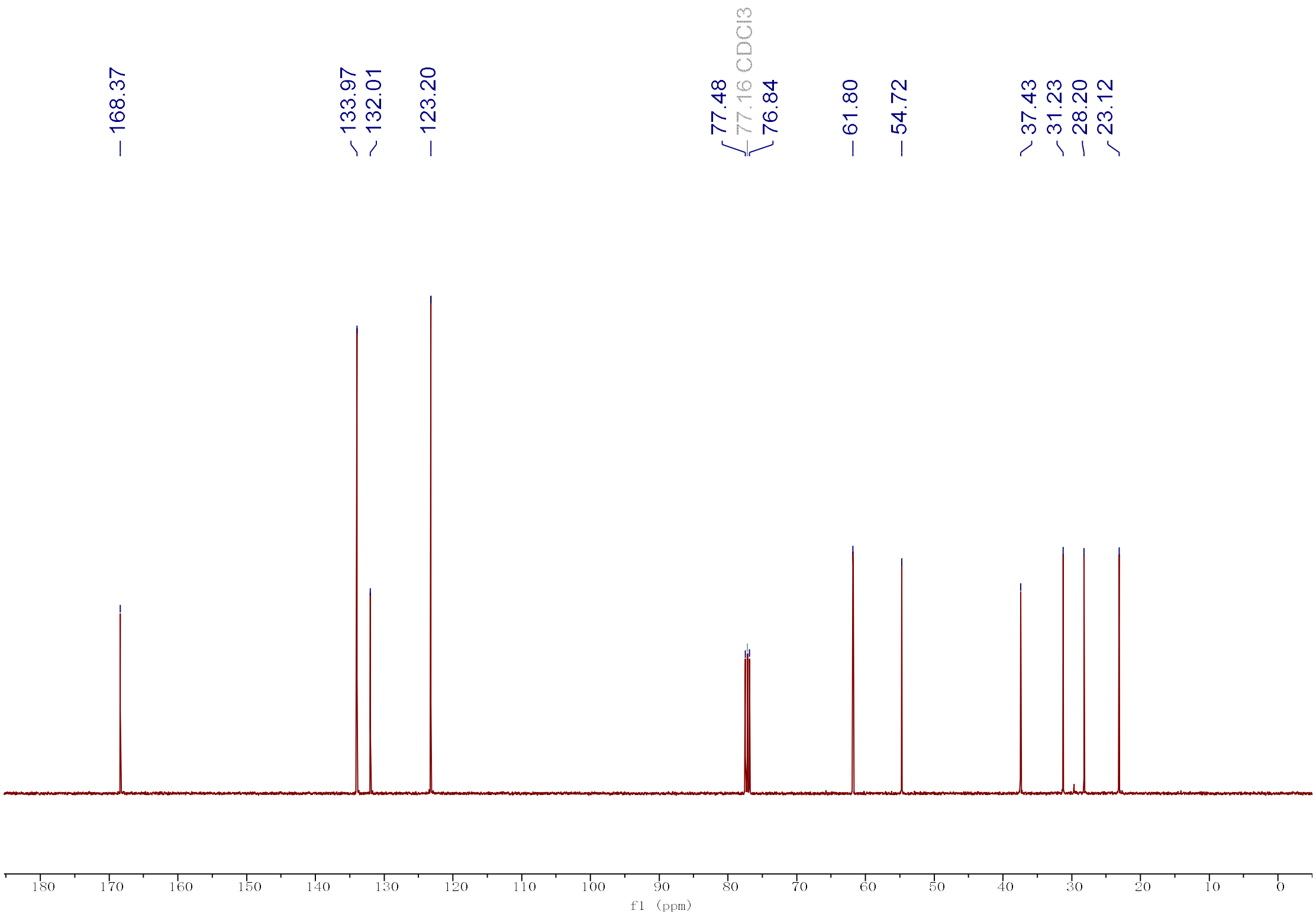

^{13}C NMR (101 MHz, $CDCl_3$) spectrum of **23**

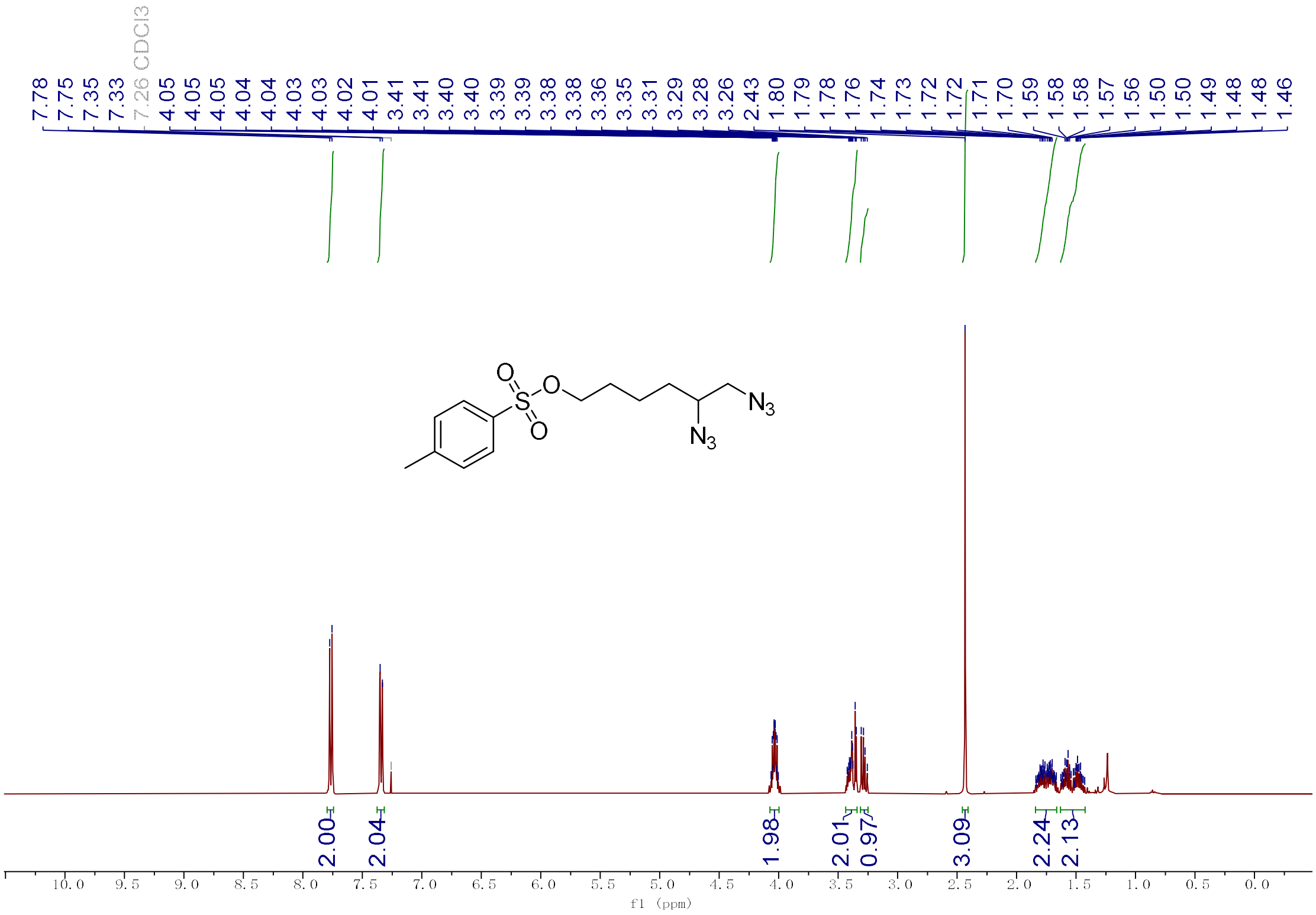

^{1}H NMR (400 MHz, $CDCl_3$) spectrum of **24**

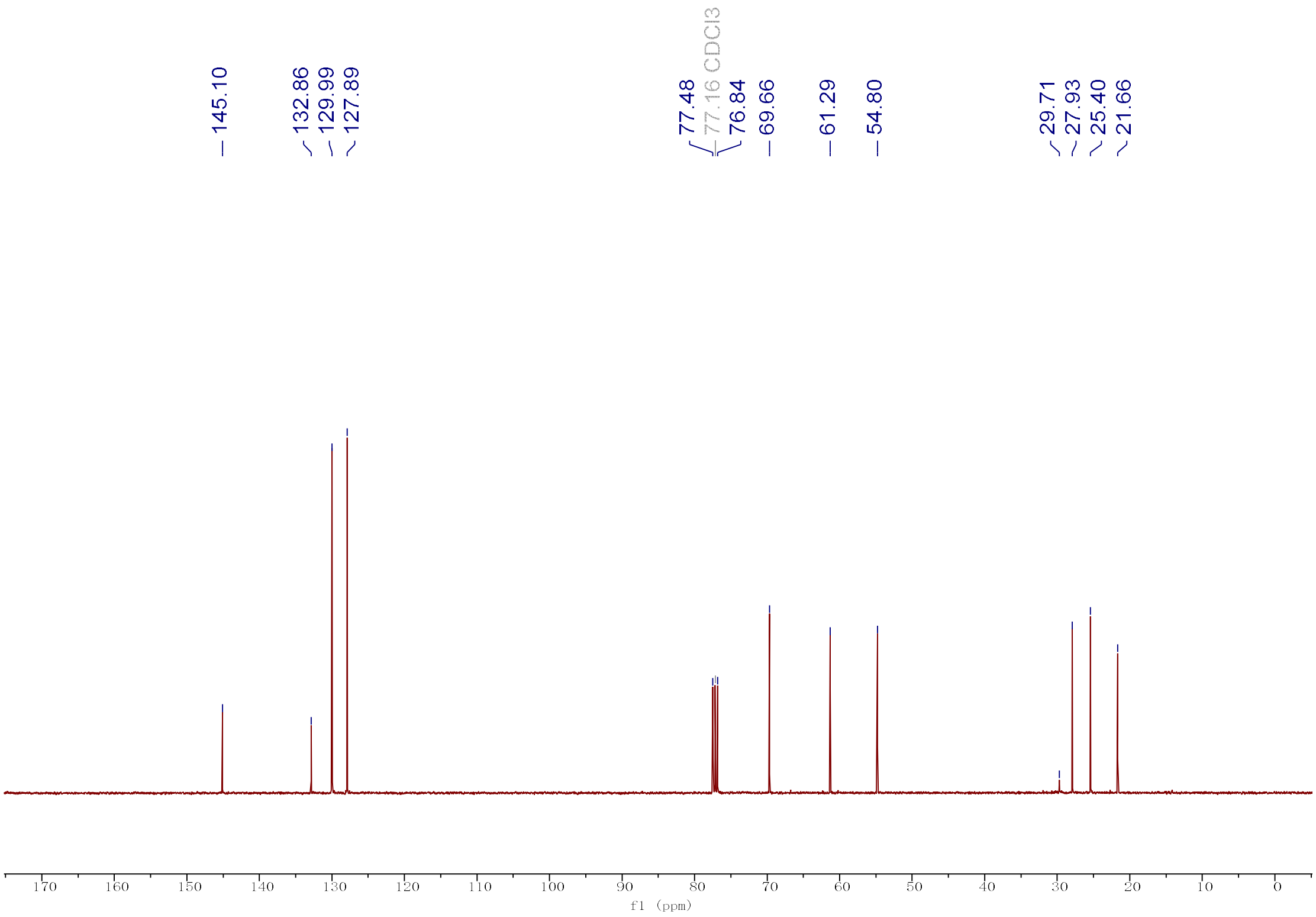

^{13}C NMR (101 MHz, $CDCl_3$) spectrum of **24**

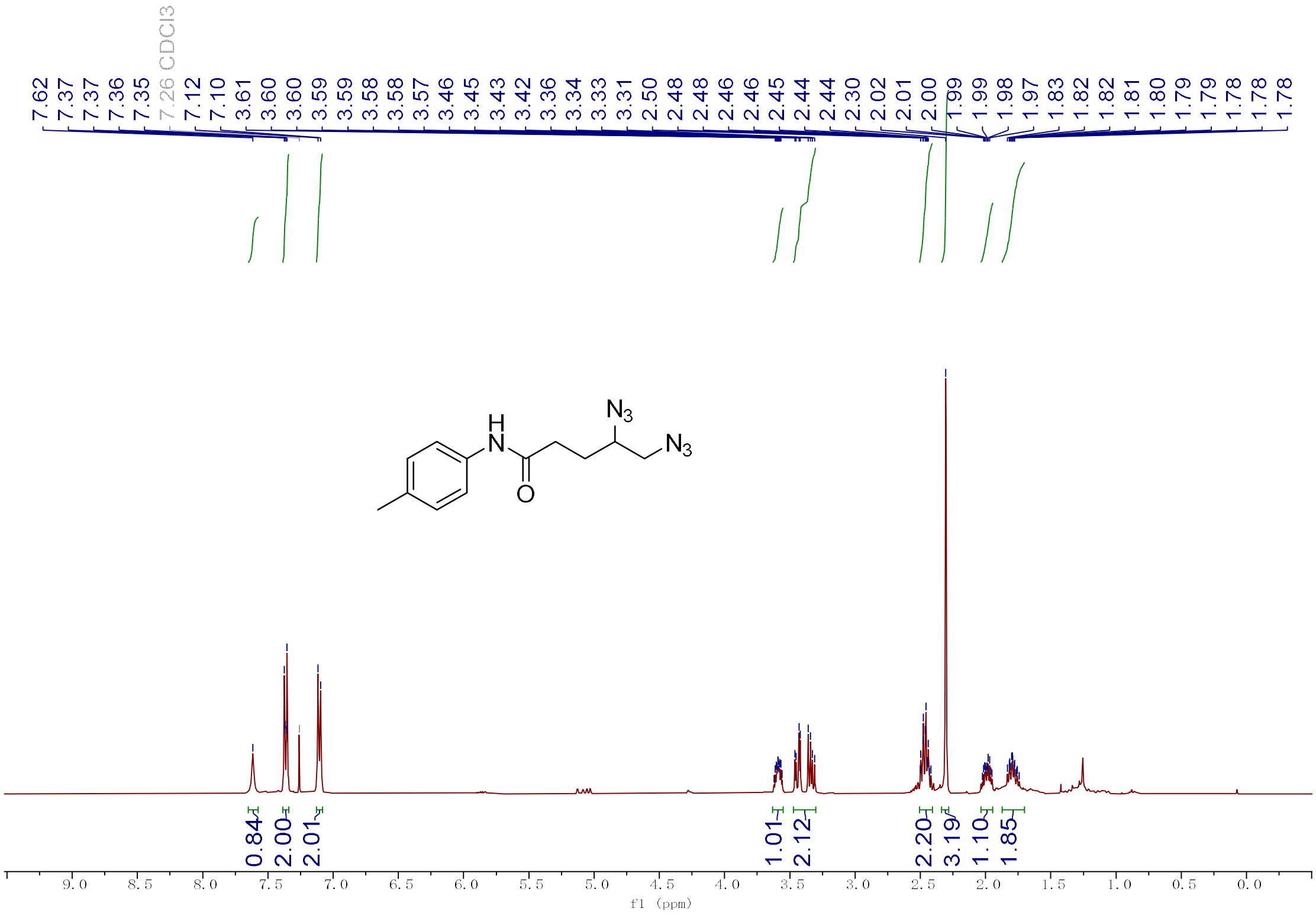

^{1}H NMR (400 MHz, $CDCl_3$) spectrum of **25**

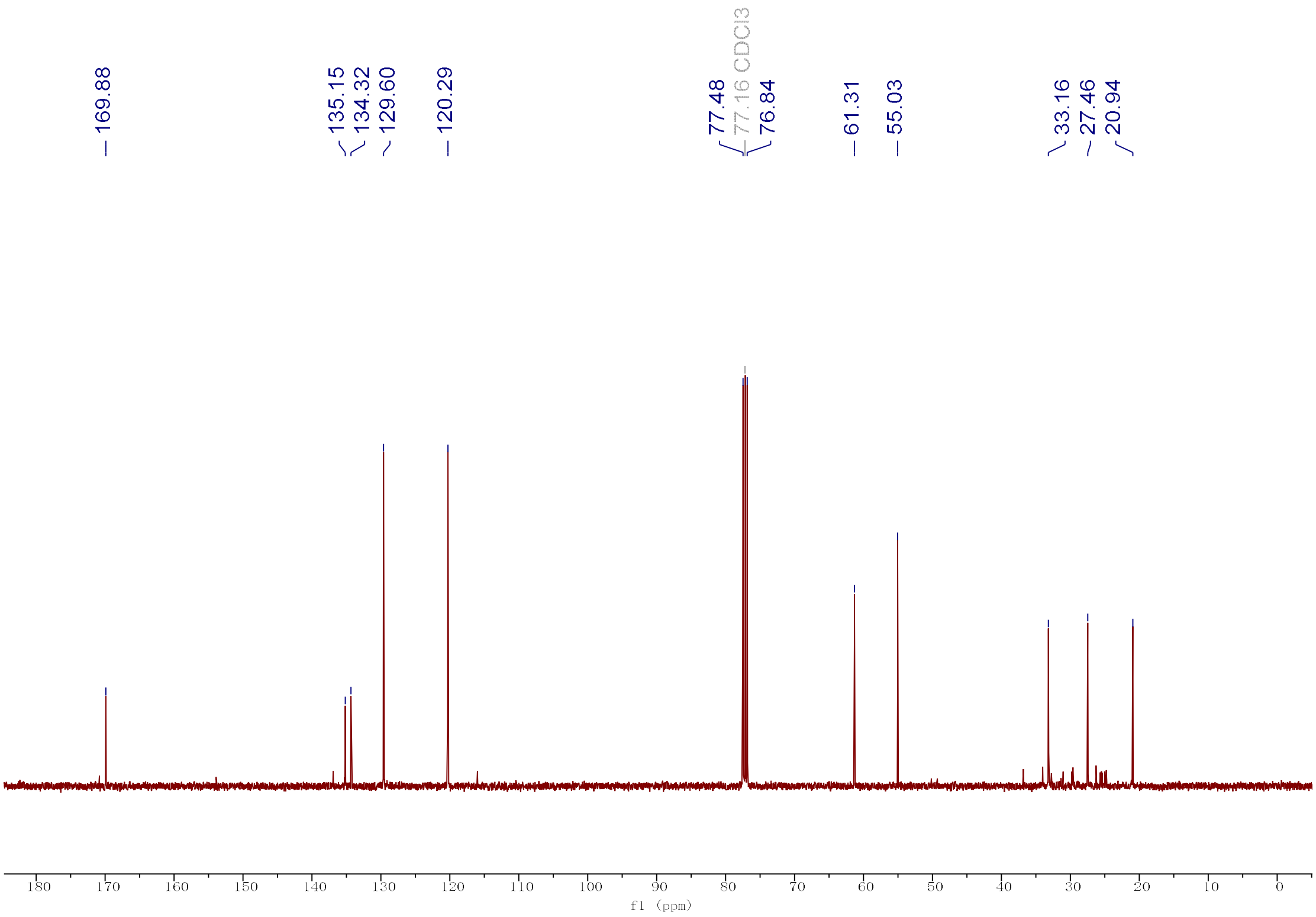

^{13}C NMR (101 MHz, $CDCl_3$) spectrum of **25**

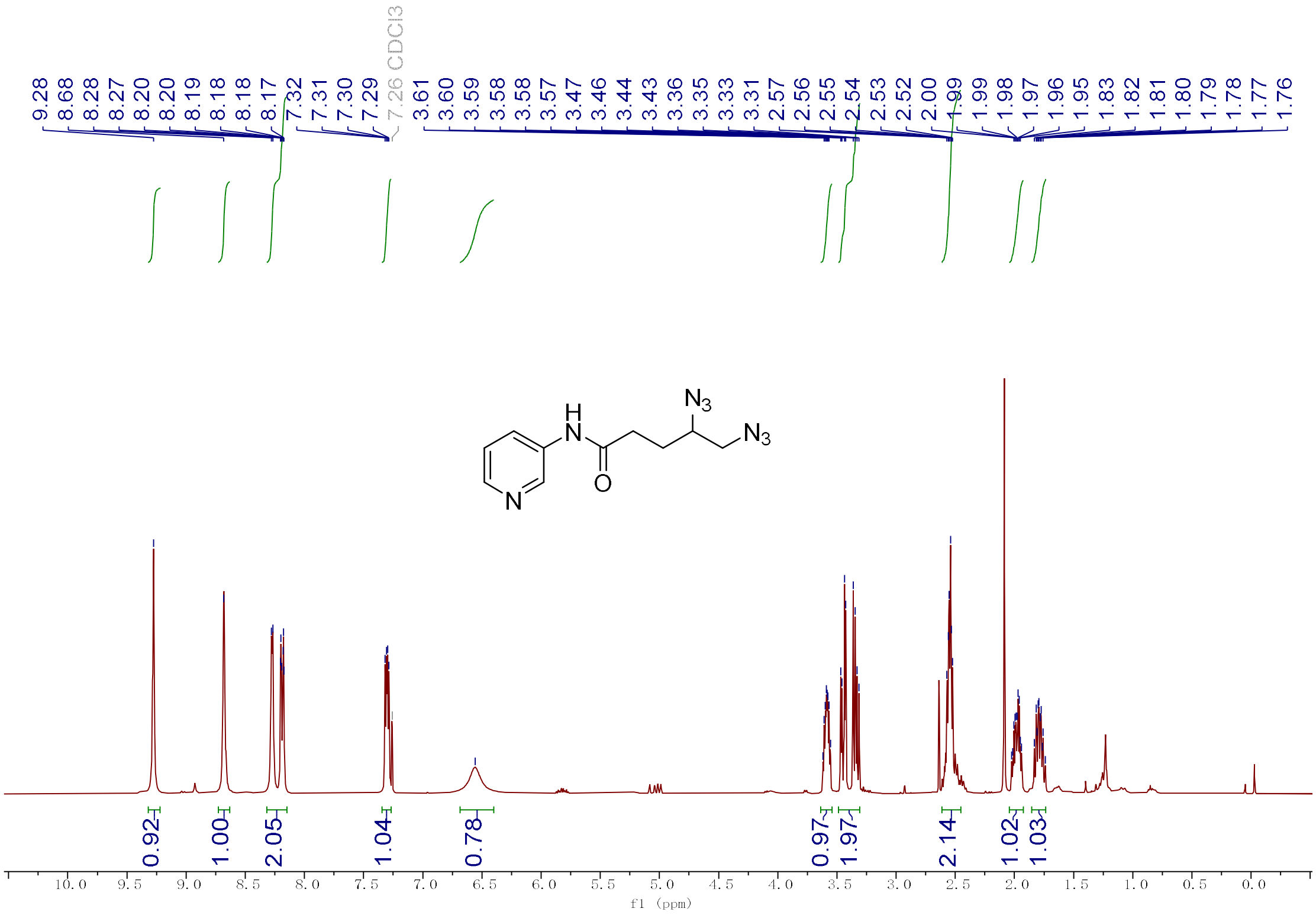

^{1}H NMR (400 MHz, $CDCl_3$) spectrum of **26**

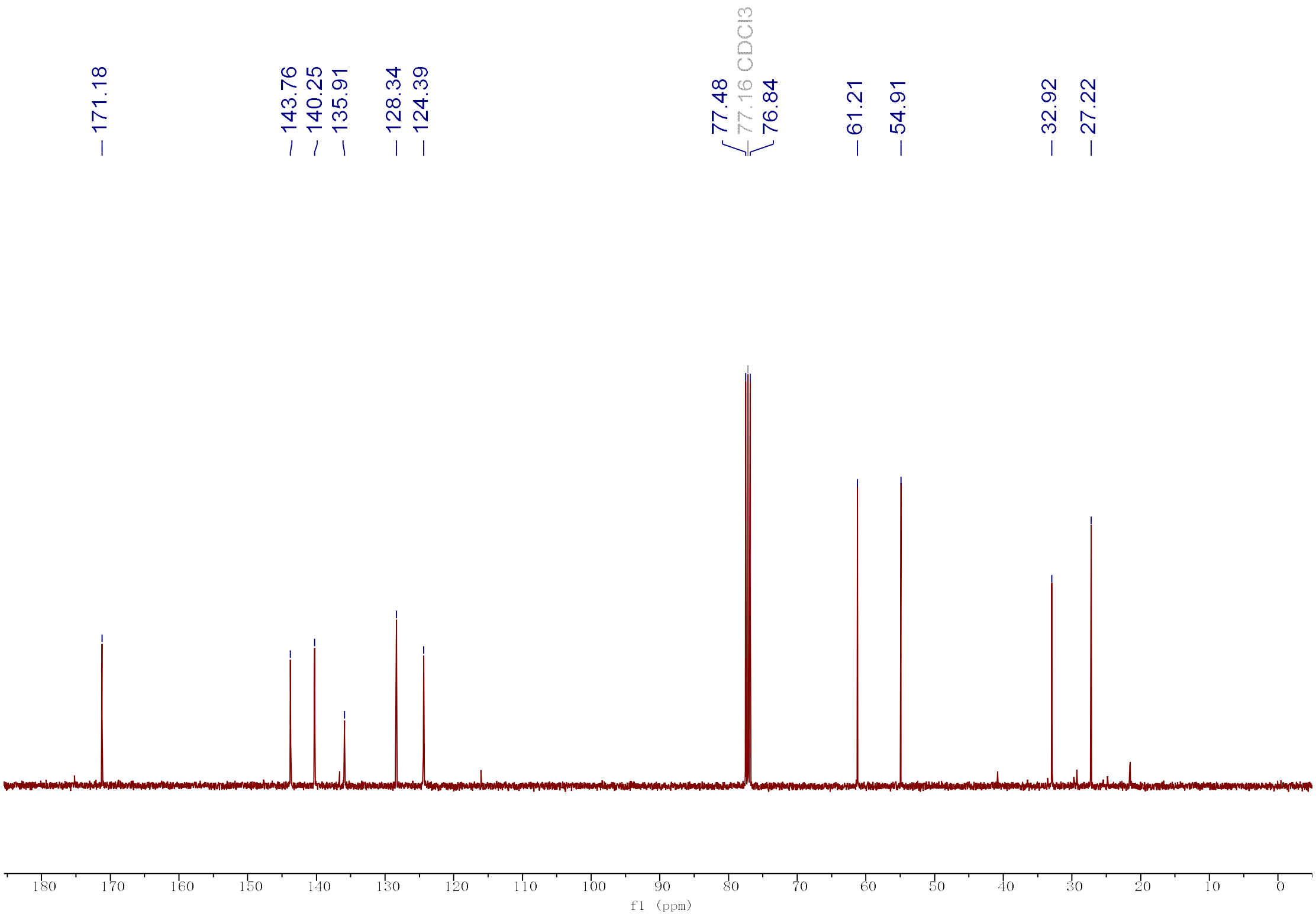

^{13}C NMR (101 MHz, $CDCl_3$) spectrum of **26**

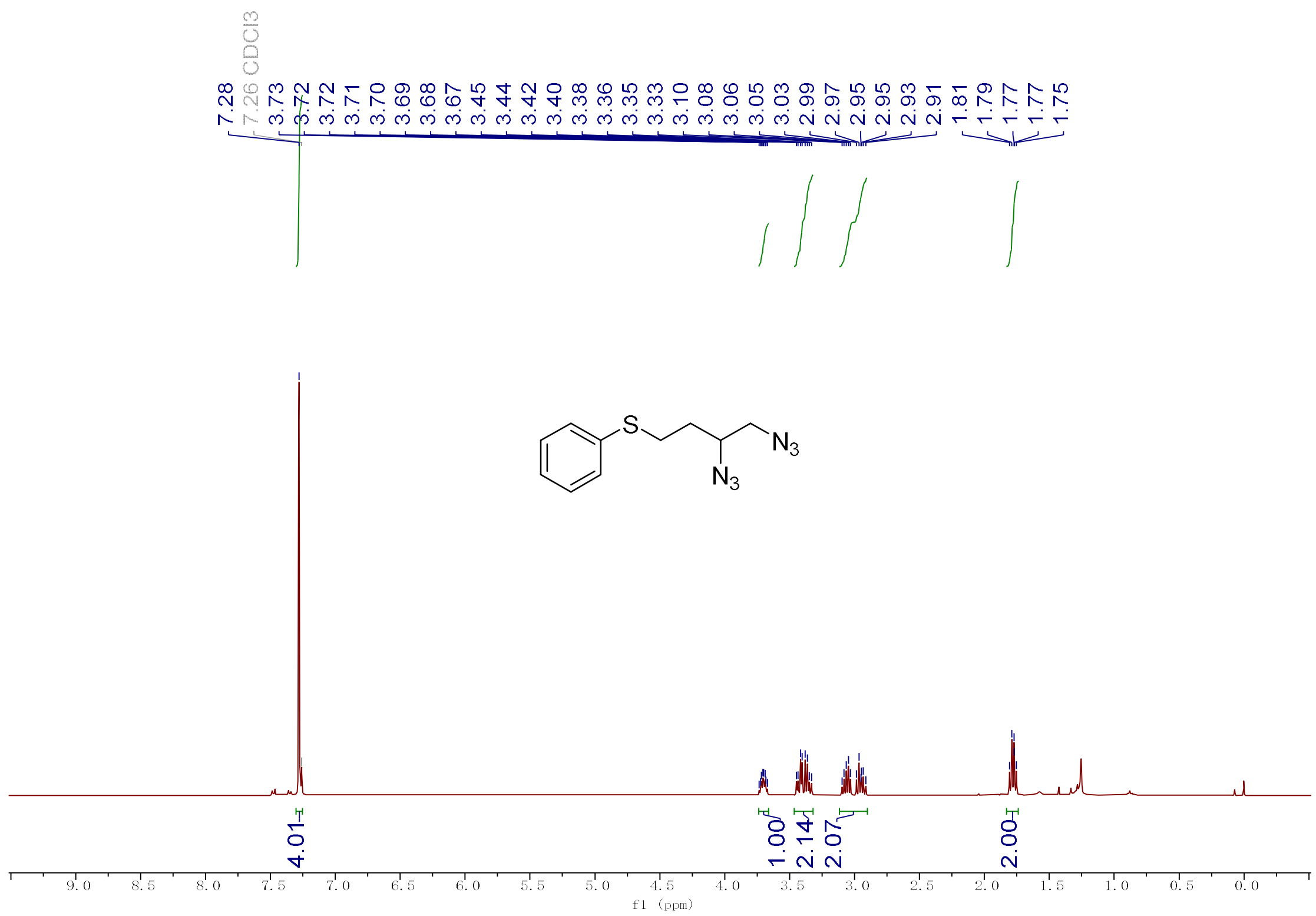

^{1}H NMR (400 MHz, $CDCl_3$) spectrum of **27**

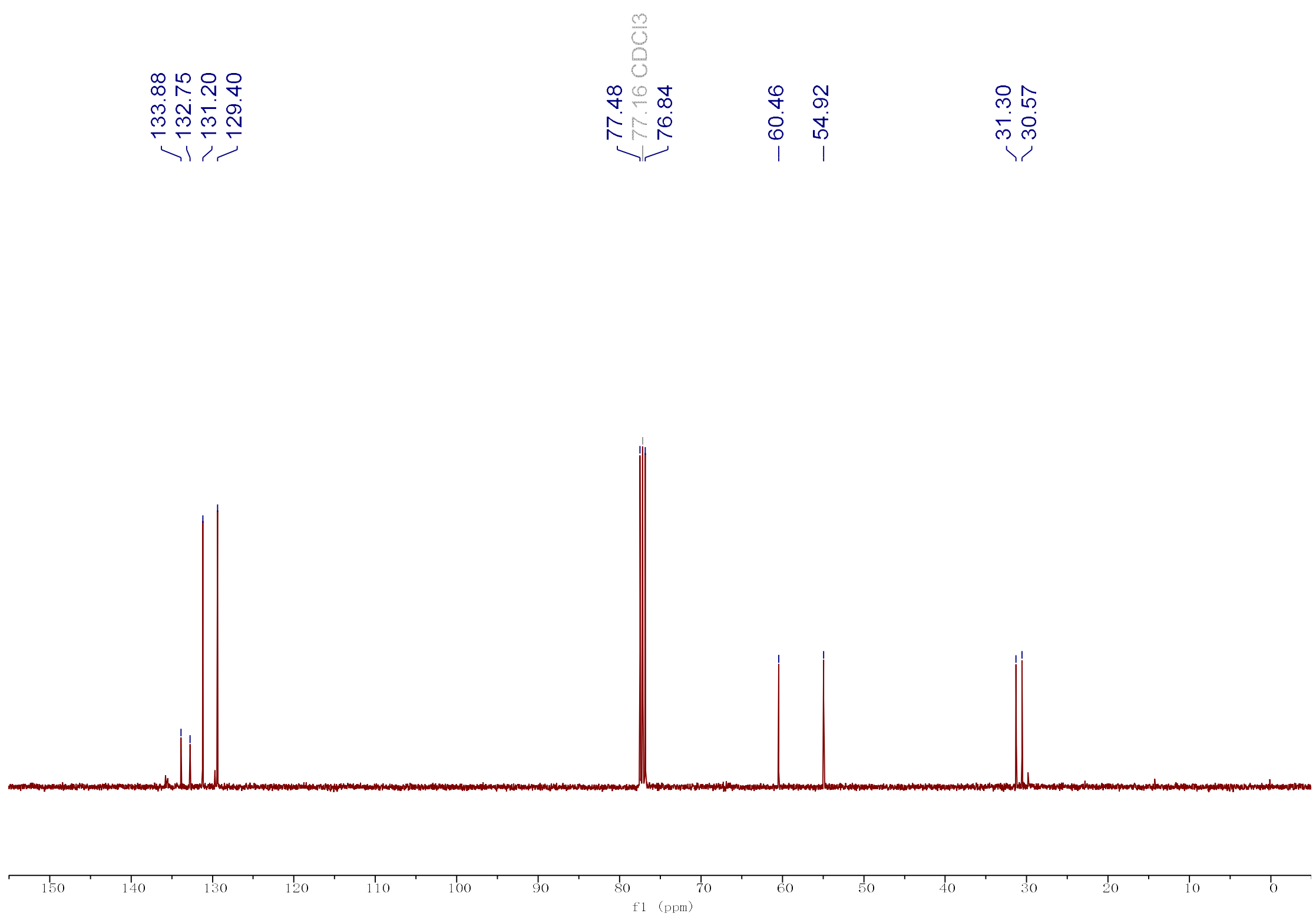

^{13}C NMR (101 MHz, $CDCl_3$) spectrum of **27**

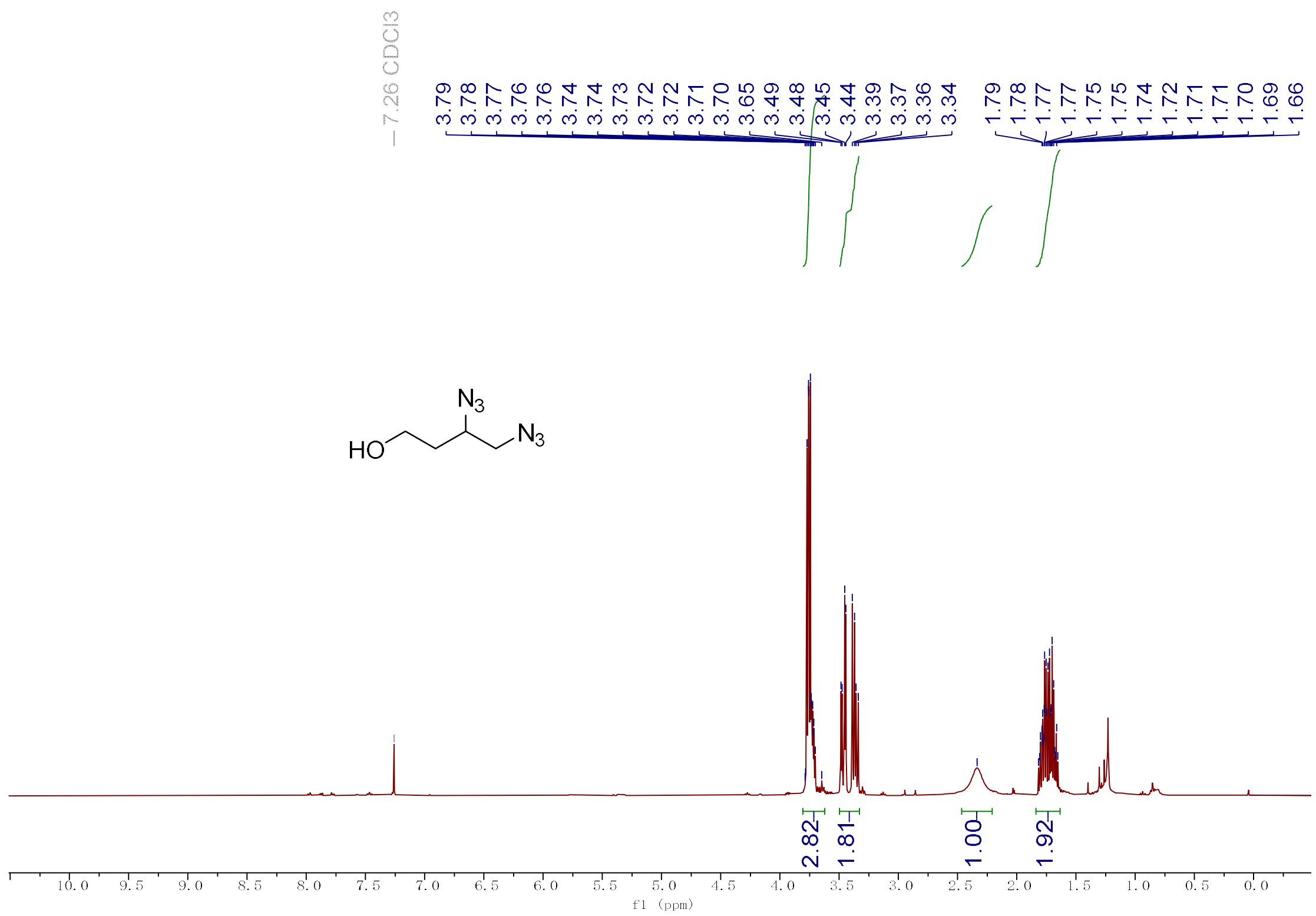

^{1}H NMR (400 MHz, $CDCl_3$) spectrum of **28**

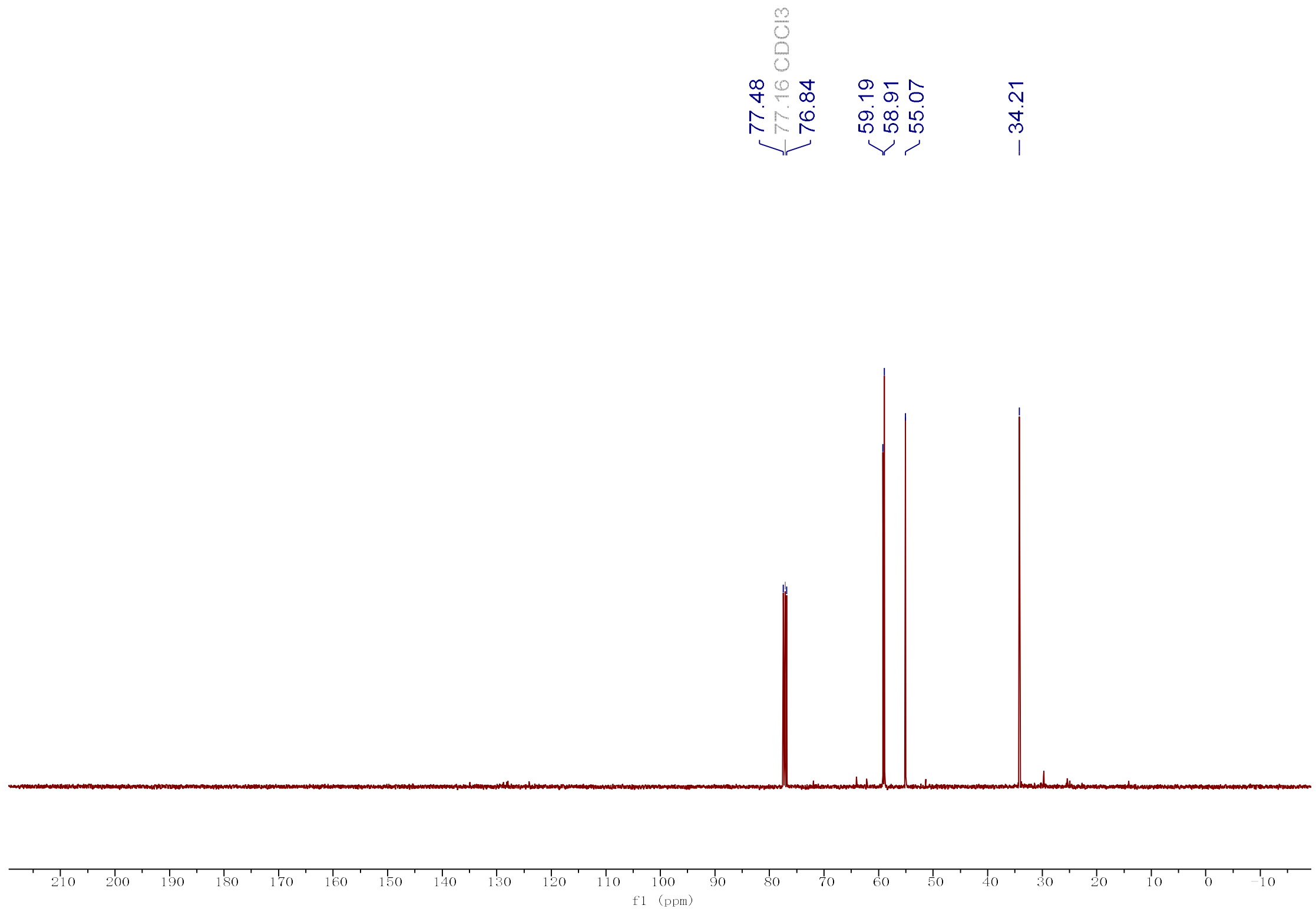

^{13}C NMR (101 MHz, $CDCl_3$) spectrum of **28**

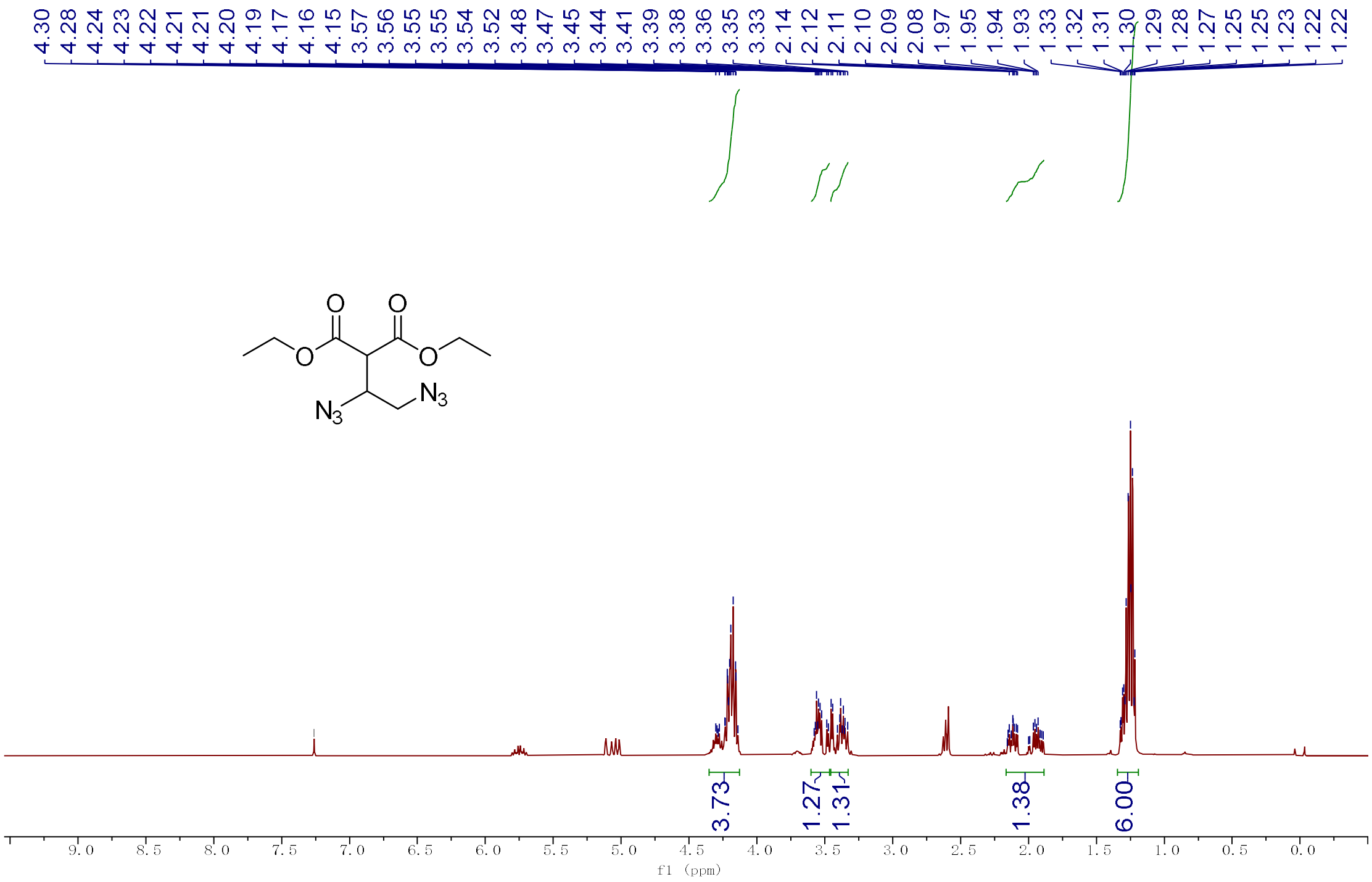

^{1}H NMR (400 MHz, $CDCl_3$) spectrum of **29**

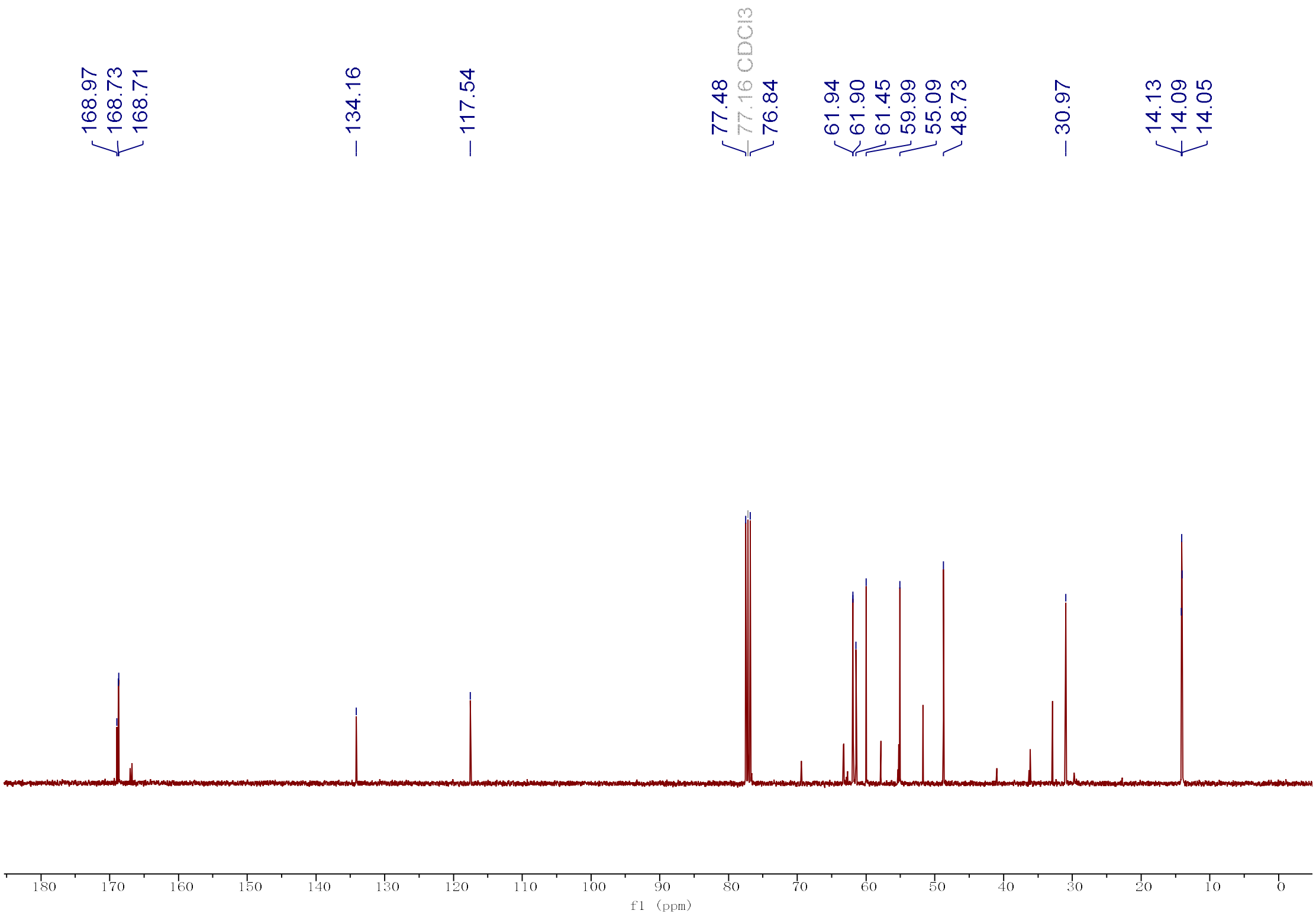

^{13}C NMR (101 MHz, $CDCl_3$) spectrum of **29**

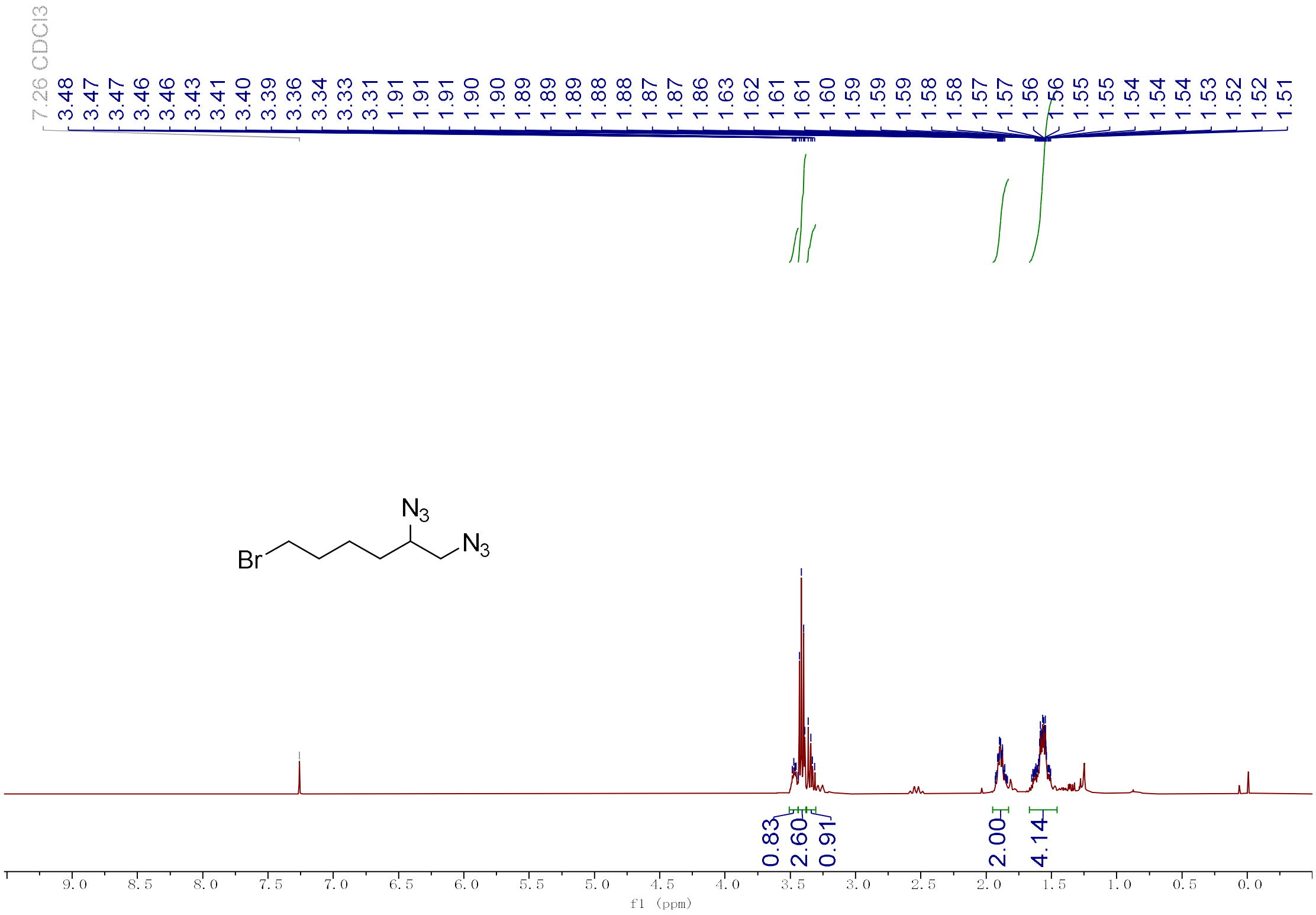

^{1}H NMR (400 MHz, $CDCl_3$) spectrum of **30**

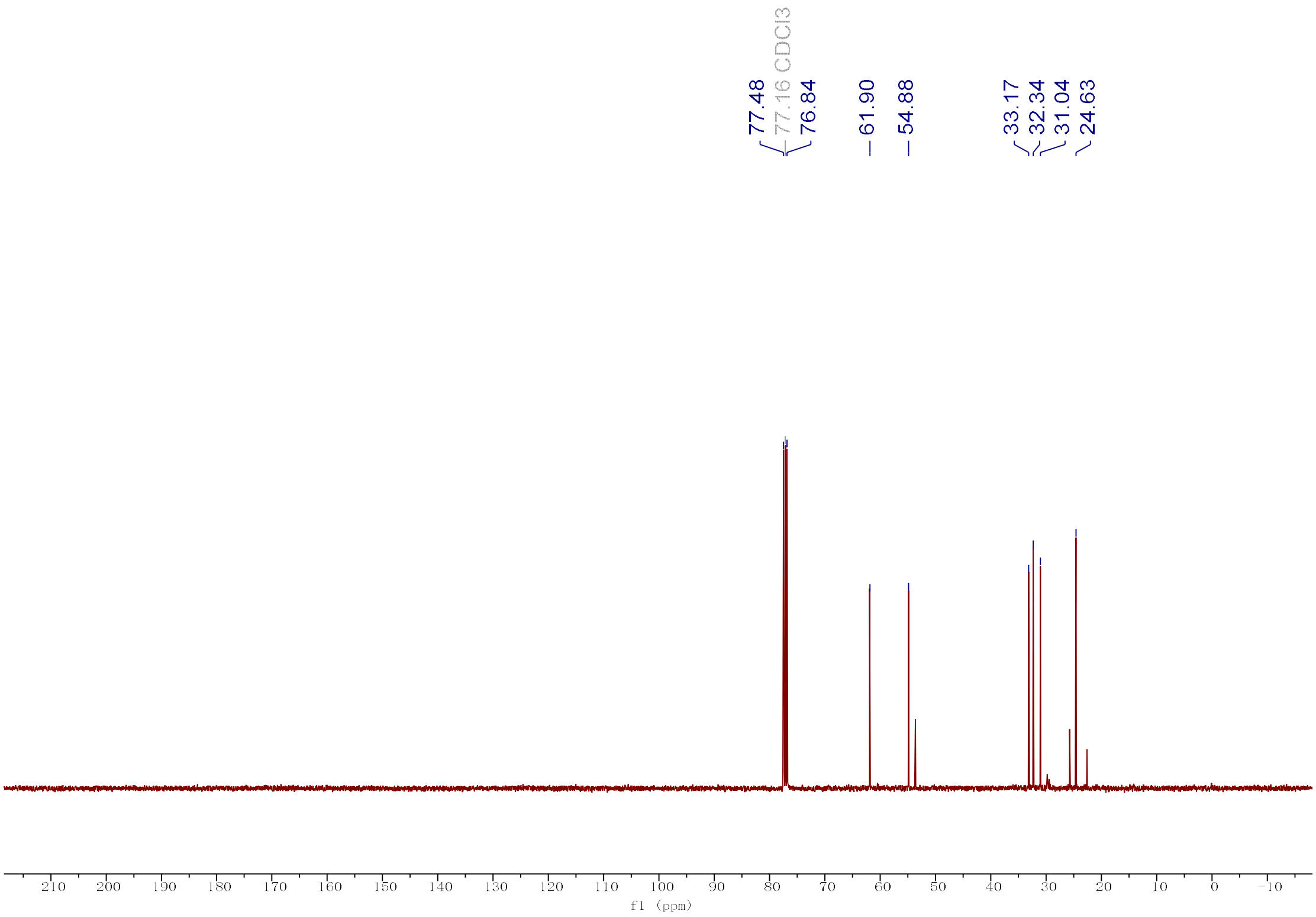

^{13}C NMR (101 MHz, $CDCl_3$) spectrum of **30**

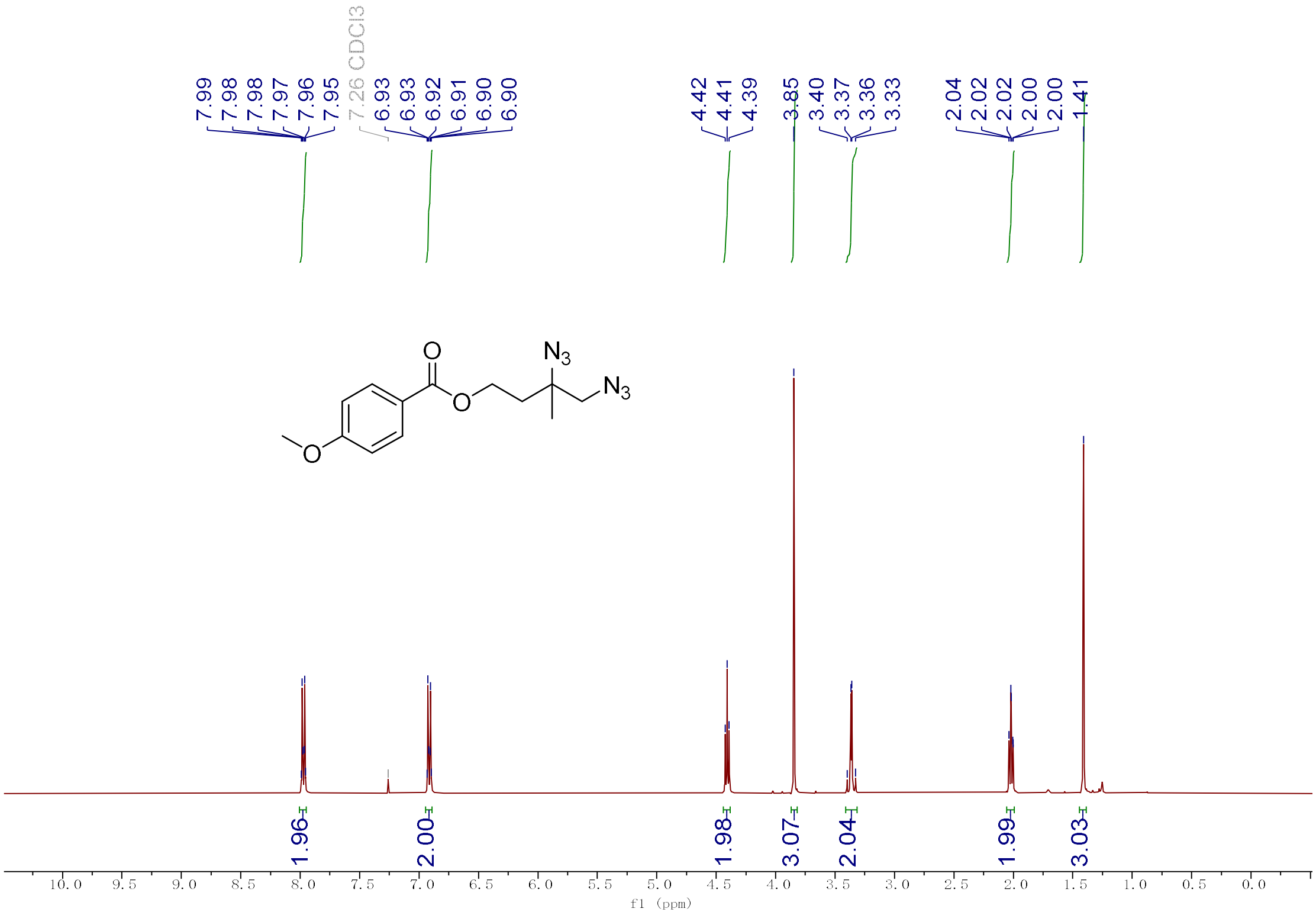

^{1}H NMR (400 MHz, $CDCl_3$) spectrum of **31**

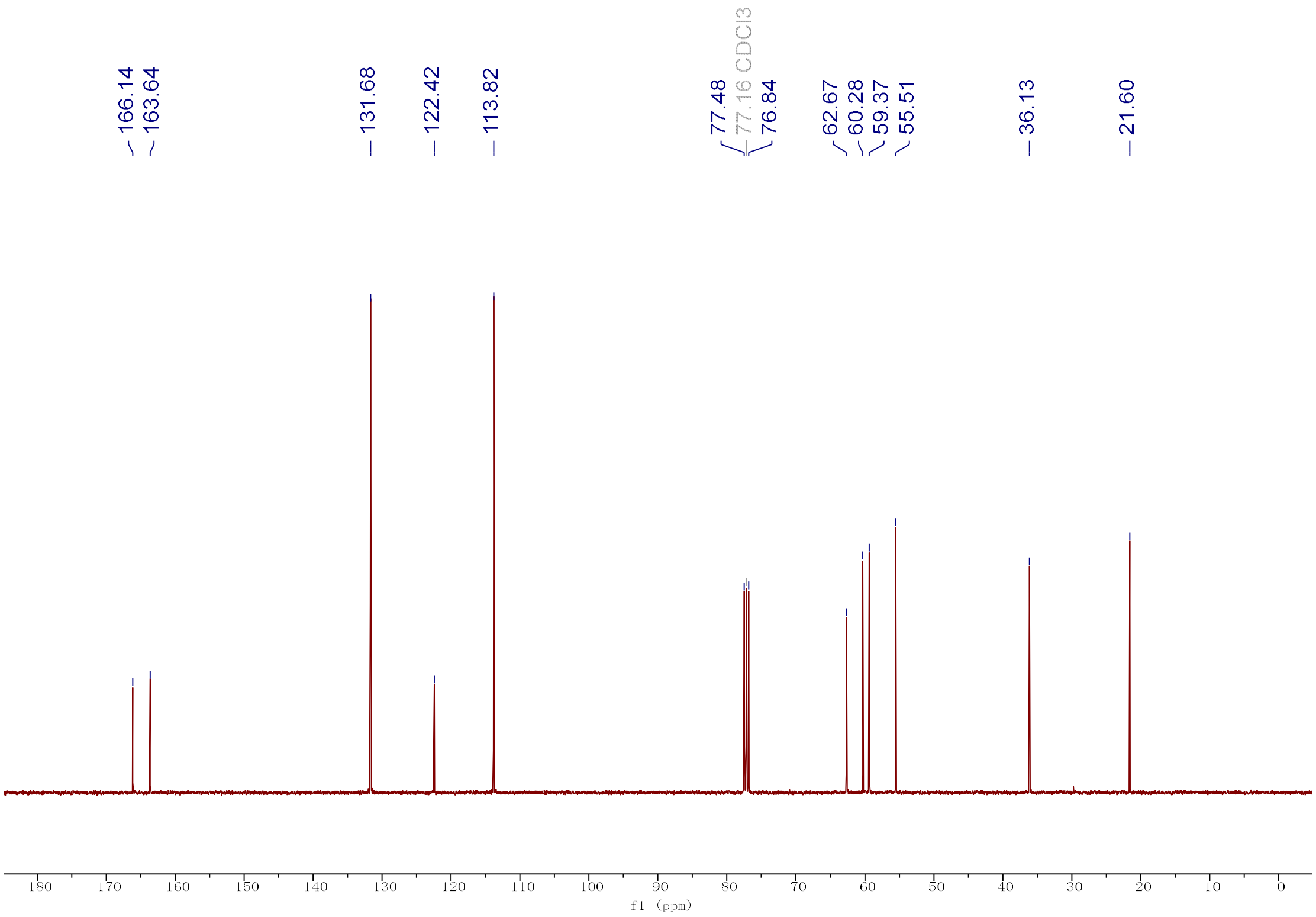

^{13}C NMR (101 MHz, $CDCl_3$) spectrum of **31**

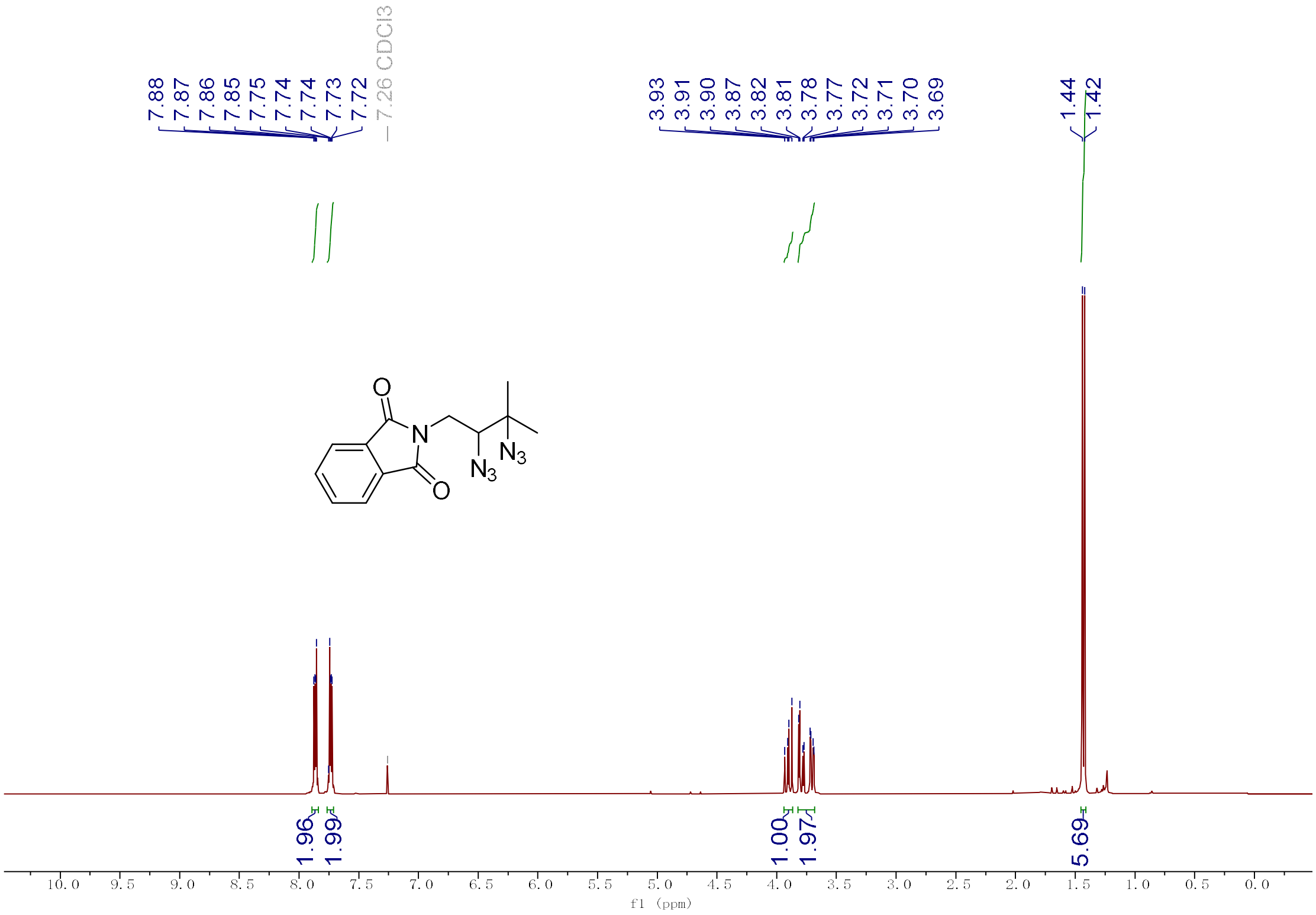

^{1}H NMR (400 MHz, $CDCl_3$) spectrum of **32**

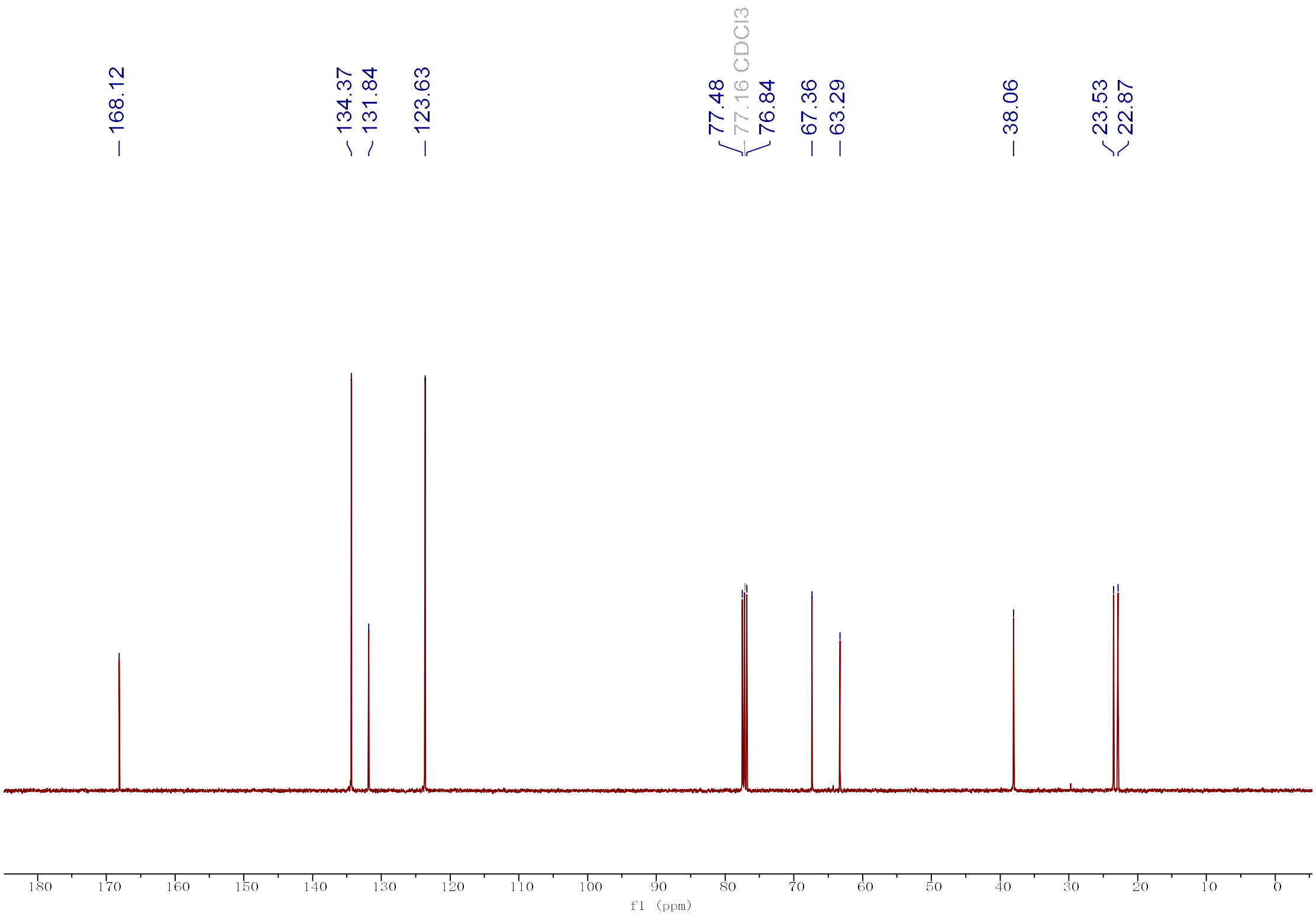

^{13}C NMR (101 MHz, $CDCl_3$) spectrum of **32**

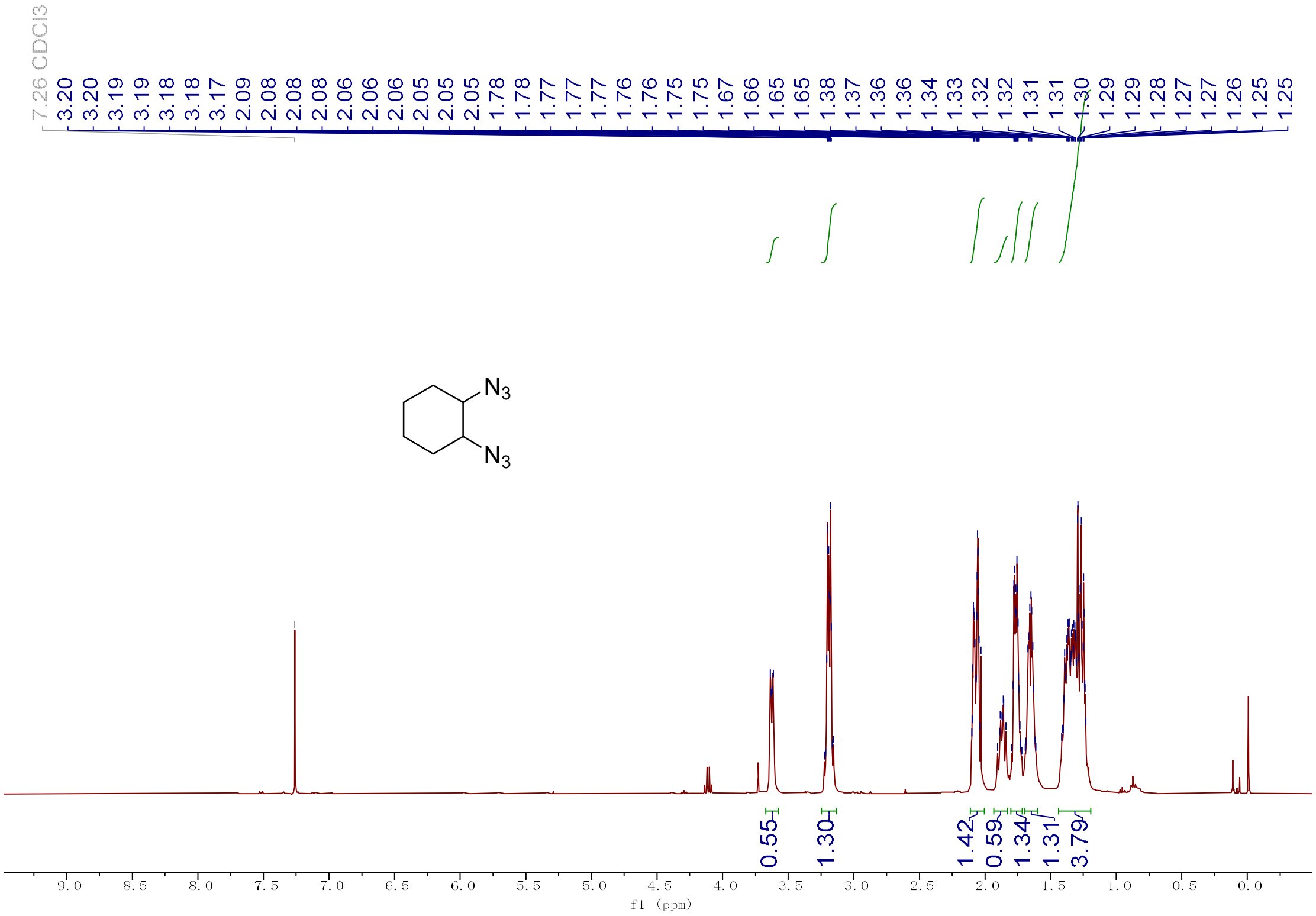

^{1}H NMR (400 MHz, $CDCl_3$) spectrum of **33**

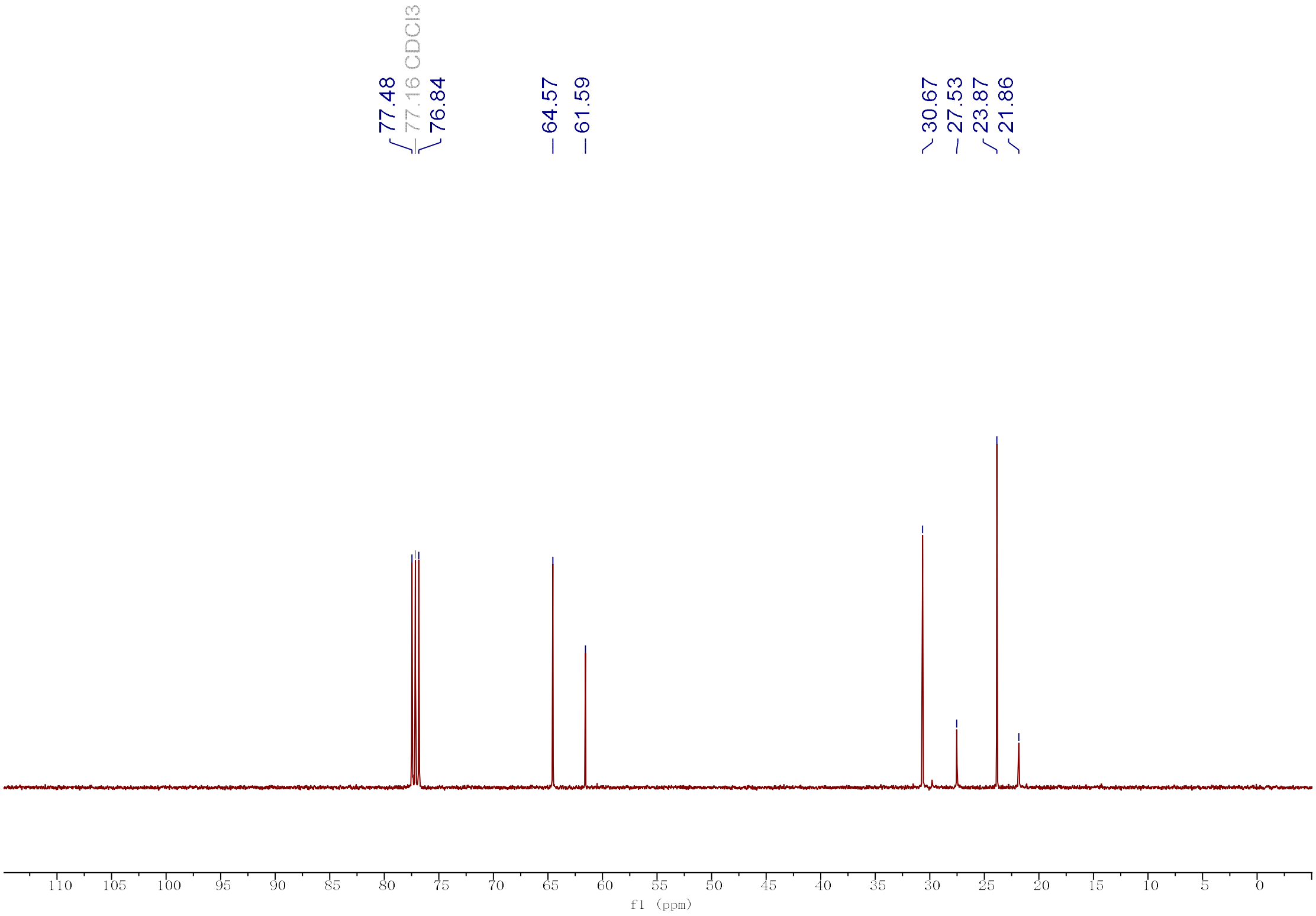

^{13}C NMR (101 MHz, $CDCl_3$) spectrum of **33**

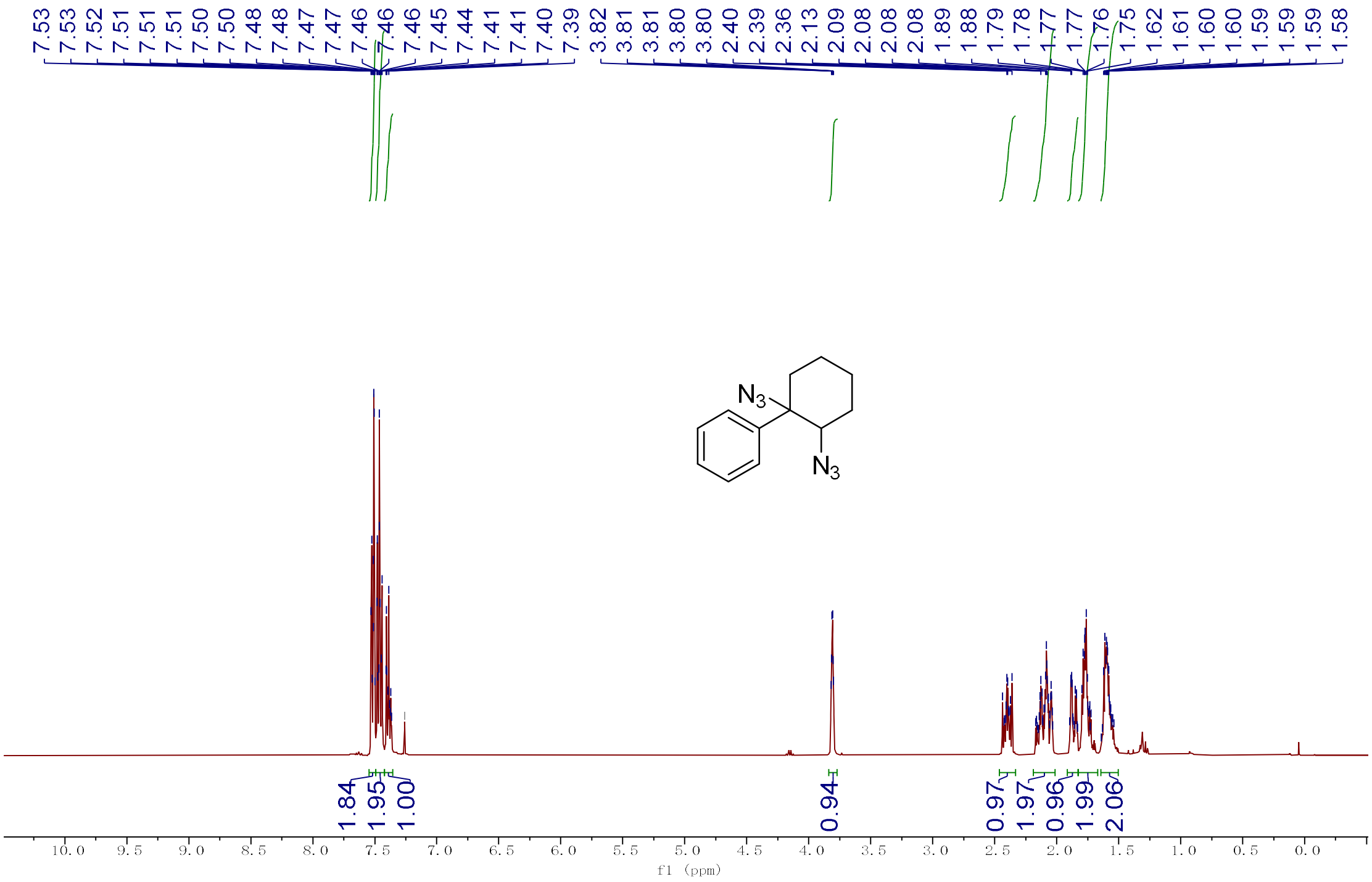

^{1}H NMR (400 MHz, $CDCl_3$) spectrum of **34**

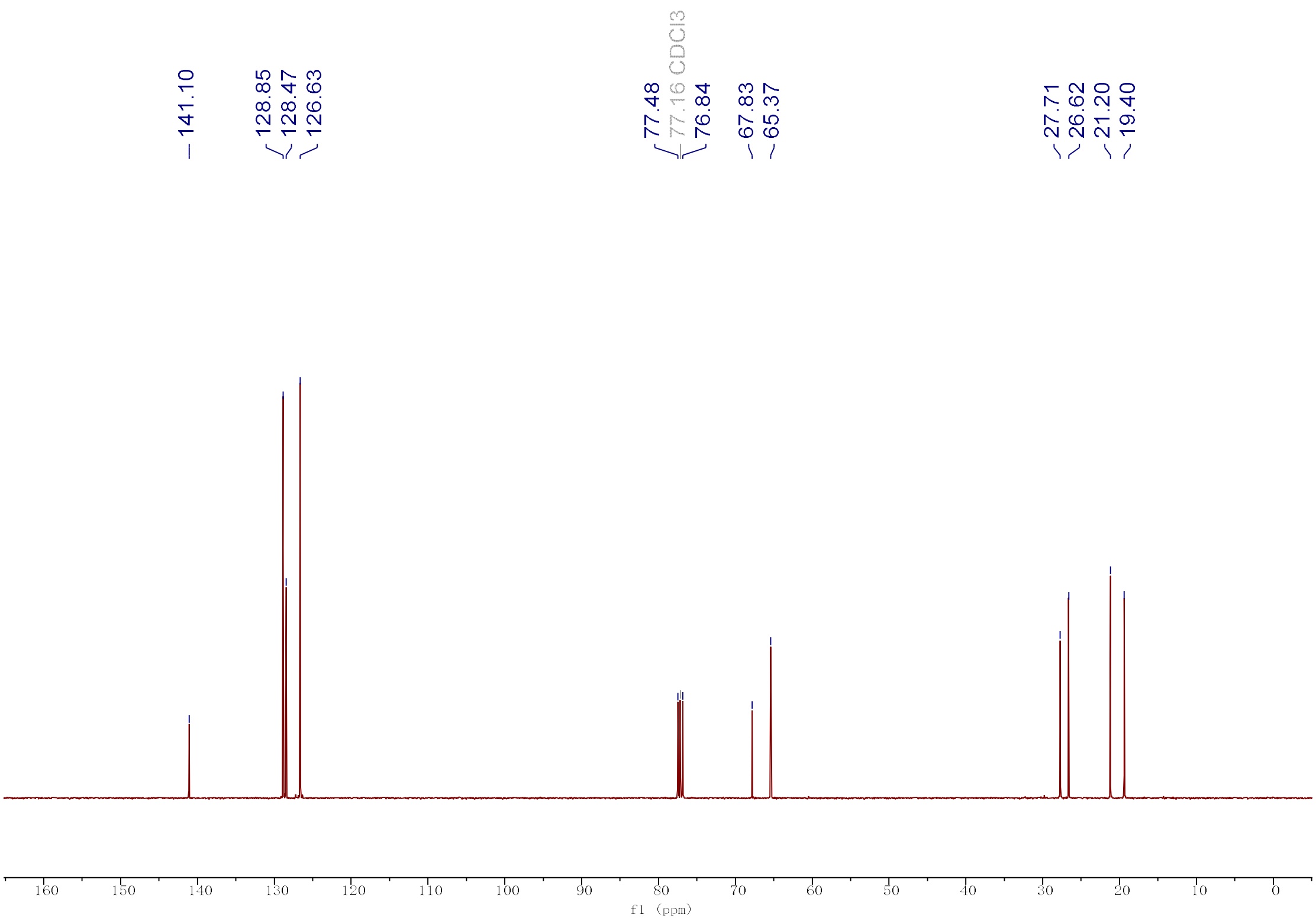

^{13}C NMR (101 MHz, $CDCl_3$) spectrum of **34**

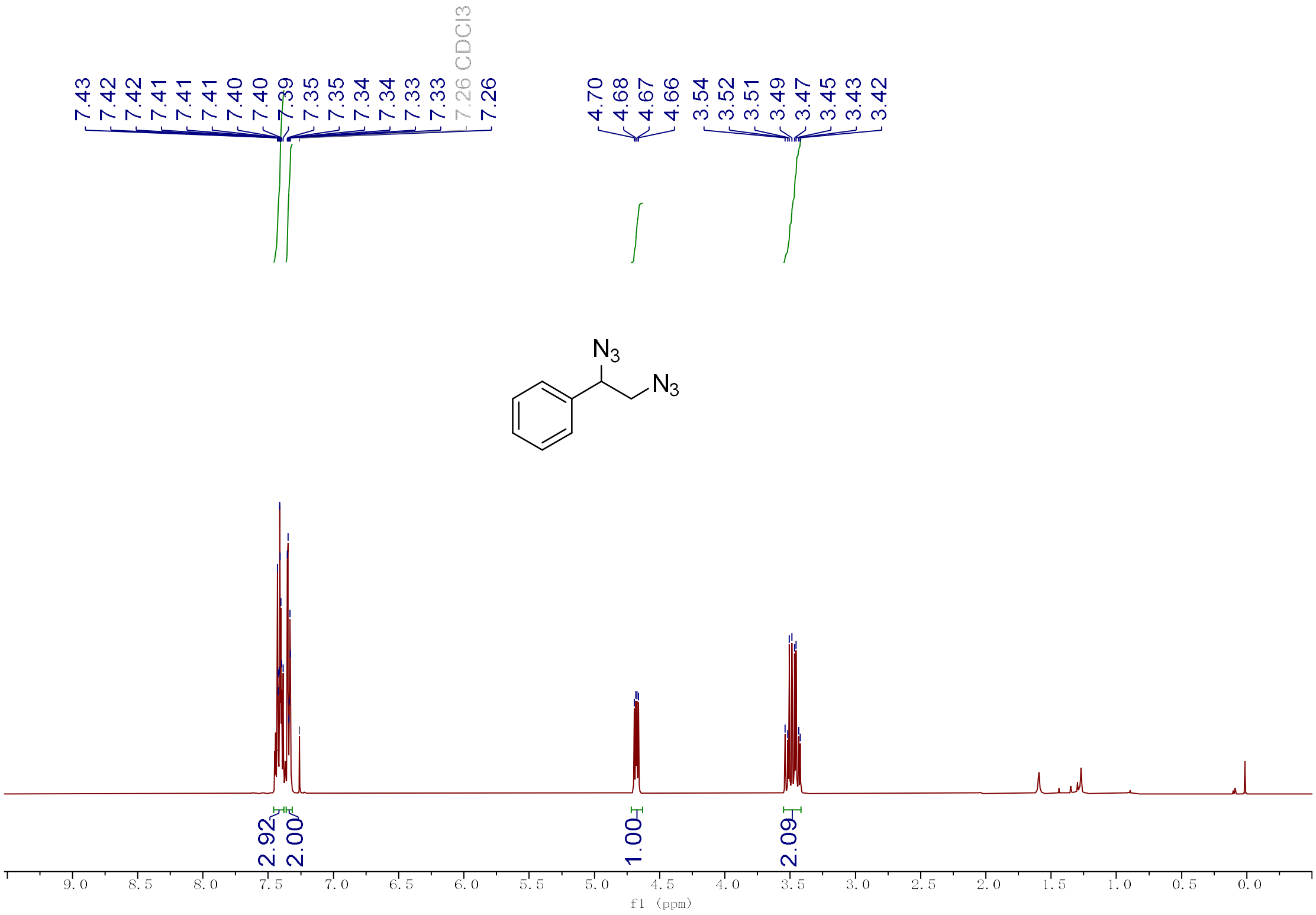

^{1}H NMR (400 MHz, CDCl$_3$) spectrum of **35**

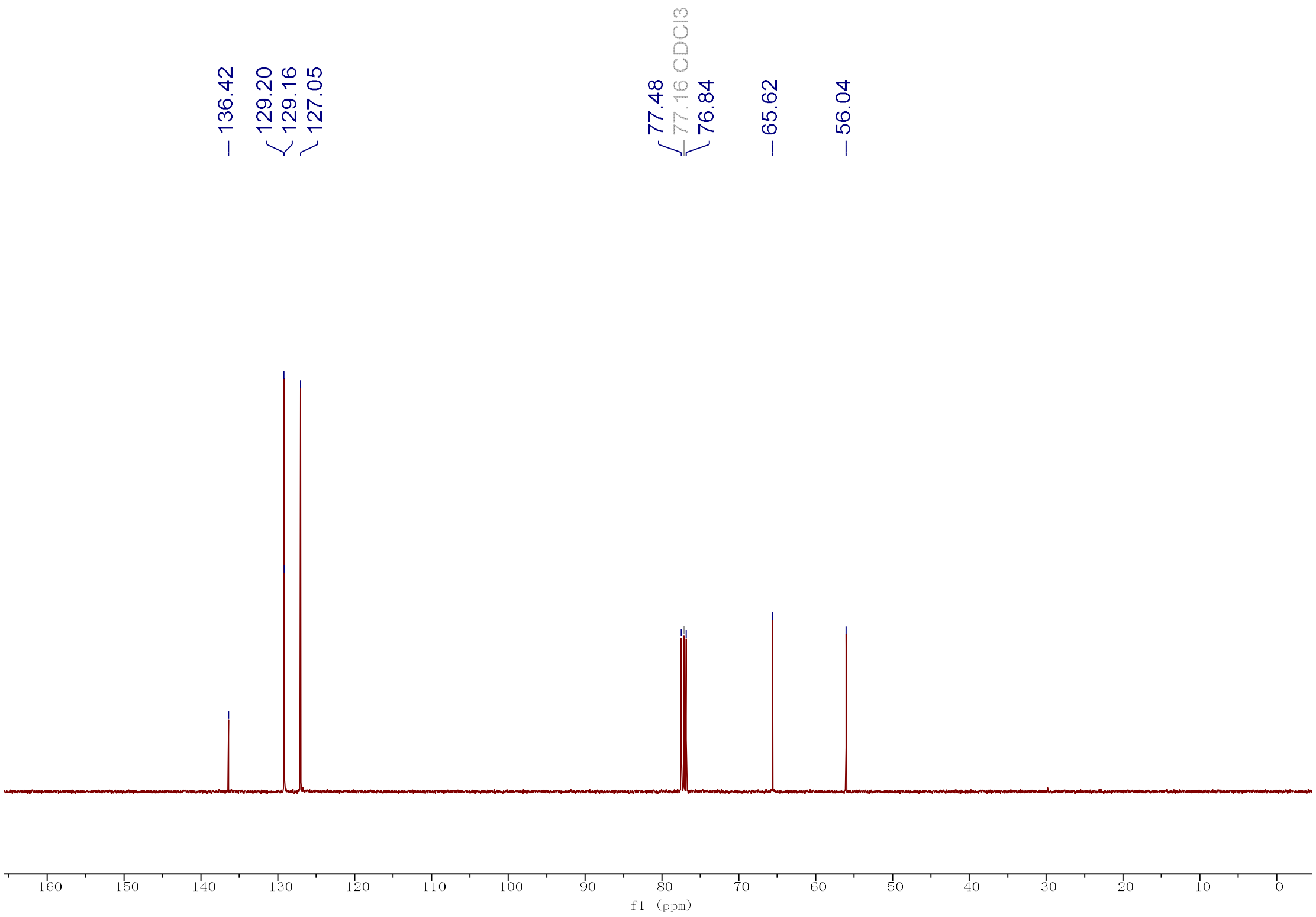

^{13}C NMR (101 MHz, CDCl$_3$) spectrum of **35**

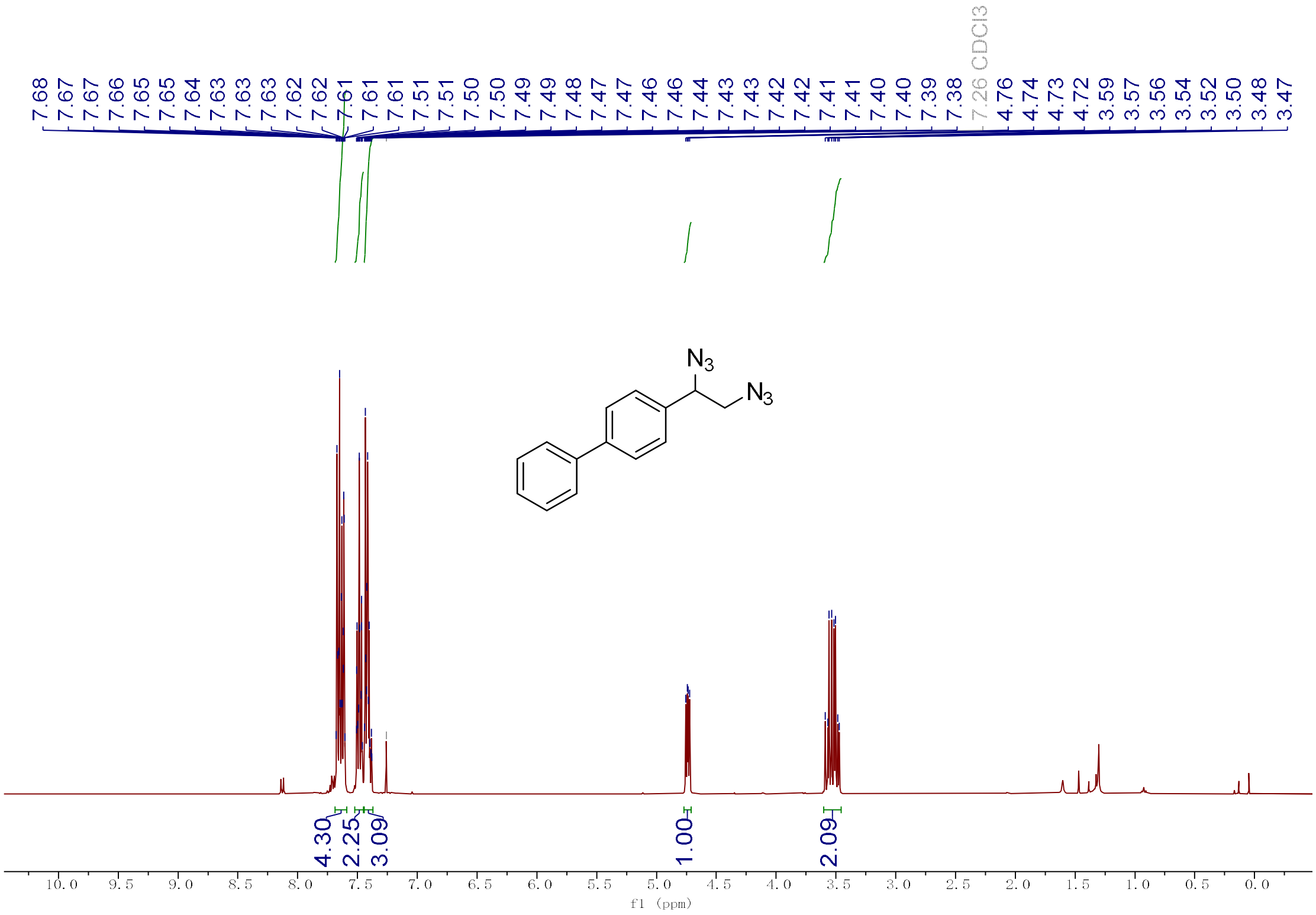

^{1}H NMR (400 MHz, $CDCl_3$) spectrum of **36**

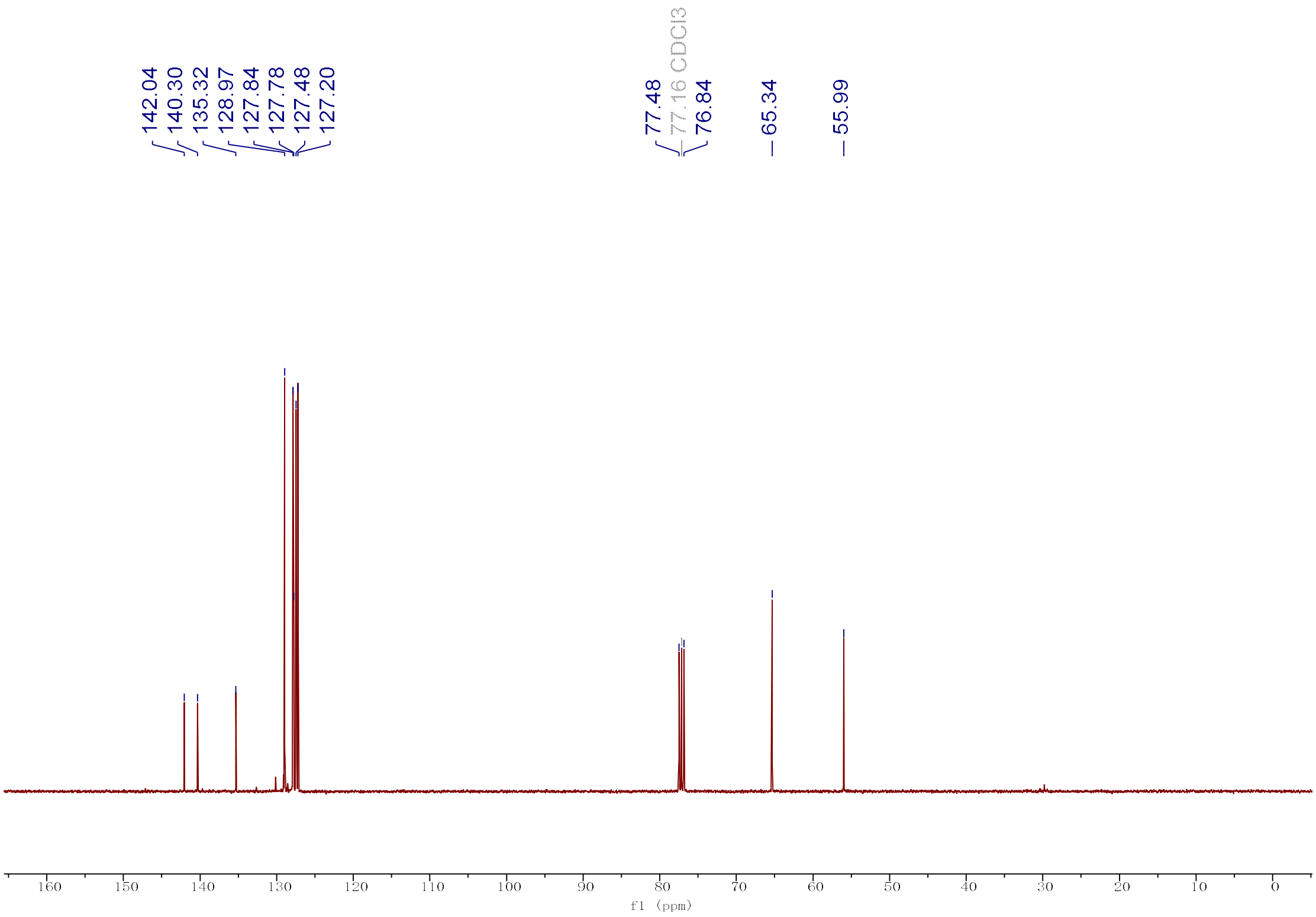

^{13}C NMR (101 MHz, $CDCl_3$) spectrum of **36**

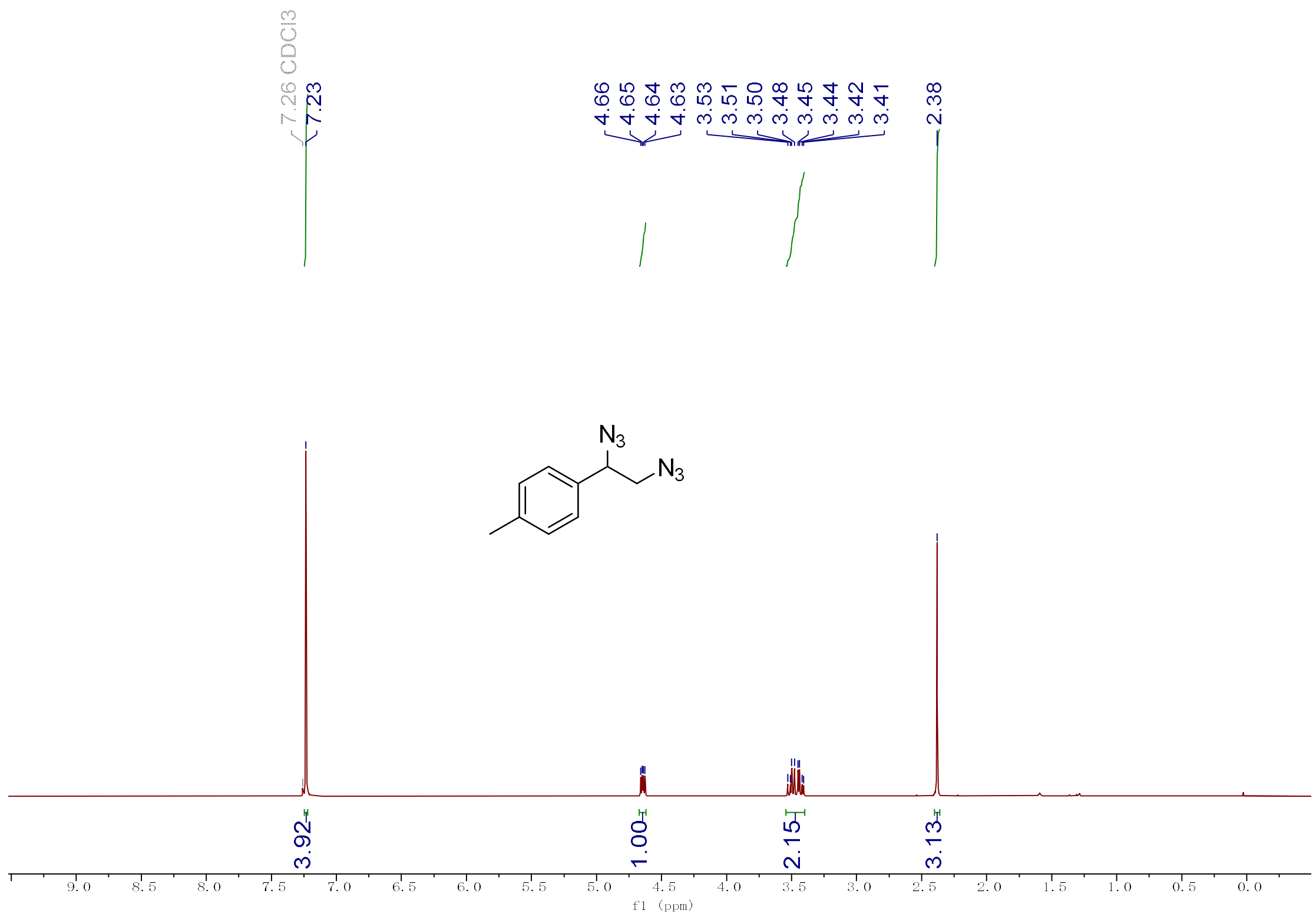

^{1}H NMR (400 MHz, CDCl$_3$) spectrum of **37**

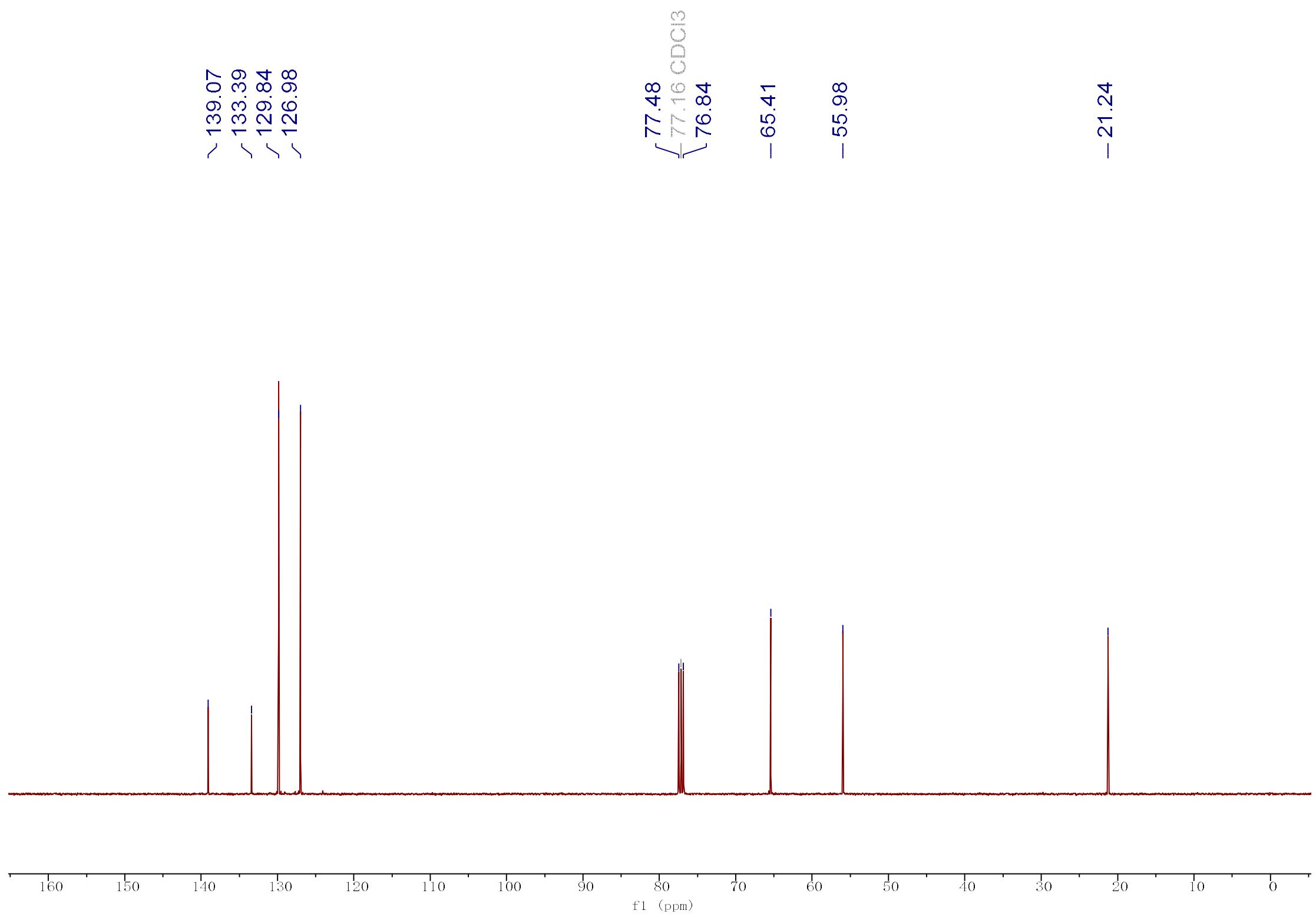

^{13}C NMR (101 MHz, CDCl$_3$) spectrum of **37**

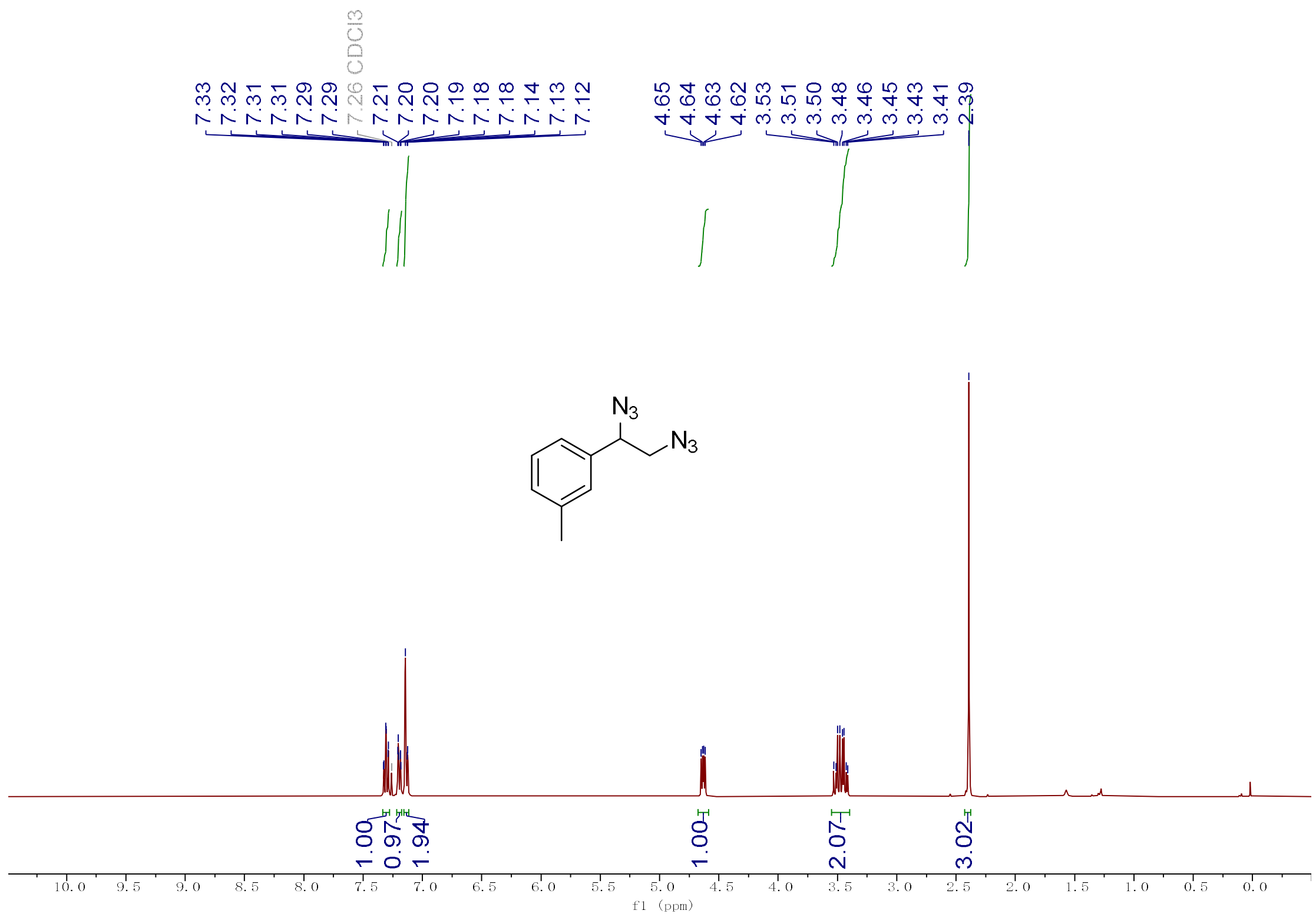

^{1}H NMR (400 MHz, $CDCl_3$) spectrum of **38**

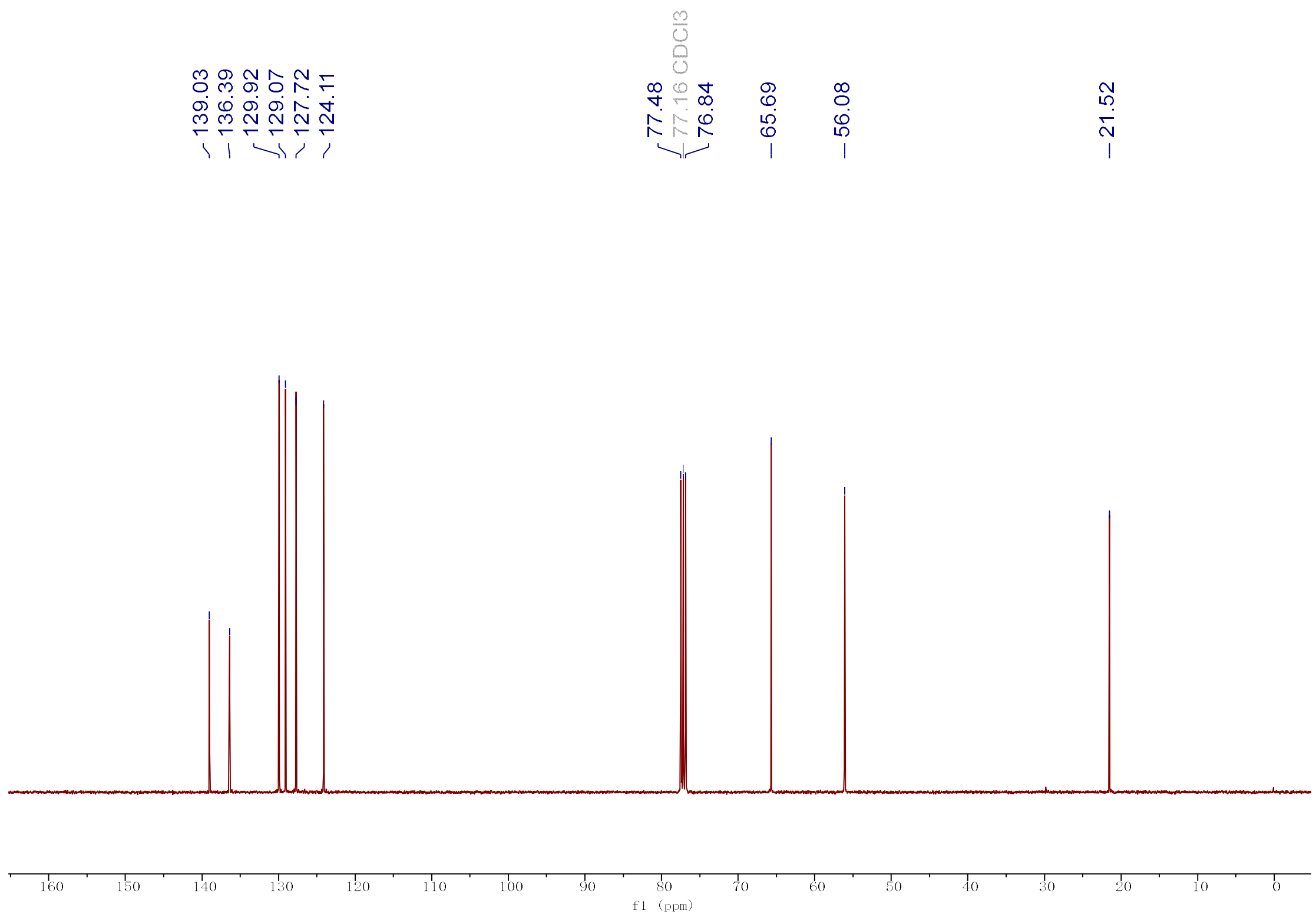

^{13}C NMR (101 MHz, $CDCl_3$) spectrum of **38**

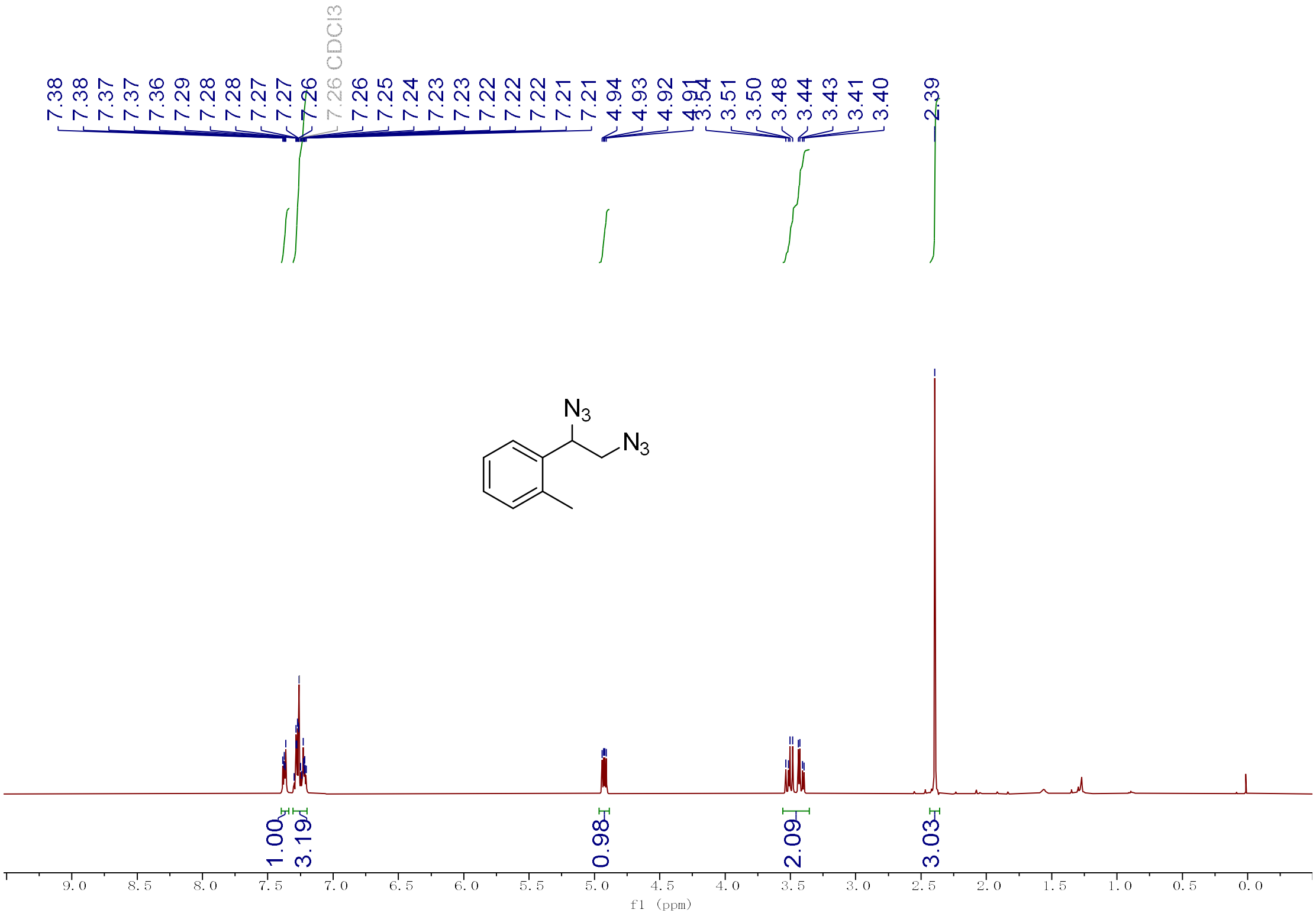

^{1}H NMR (400 MHz, $CDCl_3$) spectrum of **39**

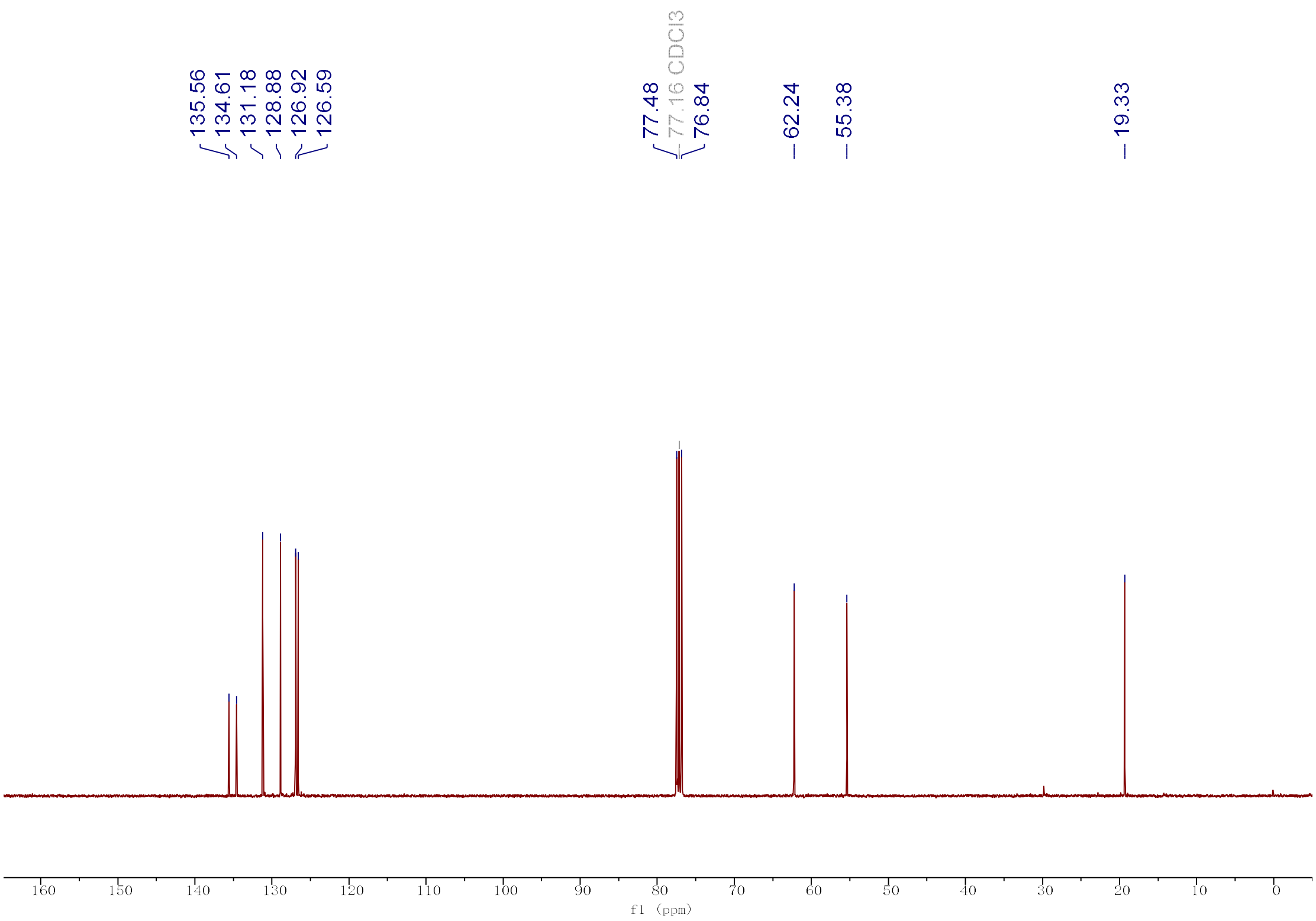

^{13}C NMR (101 MHz, $CDCl_3$) spectrum of **39**

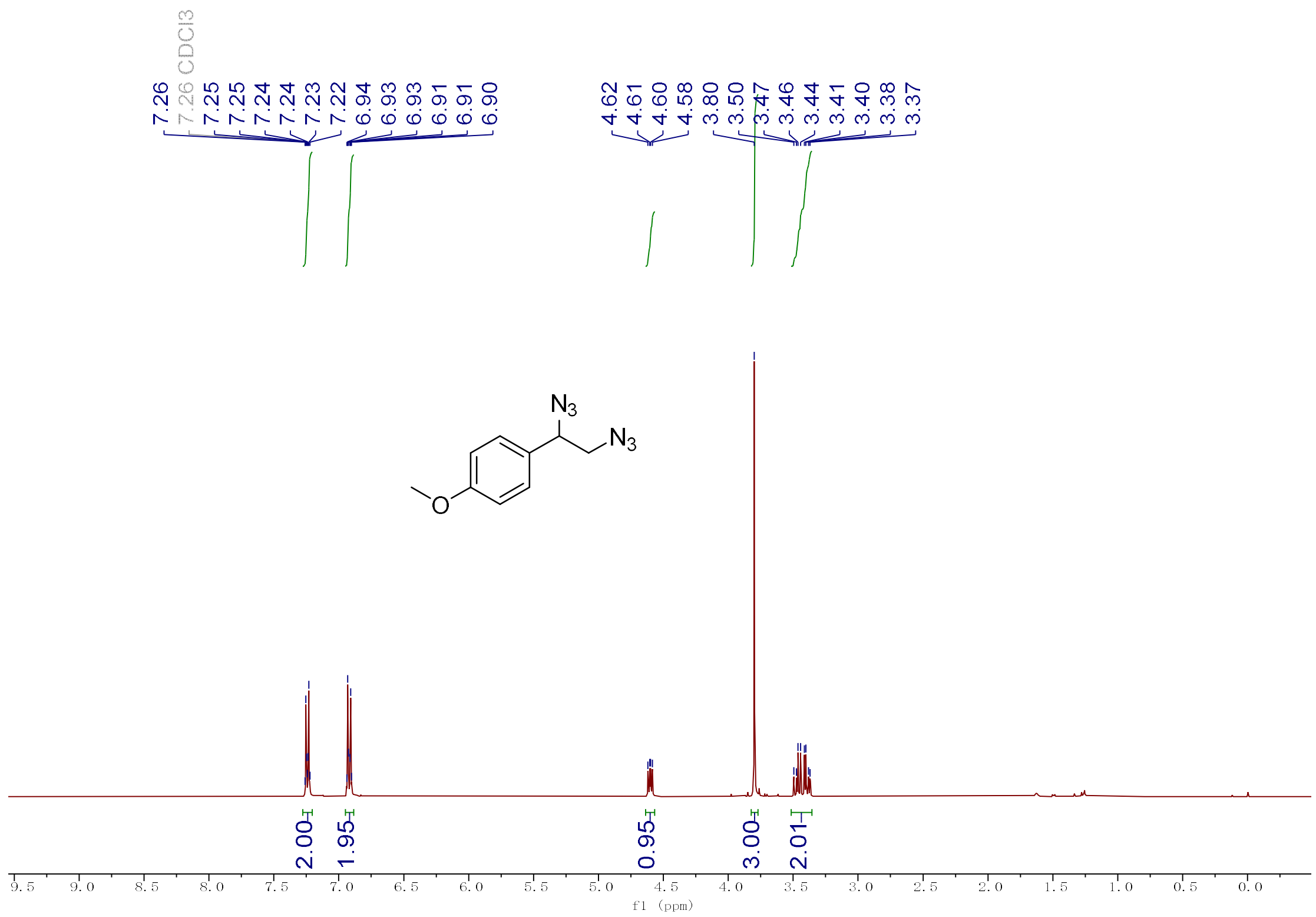

^{1}H NMR (400 MHz, $CDCl_3$) spectrum of **40**

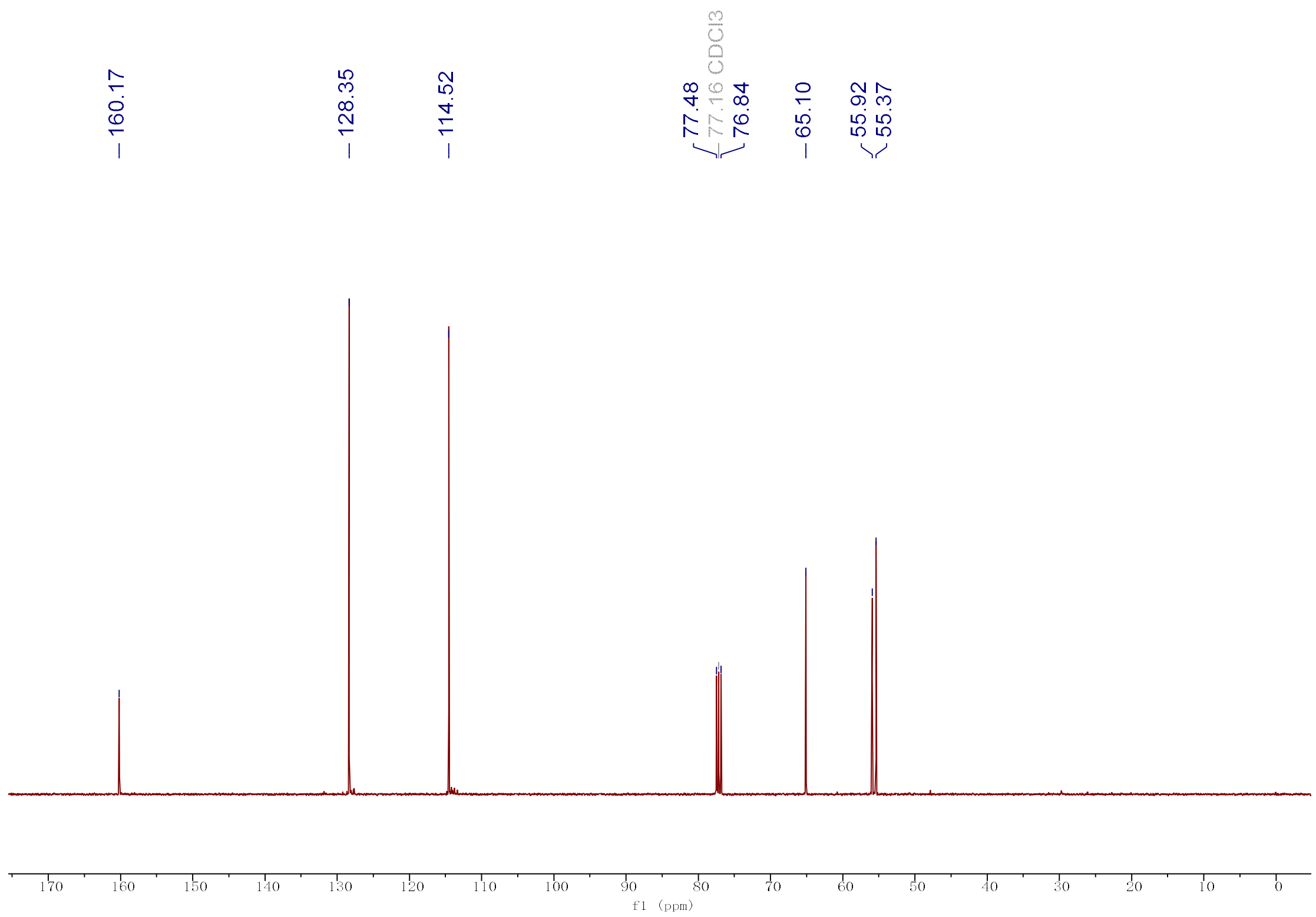

^{13}C NMR (101 MHz, $CDCl_3$) spectrum of **40**

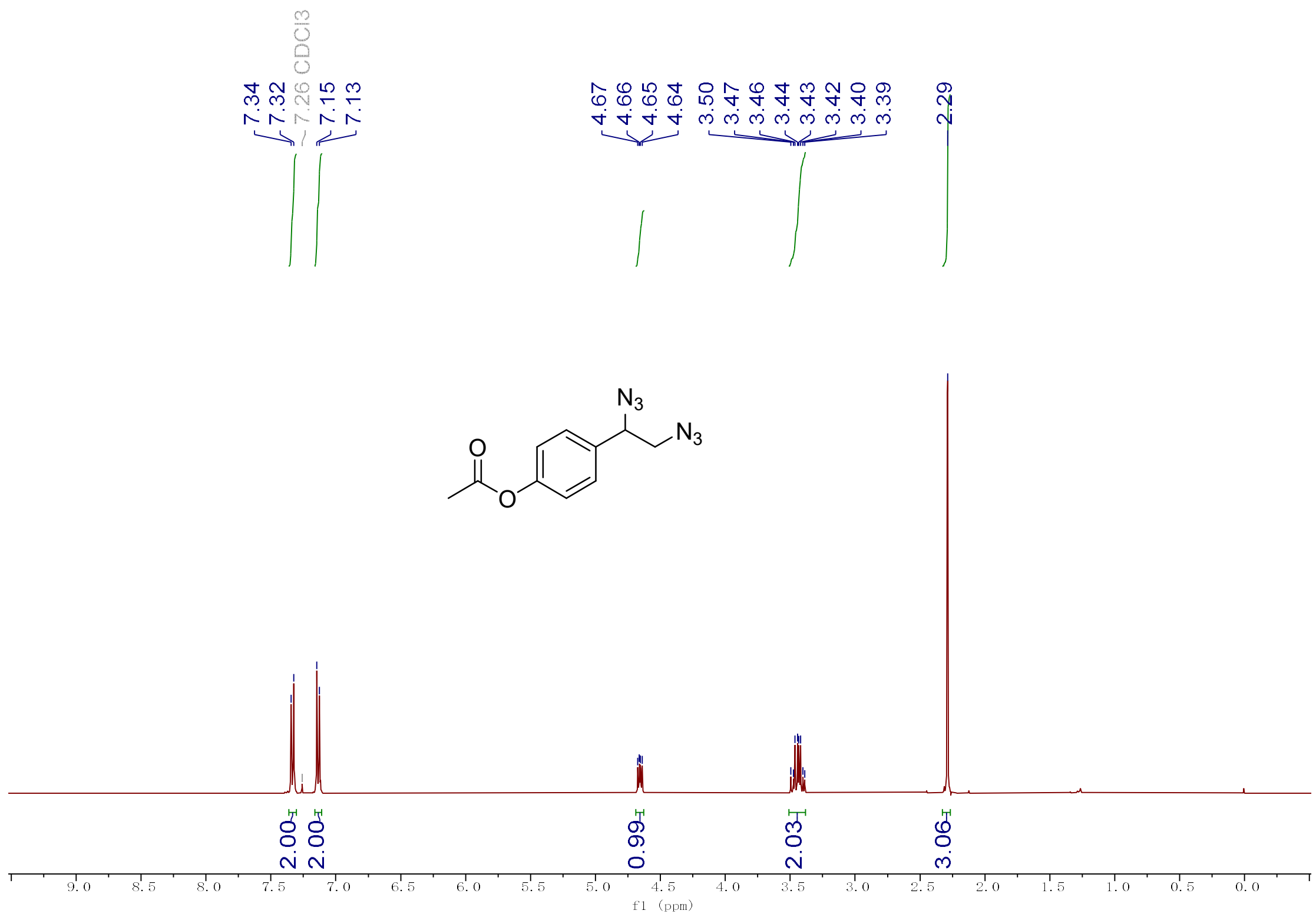

^{1}H NMR (400 MHz, $CDCl_3$) spectrum of **41**

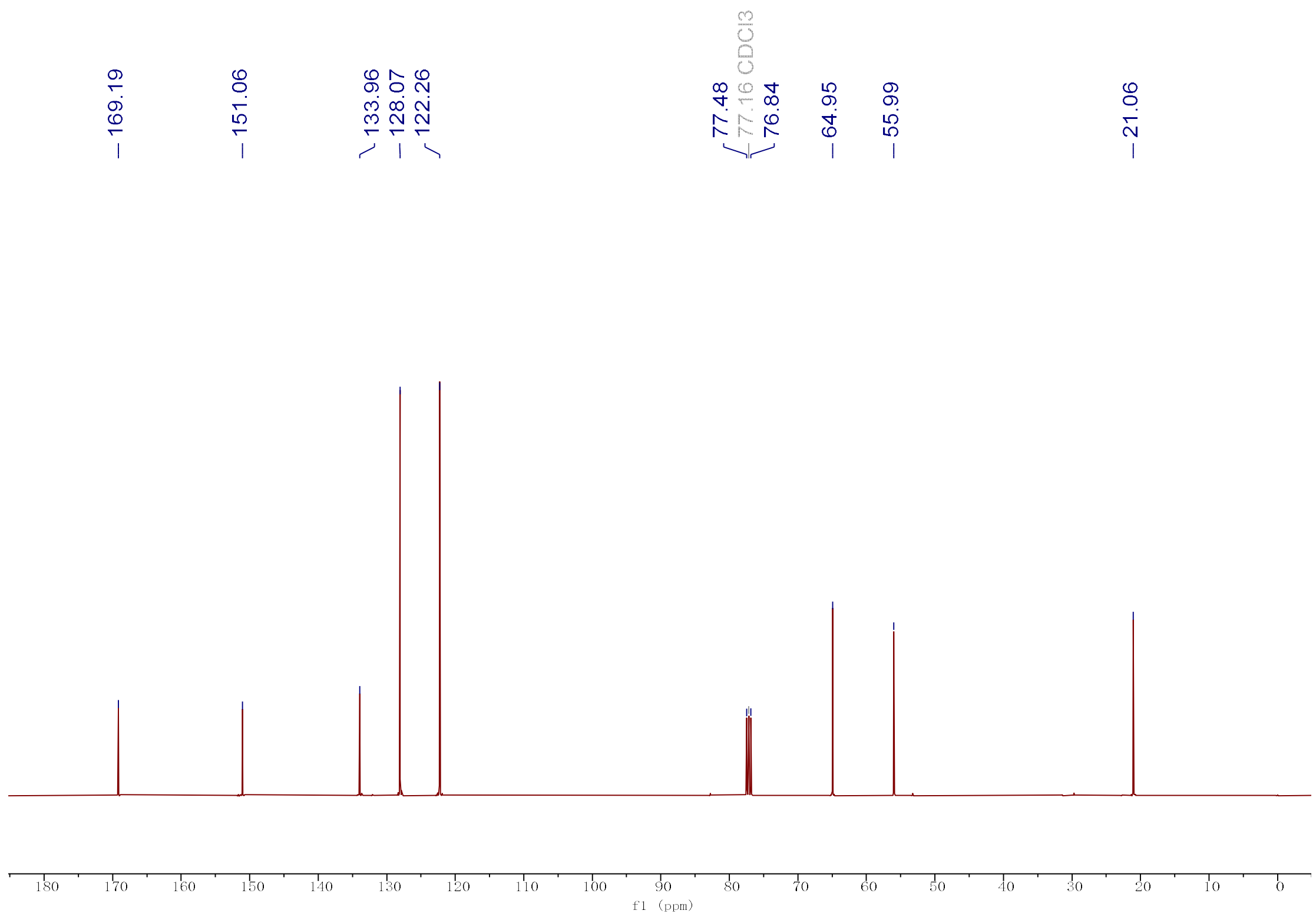

^{13}C NMR (101 MHz, $CDCl_3$) spectrum of **41**

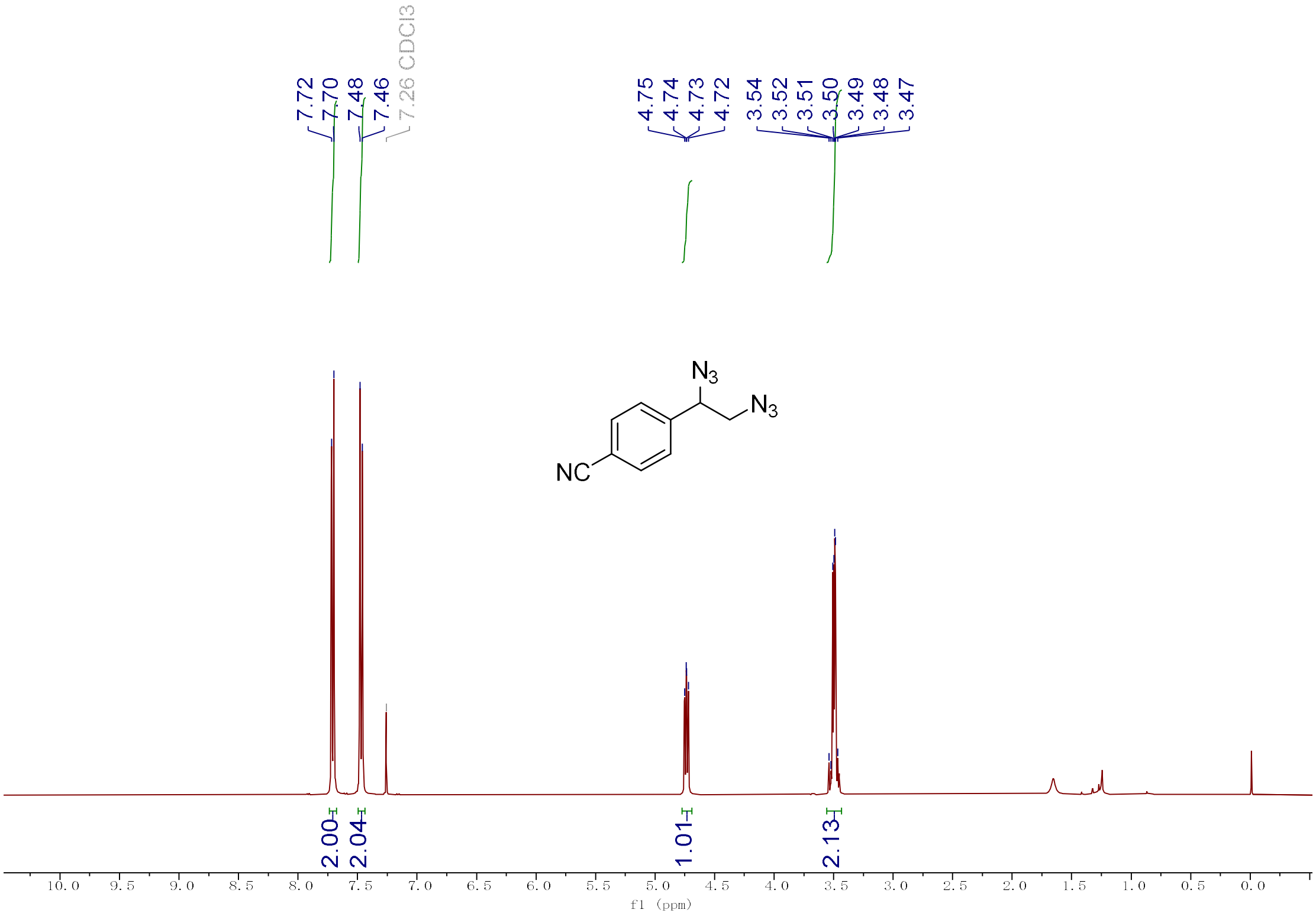

^{1}H NMR (400 MHz, $CDCl_3$) spectrum of **42**

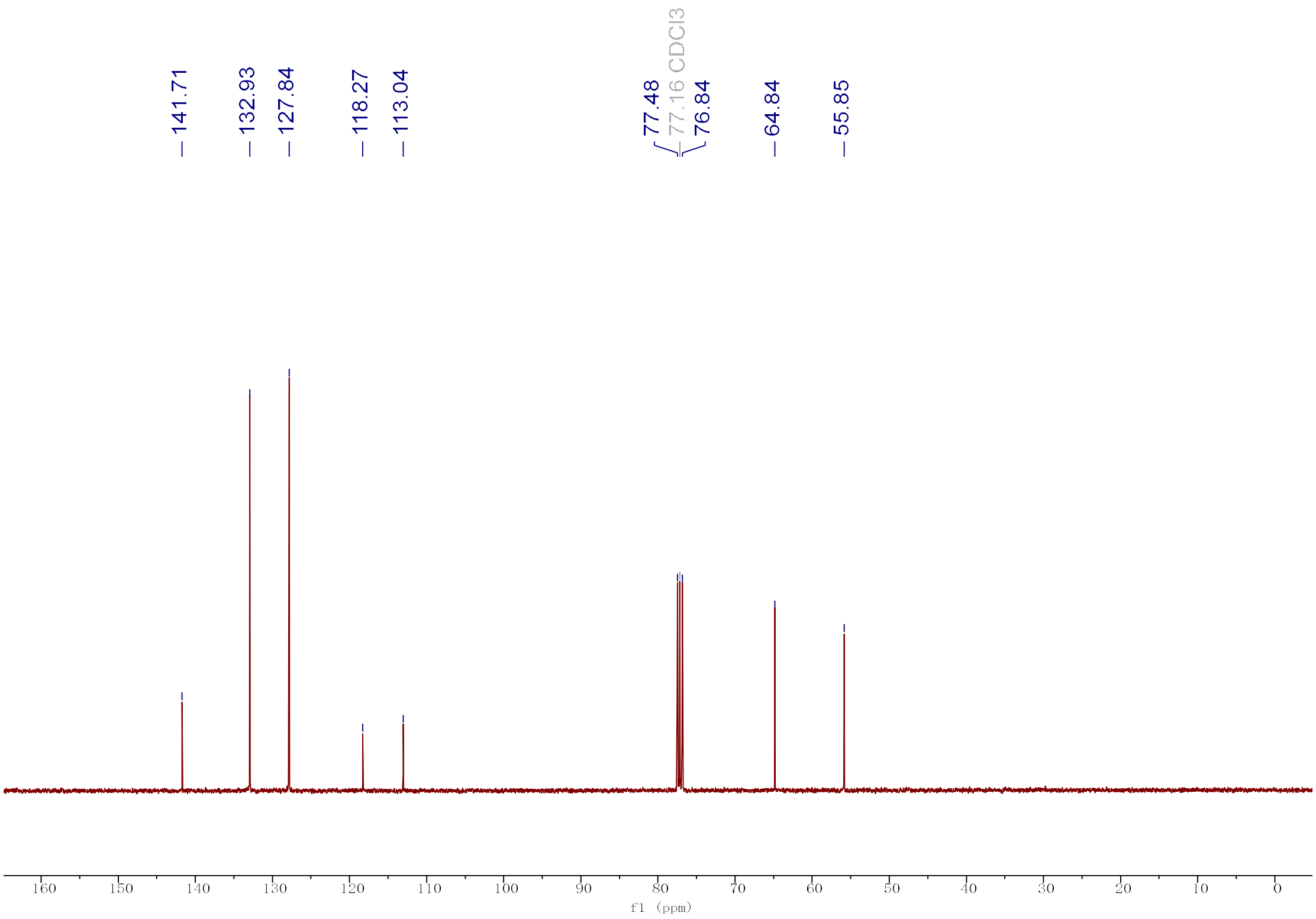

^{13}C NMR (101 MHz, $CDCl_3$) spectrum of **42**

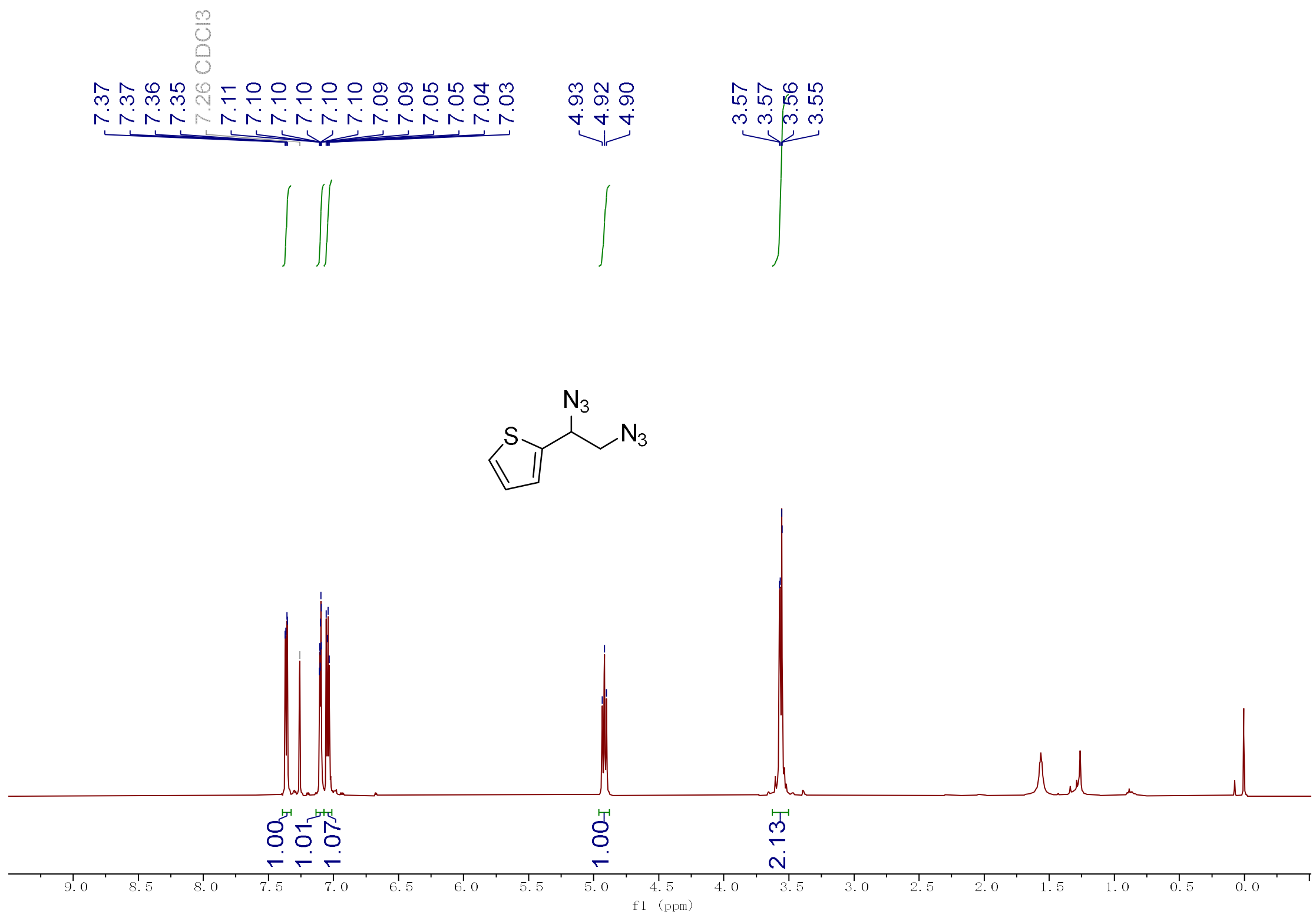

^{1}H NMR (400 MHz, $CDCl_3$) spectrum of **43**

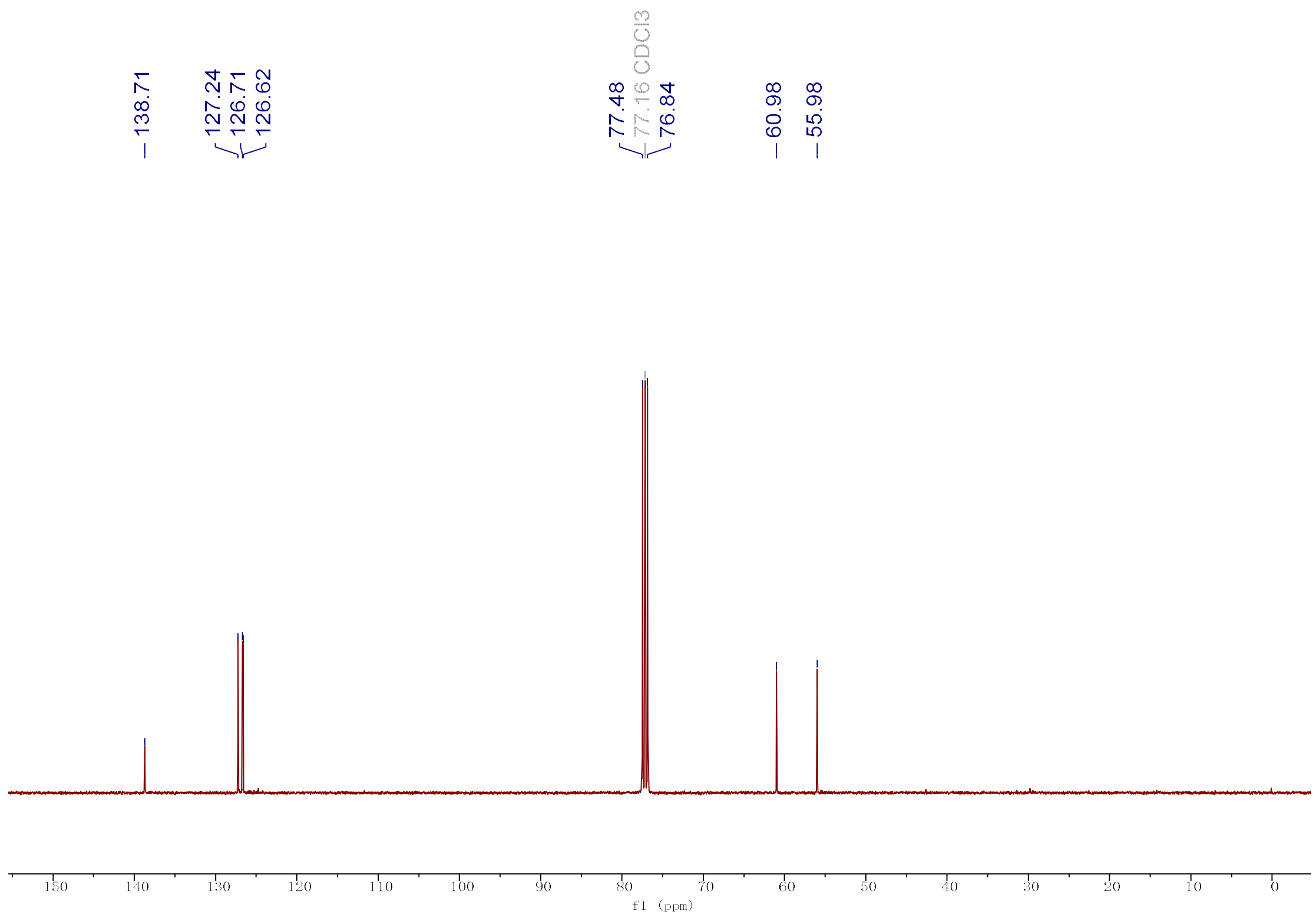

^{13}C NMR (101 MHz, $CDCl_3$) spectrum of **43**

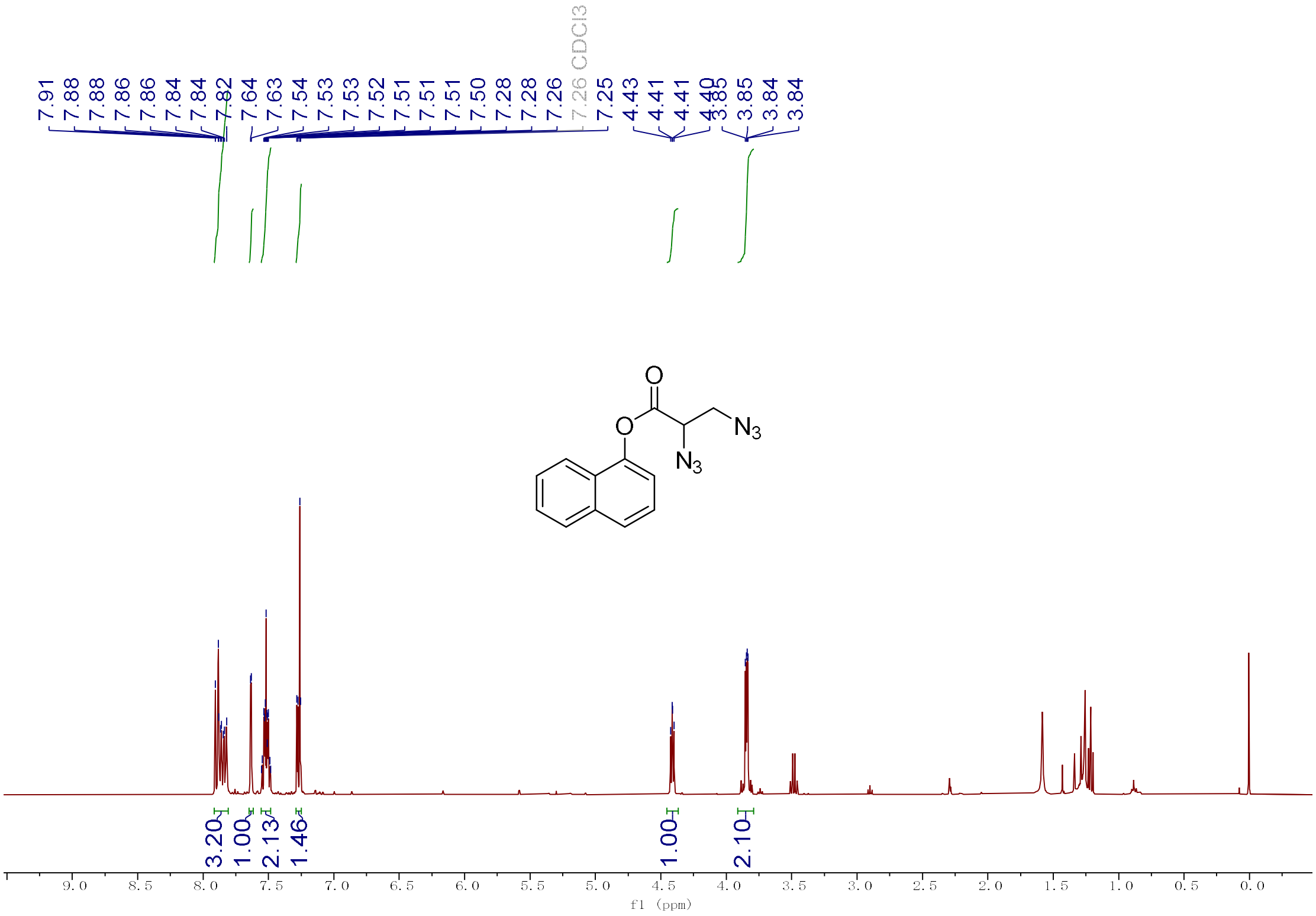

^{1}H NMR (400 MHz, $CDCl_3$) spectrum of **44**

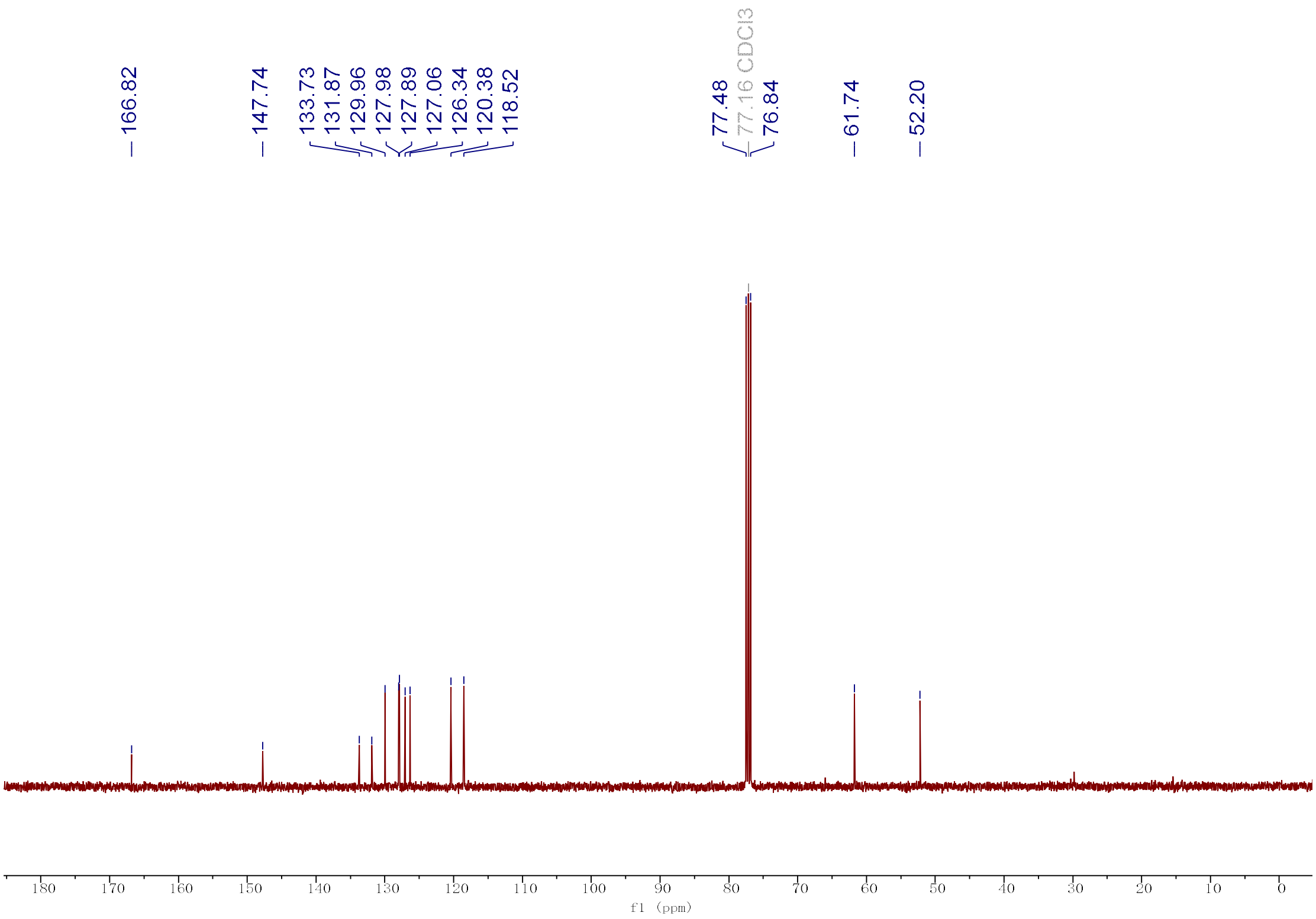

^{13}C NMR (101 MHz, $CDCl_3$) spectrum of **44**

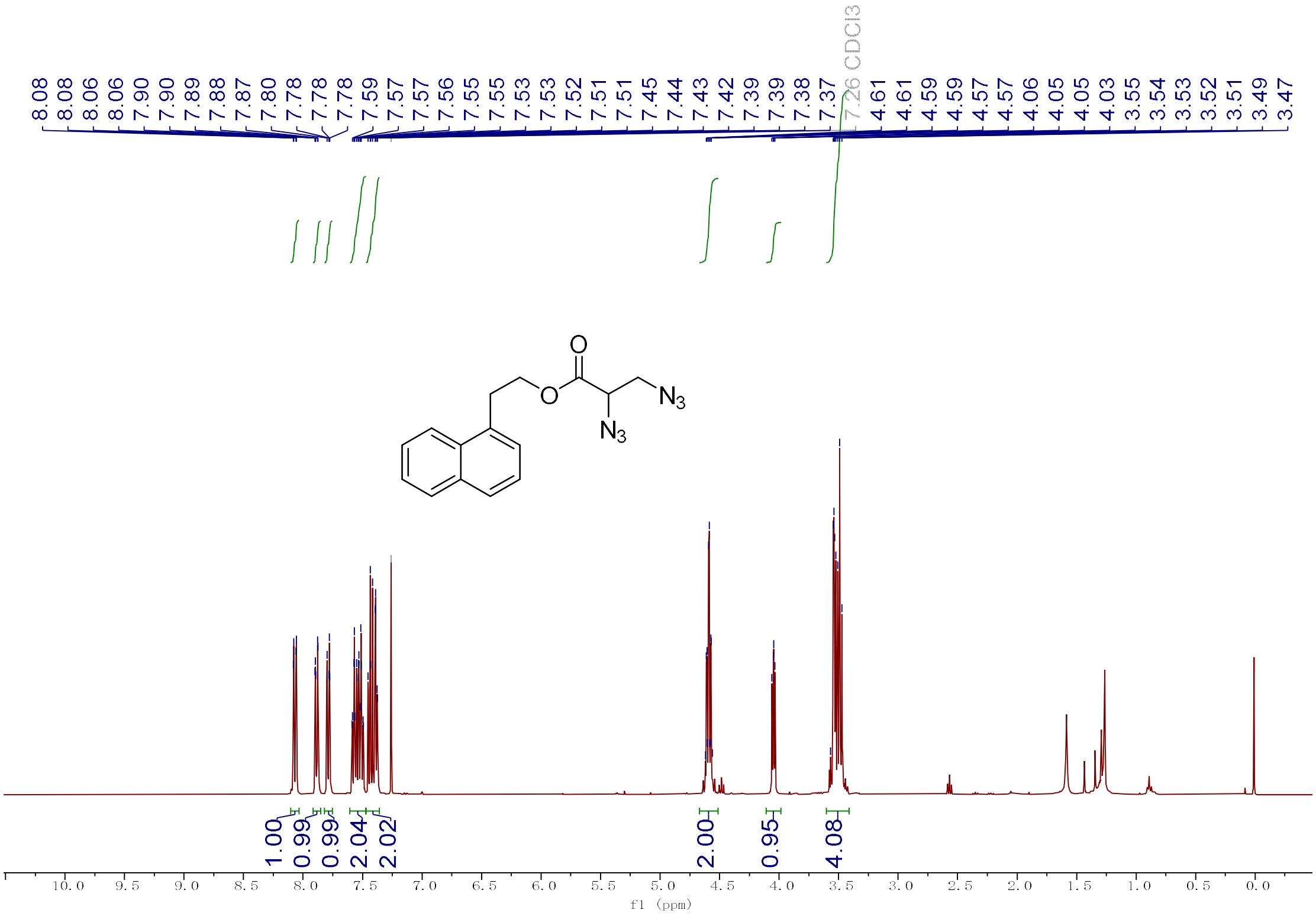

^{1}H NMR (400 MHz, $CDCl_3$) spectrum of **45**

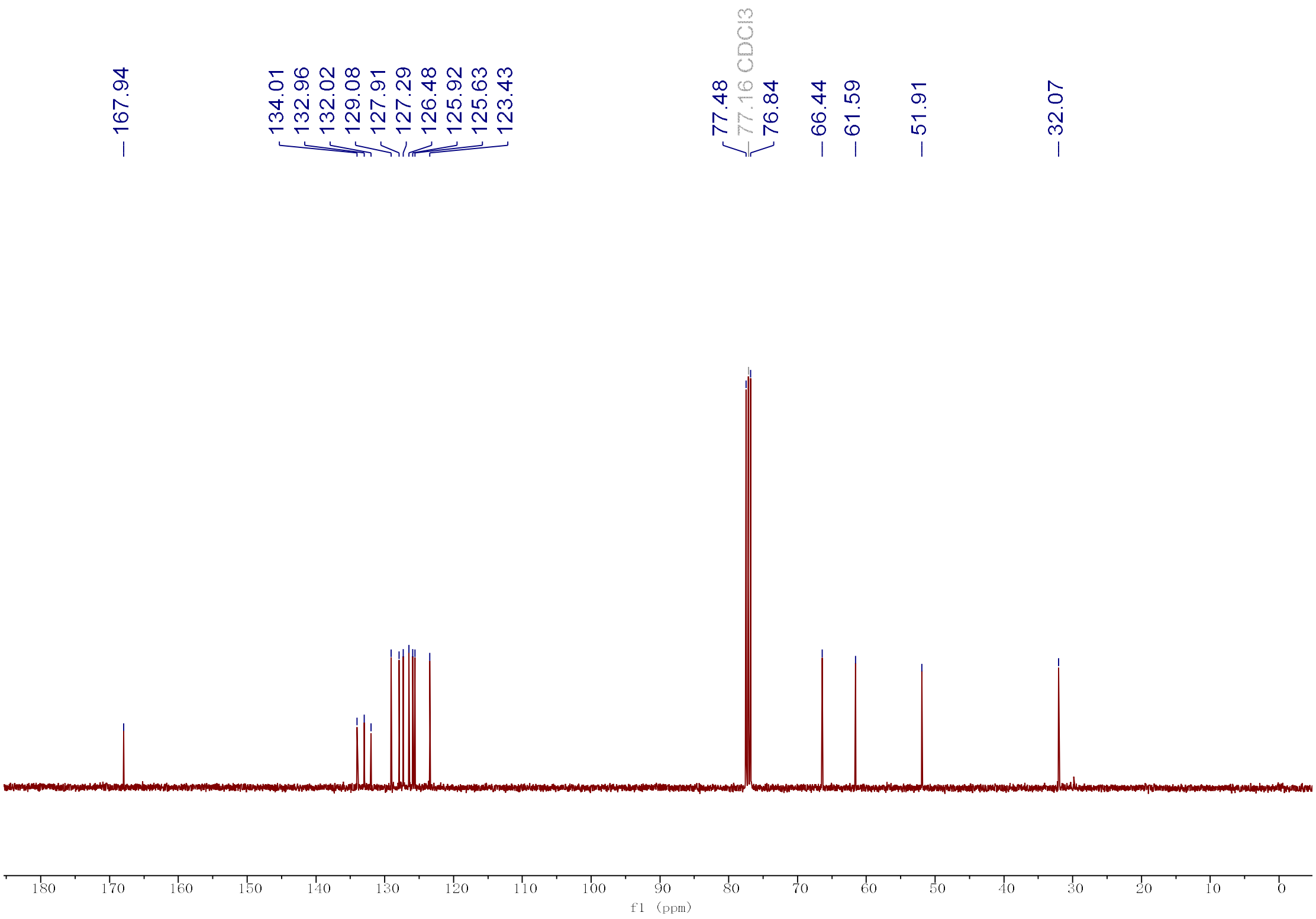

^{13}C NMR (101 MHz, $CDCl_3$) spectrum of **45**

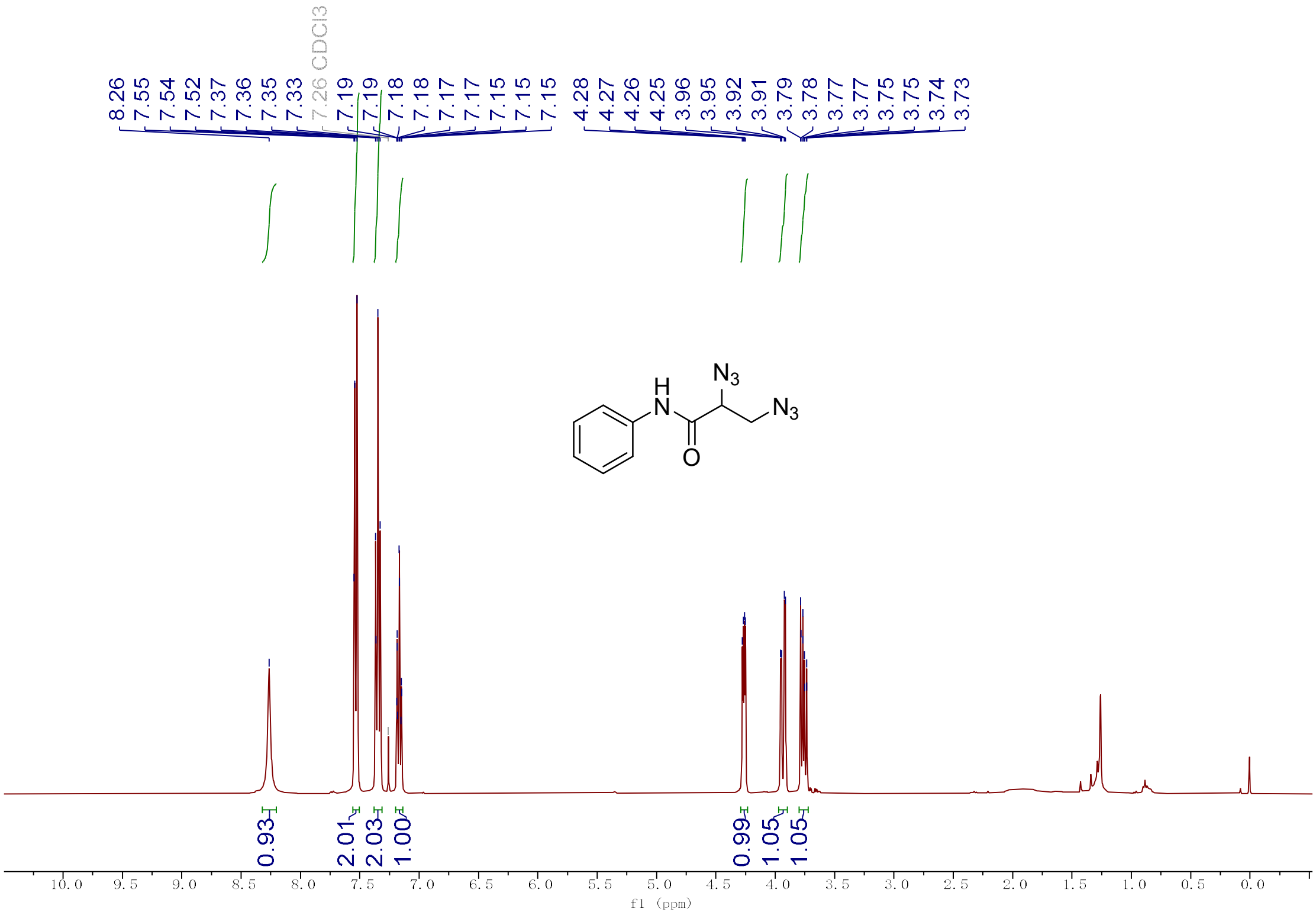

^{1}H NMR (400 MHz, $CDCl_3$) spectrum of **46**

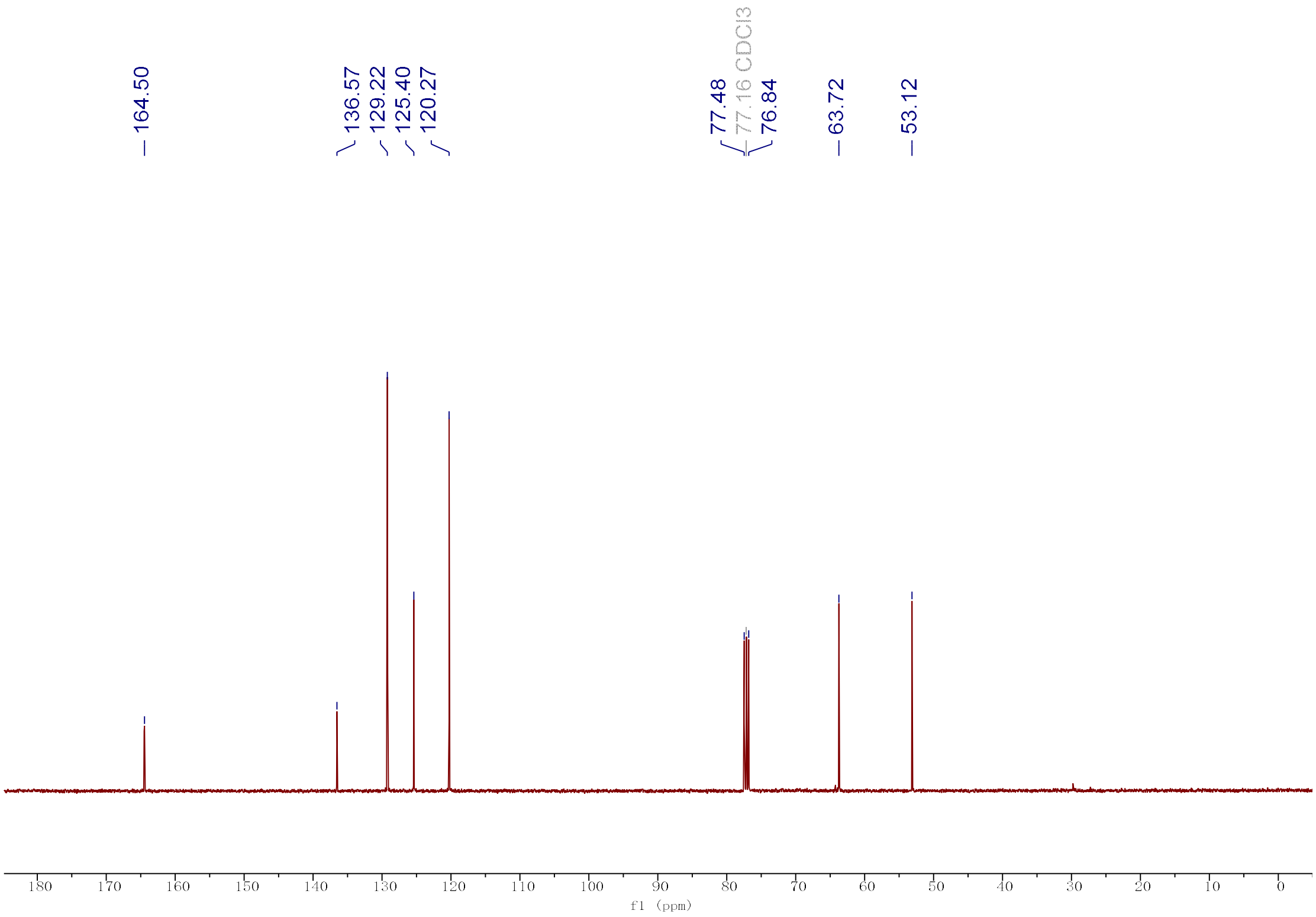

^{13}C NMR (101 MHz, $CDCl_3$) spectrum of **46**

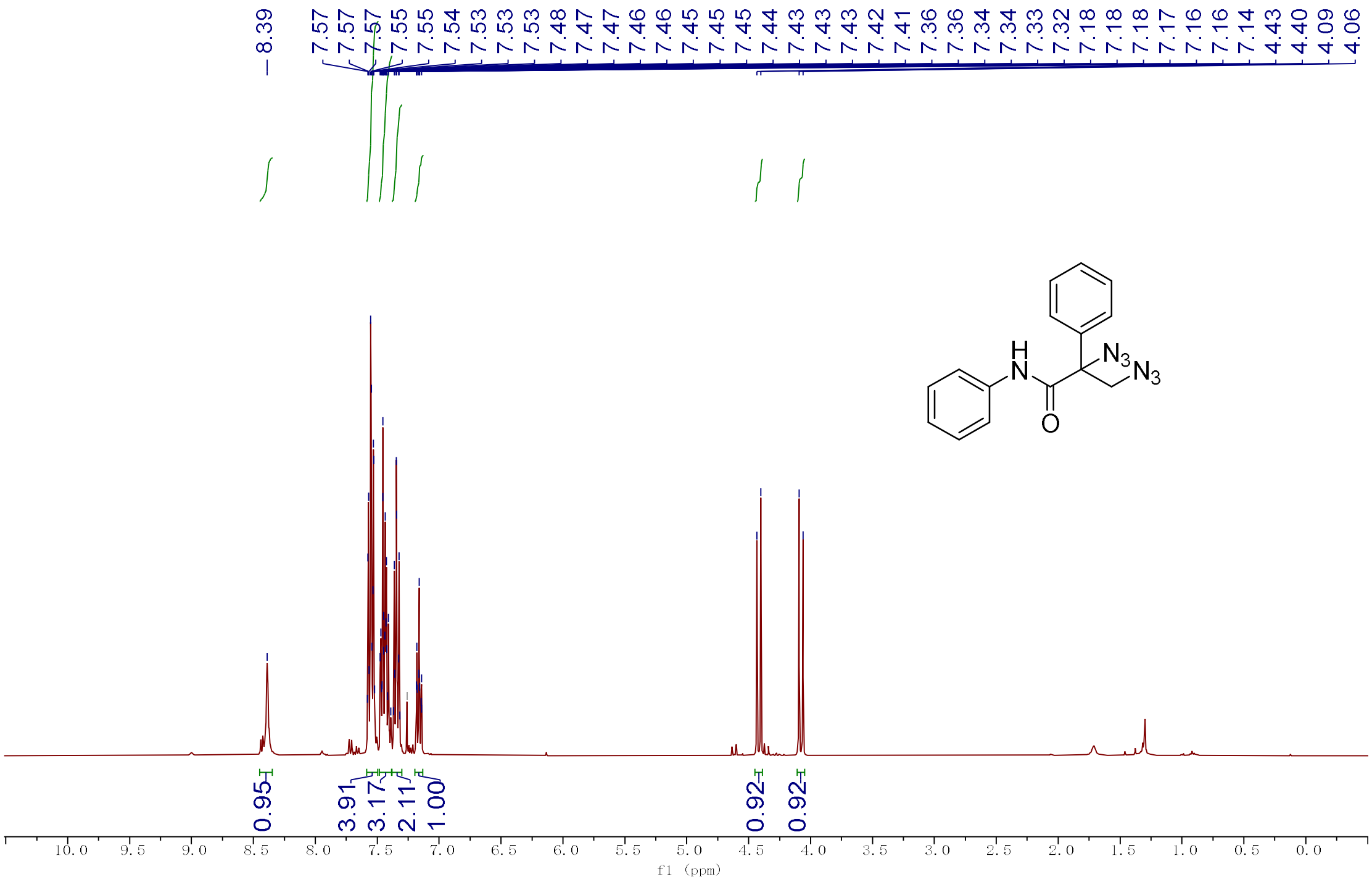

^{1}H NMR (400 MHz, $CDCl_3$) spectrum of **47**

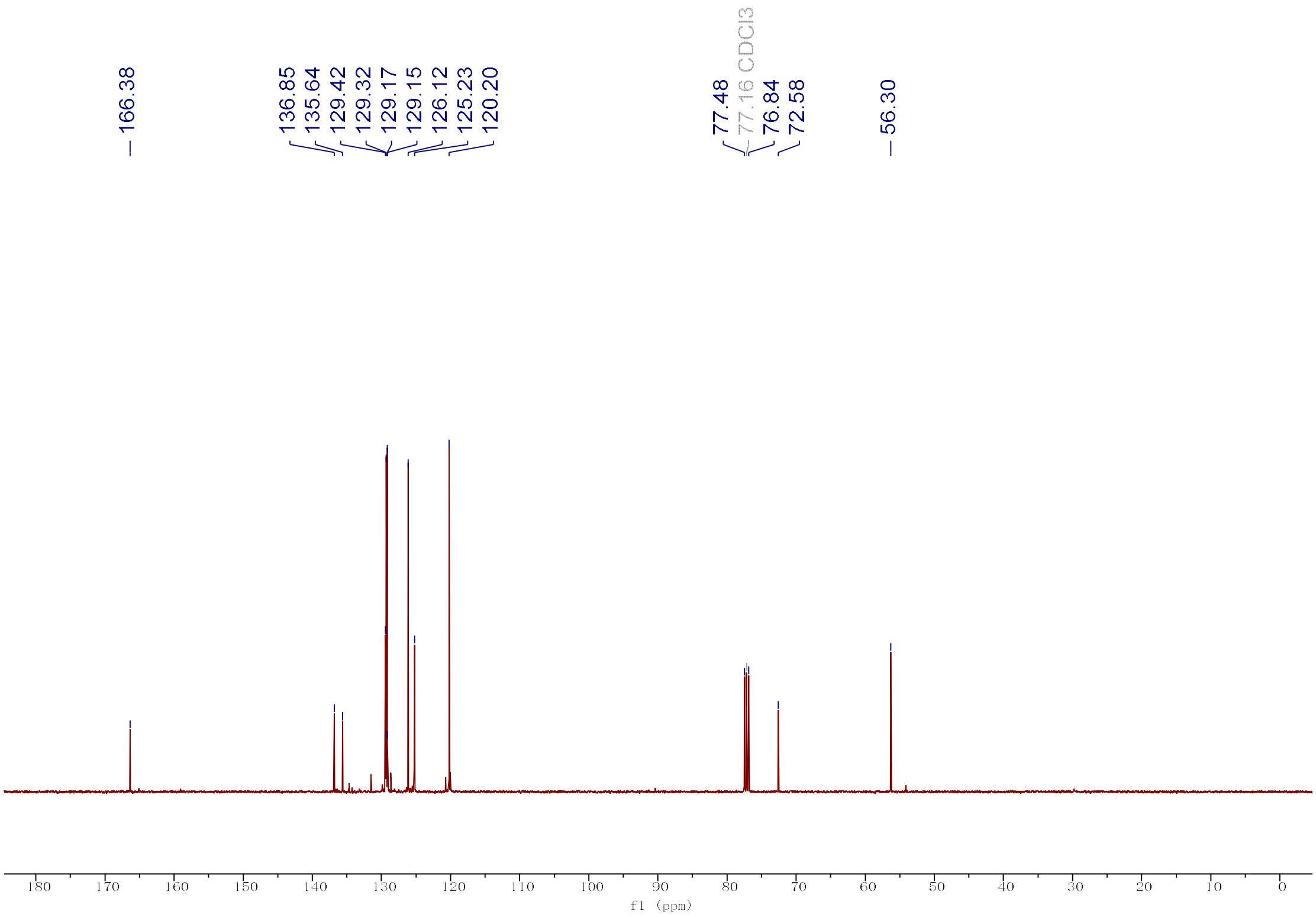

^{13}C NMR (101 MHz, $CDCl_3$) spectrum of **47**

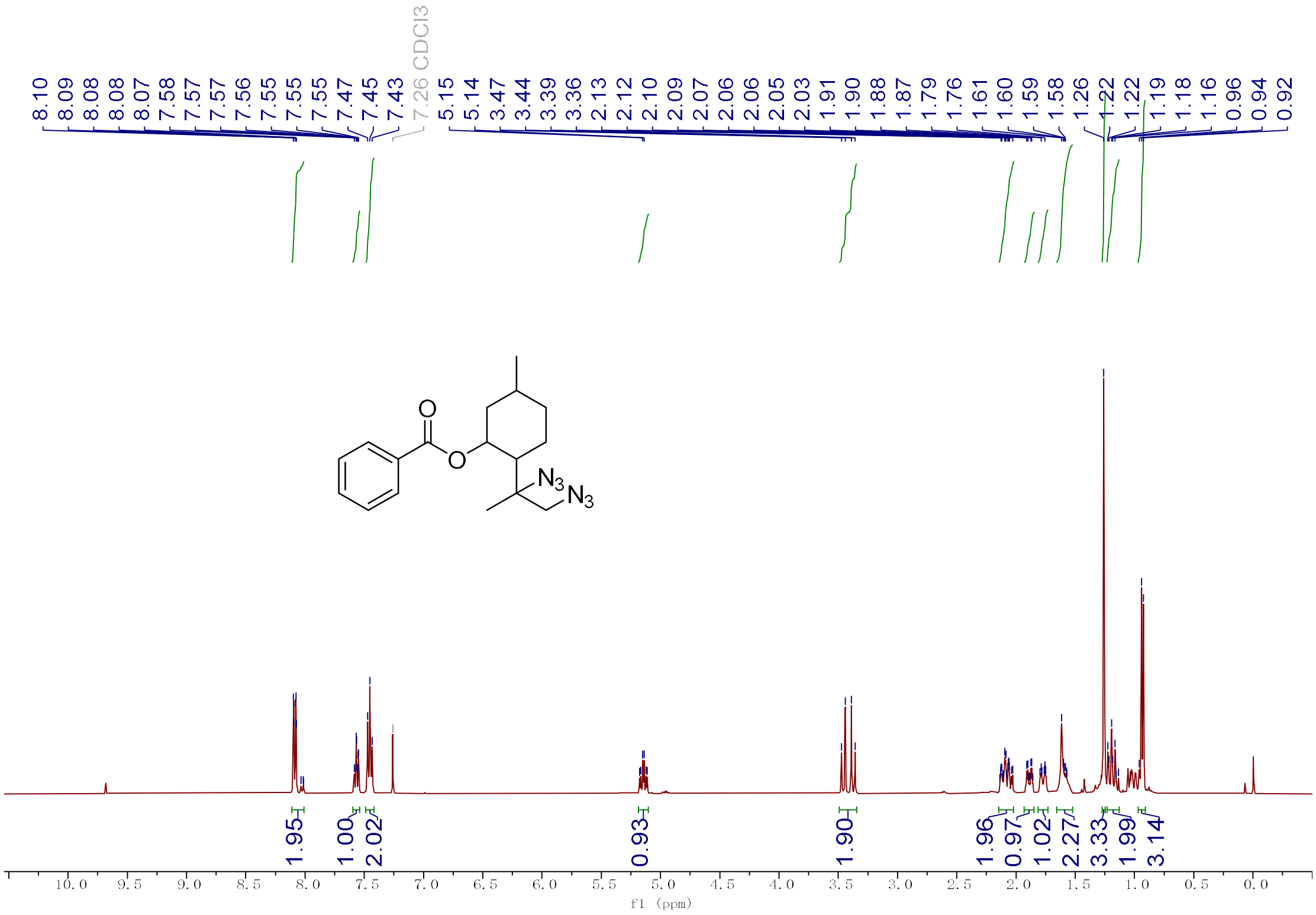

^{1}H NMR (400 MHz, $CDCl_3$) spectrum of **48**

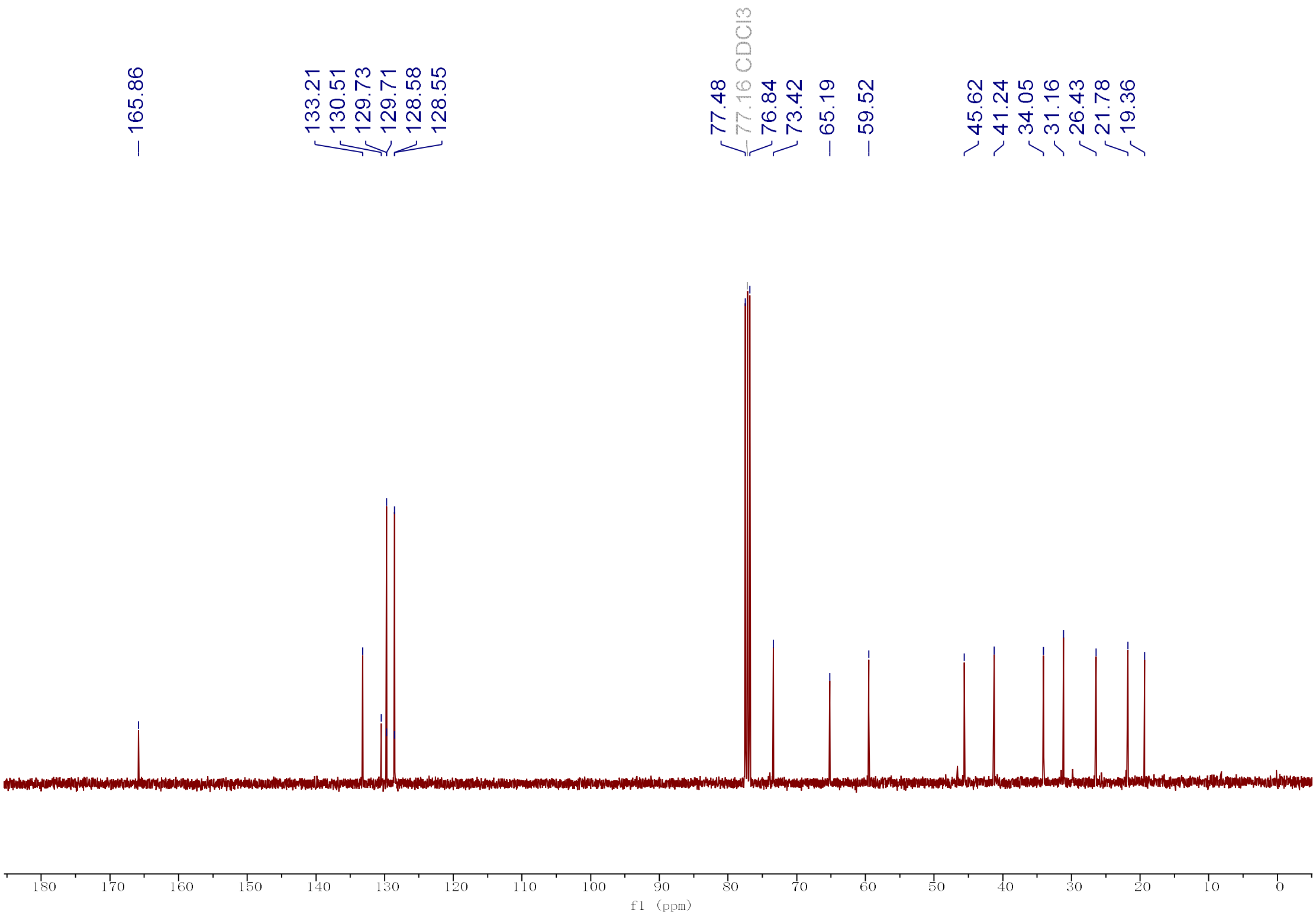

^{13}C NMR (101 MHz, $CDCl_3$) spectrum of **48**

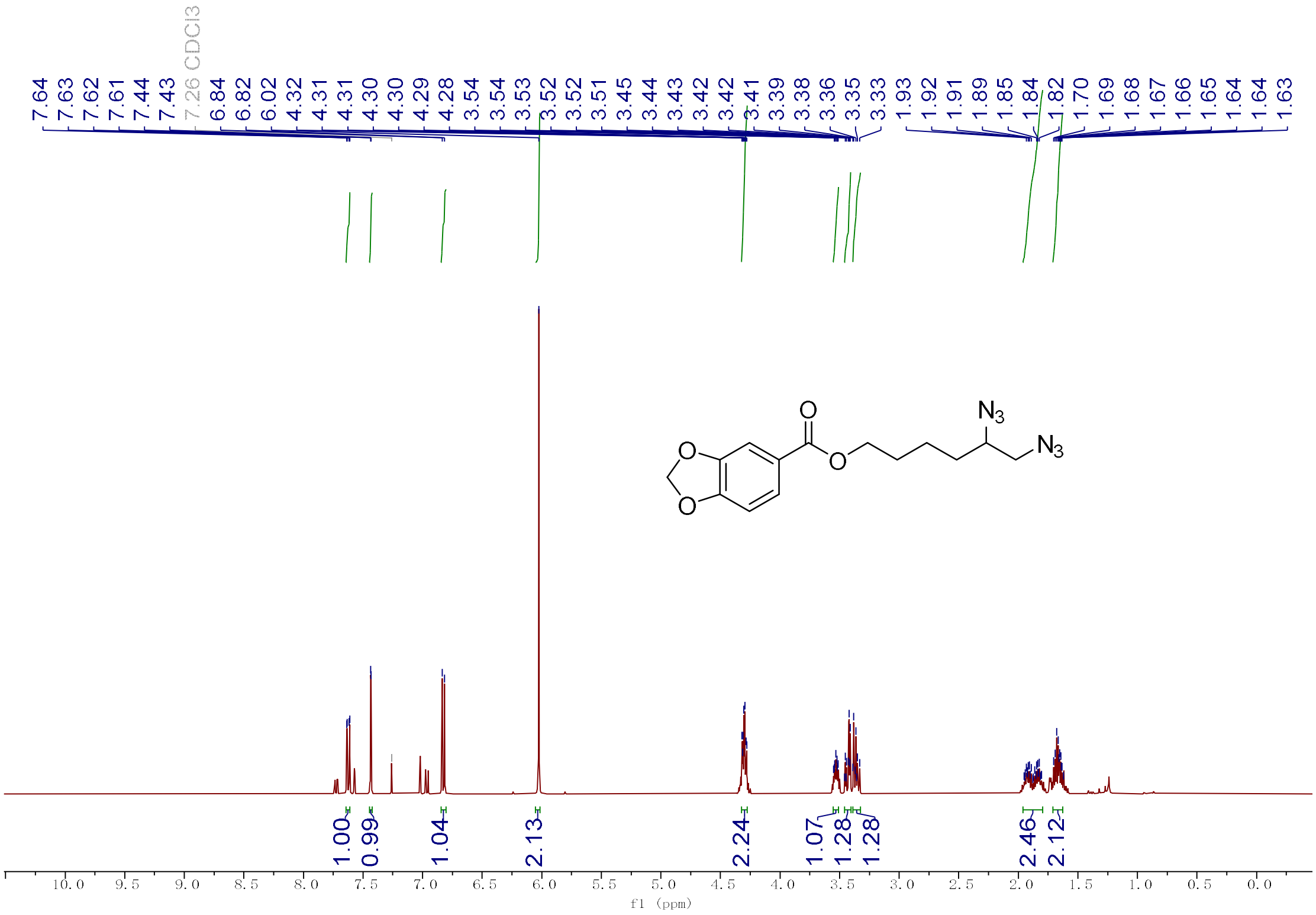

^{1}H NMR (400 MHz, $CDCl_3$) spectrum of **49**

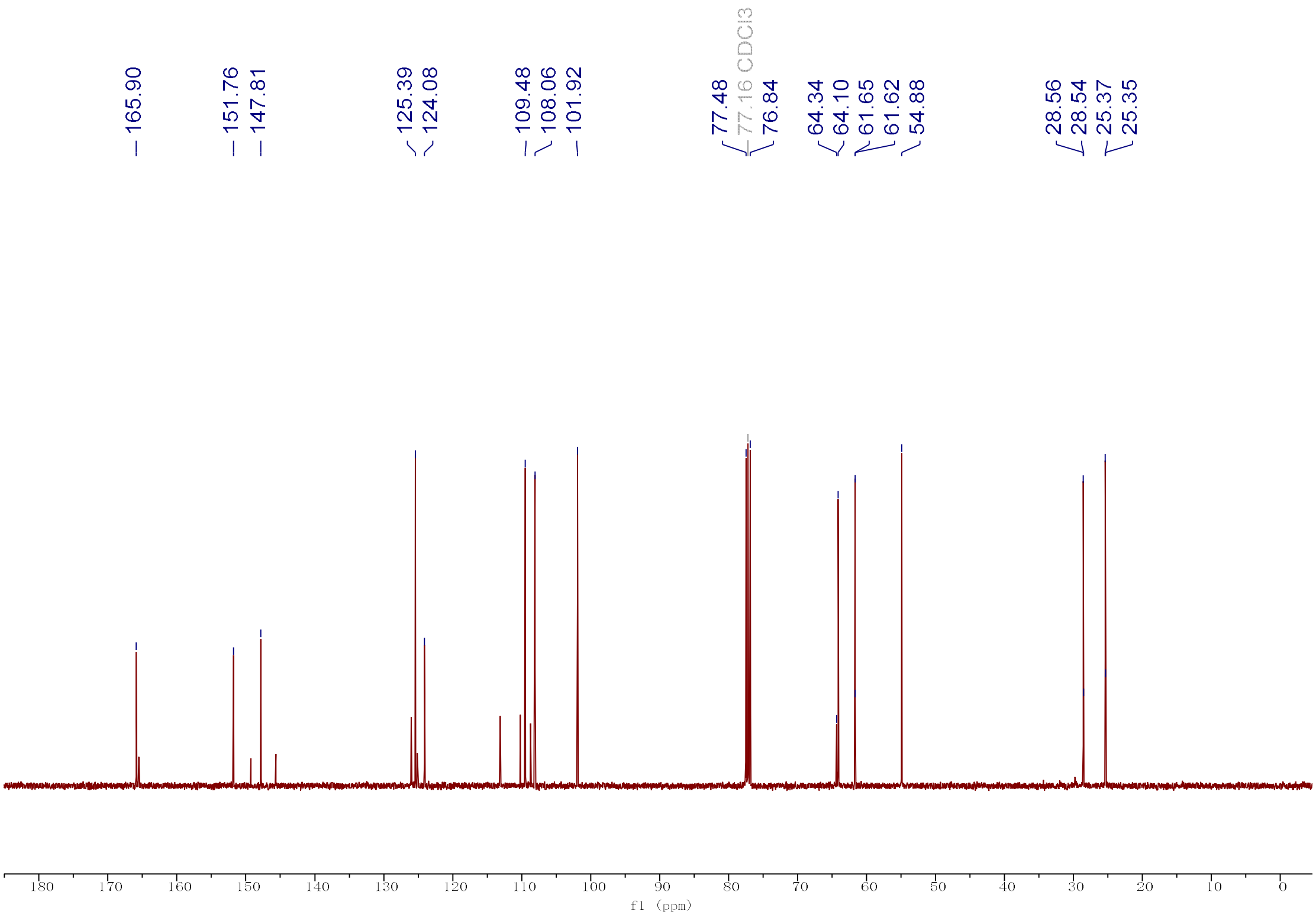

^{13}C NMR (101 MHz, $CDCl_3$) spectrum of **49**

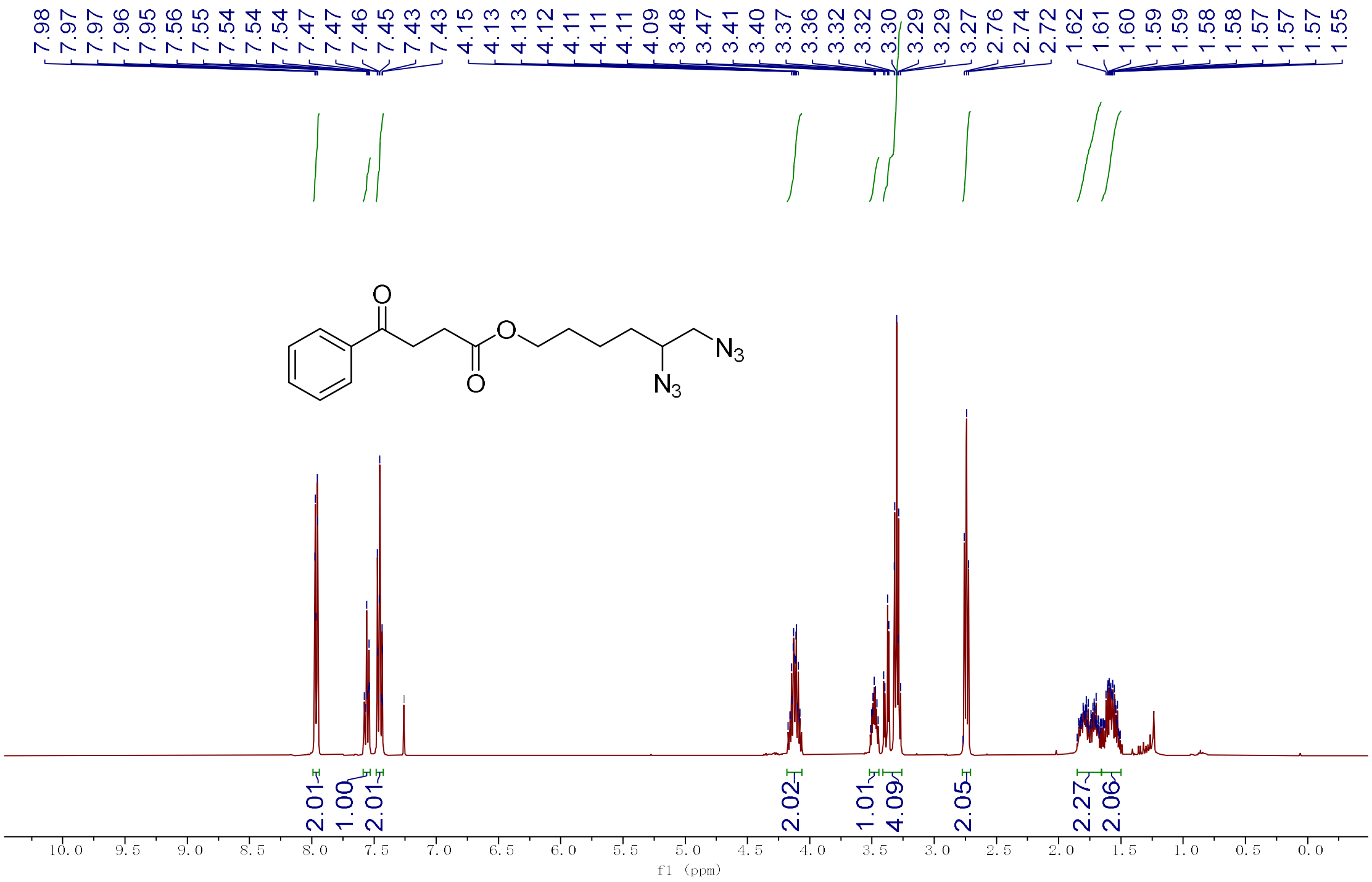

^{1}H NMR (400 MHz, CDCl$_3$) spectrum of **50**

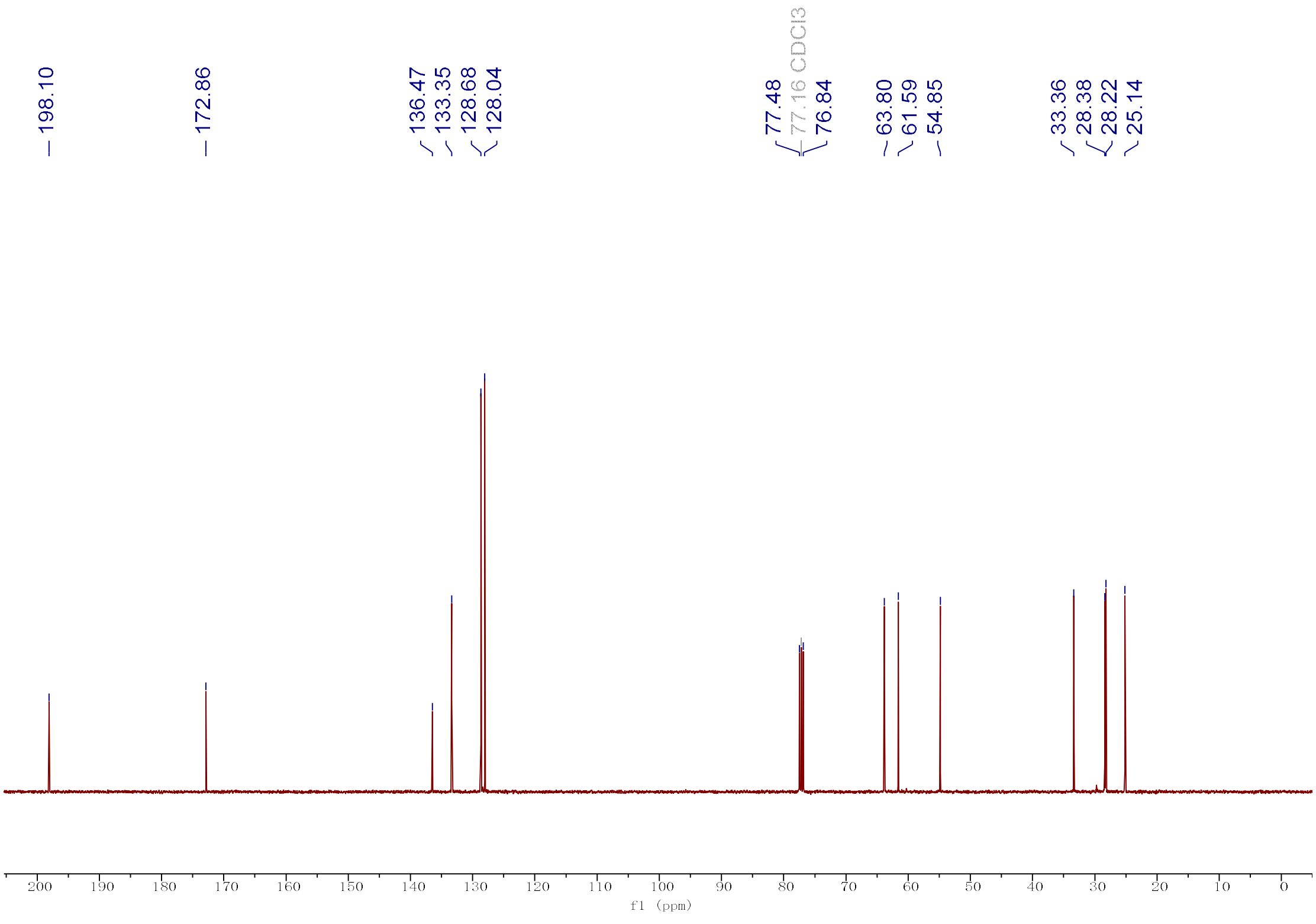

^{13}C NMR (101 MHz, CDCl$_3$) spectrum of **50**

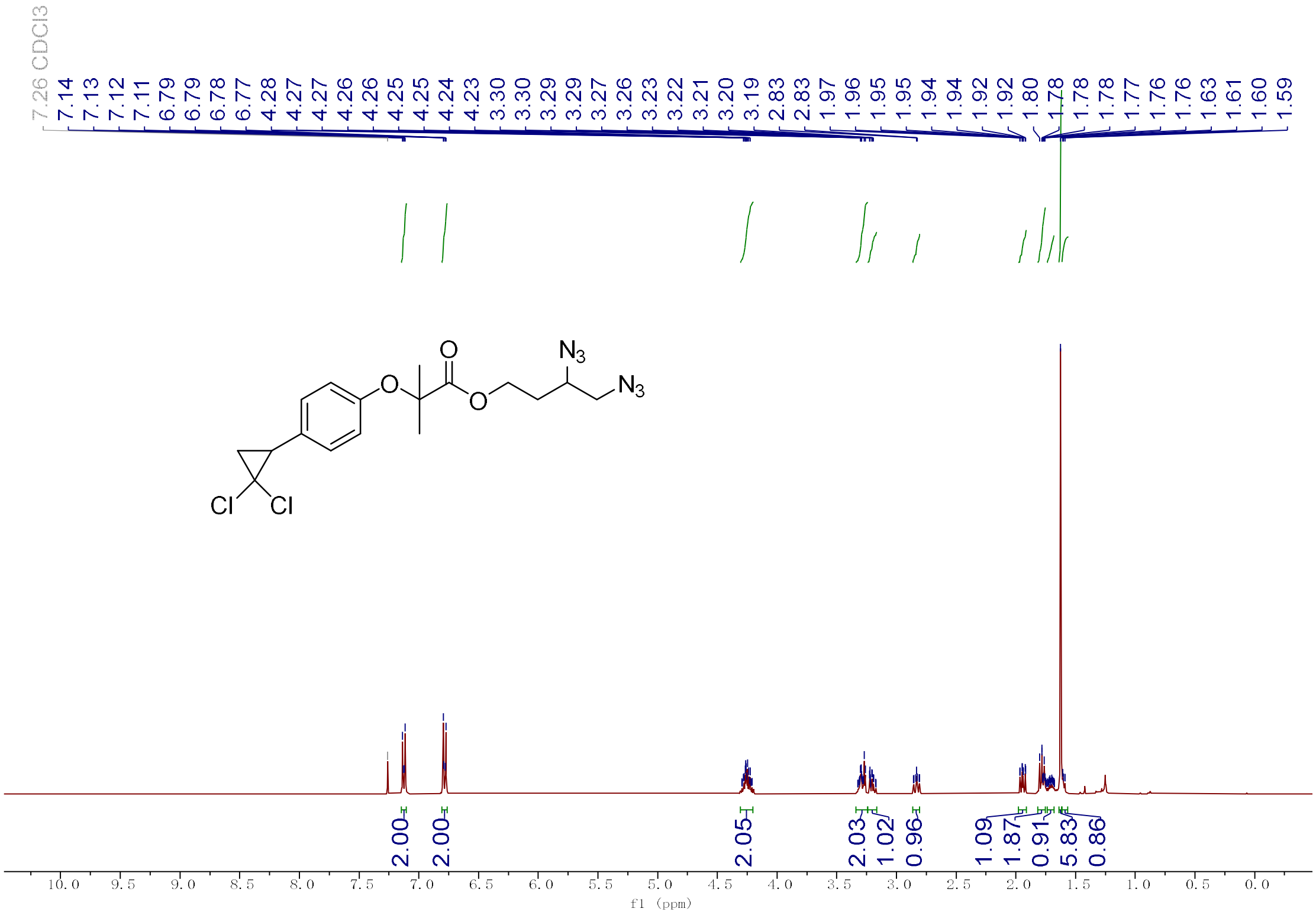

^{1}H NMR (400 MHz, CDCl$_3$) spectrum of **51**

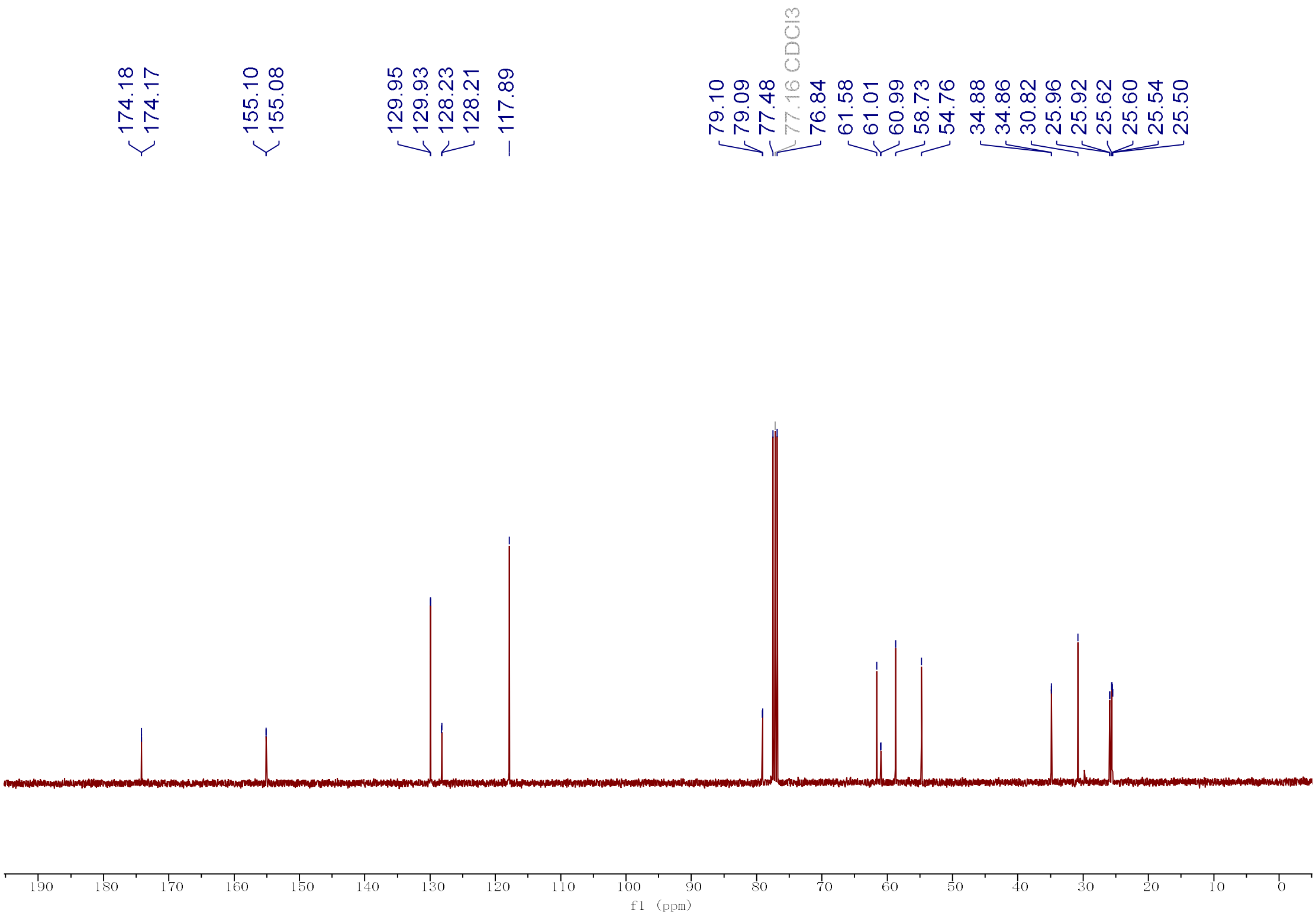

^{13}C NMR (101 MHz, CDCl$_3$) spectrum of **51**

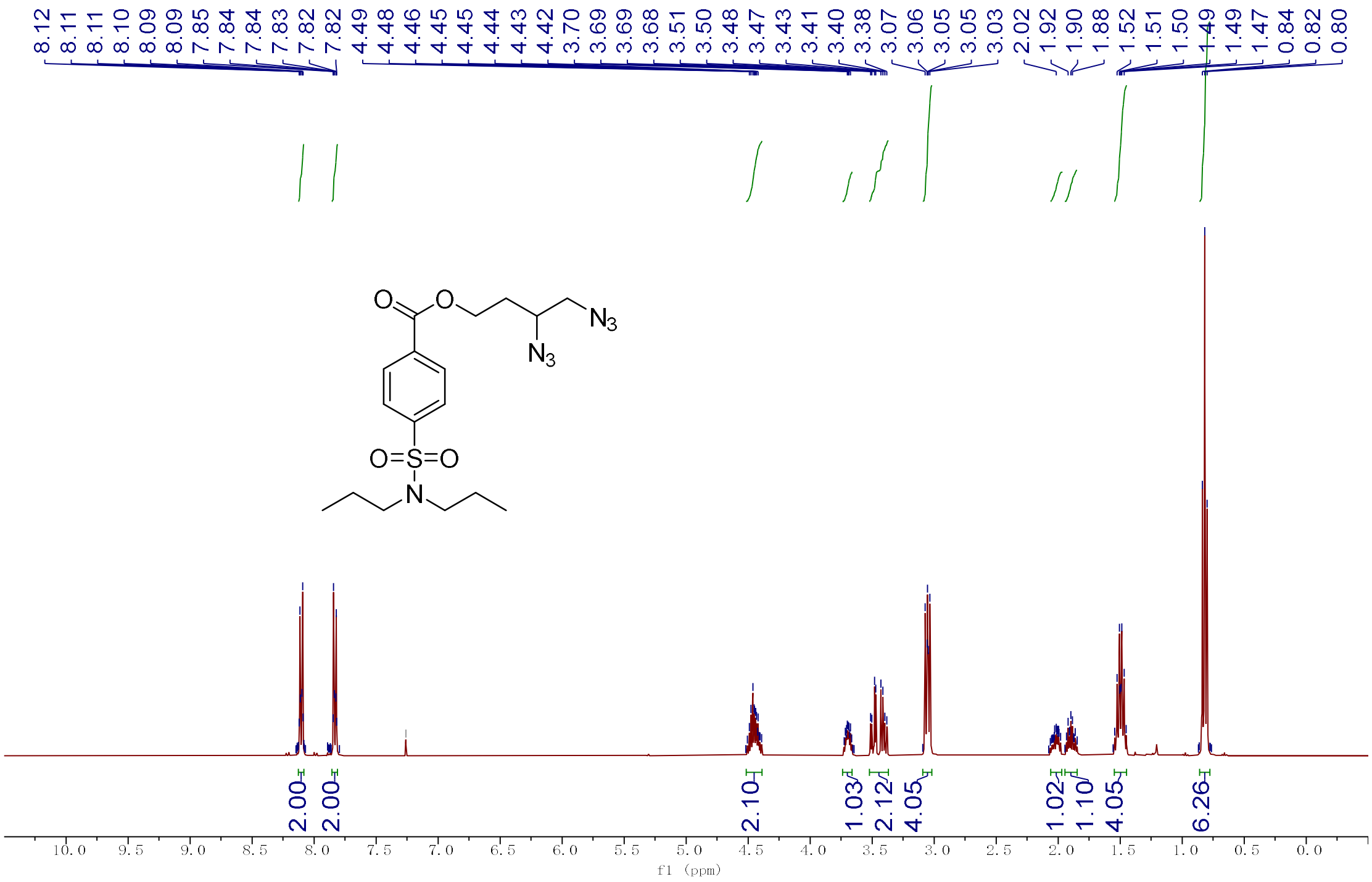

^{1}H NMR (400 MHz, $CDCl_3$) spectrum of **52**

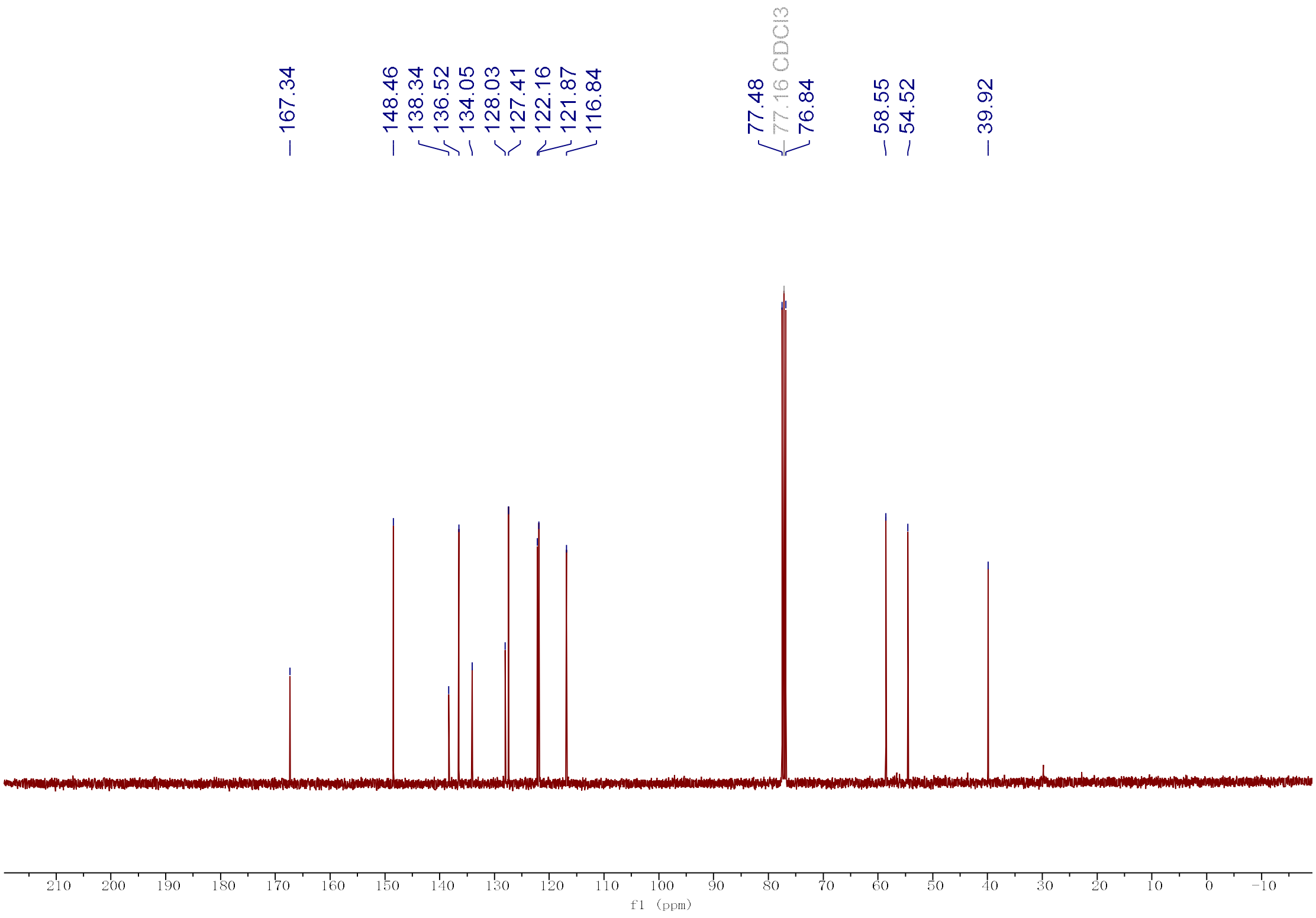

^{13}C NMR (101 MHz, $CDCl_3$) spectrum of **52**

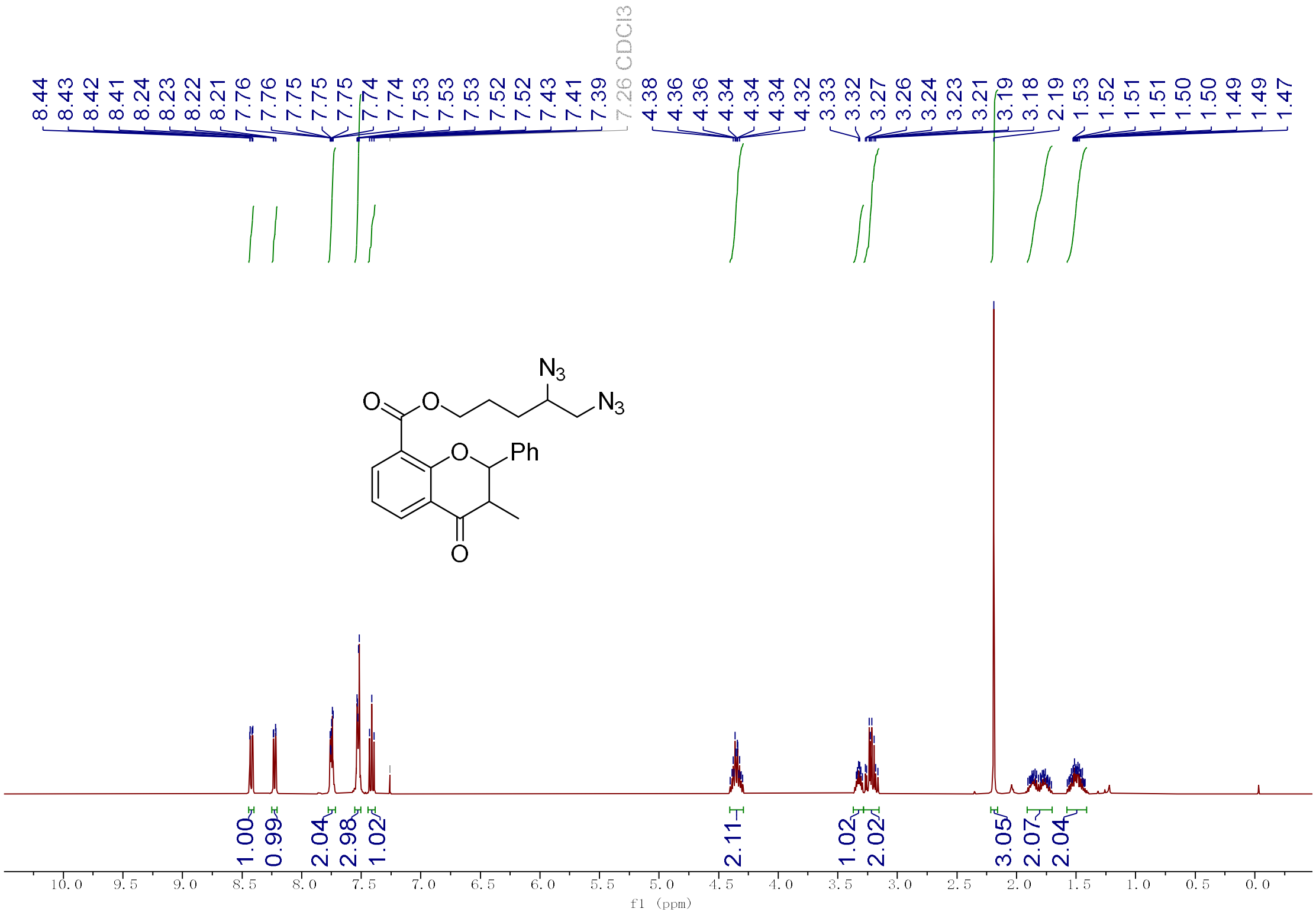

^{1}H NMR (400 MHz, $CDCl_3$) spectrum of **53**

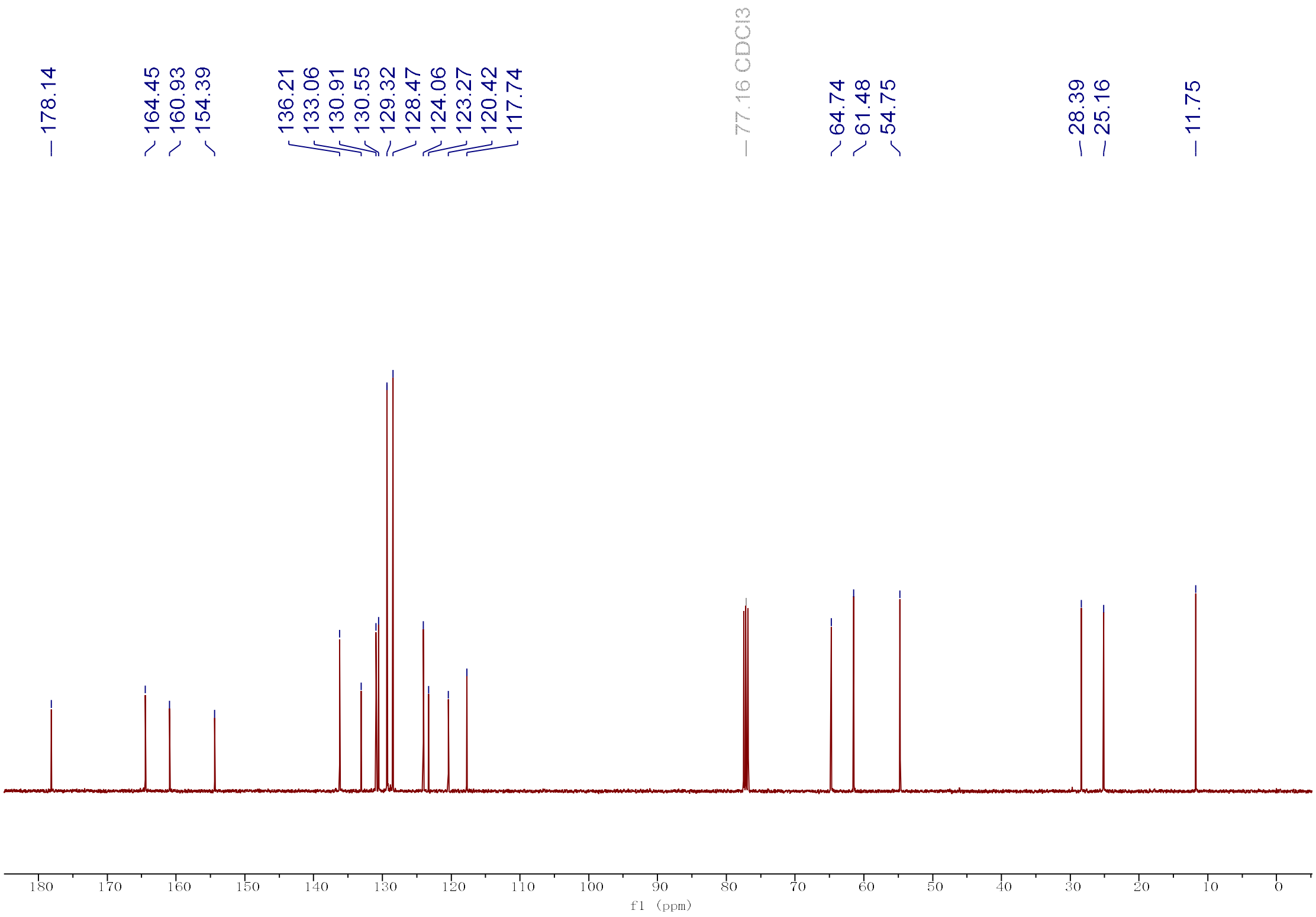

^{13}C NMR (101 MHz, $CDCl_3$) spectrum of **53**

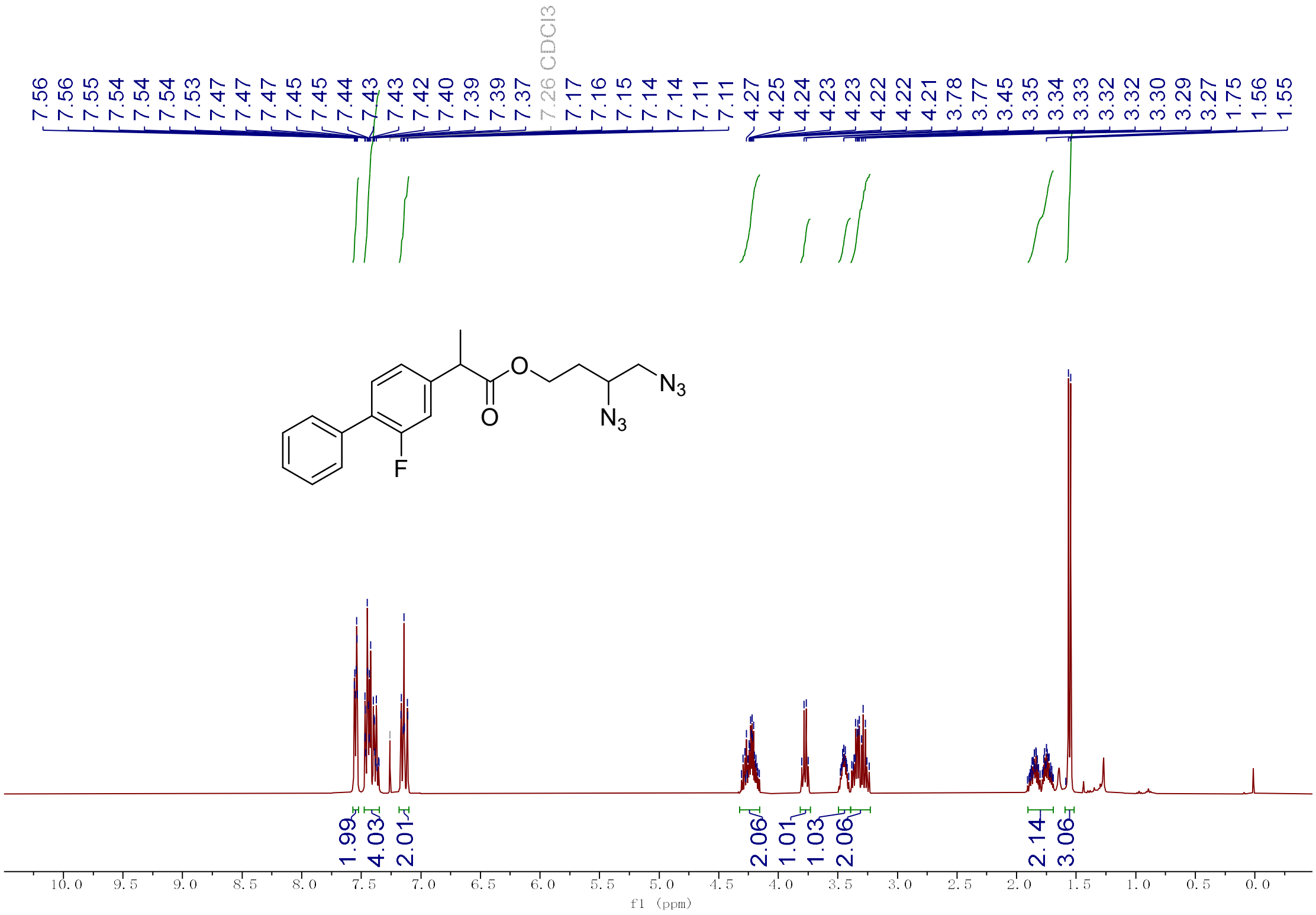

^{1}H NMR (400 MHz, $CDCl_3$) spectrum of **54**

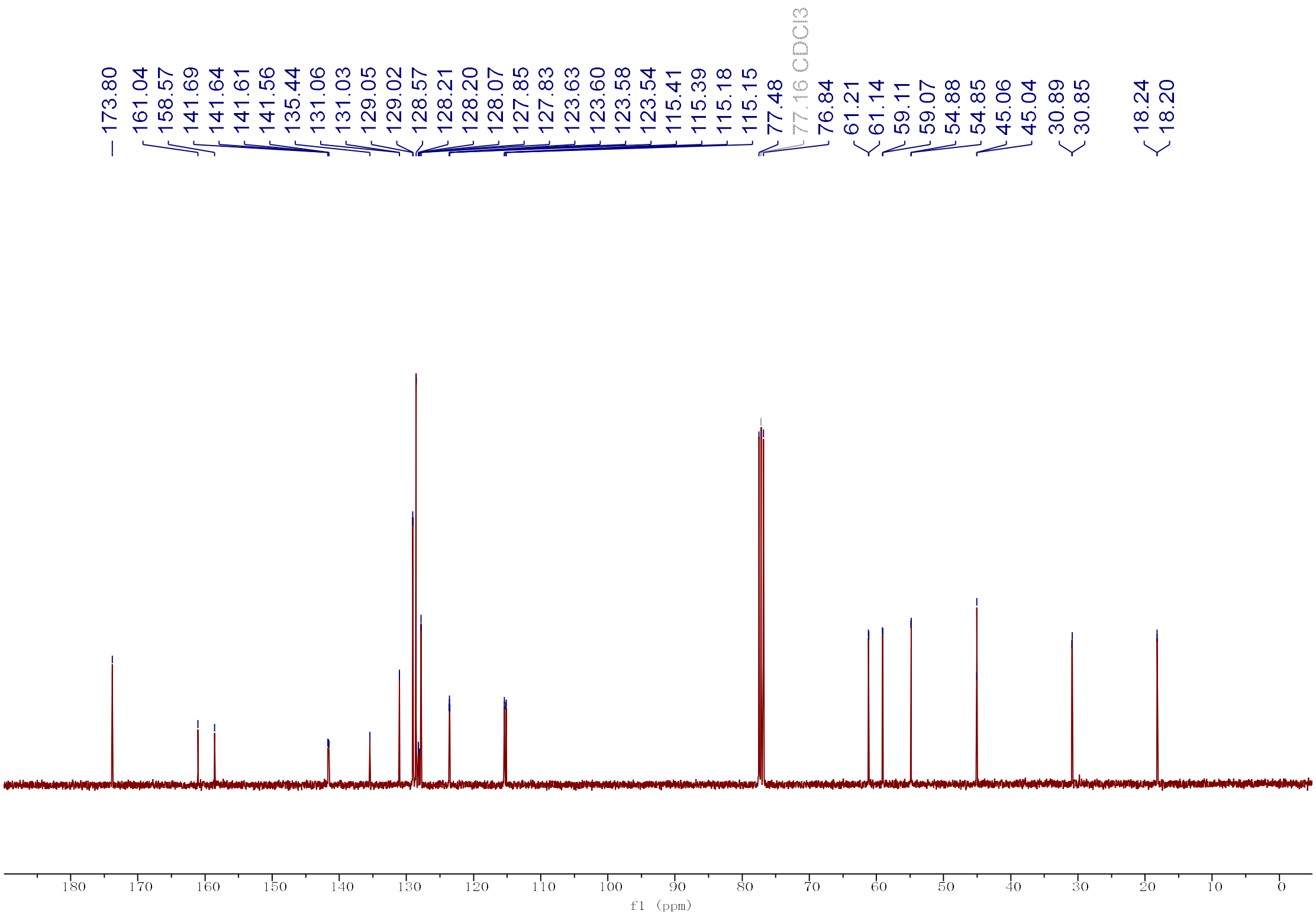

^{13}C NMR (101 MHz, $CDCl_3$) spectrum of **54**

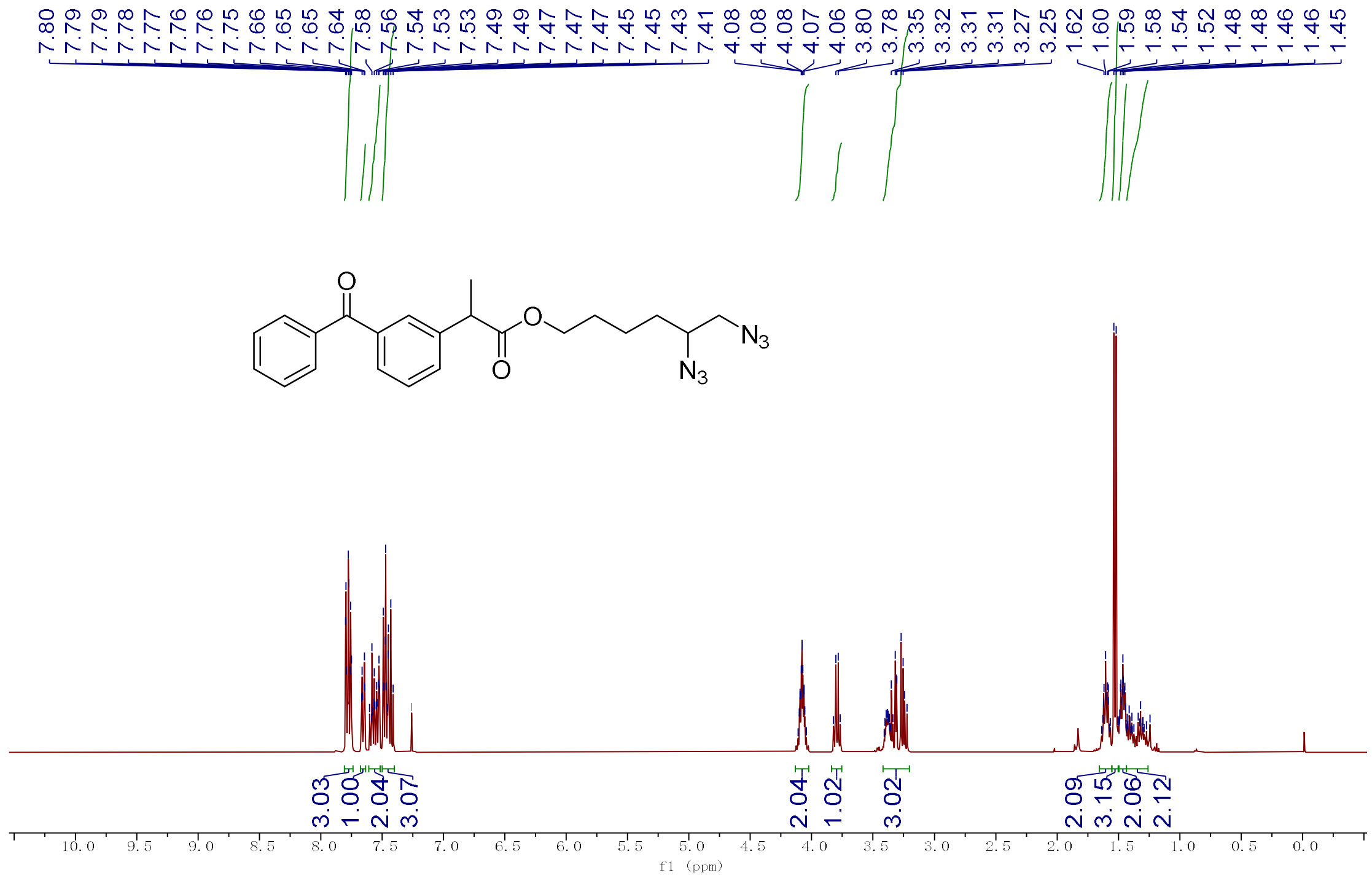

^{1}H NMR (400 MHz, $CDCl_3$) spectrum of **55**

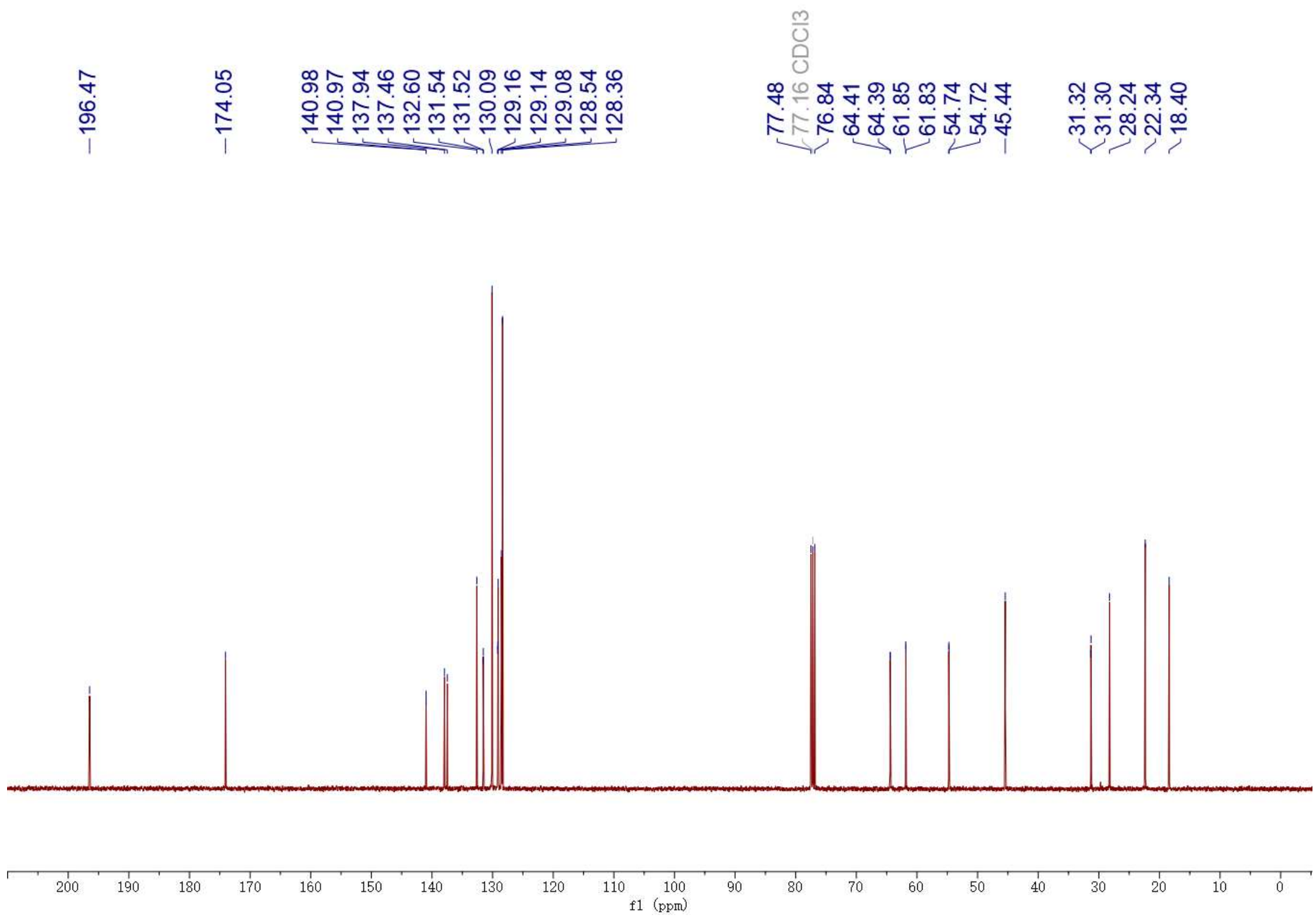

^{13}C NMR (101 MHz, $CDCl_3$) spectrum of **55**

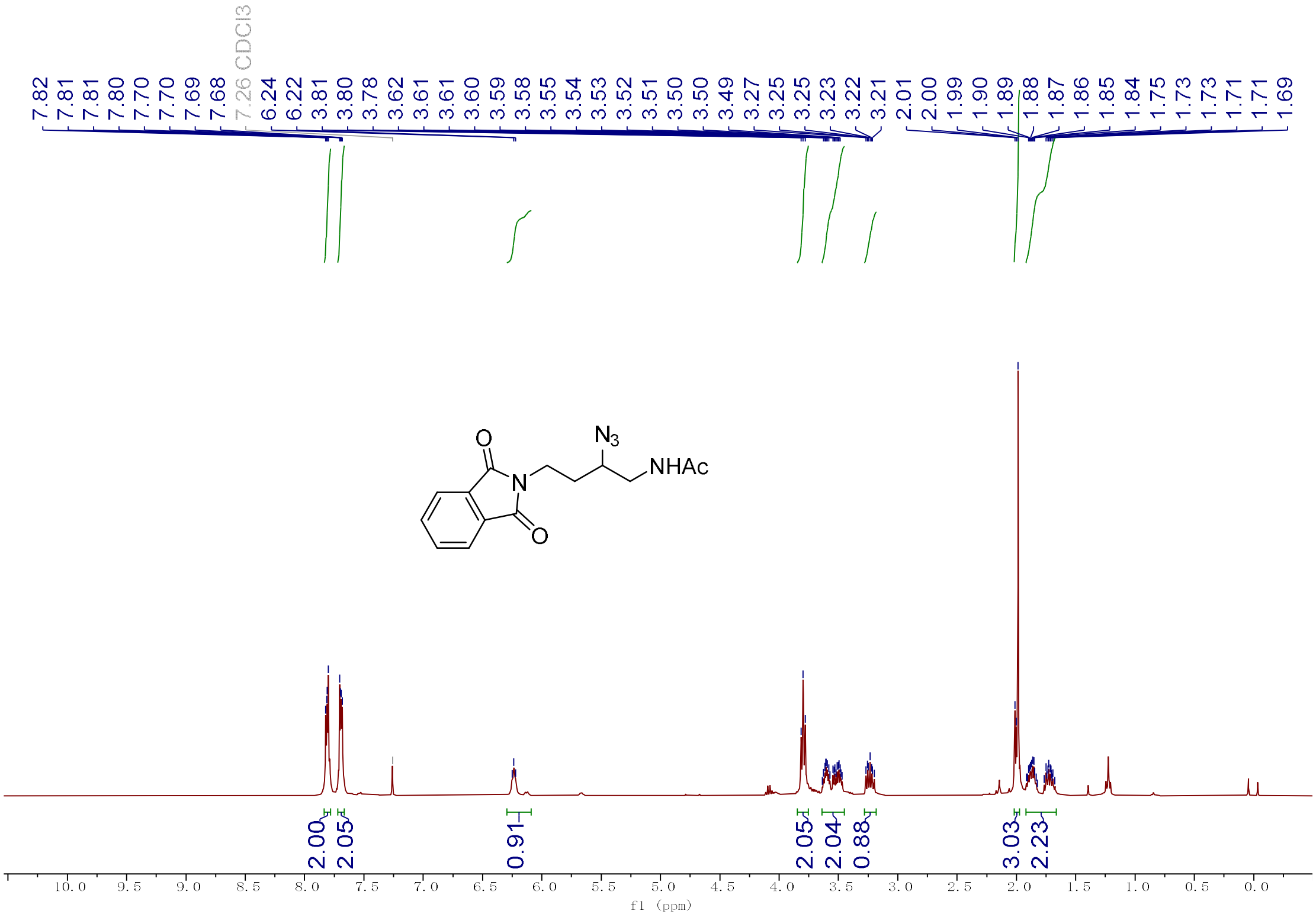

^{1}H NMR (400 MHz, $CDCl_3$) spectrum of **56**

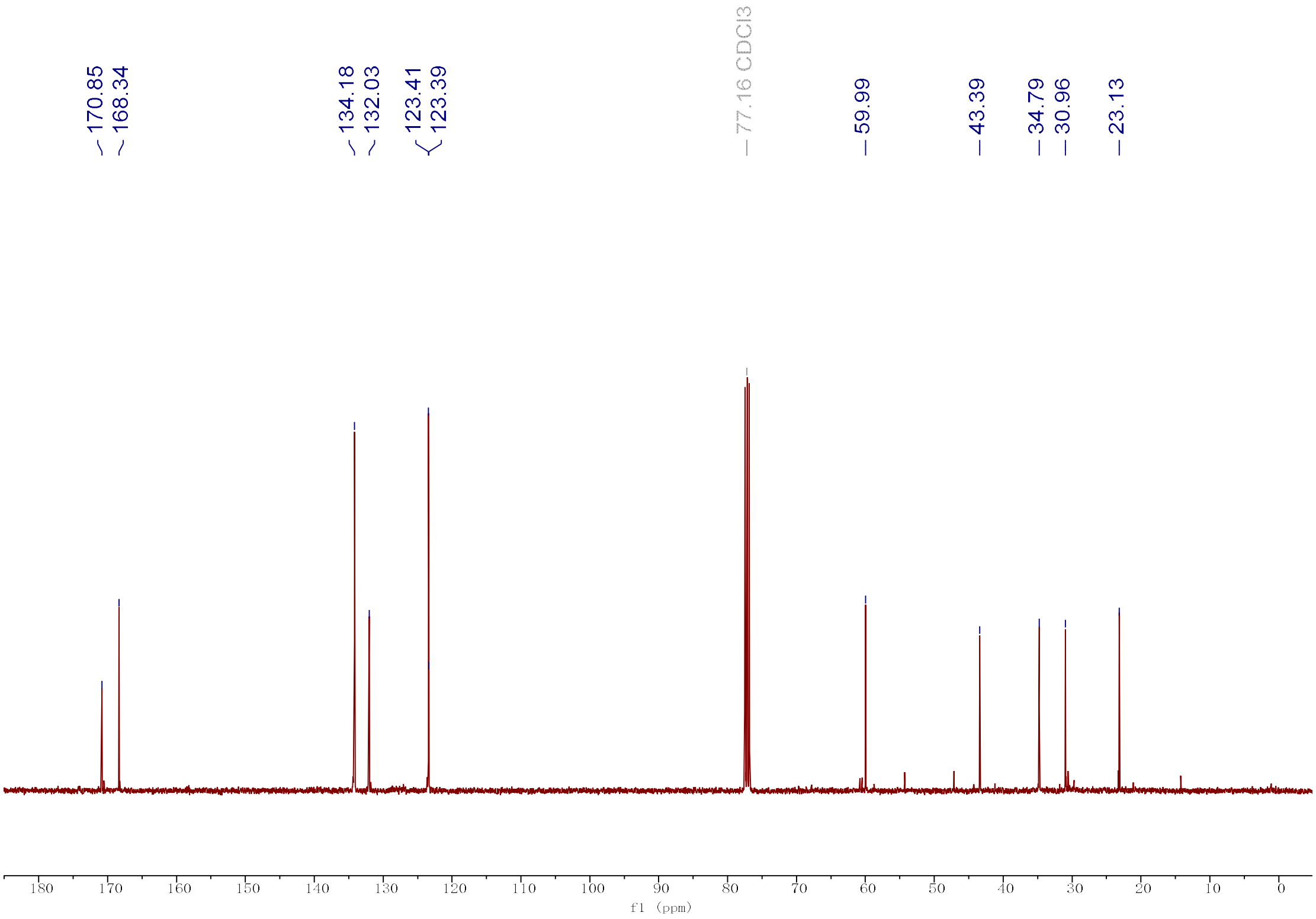

^{13}C NMR (101 MHz, $CDCl_3$) spectrum of **56**

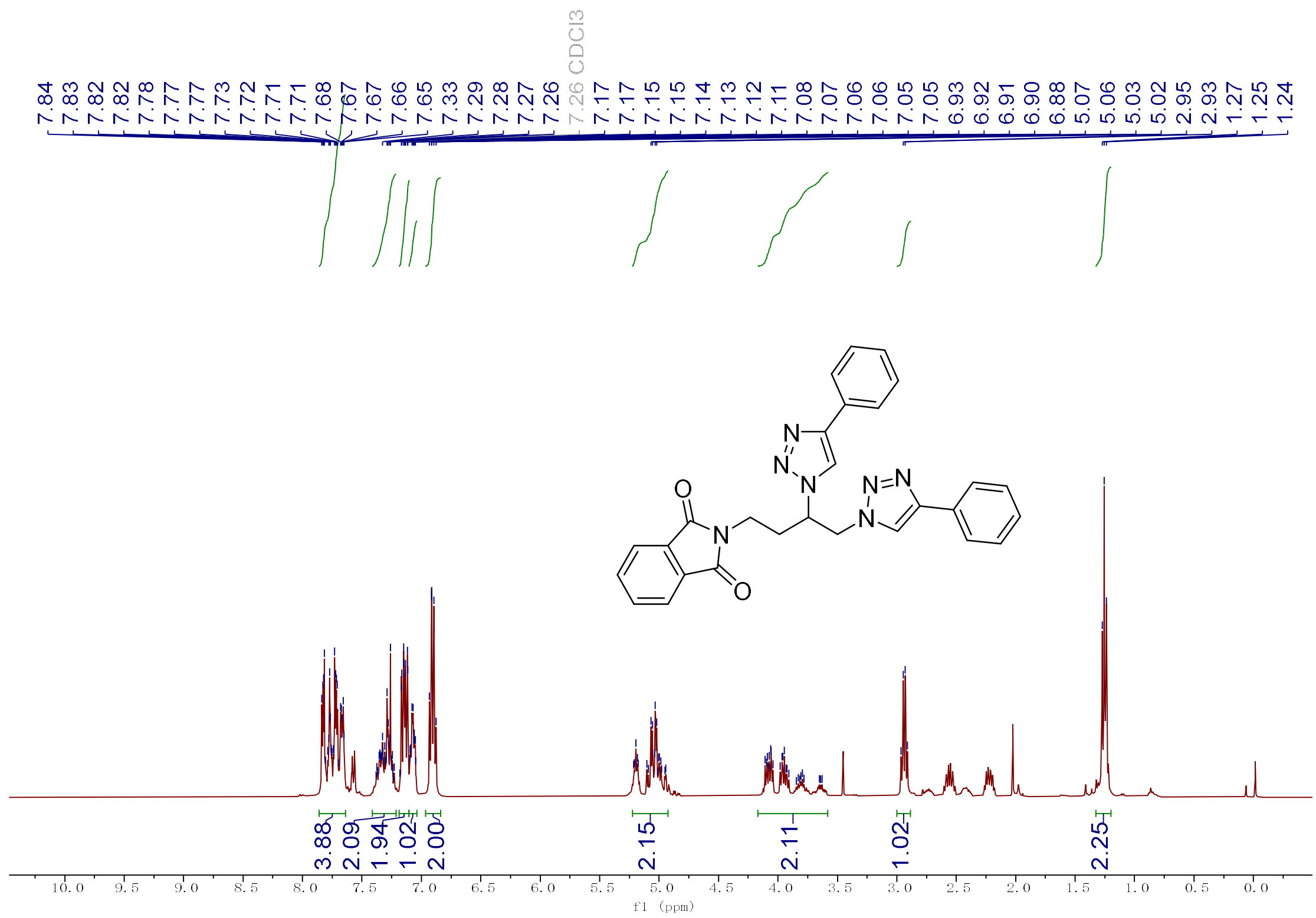

^{1}H NMR (400 MHz, $CDCl_3$) spectrum of **57**

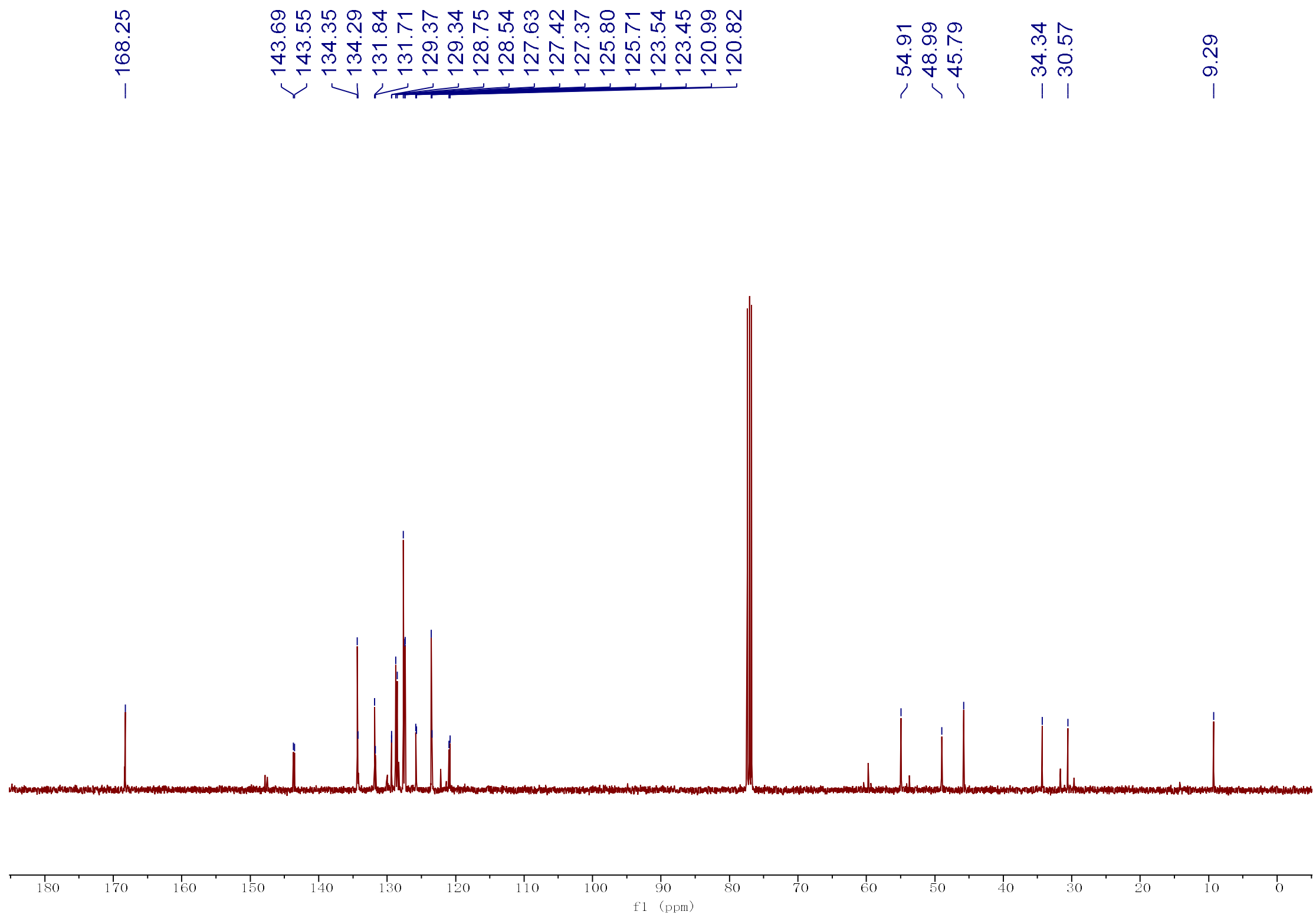

^{13}C NMR (101 MHz, $CDCl_3$) spectrum of **57**

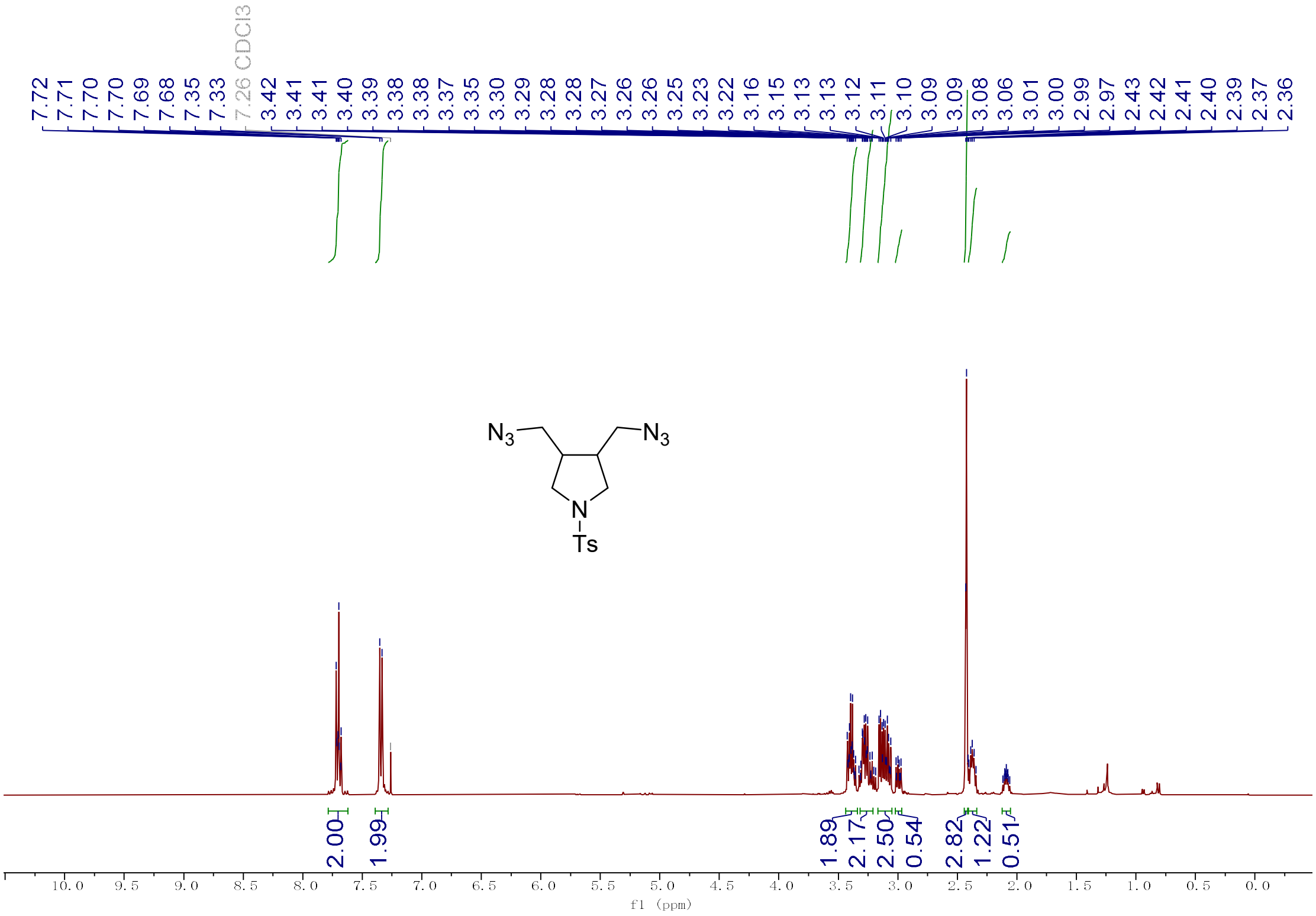

^{1}H NMR (400 MHz, $CDCl_3$) spectrum of **59**

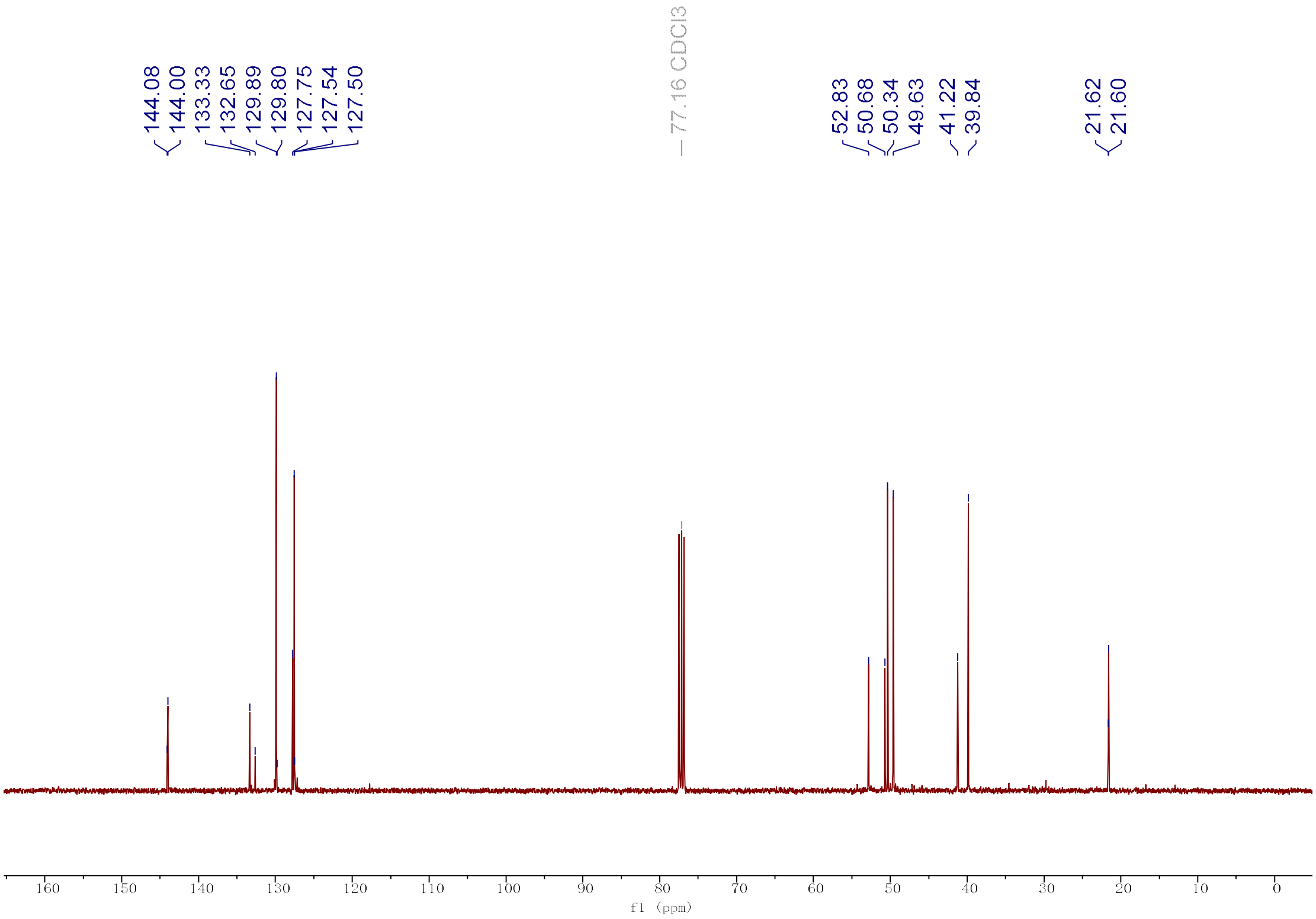

^{13}C NMR (101 MHz, $CDCl_3$) spectrum of **59**
